# Nonacethrene Unchained: A Cascade to Chiral Contorted Conjugated Hydrocarbon with Two sp^3^-Defects

**DOI:** 10.1021/jacsau.2c00190

**Published:** 2022-07-09

**Authors:** Daniel Čavlović, Daniel Häussinger, Olivier Blacque, Prince Ravat, Michal Juríček

**Affiliations:** † Department of Chemistry, University of Zurich, Winterthurerstrasse 190, 8057 Zurich, Switzerland; ‡ Department of Chemistry, University of Basel, St. Johanns-Ring 19, 4056 Basel, Switzerland; § Institute of Organic Chemistry, University of Würzburg, Am Hubland, 97074 Würzburg, Germany

**Keywords:** cethrene, helical diradicaloid, π-radical cascade, reaction mechanism, chiral contorted hydrocarbon, sp^3^-defect, circularly polarized luminescence

## Abstract

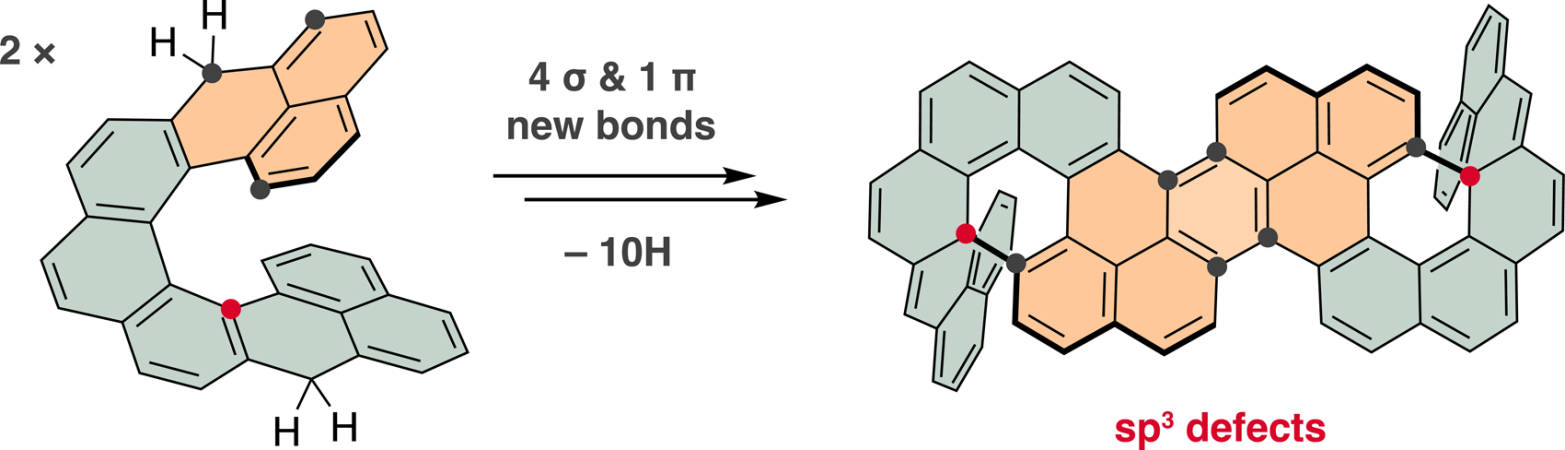

We demonstrate that structurally complex carbon nanostructures can be achieved via a synthetic approach that capitalizes on a π-radical reaction cascade. The cascade is triggered by oxidation of a dihydro precursor of helical diradicaloid nonacethrene to give a chiral contorted polycyclic aromatic hydrocarbon named hypercethrene. In this ten-electron oxidation process, four σ-bonds, one π-bond, and three six-membered rings are formed in a sequence of up to nine steps to yield a 72-carbon-atom warped framework, comprising two configurationally locked [7]helicene units, a fluorescent peropyrene unit, and two precisely installed sp^3^-defects. The key intermediate in this cascade is a closed nonacethrene derivative with one quaternary sp^3^-center, presumably formed via an electrocyclic ring closure of nonacethrene, which, when activated by oxidation, undergoes a reaction cascade analogous to the oxidative dimerization of phenalenyl to peropyrene. By controlling the amount of oxidant used, two intermediates and one side product could be isolated and fully characterized, including single-crystal X-ray diffraction analysis, and two intermediates were detected by electron paramagnetic resonance spectroscopy. In concert with density functional theory calculations, these intermediates support the proposed reaction mechanism. Compared to peropyrene, the absorption and emission of hypercethrene are slightly red-shifted on account of extended π-conjugation and the fluorescence quantum yield of 0.45 is decreased by a factor of ∼2. Enantiomerically enriched hypercethrene displays circularly polarized luminescence with a brightness value of 8.3 M^–1^ cm^–1^. Our results show that reactions of graphene-based π-radicals—typically considered an “undefined decomposition” of non-zero-spin materials—can be well-defined and selective, and have potential to be transformed into a step-economic synthetic method toward complex carbon nanostructures.

## Introduction

Polycyclic aromatic hydrocarbons (PAHs) with diradicaloid singlet ground state are investigated as molecular components of materials that display conductivity and magnetism.^1−4^ These properties arise from two typical characteristics of diradicaloid compounds, namely, small energy gap between the highest occupied molecular orbital (HOMO) and the lowest unoccupied molecular orbital (LUMO), and small singlet–triplet (S–T) energy gap.^4−6^ The diradical character of PAHs with a Kekulé electronic structure is achieved with quinoidal subunits, such as *ortho*- and *para*-quinodimethane (*o*-QDM and *p*-QDM, respectively), with each unit stabilizing the diradical resonance structure of a PAH by one Clar’s sextet. A large variety of Kekulé diradicaloids have been prepared by extending the structures of *o*-QDM and *p*-QDM. The classic as well as more recent examples—including Thiele’s,^7,8^ Chichibabin’s,^8−10^ and Müller’s^11^ hydrocarbons and their analogs,^12^ (peri)acenes,^13,14^ bis(phenalenyls),^15−17^ indenofluorenes,^18−20^ sigmarene,^21^ zethrenes,^22−24^ and cethrenes^25,26^—demonstrate the versatility of molecular design and properties of PAHs with singlet diradical character.

The electronic structure of Kekulé diradicaloids is of interest also from another fundamental standpoint—reactivity—as illustrated^27^ by the parent compounds *o*-QDM and *p*-QDM. Their reactivity is dual in nature, that is, it shows characteristics of closed-shell and open-shell systems. As an example, *o*-QDM undergoes thermal dimerization both via a concerted [4 + 2] cycloaddition mechanism as well as via a diradical mechanism yielding [4 + 4] cycloaddition dimer, which is formally a symmetry-forbidden process. A similar dual reactivity has recently been observed for sigmarene.^21^ In the past, various aspects of reactivity of these and analogous systems were subject to extensive investigations.^6,28−30^ In contrast, the current research is largely focused on the properties of extended diradicaloids, with efforts being made to suppress^17,30−32^ their reactivity in order to obtain stable or persistent systems. A few recent reports indicate, however, that the reactivity of “unchained” diradicaloids, often regarded as an undesired or a decomposition feature, can be utilized to create function,^25,33−37^ develop new methods,^38,39^ and deepen our chemical concepts.^40,41^

**Scheme 1:**
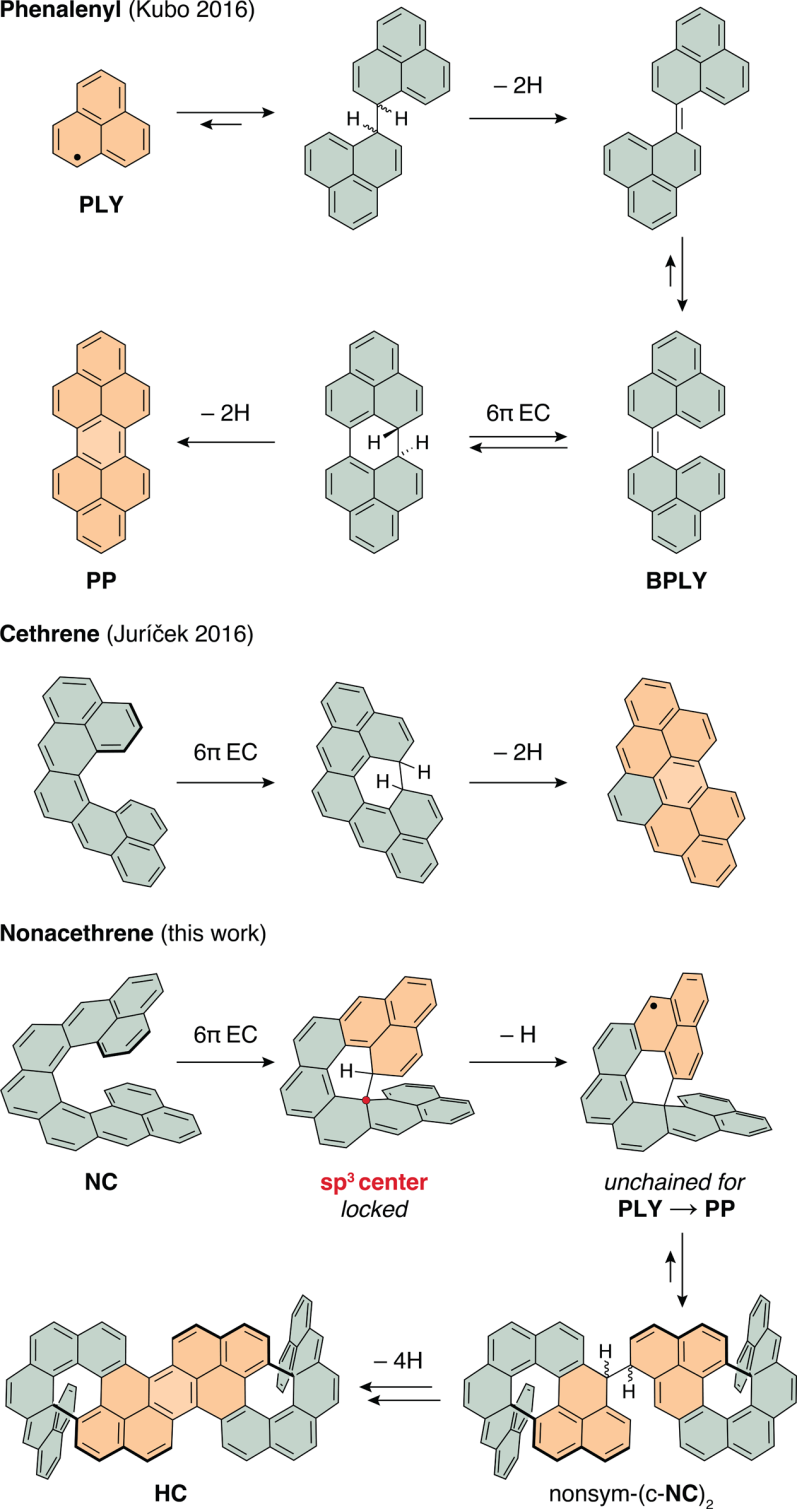
π-Radical Reactivity Overview (Top) Decomposition pathway of **PLY** to **PP** via 6π EC of **BPLY**. (Middle) Cethrene’s 6π EC followed by oxidation to a benzo-**PP** derivative. (Bottom) 6π EC of **NC** “locks” the formed intermediate with a quaternary sp^3^-center (red). Upon oxidation, this intermediate is “unchained” for the **PLY**–**PP** cascade to **HC** via a non-symmetric dimer (nonsym-(c-**NC**)_2_).

Recent examples are biphenalenylidene (**BPLY**, Scheme 1, top), an intermediate on the decomposition pathway of phenalenyl (**PLY**) reported by Kubo et al.,^28,42^ and cethrene,^26^ a helical diradicaloid developed in our laboratory (Scheme 1, middle), which undergo a thermal 6π electrocyclization (EC). Even though this process is formally symmetry-forbidden in both compounds, it proceeds rapidly at temperatures below ambient as a result of small HOMO–LUMO gaps and thus low-lying doubly excited states in **BPLY** and cethrene, which contribute to the lowering of the activation energies.

Using a dimethyl derivative of cethrene, in which the methyl groups prevent the oxidation to a flat hydrocarbon as observed for parent cethrene (Scheme 1, middle), we conceptualized^25^ the working principle of a magnetic photoswitch that could be transformed reversibly between a magnetically active diradicaloid form and a magnetically inactive closed-shell form by light. Because the S–T gap of dimethylcethrene turned out to be too high to observe an electron paramagnetic resonance (EPR) signal at room temperature,^43^ an extension of the helical backbone by two rings—to give nonacethrene (**NC**)—would overcome two challenges at once. First, the π-extended structure of **NC** should possess a significantly lower S–T gap enabling the detection of an EPR signal. Second, after the 6π EC ring closure, oxidation to a flat hydrocarbon as in the case of cethrene is not possible because the EC closure results in one quaternary sp^3^-center that “locks” the closed **NC** intermediate (Scheme 1, bottom). Contrary to our expectations, the closed intermediate could not be isolated or detected because it readily oxidizes to a radical that is poised to undergo the **PLY**–**PP** transformation. Here, we present the study of this unexpected reaction cascade, including mechanistic insights as well as characterization of two intermediates and a structurally complex final product, a chiral contorted conjugated hydrocarbon with two quaternary sp^3^-defects, named hypercethrene (**HC**; Scheme 1, bottom).

## Results and Discussion

### Synthesis

**Scheme 2:**
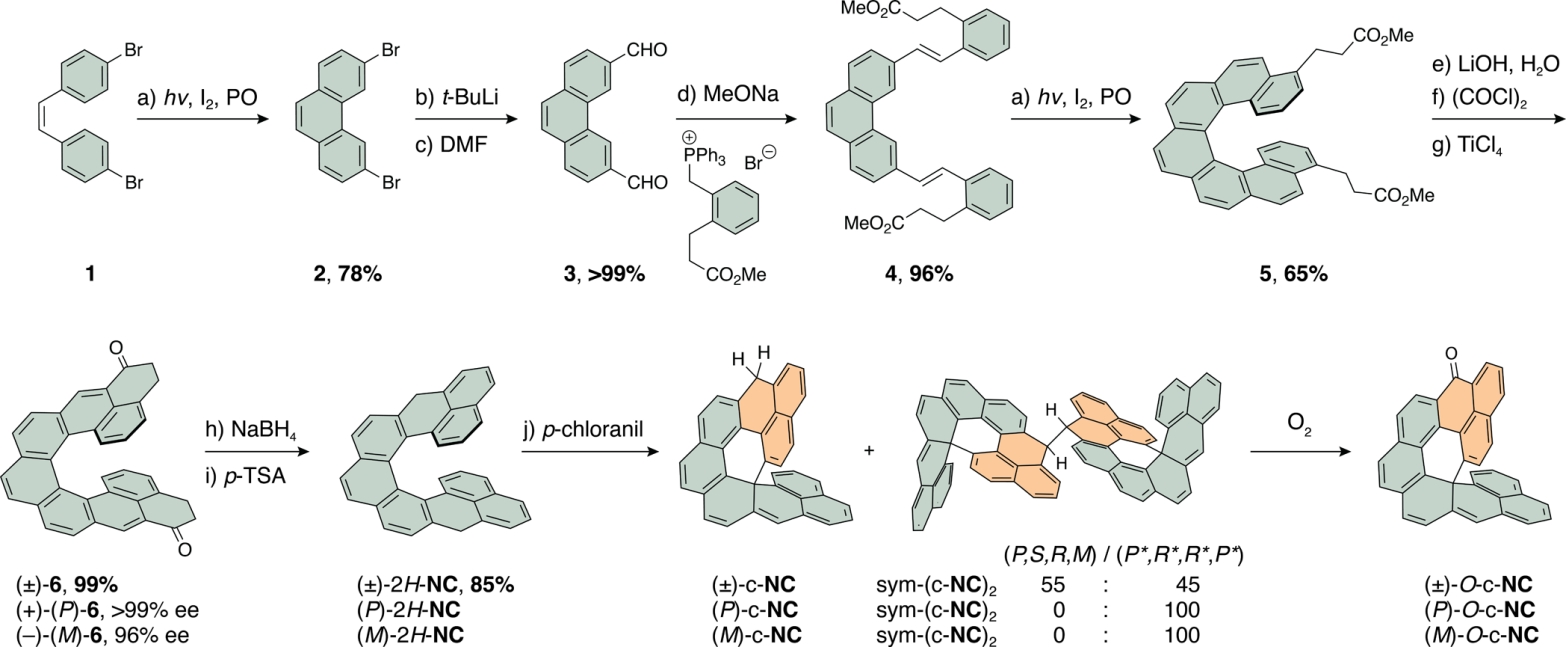
Synthesis of Dihydro Precursor of Nonacethrene (2*H*-NC) and its Oxidation to *O*-c-NC Detailed reaction conditions: (a) *h*ν, I_2_, propylene oxide, toluene, 20 °C, 14 h; (b) *t*-BuLi, THF, −78 °C, 0.5 h; (c) DMF, −78 °C to RT, 1 h; (d) MeONa, (2-(3-methoxy-3-oxopropyl)benzyl)triphenylphosphonium bromide, THF, RT, 12 h; (e) LiOH, THF/H_2_O 10:1, reflux, 16 h; (f) (COCl)_2_, 65 °C, 2 h; (g) TiCl_4_, CH_2_Cl_2_, −40 to −25 °C, 5 h; (h) NaBH_4_, CH_2_Cl_2_/EtOH 2:1, RT, 1 h; (i) *p*-TSA, toluene, 90 °C, 5 min; (j) *p*-chloranil, benzene-*d*_6_, RT, 16 h. PO = propylene oxide, THF = tetrahydrofuran, DMF = *N*,*N*′-dimethylformamide, RT = room temperature, *p*-TSA = *p*-toluenesulfonic acid.

The dihydro precursor of **NC**, 2*H*-**NC**, was synthesized starting from commercially available **1** (Scheme 2). Alternatively, **1** can be freshly prepared in one step as described in the literature.^44,45^ The first step toward 2*H*-**NC** is a photocyclodehydrogenation of **1** to obtain 3,7-dibromophenanthrene (**2**) in 78% yield, which can be performed at high concentrations (>2 mM) due to the high photostability of the starting material and the product. In the next step, a twofold lithium–halogen exchange^46^ followed by a formylation gave, after aqueous acidic workup, dialdehyde **3** in >99% yield. Then, a Wittig reaction afforded an isomeric mixture^47^ of **4**, which was subjected to another twofold photocyclodehydrogenation to yield helicene **5** in 65% yield over the two steps. The ester moieties in **5** were hydrolyzed under basic conditions to a give a diacid intermediate, which was transformed into the corresponding acyl chloride intermediate. Subsequent intramolecular Friedel–Crafts acylation with TiCl_4_^48^ resulted in diketone **6** in 99% yield over the three steps.

**Figure 1:**
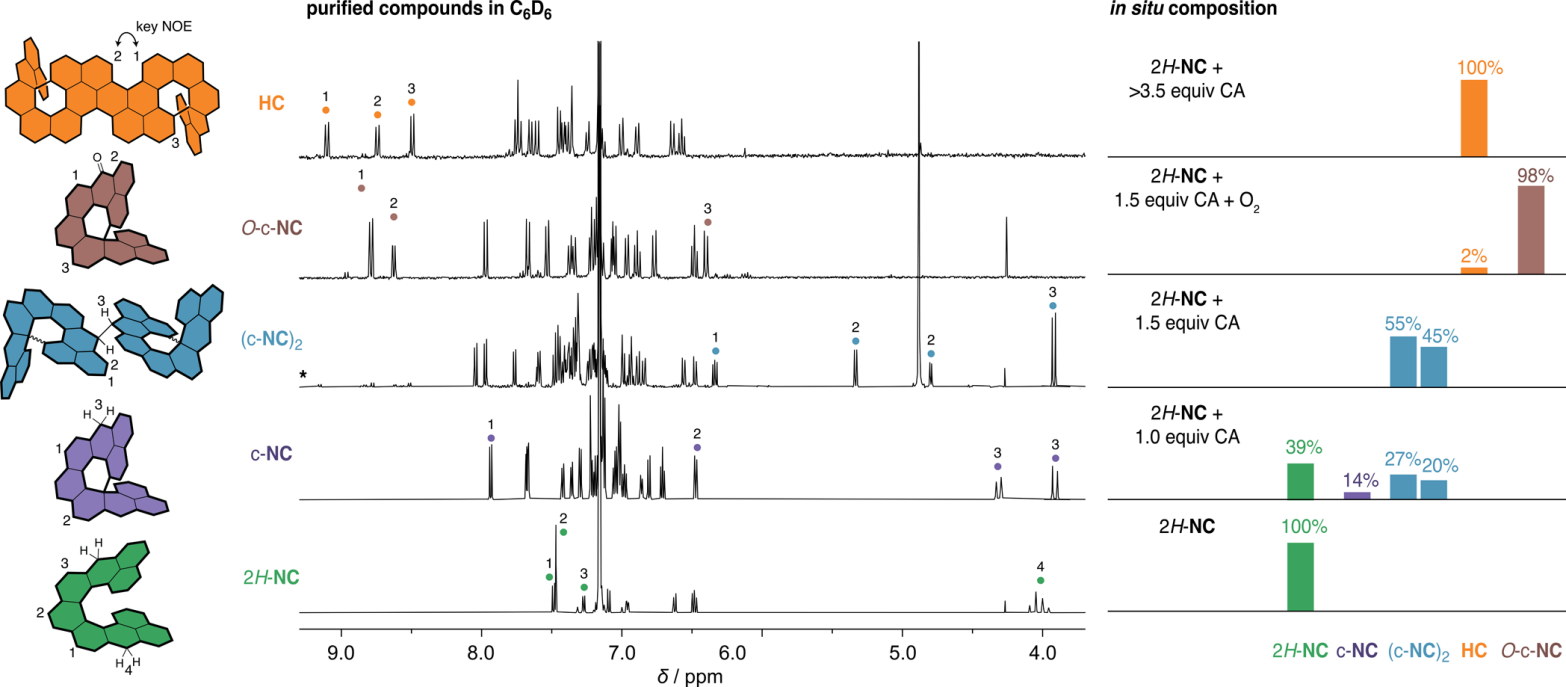
^1^H NMR (600 MHz, 298 K, C_6_D_6_) spectra of isolated, purified, and characterized compounds from the “unchained” reaction cascade with a partial assignment of the ^1^H resonances. The amount of oxidant and the corresponding in situ composition are illustrated on the right. * Not purified. (c-**NC**)_2_ was obtained as a mixture of two diastereoisomers: sym-(*P*,*S*,*R*,*M*)-(c-**NC**)_2_ (∼55%) and sym-(P*,R*,R*,P*)-(c-**NC**)_2_ (∼45%). CA = *p*-chloranil.

**Figure 2:**
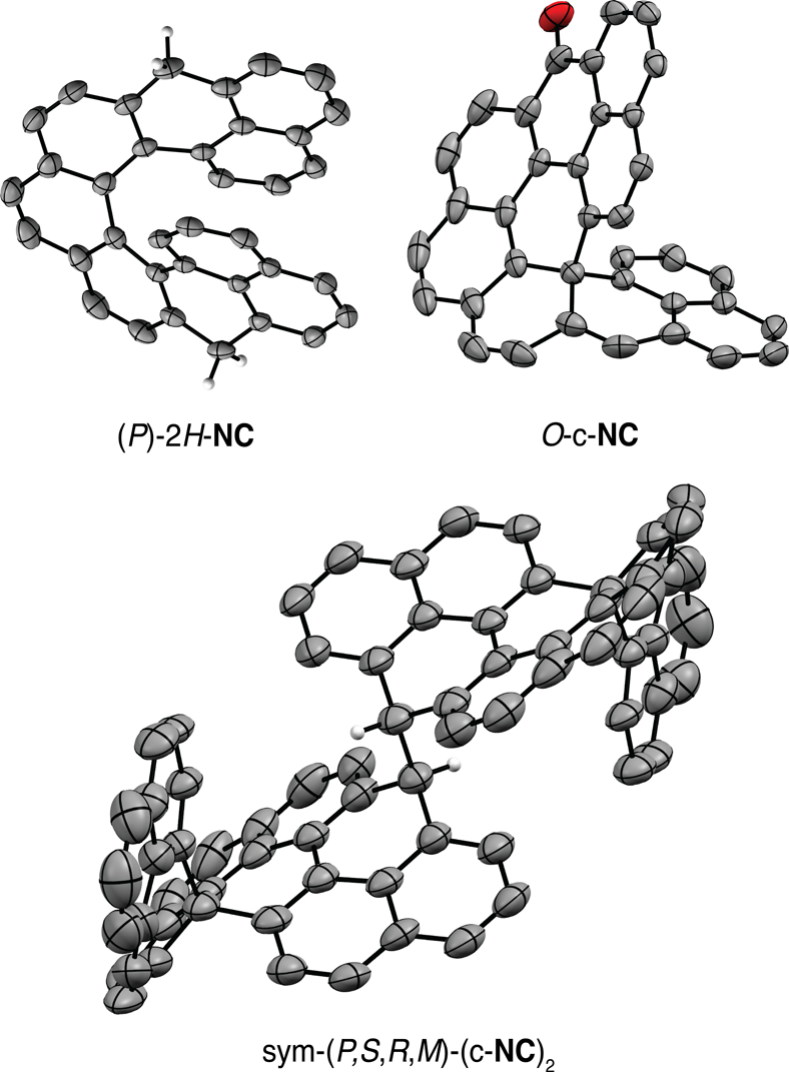
Perspective views of the solid-state structures of compounds involved in the cascade from the SC-XRD analysis. The thermal ellipsoids are shown at the 50% probability level. Color code: C/gray, O/red, H/white. Most of hydrogen atoms are omitted for clarity.

Finally, reduction with sodium borohydride and elimination of water with *para*-toluenesulfonic acid yielded the dihydro precursor 2*H*-**NC** in 85% yield over the two steps. As observed before for analogous systems,^49^ isomerization occurred during the dehydration step, where benzylic CH_2_-groups migrated to give the most stable dihydro isomer featuring one phenanthrene and two naphthalene subunits. Enantiomerically enriched samples of (*P*)- and (*M*)-2*H*-**NC** were obtained from **6** resolved into enantiomers using high-performance liquid chromatography (HPLC) equipped with a chiral stationary phase (Figures S7–S9). The structure of 2*H*-**NC** was unambiguously confirmed by 1D and 2D NMR spectroscopy, with full assignment of proton and carbon resonances (Figure 1 and Supporting Information), high-resolution mass spectrometry (HRMS), and single-crystal X-ray diffraction (SC-XRD) analysis of (*P*)-2*H*-**NC** (Figure 2); the single crystals were obtained by slow evaporation of solvents from a solution of (*P*)-2*H*-**NC** in CH_2_Cl_2_ and hexanes.

**Scheme 3:**
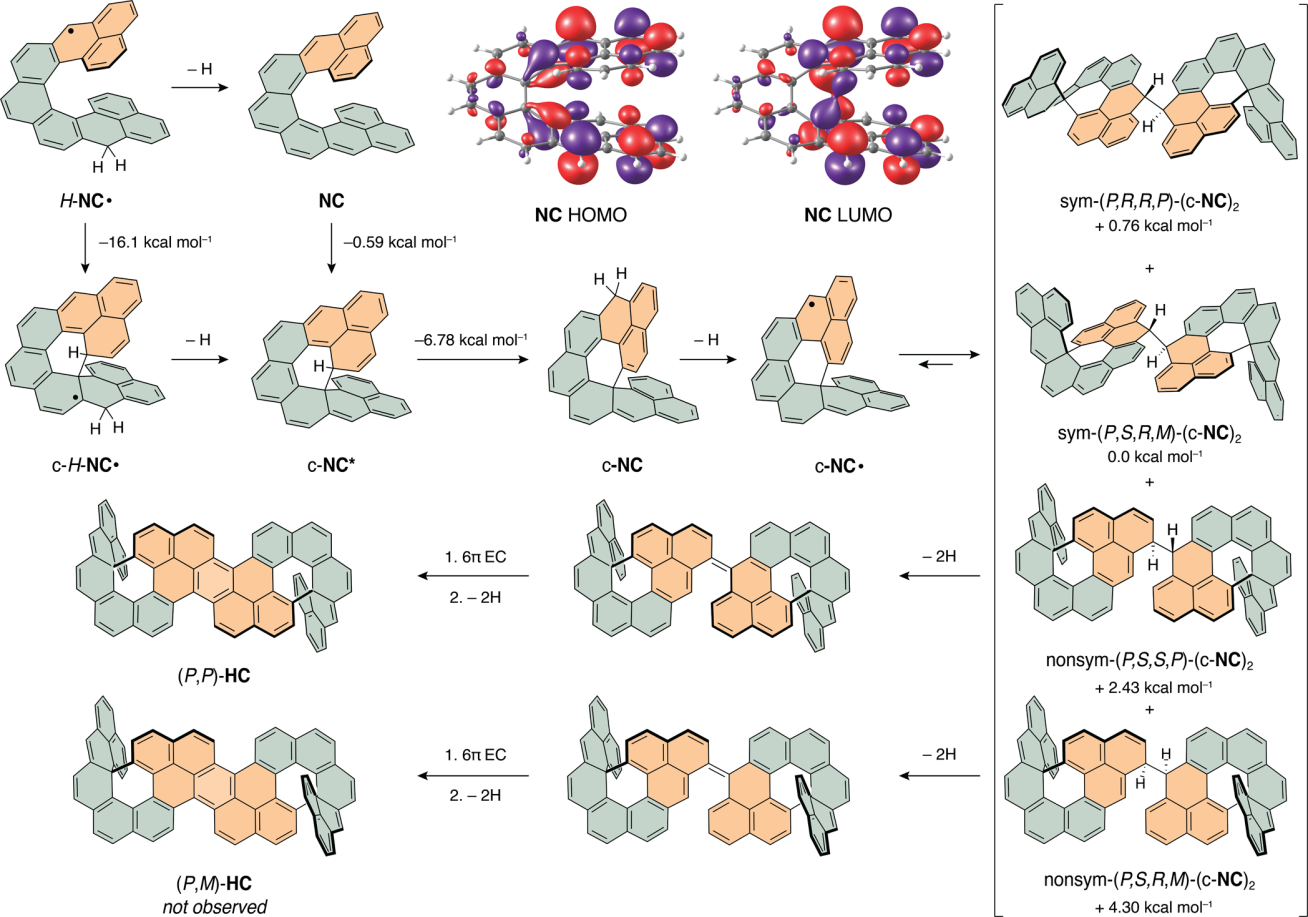
Proposed Mechanism for the Transformation of *H*-**NC**^**•**^ to **HC** Calculated relative energies (density functional theory (DFT)/ωB97XD/Def2SVP) are shown for isomeric structures. Note: Absolute configurations of the closed helicene units are assigned using helicity descriptors *P* and *M* instead of descriptors *R* and *S* for stereogenic sp^3^-centers, which makes it easier to relate these structures to their precursors featuring open helicene units.

With the aim to obtain **NC**, final oxidation under oxygen-free conditions with *p*-chloranil (CA) was performed. Unexpectedly, a variety of compounds formed and their relative ratio was dependent on the reaction time and the amount of oxidant used. Initially, c-**NC**, (c-**NC**)_2_, and *O*-c-**NC** (Scheme 2) were identified, but further experiments revealed additional species, including **HC** (Scheme 3).

### Oxidation Studies

Discovering the complex reactivity of the system, a systematic study of the oxidation process was carried out by varying the amount of oxidant CA. In this set of experiments, either 0.5, 1.0, 1.5, 2.0, 2.5, 3.0, or 3.5 equivalents of oxidant were added to a solution of 2*H*-**NC** in benzene-*d*_6_ (*c* ∼ 7 mM) by using a 50 mM solution of CA in benzene-*d*_6_, all solutions being argon-saturated. The reaction progress was monitored in time by ^1^H NMR spectroscopy, which revealed the composition of the reaction mixture at different stages. The individual oxidation experiments were compared, when significant changes were no longer occurring over time (Figure 1).

Upon the addition of 0.5 and 1.0 equiv of CA, two major species were observed in addition to the starting material 2*H*-**NC**. These species were identified as c-**NC**, a product of the apparent EC of **NC** followed by a hydrogen shift, and (c-**NC**)_2_, a σ-dimer of oxidized c-**NC** (Scheme 2). With 1.5 equiv of CA, only (c-**NC**)_2_ was present. All experiments exceeding 1.5 equiv of oxidant lead to an unexpected outcome, formation of a new compound identified as **HC**. Recalling the findings of Kubo^42^ and those from our laboratories,^26,39^ this result suggests further oxidation of the σ-dimer (c-**NC**)_2_, followed by steps analogous to the **PLY**–**PP** cascade. In the conversion of 2*H*-**NC** to **HC**, overall four new σ-bonds and one new π-bond are formed, which is accompanied by a loss of ten hydrogen atoms (Scheme 1). Because CA is a two-electron oxidant, 3.5 equiv of CA is required to achieve full conversion. In some experiments, a compound identified as *O*-c-**NC** (Scheme 2) was observed in varying amounts, even in experiments that were conducted under identical conditions. It was therefore rationalized that this product is formed due to the trace amounts of oxygen present in the samples, which vary from sample to sample. Exposure of (c-**NC**)_2_ to air resulted in a clean transformation to *O*-c-**NC**, ultimately proving this hypothesis.

Additionally, the oxidation of 2*H*-**NC** was performed with 2,3-dichloro-5,6-dicyano-1,4-benzoquinone (DDQ) as an oxidant. Surprisingly, upon the addition of an excess of DDQ (>3.5 equiv), the final product **HC** was not observed and the cascade proceeded only up to the (c-**NC**)_2_ intermediate. This result suggests that in benzene, DDQ is a weaker oxidant than CA, presumably due to the formation of a charge-transfer complex with the solvent. In the oxidations mediated by DDQ, a mixture of additional species was observed and upon exposure to air, a new diketo side product 2*O*-**NC** (Figure S118) was isolated in addition to *O*-c-**NC**. 2*O*-**NC** is an oxidation product of species that did not undergo a ring closure, most likely monoradical *H*-**NC**^**•**^ (Scheme 3), which is in equilibrium with its σ-dimers analogous to (c-**NC**)_2_ (see section Proposed Mechanism below for further details).

Electrochemical cyclic and differential pulse voltammetry measurements of 2*H*-**NC** in CH_2_Cl_2_ with [Bu_4_N][PF_6_] as the supporting electrolyte revealed two irreversible oxidation waves (0.60 and 0.90 V vs Fc/Fc^+^) and with sweep rates >100 mV, two irreversible reduction bands (Figure S15). This observation suggests reactive and short-lived intermediates, which is in accordance with the complex nature of the cascade process.

### Structure Identification

To validate the structures of the observed intermediates c-**NC** and (c-**NC**)_2_, the side product *O*-c-**NC**, and the final product **HC**, their proton and carbon resonances were all fully assigned by means of 2D NMR techniques, namely, COSY, TOCSY, NOESY/ROESY, HMQC/HSQC, and HMBC. For these measurements, c-**NC**, *O*-c-**NC**, and **HC** were isolated from the crude mixtures, when they formed in the highest quantities, depending on the amount of oxidant used (Figure 1). Compound (c-**NC**)_2_ was studied as formed when 1.5 equiv of CA was used. An overview of the compounds and partial assignment of their proton resonances is shown in Figure 1. Full assignment of proton and carbon resonances is available in the Supporting Information. The structures of (c-**NC**)_2_ and *O*-c-**NC** were additionally confirmed by SC-XRD (Figure 2).

The assignment for c-**NC** and *O*-c-**NC** was straightforward. It was used to identify (c-**NC**)_2_ and **HC** because of the similarity of the structures. In general, most of the characteristic carbon shifts were not influenced significantly by the structural changes. The most characteristic shifts were those of the newly formed quaternary sp^3^-carbon atom (50.1 ppm for c-**NC**) and three of the four neighboring sp^2^-carbon atoms that were quaternary already prior to the closure (134.3, 136.4, and 143.6 ppm for c-**NC**). When comparing the ^1^H NMR spectrum of (c-**NC**)_2_ to that of c-**NC**, the main difference was that there was only one sp^3^-proton instead of two, suggesting a substituent attached to this carbon atom. HMBC showed a strong correlation between the sp^3^-proton and the carbon atom attached directly to the sp^3^-carbon atom, which indicated that a σ-dimer had formed. The formation of the σ-dimer was further supported by the fact that two species with identical structure but different chemical shifts (e.g., 3.93 and 3.90 ppm for the sp^3^-proton) were present in a ratio of 1:1.2. This observation indicates formation of two diastereomeric structures, which had to be symmetric because only one set of signals was observed for two monomeric units in each case.

To identify the diastereomers, the oxidation experiment with CA was repeated using enantiomerically enriched (*P*)- and (*M*)-2*H*-**NC** (*P*, >99% ee; *M*, 96% ee). In both cases, an identical spectrum was obtained (Figure S3), which means that the diastereomer with a singlet at 3.90 ppm had to be sym-(*P*,*R*,*R*,*P*)-(c-**NC**)_2_^50^ in the case of (*P*)-2*H*-**NC** and sym-(*M*,*S*,*S*,*M*)-(c-**NC**)_2_ in the case of (*M*)-2*H*-**NC**, and the other diastereomer had to be achiral sym-(*P*,*S*,*R*,*M*)-(c-**NC**)_2_. The structure of the achiral diastereomer was unambiguously confirmed by SC-XRD (Figure 2).

For the complete assignment of **HC** (Figure S26), the NMR spectra were measured in tetrachloroethane-*d*_2_ due to better solubility compared to benzene and no overlap with the solvent residual peak (Figures S109–S117). The key NOE correlation of the proton 1 and proton 2 unambiguously confirms the structure of **HC** and is depicted with an arrow in Figure 1. This result was surprising because this product cannot be formed from the observed symmetric σ-dimers sym-(c-**NC**)_2_.

### DFT Calculations

To shed light on the mechanism of this reaction cascade, density functional theory (DFT) calculations were performed at the ωB97XD/Def2SVP level of theory (Scheme 3). The first compound to be detected, isolated, and characterized in the reaction cascade is c**-NC**. There are two reasonable pathways from 2*H*-**NC** to c**-NC**, both starting with oxidation of 2*H*-**NC** to *H*-**NC**^**•**^. From *H*-**NC**^**•**^, either a second oxidation takes place to give **NC**, which then undergoes EC to give c-NC*. Alternatively, *H*-**NC**^**•**^ undergoes a radical ring closure to c-*H*-**NC**^**•**^ and then oxidation to c-NC*. By comparing optimized geometries of **NC**/c-NC* and *H*-**NC**^**•**^/c-*H*-**NC**^•^, both processes are thermodynamically favored (by 0.59 and 16.1 kcal mol^–1^, respectively). In both cases, c-NC* isomerizes to c-**NC**, which is 6.78 kcal mol^–1^ more stable than c-NC*.

c-**NC** then oxidizes to c-**NC**^**•**^ (or c-NC* directly oxidizes to c-**NC**^**•**^), which is in equilibrium with several possible σ-dimers.^51^ Analogous σ-dimerizations are well known in the literature.^49,52,53^ On comparing sym-(P*,R*,R*,P*)-(c-**NC**)_2_ and sym-(*P*,*R*,*S*,*M*)-(c-**NC**)_2_, the difference in energy is only 0.76 kcal mol^–1^ in favor of the latter, which corroborates the observations by NMR spectroscopy. Based on the structure of **HC**, it is clear that **HC** must be formed from a non-symmetric (c-**NC**)_2_, that is, a dimer formed by linking two monomeric c-**NC**^**•**^ units via different positions. The most stable non-symmetric dimers identified by DFT are nonsym-(P*,R*,R*,P*)-(c-**NC**)_2_ and nonsym-(P*,R*,S*,P*)-(c-**NC**)_2_. They are equal in energy and both give (P*,P*)-**HC** upon completion of the cascade (Scheme 3 and Tables S3 and S4). These dimers are higher in energy by 1.67 and 2.43 kcal mol^–1^ when compared to sym-(P*,R*,R*,P*)-(c-**NC**)_2_ and sym-(*P*,*R*,*S*,*M*)-(c-**NC**)_2_, respectively, and are therefore present in quantities not detected in the acquired ^1^H NMR spectrum. The most stable non-symmetric dimer that would lead to achiral (*P*,*M*)-**HC**, which was not observed, is nonsym-(*P*,*R*,*S*,*M*)-(c-**NC**)_2_ (Scheme 3). This dimer is higher in energy by ∼2 kcal mol^–1^ when compared to the most stable non-symmetric dimers.

### Proposed Mechanism

The proposed mechanism for the 2*H*-**NC**–**HC** cascade is outlined in Scheme 3 and involves the formation of the first observable intermediate c-**NC**, its oxidation to c-**NC**^**•**^, which undergoes the **PLY**–**PP** cascade to form **HC**, the final product.

**Figure 3:**
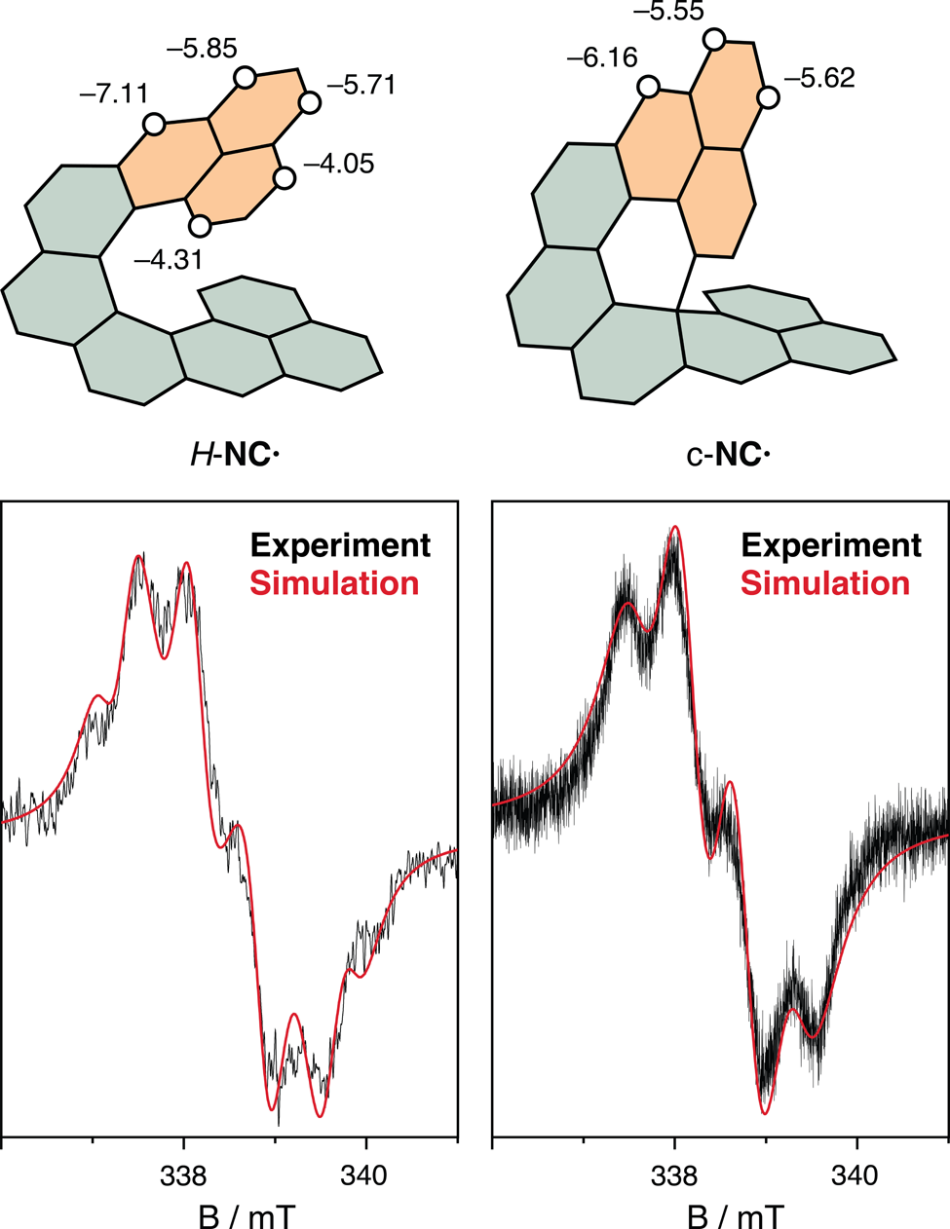
Comparison of the experimental and simulated EPR spectra of *H*-**NC**^**•**^ (left) and c-**NC**^**•**^ (right). Experimental parameters: scan time 1800 s, modulation amplitude 0.2 mT, microwave power 10 mW. Simulation parameters: main hyperfine coupling constants from DFT (Figures S124 and S125) that were used for the fitting were scaled by 75% (*H*-**NC**^**•**^; line width = 3.5 G) and 80% (c-**NC**^**•**^; line width = 5.0 G).

Initially, we thought that c-**NC** is formed via a 6π EC of **NC** and subsequent hydrogen shift. The reason for not observing **NC** as an intermediate could be a very low activation energy of EC due to **NC**’s small HOMO–LUMO gap (1.17 eV), which is significantly smaller than that of cethrene (1.69; 1.65 eV, onset of absorption^26^). Since the low-lying doubly excited state contributes to the lowering of the activation energy of this formally forbidden thermal process,^41^ EC of **NC** is expected to be much faster than that of cethrene (2× vs 1× antibonding interaction in the HOMO of **NC** vs cethrene; Scheme 3). When formed, **NC** would therefore immediately cyclize to c-NC* and would not be detected. DFT calculations offer another possibility, namely, radical closure of mono-oxidized 2*H*-**NC**, *H*-**NC**^**•**^, and subsequent oxidation to c-NC*. Based on calculations, both routes are plausible and none of them can be excluded. The intermediate *H*-**NC**^**•**^ could be detected when DDQ was used as an oxidant instead of CA. Upon the addition of 0.5 equiv of DDQ, no (c-**NC**)_2_ σ-dimers were formed but EPR spectroscopy revealed the presence of *H*-**NC**^**•**^ (Figure 3, left). The proton hyperfine coupling constants used for the simulation of the experimental EPR signal proportionally match those calculated for *H*-**NC**^**•**^ (Figure S125). The NMR spectrum revealed new species, which we tentatively assigned to the σ-dimers of *H*-**NC**^**•**^. The fact that DDQ yields *H*-**NC**^**•**^ and that 2*O*-**NC** can be isolated when the mixture is in contact with air suggests that both the oxidation to **NC** and the radical ring closure to c-*H*-**NC**^**•**^ must be slower than the mono-oxidation of 2*H*-**NC** under these conditions. For isomerization of c-NC* to c-**NC**, a twofold [1,3] or a [1,5] suprafacial sigmatropic hydrogen shift can be imagined as a mechanism for this hydrogen migration, in addition to acid-catalyzed isomerization (protons provided by reduced CA).

Oxidation of 2*H*-**NC** with 0.5 equiv of CA (a two-electron oxidant) results in a mixture of 2*H*-**NC**, c-**NC**, and (c-**NC**)_2_. This result indicates that the oxidation of 2*H*-**NC** proceeds at a comparable rate as the oxidation of c-**NC**. Upon addition of 1.0 equiv of CA, the amount of c-**NC** decreased and more (c-**NC**)_2_ formed, while 2*H*-**NC** was still present (Figure 1). With 1.5 equiv of CA, a point is reached where only (c-**NC**)_2_ is observed, which implies that the oxidation of (c-**NC**)_2_ is the rate-determining step of the cascade.

The two observed (c-**NC**)_2_ species are in equilibrium with the monomeric radical species, c-**NC**^**•**^. The presence of c-**NC**^**•**^ was validated by EPR spectroscopy of a solution of (c-**NC**)_2_, where the radical species could be detected even after 2 weeks of storing (c-**NC**)_2_ in the glovebox (Figure 3, right). A higher signal intensity could not be achieved as the equilibrium is largely shifted toward the dimer. The simulated EPR spectrum reproduces well the main features of the experimental one, and the proton hyperfine coupling constants used for the simulation proportionally match the calculated ones (Figures 3 and S124). The spin density map shows that the highest spin density is at the carbon atom, where the σ-bond is formed between the monomeric units of the observed most stable (c-**NC**)_2_ σ-dimers (see the Supporting Information). The neighboring carbon atom also shows a significant spin density, which makes the non-symmetric dimers plausible. Variable-temperature ^1^H NMR measurements of (c-**NC**)_2_ revealed that the signals of the more stable sym-(*P*,*S*,*R*,*M*)-(c-**NC**)_2_ decreased in intensity with increasing temperature and the spectrum was fully restored after cooling, confirming that c-**NC**^**•**^ and (c-**NC**)_2_ are in dynamic equilibrium.

An explanation as to why only the isolated (P*,P*)-**HC** isomer is formed is that the oxidation of the non-symmetric dimer, non-sym-(P*,R*, R*,P*)-(c-**NC**)_2_, which is higher in energy than the symmetric ones, has a smaller activation energy and proceeds faster than the oxidation of the symmetric dimers, which are in equilibrium with the non-symmetric one. This isomer is obtained even when the cascade is carried out from racemic 2*H*-**NC**. This observation is in accordance with DFT calculations (vide supra). In between (c-**NC**)_2_ and (P*,P*)-**HC**, no further intermediates could be detected, indicating that EC and the second oxidation step are faster than the oxidation of (c-**NC**)_2_.

### Absorption and Emission

**Figure 4:**
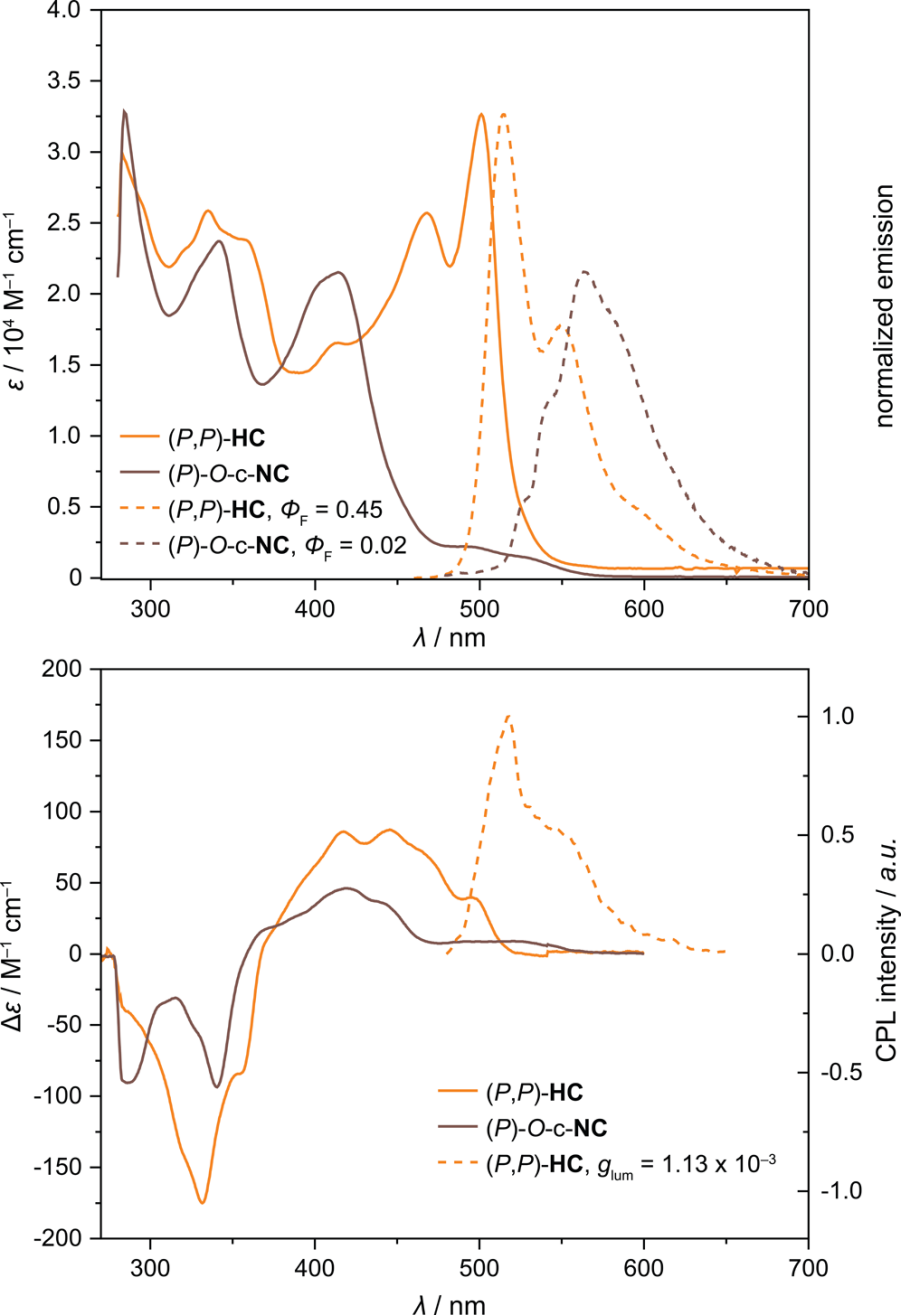
Absorption and emission (top) and circular dichroism (CD) and circularly polarized luminescence (CPL; bottom) spectra of enantioenriched (*P*,*P*)-**HC** and (*P*)-*O*-c-**NC** at 20 °C in toluene. Φ_F_ = quantum yield. *g*_lum_ = luminescence dissymmetry factor.

The absorption and emission spectra of **HC** were recorded in toluene at 20 °C (Figure 4). The profile of the UV–vis absorption spectrum of **HC** shows high similarity to those of functionalized peropyrene derivatives reported in the literature,^54,55^ suggesting that the absorption properties arise mainly from the **PP** unit of **HC**. For a comparison, *O*-c**-NC** absorbs strongly in the UV and weakly in the visible region. Similar to absorption, the profile of the emission of **HC** also resembles that of **PP**. The fluorescence quantum yield (Φ_F_) of **HC** in toluene is 0.45 (excitation at 470 nm, Figure 4) with a Stokes shift of 60 meV. The Φ_F_ is lower by a factor of ∼2 compared to **PP**,^56^ which can be attributed to the twisted structure^57−59^ of the **PP** unit induced by strain from the locked helicene units. For a comparison, *O*-c**-NC** shows a much larger Stokes shift of 840 meV, but a small Φ_F_ of 0.02, on account of quenching of the fluorescence by the carbonyl group.

### Circular Dichroism

The (*P*) and (*M*) enantiomers of **6** were separated by HPLC equipped with a chiral stationary phase (Figures S7–S9), and the absolute configuration was assigned with the aid of circular dichroism (CD) spectra (Figure S10), time-dependent (TD)-DFT calculations (Figure S11), and optical rotation ( (*P*) = +4095;  (*M*) = −4018, lit.^60^ ((*P*)-[7]helicene) ∼ +6200). In the CD spectra (Figure S10), the enantiomers displayed mirror-image Cotton effects. The (*P*) enantiomer (>99% ee) of **6** was reduced and dehydrated to obtain the (*P*) enantiomer of 2*H***-NC**. The reaction cascade with (*P*)-2*H***-NC** afforded the corresponding (*P*,*P*)**-HC** and (*P*)-*O*-c**-NC** that were isolated. CD spectra for both compounds are shown in Figure 4 and match those predicted by TD-DFT (Figures S12 and S13).

### Circularly Polarized Luminescence

Enantiomerically enriched (*P*,*P*)-**HC** showed good circularly polarized luminescence (CPL) response with an absorption dissymmetry factor *g*_abs_ = 1.13 × 10^–3^ at 502 nm (Figure S14). Interestingly, the luminescence dissymmetry factor *g*_lum_ has the same value of 1.13 × 10^–3^ at 520 nm (Figure 4). The *g*_lum_/*g*_abs_ value as a measure of excited state relaxation for helicenes and helicenoids is typically^61,62^ ∼0.6. The deviation from unity is accounted for the flexibility of helicenes and helicenoids.^63^ For (*P*,*P*)-**HC**, however, the *g*_lum_/*g*_abs_ value is precisely 1.0 suggesting high rigidity, as expected for this structure with both helicene units locked via sp^3^-centers. For a better quantification of CPL emitter efficiency and better comparison, CPL brightness (*B*_CPL_) was introduced as a measuring quantity.^64^*B*_CPL_ for (*P*,*P*)-**HC** is 8.3 M^–1^ cm^–1^, which is right at the median of values reported for [7]helicenes (including heteroatom containing helicenes).

## Conclusions

The reactivity of graphene-based π-radicals and π-radicaloids is to a large extent an unexplored territory. The research in this field is primarily focused on the properties that arise from the presence of unpaired electrons and reactivity of these systems is prevented. Consequently, reaction pathways and byproducts are typically not investigated in depth, when decomposition occurs, giving an impression that they are undefined. In this work, we demonstrated that reactivity of “unchained” diradicaloid molecules can be well-defined and has potential to become a useful synthetic tool to access complex carbon nanostructures via a series of multiple selective steps. The key element of the presented cascade is the formation of a quaternary sp^3^-center—an “sp^3^-defect”—during an EC or a radical ring-closing step, which locks the system from full fusion previously observed for cethrene (Scheme 1). Upon oxidation, the system is activated for the second cascade round, analogous to oxidative dimerization of phenalenyl to peropyrene. The overall result is a sequence of up to nine steps, where four new σ-bonds and one new π-bond are formed to give a conjugated contorted nanocarbon **HC**, containing two precisely installed sp^3^-defects. By modulating the amount of oxidant, various intermediates of this cascade could be identified or even isolated and characterized. The photophysical properties of **HC** mostly resemble those of peropyrene, including its fluorescence with a quantum yield of 0.45. Resolution of enantiomers achieved via preparative chiral-stationary-phase HPLC made it possible to perform the reaction cascade with enantiomerically enriched (*P*) starting material. The isolated (*P*,*P*)-**HC** showed circular dichroism and circularly polarized luminescence with a brightness value of 8.3 M^–1^ cm^–1^. Our results show that reactivity of graphene-based π-radical(oid)s is a promising synthetic tool toward carbon nanostructures.

## Experimental Section

### Synthesis and Characterization

The experimental procedures and characterization data for all new compounds described in this work are compiled in the Supporting Information. Two routes A and B (Schemes S1 and S2, respectively) were followed to obtain 2*H*-**NC**. Overall, the total number of steps is the same for both approaches, but the twofold cyclization to obtain helicene **15** (route B) is not ideal as a method due to the partial loss of one or both bromine atoms and tedious separation of the byproducts from the desired helicene **15**. The side chains were therefore installed prior to the twofold cyclization (route A, Schemes 2 and S1). Consequently, all carbon atoms of the **NC** scaffold are installed one step earlier and the linear synthetic sequence is shorter by converging the route. Route A is also more scalable because the photocyclization step, which needs to be performed at low concentrations (∼10^–3^ M), is closer to the end of the synthetic pathway.

All chemicals and solvents were purchased from commercial sources and were used without further purification unless stated otherwise. The reactions and experiments that are sensitive to dioxygen were performed using Schlenk techniques and nitrogen- or argon-saturated solvents.

### EPR Spectroscopy

The EPR spectra were recorded in nitrogen-saturated benzene on an X-band bench-top EPR spectrometer (9.48 GHz) using the following instrumental parameters: magnetic field 336–341 mT, scan time 1800 s, modulation amplitude 0.2 mT, microwave power 10 mW.

### UV–vis, CD, and Optical Rotation Spectroscopy

The specific optical rotation  (in ° mL dm^–1^ g^–1^) was measured at the indicated temperature *T* (in °C) and the concentration *c* (in g/100 mL). Infrared (IR) spectra were recorded on a Fourier transform infrared (FT-IR) attenuated total reflection (ATR) spectrophotometer, where samples were applied as neat samples or as films. The UV–vis measurements were performed on an Agilent 8453 spectrophotometer. CD spectra were recorded on a JASCO J-1000 Series CD spectropolarimeter.

### Fluorescence and CPL Spectroscopy

Fluorescence measurements were carried out using a calibrated Edinburgh Instruments FS5 spectrofluorometer equipped with an SC-25 Temperature Controlled Holder TE-Cooled-Standard cell for emission spectra and an SC-30 Integrating Sphere cell for obtaining quantum yields. All samples were measured in quartz-glass fluorescence cuvettes with a 1 cm path length using spectroscopy-grade solvents. CPL spectra were recorded with a customized JASCO CPL-300/J-1500 hybrid spectrometer.

### NMR Spectroscopy and HRMS

The NMR experiments were performed on NMR spectrometers operating at 400, 500, or 600 MHz proton frequencies. Standard pulse sequences were used. Chemical shifts (δ) are reported in parts per million (ppm) relative to the solvent residual peak (^1^H and ^13^C NMR, respectively): CDCl_3_ (δ = 7.26 and 77.16 ppm^65^), CD_2_Cl_2_ (δ = 5.32 and 53.84 ppm^65^), and C_2_D_2_Cl_4_ (δ = 6.00 and 73.78 ppm^66^). High-resolution mass spectra were measured as HR-EI-MS, HR-ESI-MS, or HR-APCI-MS.

### Single-Crystal X-ray Diffraction

All information about experimental setup, conditions, and software is compiled in the Supporting Information (section S7). Suitable crystals of (*P*)-**6** for X-ray diffraction (XRD) were grown by slow evaporation from hexanes/CH_2_Cl_2_ solvent mixture. Suitable crystals of (*P*)-2*H*-**NC** for XRD were grown by slow evaporation from CHCl_3_. Suitable crystals of (±)-*O*-c-**NC** for XRD were grown by slow evaporation from heptane/toluene solvent mixture. Suitable crystals of (*P,S,R,M*)-(**NC**)_2_ for XRD were grown by slow evaporation from oxygen-free benzene-*d*_6_ in an NMR tube.

### DFT Calculations

DFT calculations were performed using Gaussian 16 suite.^67^ The multireference CASSCF calculations were performed using ORCA 4.2.1.^68^ Geometries were optimized using ωB97XD functional and Def2SVP basis set in the gas phase. The frequency analysis was performed to verify the stationary-state geometry, where no imaginary frequency was found. TD-DFT calculations were performed on the ωB97XD/Def2SVP-optimized geometries at the ωB97XD/Def2SVP level of theory. The effect of the solvent was accounted for using PCM (with toluene as the solvent). SpecDis and Chemcraft software were used to analyze the TD-DFT-calculated spectra and generate graphical images of the frontier molecular orbitals, respectively.

For **NC**, the singlet geometries were optimized with spin-restricted and spin-unrestricted broken-symmetry wavefunctions, whereas the triplet geometry was optimized with spin-unrestricted wavefunctions. For the spin-restricted wavefunction, the RHF → UHF instability was found, which supports the open-shell singlet ground state for **NC**. The HOMO–LUMO energy gaps of cethrene (1.69 eV) and **NC** (1.17 eV) were determined by geometry optimization of a broken-symmetry singlet state at the B3LYP-GD3BJ/Def2TZVPP level of theory. The B3LYP functional was used because the obtained HOMO–LUMO energy gap of cethrene was close to the experimental optical energy gap (1.65 eV^26^). The diradical character (0.9) was estimated using multireference CASSCF(2,2) calculation. The adiabatic singlet–triplet energy gap (0.17 kcal mol^–1^) was calculated using the CASSCF(2,2)/NEVPT2/Def2TZVPP approach on DFT-optimized singlet and triplet geometries.

## Supporting Information Available

Synthetic procedures and characterization data for all new compounds; UV–vis, HPLC, CD, EPR, XRD, and DFT calculation details; assignment of ^1^H and ^13^C NMR resonances; copies of NMR and HRMS spectra; and Cartesian coordinates (PDF)
Crystallographic data for CCDC 2157012 ((*P*)-**6**) (CIF)
Crystallographic data for 2157015 (2*H*-**NC**) (CIF)
Crystallographic data for 2157016 ((c-**NC**)_2_) (CIF)
Crystallographic data for 2157017 (O-c-**NC**) (CIF)

## Supplementary Material

au2c00190_si_001.pdf

au2c00190_si_002.cif

au2c00190_si_003.cif

au2c00190_si_004.cif

au2c00190_si_005.cif

## Accession Codes

CCDC 2157012 ((*P*)-**6**), 2157015 (2*H*-**NC**), 2157016 ((c-**NC**)_2_), and 2157017 (*O*-c-**NC**) contain the supplementary crystallographic data for this paper. These data can be obtained free of charge via www.ccdc.cam.ac.uk/data_request/cif, or by emailing data_request@ccdc.cam.ac.uk, or by contacting The Cambridge Crystallographic Data Centre, 12 Union Road, Cambridge CB2 1EZ, UK; fax: +44 1223 336033.
