# Supplementary material for: Nonacethrene Unchained: A Cascade to Chiral Contorted Conjugated Hydrocarbon with Two sp3-Defects
Source: JACS Au. 2022 Jul 9;2(7):1616–26. doi: 10.1021/jacsau.2c00190 (PMC9326821; doi:10.1021/jacsau.2c00190)
Supplement: Supplementary file 1 — au2c00190_si_001.pdf [file au2c00190_si_001.pdf]

Supporting Information for

Nonacethrene Unchained: A Cascade to Chiral Contorted Conjugated Hydrocarbon with Two sp^3-Defects

Daniel Čavlović,[†] Daniel Häussinger,[‡] Olivier Blacque,[†] Prince Ravat,[*,‡,§] and Michal Juríček[*,†,‡]

[†]Department of Chemistry, University of Zurich, Winterthurerstrasse 190, 8057 Zurich, Switzerland
[‡]Department of Chemistry, University of Basel, St. Johanns-Ring 19, 4056 Basel, Switzerland
[§]Institute of Organic Chemistry, University of Würzburg, Am Hubland, 97074 Würzburg, Germany

Table of contents

**S1.**	**Supporting figures**	**S2**
**S2.**	**Synthesis**	**S4**
**S3.**	**UV–Vis spectroscopy**	**S19**
**S4.**	**HPLC separation of enantiomers**	**S21**
**S5.**	**CD spectroscopy and CPL**	**S23**
**S6.**	**CV and DPV**	**S26**
**S7.**	**X-ray crystallography**	**S27**
**S8.**	**Assignment of ^{1}H and ^{13}C NMR resonances**	**S37**
**S9.**	**Copies of NMR spectra**	**S41**
**S10.**	**DFT calculations**	**S89**
**S11.**	**Cartesian coordinates**	**S97**
**S12**	**Copies of HR-MS data**	**S127**
**S13.**	**References**	**S142**

HAPPY BIRTHDAY SIR FRASER STODDART!

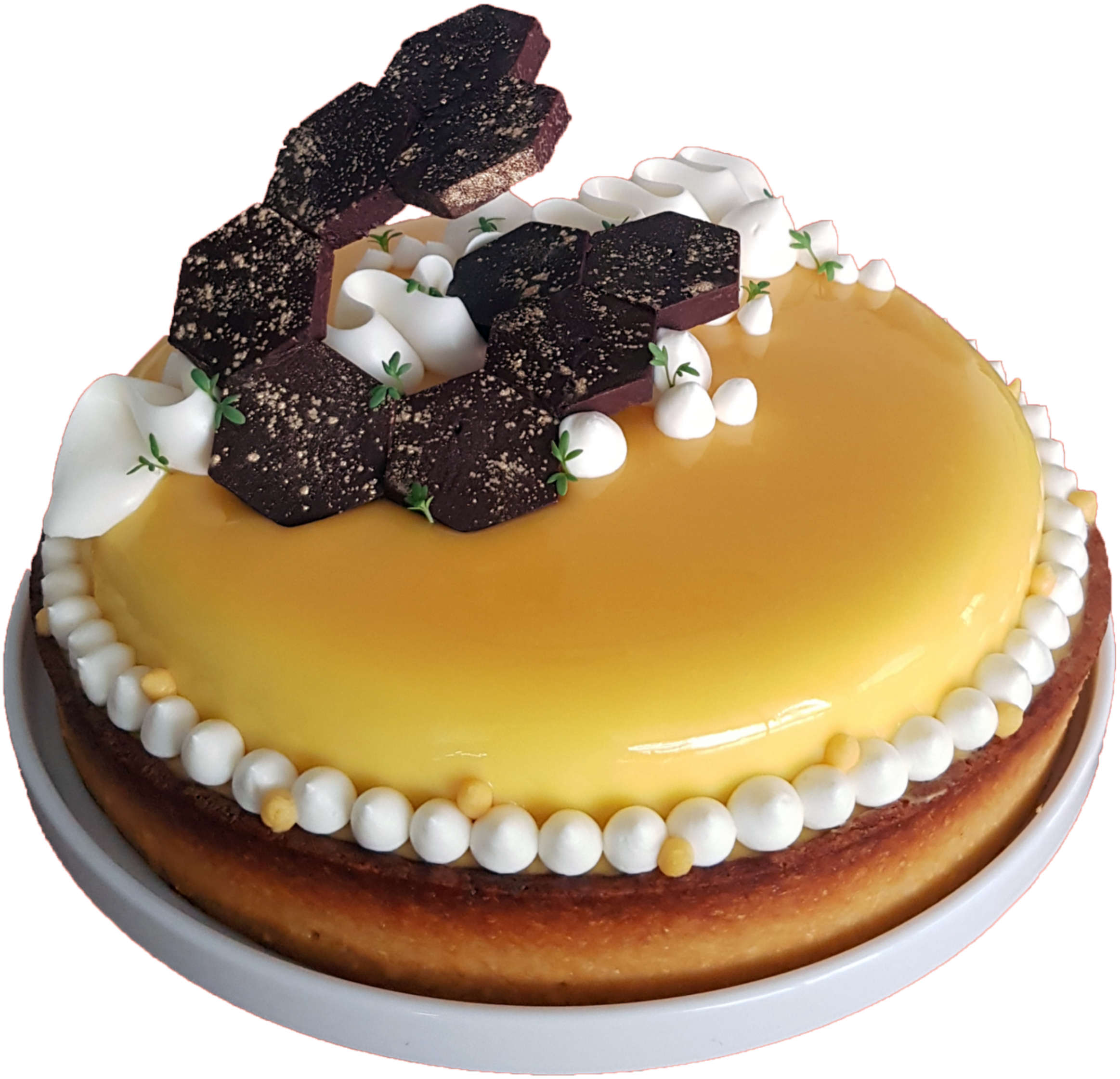

Figure S1. Birthday cake decorated with nonacethrene dedicated to Prof. Sir Fraser Stoddart on the occasion of his 80th birthday. The credit for the preparation and decoration of this beautiful and delicious cake goes to Dr. Lenka Štacková.

S1. Supporting figures

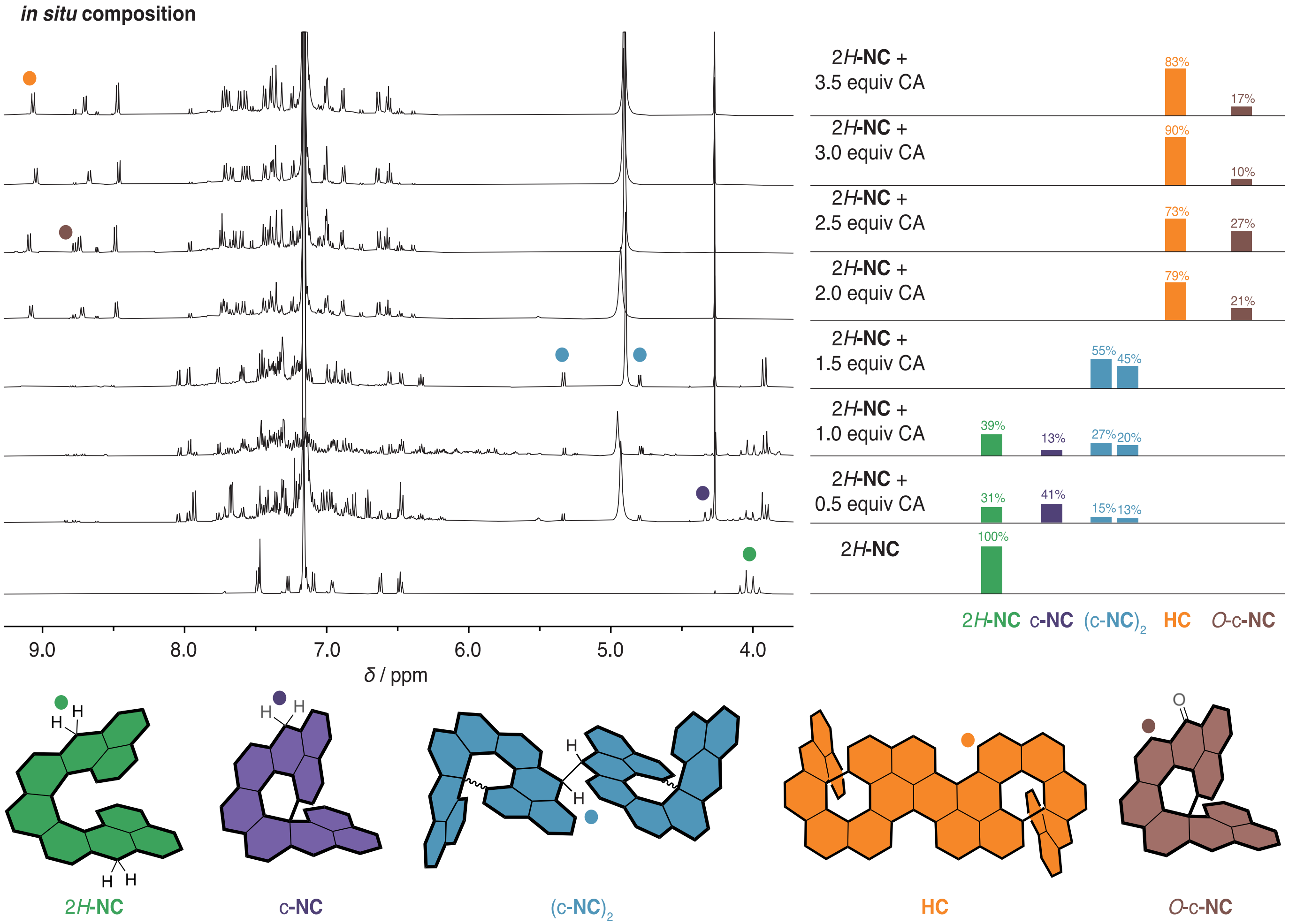

Figure S2. Comparison of ^{1}H NMR (400 MHz, 298K, C_6D_6) spectra of 2*H*-**NC** before and after the addition of different amounts of *p*-chloranil (CA). The most diagnostic peaks are highlighted with a dot in the corresponding color. Relative compositions are depicted with colored bars on the right and the height of the bars is determined by integration of the diagnostic peaks. (c-**NC**)$_2$ resulted in a mixture of two diastereoisomers: sym-(*P**,*R**,*R**,*P**)-(c-**NC**)$_2$ and sym-(*P**,*S**,*R**,*M**)-(c-**NC**)$_2$.

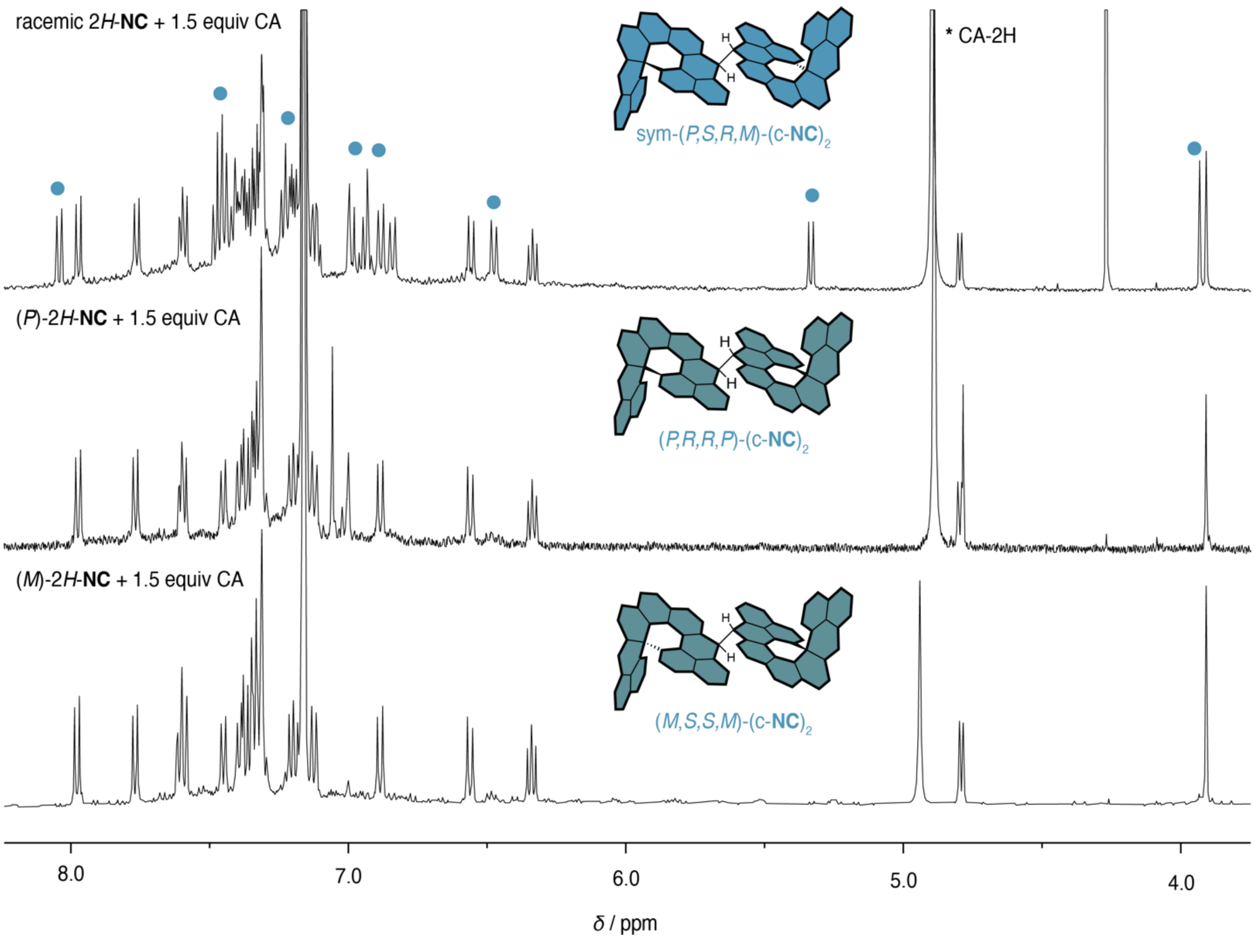

Figure S3. Comparison of ^{1}H NMR (400 MHz, 298K, C_6D_6) spectra of racemic 2*H*-**NC** (top), (*P*)-2*H*-**NC** (middle), and (*M*)-2*H*-**NC** (bottom) after the addition of 1.5 equivalents of *p*-chloranil (CA). CA-2H = *p*-tetrachlorohydroquinone.

S2. Synthesis

Scheme S1. Overview of Synthetic Route **A**.

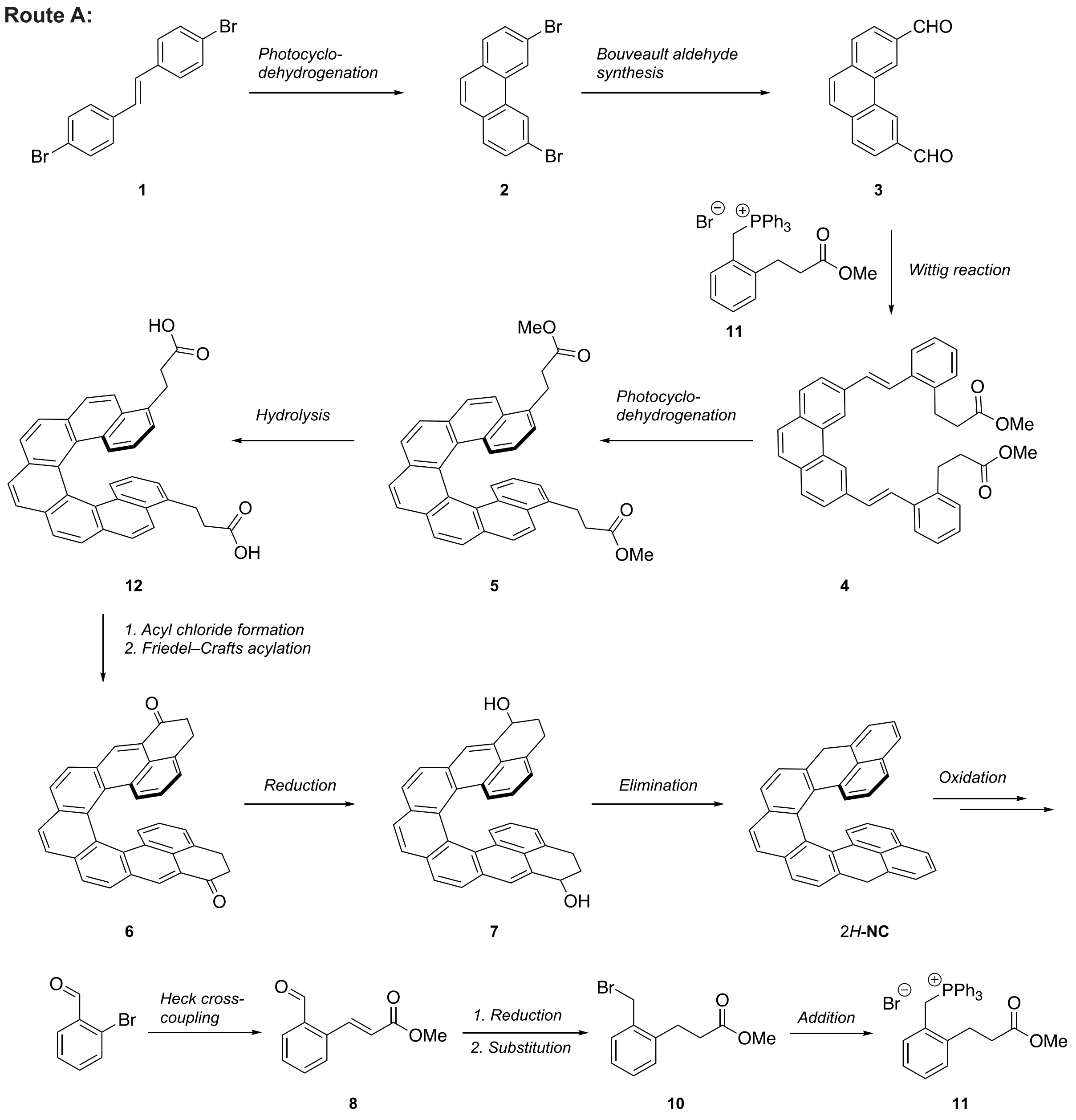

Scheme S2. Overview of Synthetic Route **B**.

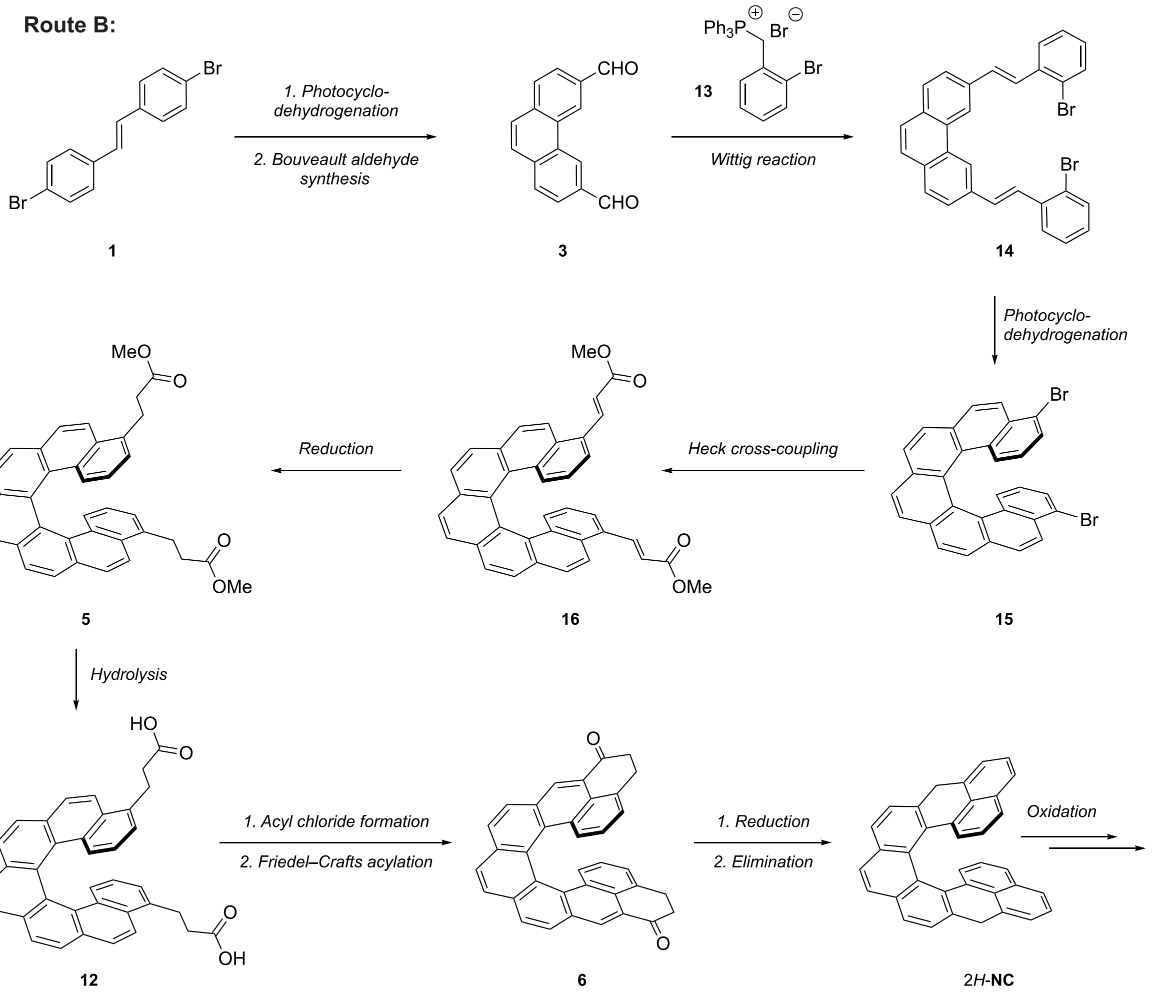

Route A:

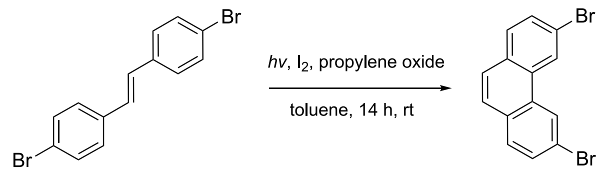

1 **2, 78%**

3,6-Dibromophenanthrene (2). The photocyclodehydrogenation reaction was performed using an immersion-well photoreactor system equipped with quartz cooling and immersion tubes and a 150 W medium-pressure mercury lamp. An argon-saturated solution of (*E*)-4,4'-dibromostilbene (**1**, 500 mg, 1.48 mmol, 1.0 equiv), iodine (830 mg, 3.27 mmol, 2.2 equiv), and propylene oxide (20 mL) in toluene (680 mL) was irradiated for 14 h. The resulting solution was washed with aqueous $NaHSO_3$ (10 wt%) and water. The organic layers were dried with $MgSO_4$ and filtered before the solvent was evaporated. The residue was passed through a short pad of silica gel using CH_2Cl_2 as an eluent. After evaporation of the solvents, the residue was recrystallized from CH_2Cl_2 to afford the desired product (390 mg, 78%) as a colorless crystalline solid (mp 192.2–192.8 °C). ^1H NMR (400 MHz, CD_2Cl_2, ppm): δ 8.75 (d, J = 1.9 Hz, 2H), 7.80 (d, J = 8.5 Hz, 2H), 7.76–7.70 (m, 4H). ^{13}C NMR (101 MHz, CD_2Cl_2, ppm): δ 131.2, 131.0, 130.8, 130.6, 127.2, 126.0, 121.5. IR: 1587, 1407, 847, 836, 517 cm^{-1}. HRMS (EI) m/z: $[M]^+$ Calcd for $C_{14}H_8Br_2$ 333.89873; Found 333.89919.

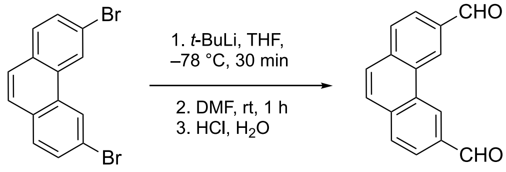

2 **3, >99%**

Phenanthrene-3,6-dicarbaldehyde (3). To a solution of 3,6-dibromophenanthrene (**2**, 339 mg, 1.01 mmol, 1.0 equiv) in THF (15 mL) at –78 °C, *t*-BuLi (1.6 M in pentane, 2.6 mL, 4.2 mmol, 4.1 equiv) was added dropwise and the mixture was stirred for 30 minutes before anhydrous DMF (0.8 mL, 10 mmol, 10 equiv) was added. Then, the cooling was removed and the mixture was stirred for additional 1 h. The resulting mixture was poured into aqueous HCl (2 M) and extracted with CH_2Cl_2. The combined organic layers were dried over anhydrous $MgSO_4$ and filtered to afford the desired product (236 mg, >99%) as a colorless solid (mp 222.0–223.9 °C). ^1H NMR (400 MHz, CD_2Cl_2, ppm): δ 10.29 (s, 2H), 9.25 (d, J = 1.5 Hz, 2H), 8.13 (dd, J = 8.2, 1.5 Hz, 2H), 8.07 (d, J = 8.2 Hz, 2H), 7.97 (s, 2H). ^{13}C NMR (101 MHz, CD_2Cl_2, ppm): δ 192.4, 136.4, 135.4, 130.7, 130.2, 130.0, 127.3, 126.1. IR: 1619, 1613, 1198, 851, 768, 518 cm^{-1}. HRMS (ESI) m/z: $[M + H]^+$ Calcd for $C_{16}H_{10}O_2$ 235.0754; Found 235.0753.

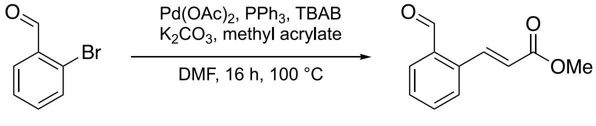

8, 98%

Methyl (*E*)-3-(2-formylphenyl)acrylate (8). A mixture of 2-bromobenzaldehyde (2.08 g, 11.2 mmol, 1.0 equiv), methyl acrylate (4.00 mL, 44.8 mmol, 4.0 equiv), PPh_3 (881 mg, 3.36 mmol, 0.30 equiv), $Pd(OAc)_2$ (378 mg, 1.68 mmol, 0.15 equiv), K_2CO_3 (1.55 g, 11.2 mmol, 1.0 equiv), tetrabutylammonium bromide (3.68 g, 11.2 mmol, 1.0 equiv), and DMF (50 mL) was heated at 100 °C for 16 h under an argon atmosphere. After cooling to room temperature, the reaction mixture was poured into aqueous HCl (2 M) and extracted with CH_2Cl_2. The combined organic layers were washed with water, dried over anhydrous $MgSO_4$, and filtered. After evaporation of the solvents, the residue was purified by column chromatography over silica gel using CH_2Cl_2 as an eluent to afford the desired product (2.09 g, 98%) as an orange solid (mp 49.0–52.1 °C). ^1H NMR (400 MHz, CD_2Cl_2, ppm): δ 10.26 (s, 1H), 8.52 (d, J = 15.9 Hz, 1H), 7.91–7.84 (m, 1H), 7.70–7.66 (m, 1H), 7.64 (dddd, J = 7.6, 7.1, 1.5, 0.5 Hz, 1H), 7.59 (dddd, J = 7.3, 7.2, 1.5, 0.5 Hz, 1H), 6.40 (d, J = 15.9 Hz, 1H), 3.81 (s, 3H). ^{13}C NMR (101 MHz, CD_2Cl_2, ppm): δ 192.5, 167.0, 141.7, 136.7, 134.3, 134.2, 133.2, 130.4, 128.3, 122.9, 52.1. IR: 1718, 1699, 1282, 1196, 1175, 766 cm^{-1}. HRMS (ESI) m/z: $[M + H]^+$ Calcd for $C_{11}H_{11}O_3$ 191.07027; Found 191.07044.

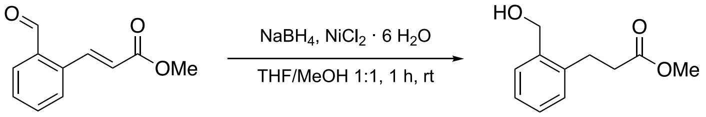

8

9, 88%

Methyl 3-(2-(hydroxymethyl)phenyl)propanoate (9). A mixture of methyl (*E*)-3-(2-formylphenyl)acrylate (**8**, 7.35 g, 38.6 mmol, 1.0 equiv), methanol (50 mL), and THF (50 mL) was cooled to 0 °C before $NaBH_4$ (5.86 g, 155 mmol, 4.0 equiv) was added in three portions. The resulting mixture was stirred at 0 °C for 15 min before nickel(II) chloride hexahydrate (4.61 g, 19.4 mmol, 0.5 equiv) was added in three portions. The cooling was removed and the mixture was stirred for 45 min. Then, the mixture was poured into aqueous HCl (2M) and extracted with CH_2Cl_2. The combined organic layers were dried over $MgSO_4$ and filtered over a pad of celite. After evaporation of solvents, the desired product was obtained as light-brown liquid (6.64 g, 88%). ^1H NMR (400 MHz, $CDCl_3$, ppm): δ 7.37 (dd, J = 7.2, 1.8 Hz, 1H), 7.31–7.15 (m, 3H), 4.73 (s, 2H), 3.66 (s, 3H), 3.04 (t, J = 7.63 Hz, 2H), 2.70 (t, J = 7.64 Hz, 2H). ^{13}C NMR (101 MHz, $CDCl_3$, ppm): δ 173.8, 138.9, 138.6, 129.2 (2 overlapped signals), 128.4, 126.9, 63.4, 51.9, 35.3, 27.1. IR: 3423, 2951, 1733, 1436, 1199, 1157, 1007, 756, 609 cm^{-1}. HRMS (EI) m/z: $[M + Na]^+$ Calcd for $C_{11}H_{14}O_3$ 217.08352; Found 217.08350.

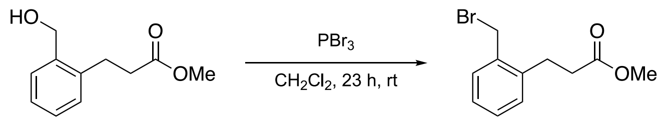

9 **10, 79%**

Methyl 3-(2-(bromomethyl)phenyl)propanoate (10). A mixture of methyl 3-(2-(hydroxymethyl)phenyl)propanoate (**9**, 1.68 g, 8.65 mmol, 1.0 equiv), phosphorus tribromide (0.85 mL, 9.0 mmol, 1.0 equiv), and CH_2Cl_2 (30 mL) was stirred at room temperature for 23 h before the mixture was carefully poured into water. The mixture was extracted with CH_2Cl_2 and the combined organic layers were dried over anhydrous $MgSO_4$ and filtered. After evaporation of the solvents, the crude product was purified by column chromatography over silica gel using CH_2Cl_2 as an eluent to afford the desired product as a pale-yellow liquid (1.76 g, 79%). ^{1}H NMR (400 MHz, CD_2Cl_2, ppm): δ 7.34 (dd, J = 7.82, 1.59 Hz, 1H), 7.30–7.25 (m, 1H), 7.21 (dd, J = 7.7, 6.1 Hz, 2H), 4.60 (s, 2H), 3.67 (s, 3H), 3.06 (dd, J = 8.6, 7.1 Hz, 2H), 2.70 (dd, J = 8.6, 7.1 Hz, 2H). ^{13}C NMR (101 MHz, CD_2Cl_2, ppm): δ 173.3, 140.1, 136.2, 131.0, 129.8, 129.6, 127.3, 51.9, 35.1, 32.1, 27.4. IR: 2951, 1732, 1436, 1210, 1160, 760, 605 cm^{-1}. HRMS (EI) m/z: $[M + Na]^+$ Calcd for $C_{11}H_{13}O_2Br$ 278.99914; Found 278.99911.

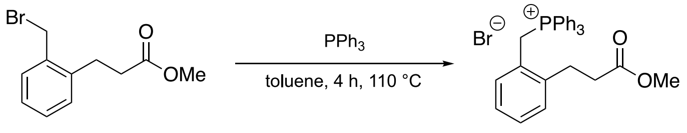

10 **11, 95%**

(2-(3-Methoxy-3-oxopropyl)benzyl)triphenylphosphonium bromide (11). A mixture of methyl 3-(2-(bromomethyl)phenyl)propanoate (**10**, 1.74 g, 6.77 mmol, 1.0 equiv), triphenylphosphine (1.86 g, 7.11 mmol, 1.1 equiv), and toluene (20 mL) was heated at 110 °C for 4 h. After cooling to room temperature, the precipitate was filtered off and extensively washed with hexanes. Drying off the precipitate under reduced pressure afforded the desired product (3.35 g, 95%) as a colorless solid (mp 217.5–218.6 °C). ^{1}H NMR (400 MHz, CD_2Cl_2, ppm): δ 7.89–7.80 (m, 3H), 7.73–7.57 (m, 12H), 7.33–7.23 (m, 1H), 7.15–6.97 (m, 3H), 5.19 (d, J = 14.1 Hz, 2H), 3.60 (s, 3H), 2.39 (t, J = 7.3 Hz, 2H), 2.15 (t, J = 7.3, 2H). ^{13}C NMR (101 MHz, CD_2Cl_2, ppm): δ 173.4, 141.7 (d, J = 5.8 Hz), 135.7 (d, J = 3.0 Hz), 134.6 (d, J = 10.0 Hz), 132.0 (d, J = 4.8 Hz), 130.6 (d, J = 12.5 Hz), 130.3 (d, J = 3.5 Hz), 129.7 (d, J = 4.1 Hz), 127.6 (d, J = 3.5 Hz), 117.8 (d, J = 85.3 Hz), 52.1, 34.9, 28.2 (d, J = 47.5 Hz), 27.1. ^{31}P NMR (162 MHz, CD_2Cl_2, ppm): δ 22.4. IR: 2988, 1729, 1437, 1111, 754, 691, 509 cm^{-1}. HRMS (EI) m/z: $[M]^+$ Calcd for $C_{29}H_{28}O_2P$ 439.18214; Found 439.18188.

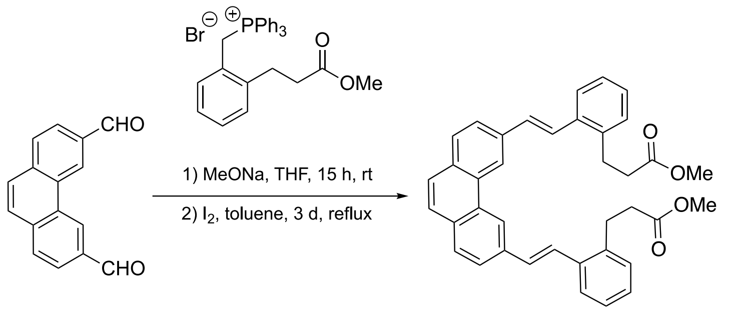

3 4, 96%

Dimethyl 3,3'-(((1*E*,1'*E*)-phenanthrene-3,6-diylbis(ethene-2,1-diyl))bis(2,1-phenylene))dipropionate (4). A mixture of methyl 3-(2-((bromotriphenyl-λ^5-phosphaneyl)methyl)phenyl)propanoate (**11**, 924 mg, 1.78 mmol, 2.1 equiv) and anhydrous THF (30 mL) was stirred at room temperature before sodium methoxide (5.4 M in MeOH, 0.30 mL, 2.2 mmol, 2.5 equiv) was added dropwise and the resulting mixture was stirred for 5 min. Then, a solution of phenanthrene-3,6-dicarbaldehyde (**3**, 203 mg, 0.867 mmol, 1.0 equiv) in anhydrous THF (20 mL) was added dropwise and the resulting mixture was stirred for 15 h at room temperature. The reaction mixture was poured into aqueous HCl (2M) solution and extracted with CH_2Cl_2. The combined organic layers were dried over anhydrous $MgSO_4$ and filtered. The solvent was evaporated and the residue was purified by column chromatography over silica gel using CH_2Cl_2 as an eluent to afford an isomeric mixture of the product. The mixture of isomers was stirred with a catalytic amount of iodine in toluene at 100 °C for 3 d before the mixture was washed with $NaHSO_3$ solution (10 wt%) and extracted with CH_2Cl_2. The combined organic layers were dried over anhydrous $MgSO_4$ and filtered. Evaporation of the solvents afforded the desired product as a pure (*E*,*E*)-isomer (461 mg, 96%) as a pale-yellow solid (mp 104.4–107.3 °C). ^{1}H NMR (400 MHz, CD_2Cl_2, ppm): δ 8.85 (s, 2H), 7.94–7.89 (m, 4H), 7.77–7.74 (m, 4H), 7.65 (d, J = 16.1 Hz, 2H), 7.38 (d, J = 16.1 Hz, 2H), 7.33–7.23 (m, 6H), 3.63 (s, 6H), 3.19 (dd, J = 8.5, 7.2 Hz, 4H), 2.67 (dd, J = 8.5, 7.2 Hz, 4H). ^{13}C NMR (101 MHz, CD_2Cl_2, ppm): δ 173.5, 139.0, 136.5, 136.4, 132.4, 131.3, 130.9, 130.0, 129.4, 128.3, 127.3, 127.1, 126.8, 126.2, 124.8, 122.2, 51.9, 35.7, 29.0. IR: 2949, 1733, 1435, 1158, 961, 837, 751, 567 cm^{-1}. HRMS (EI) m/z: $[M + H]^+$ Calcd for $C_{38}H_{34}O_4$ 555.25299; Found 555.25354.

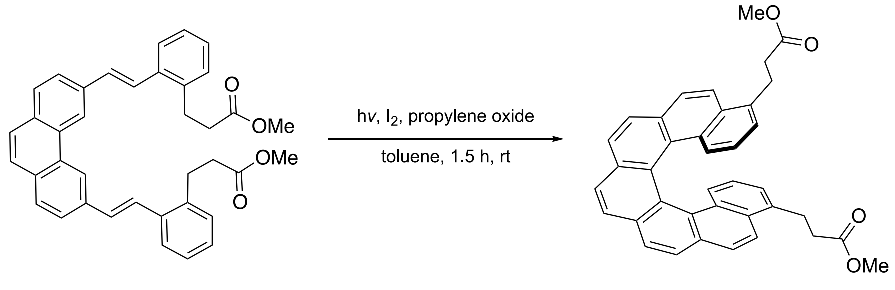

4 5, 65%

(±)-Dimethyl 3,3'-(benzo[1,2-*c*:4,3-*c*']diphenanthrene-4,15-diyl)dipropionate (5). Photocyclodehydrogenation reaction was performed in an immersion-well photoreactor system with quartz cooling tube and quartz immersion tubes containing 150 W medium-pressure mercury lamp. An argon-saturated solution of dimethyl 3,3'-(((1*E*,1'*E*)-phenanthrene-3,6-diylbis(ethene-2,1-diyl))bis(2,1-phenylene))dipropionate (**4**, 198 mg, 0.357 mmol, 1.0 equiv), iodine (203 mg, 0.785 mmol, 2.2 equiv), and propylene oxide (30 mL, 0.43 mol, 1200 equiv) in toluene (670 mL) was irradiated for 1.5 h. The resulting solution was washed with saturated $Na_2S_2O_3$ and the organic layer was separated. The solvent was evaporated and the residue was purified by column chromatography over silica gel using CH_2Cl_2/ethyl acetate (10:1) as an eluent to afford the desired product (129 mg, 65%) as a yellow solid (mp 284.8–286.5 °C). ^1H NMR (400 MHz, CD_2Cl_2, ppm): δ 8.05 (s, 2H), 8.02 (d, J = 8.2 Hz, 2H), 7.93 (d, J = 8.2 Hz, 2H), 7.79 (d, J = 8.9 Hz, 2H), 7.71 (dd, J = 8.9, 0.9 Hz, 2H), 7.00 (ddd, J = 8.5, 8.5, 0.8 Hz, 2H), 6.76 (dd, J = 7.0, 1.2 Hz, 2H), 6.28 (dd, J = 8.5, 7.0 Hz, 2H), 3.72 (s, 6H), 3.24–3.02 (m, 4H), 2.59–2.43 (m, 4H). ^{13}C NMR (101 MHz, CD_2Cl_2, ppm): δ 173.6, 135.5, 132.4, 130.8, 130.1, 129.9, 128.8, 127.5, 127.24, 127.15, 126.4, 125.8, 125.3, 123.8, 123.5, 122.7, 51.9, 35.6, 28.7. IR: 2950, 1735, 1436, 1170, 844, 775, 519 cm^{-1}. HRMS (ESI) m/z: $[M + Na]^+$ Calcd for $C_{38}H_{30}O_4$ 573.2036; Found 573.2037.

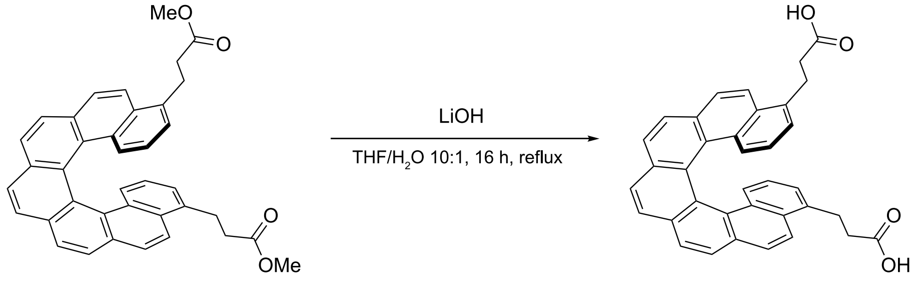

5 **12, >99%**

(±)-(3,3'-(Benzo[1,2-*c*:4,3-*c*']diphenanthrene-4,15-diyl)dipropionic acid (12). A mixture of (±)-dimethyl 3,3'-(benzo[1,2-*c*:4,3-*c*']diphenanthrene-4,15-diyl)dipropionate (**5**, 906 mg, 1.65 mmol, 1.0 equiv), lithium hydroxide monohydrate (200 mg, 3.42 mmol, 2.1 equiv), and THF/H_2O (50 mL, 4:1) was heated at 80 °C for 16 h. The reaction mixture was cooled to room temperature and concentrated under reduced pressure. The residue was poured into aqueous HCl (2 M) and extracted with CH_2Cl_2, dried over anhydrous $MgSO_4$, and filtered. Evaporation of the solvents and subsequent trituration with small amounts of cold acetone gave the desired product (855 mg, >99%) as a yellow solid (mp 251.7–252.3 °C), which was used in the next step without further purification. ^1H NMR (400 MHz, CD_3COCD_3, ppm): δ 8.16 (s, 2H), 8.12 (d, J = 8.2 Hz, 2H), 8.04 (d, J = 8.2 Hz, 2H), 7.90 (d, J = 8.9 Hz, 2H), 7.83 (dd, J = 8.9, 0.8 Hz, 2H), 6.99 (d, J = 8.5 Hz, 2H), 6.87 (dd, J = 7.0, 0.8 Hz, 2H), 6.30 (dd, J = 8.5, 7.0 Hz, 2H), 3.27–3.07 (m, 4H, partial overlap with the solvent residual peak), 2.63–2.45 (m, 4H). ^{13}C NMR (101 MHz, CD_3COCD_3/CD_3OD, ppm): δ 174.1, 136.6, 133.0, 131.4, 130.6, 130.5, 129.3, 128.2, 127.84, 127.77, 126.9, 126.1, 126.0, 123.91, 123.89, 123.4, 35.7,

29.0. IR: 3327, 2973, 2884, 1380, 1087, 1046, 880, 610 cm^{-1}. HRMS (ESI) m/z: $[M + Na]^+$ Calcd for $C_{38}H_{26}O_4$ 545.1723; Found 545.1729.

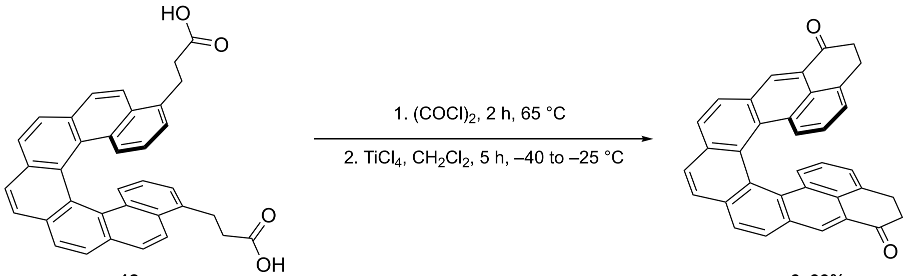

12 6, 99%

(±)-4,5,16,17-Tetrahydrobenzo[*no*]benzo[8,9]anthra[1,2-*a*]tetraphene-6,15-dione (6). A solution of (±)-(3,3'-(benzo[1,2-*c*:4,3-*c*']diphenanthrene-4,15-diyl)dipropionic acid (**12**, 76.0 mg, 0.145 mmol, 1.0 equiv) in oxalyl chloride (15 mL) was heated at 65 °C for 2 h before the excess of oxalyl chloride was removed under reduced pressure. The crude acyl chloride intermediate was dissolved in CH_2Cl_2 (30 mL) and the solution was cooled to –45 °C. $TiCl_4$ (0.20 mL, 1.8 mmol, 13 equiv) was added and the reaction mixture was stirred in the temperature range between –45 and –20 °C over 5 h. Then, it was poured into aqueous HCl (2 M) and extracted with CH_2Cl_2, dried over $MgSO_4$, and filtered. After evaporation of the solvents, the residue was purified by filtration through a short plug of silica gel using CH_2Cl_2 as an eluent to give the desired product (70.5 mg, 99%) as a yellow solid (mp 263 °C). ^1H NMR (400 MHz, CD_2Cl_2, ppm): δ 8.46 (s, 2H), 8.13 (s, 2H), 8.08 (s, 4H), 6.96 (dd, J = 8.5, 1.1 Hz, 2H), 6.86 (dddd, J = 7.0, 1.1, 1.1, 1.1 Hz, 2H), 6.35 (dd, J = 8.5, 7.0 Hz, 2H), 3.24–3.05 (m, 4H), 2.85–2.74 (m, 4H). ^{13}C NMR (101 MHz, CD_2Cl_2, ppm): δ 198.5, 133.6, 132.6, 131.7, 130.1, 129.9, 129.2, 129.1, 128.4, 128.3, 128.0, 126.0, 125.4, 124.3 (2 overlapped signals), 122.9, 39.0, 29.0. IR: 3051, 2956, 2926, 2854, 1685, 1592, 1238, 838, 768 cm^{-1}. HRMS (ESI) m/z: $[M + H]^+$ Calcd for $C_{36}H_{22}O_2$ 487.1693; Found 487.1694. $[\alpha]_D^{24.1}$ (P) + 4095° (c = 0.505, $CHCl_3$, >99% ee). $[\alpha]_D^{25.0}$ (M) – 4018° (c 0.275, $CHCl_3$, 96% ee). The enantiomers were separated using chiral-stationary-phase HPLC (ReproSil Chiral-MIA, CH_2Cl_2/hexanes (1:1), 1.0 mL min^{-1}, λ = 254 nm, $t_R(P)$ = 5.74 min, $t_R(M)$ = 6.90 min).

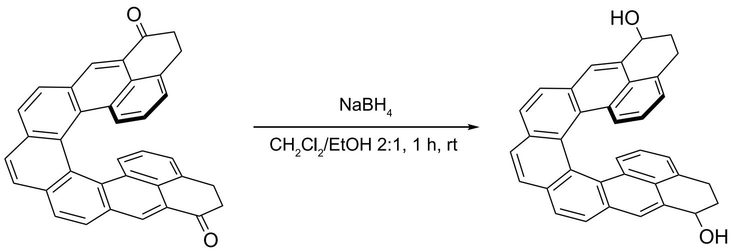

7, 88%

6

(±)-4,5,6,15,16,17-Hexahydrobenzo[*no*]benzo[8,9]anthra[1,2-*a*]tetraphene-6,15-diol (7). A solution of (±)-4,5,16,17-tetrahydrobenzo[*no*]benzo[8,9]anthra[1,2-*a*]tetraphene-

6,15-dione (**6**, 88 mg, 0.18 mmol, 1.0 equiv) and sodium borohydride (69 mg, 1.8 mmol, 1.0 equiv) in CH_2Cl_2/EtOH (30 mL, 2:1) was stirred at room temperature for 3 h before the reaction was quenched by the addition of aqueous HCl (2 M). The organic layer was separated and the aqueous layer was extracted with CH_2Cl_2. The combined organic layers were washed with saturated $NaHCO_3$, water, and brine, dried over anhydrous Na_2SO_4, and filtered. Evaporation of the solvents gave the desired product (78 mg, 88%) as a brown solid and as a mixture of three possible diastereomers, which was used in the next step without further purification. ^{1}H NMR (400 MHz, CD_3OD/CD_2Cl_2 1:1, ppm): 8.05 (s, 2H), 8.02 (d, J = 8.2 Hz, 2H), 7.93 (d, J = 8.2 Hz, 2H), 7.80 (d, J = 8.8 Hz, 2H), 7.73 (d, J = 8.9 Hz, 2H), 6.98 (d, J = 8.5 Hz, 2H), 6.78 (d, J = 7.0 Hz, 2H), 6.26 (dd, J = 8.6, 7.0 Hz, 2H), 3.20–3.03 (m, 4H), 2.59–2.36 (m, 4H). ^{13}C NMR (101 MHz, CD_3OD/CD_2Cl_2, ppm): δ 176.4, 136.1, 132.9, 131.3, 130.6, 130.5, 129.3, 127.9, 127.7, 127.6, 126.8, 126.2, 125.8, 124.2, 123.9, 123.1, 36.1, 29.1. Within the resolution limit, all three isomers show the same ^{1}H and ^{13}C shifts. IR: 3357, 2917, 2849, 1706, 1463, 882, 833, 769, 731, 613 cm^{-1}. HRMS (ESI) m/z: $[M + Na]^+$ Calcd for $C_{36}H_{26}O_2$ 513.1825; Found 513.1830.

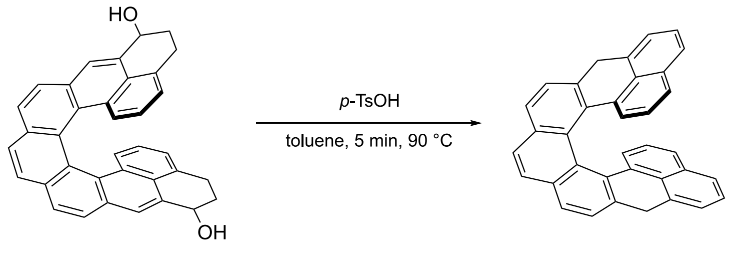

7

2*H*-NC, 96%

(±)-7,14-Dihydrobenzo[*no*]benzo[8,9]anthra[1,2-*a*]tetraphene (2*H*-NC). *p*-Toluenesulfonic acid monohydrate (9 mg, 0.05 mmol, 0.3 equiv) was added to a hot (90 °C) solution of (±)-4,5,6,15,16,17-hexahydrobenzo[*no*]benzo[8,9]anthra[1,2-*a*]tetraphene-6,15-diol (**7**, 80 mg, 0.16 mmol, 1.0 equiv) in toluene (15 mL) under an argon atmosphere and the reaction mixture was heated at 90 °C for 5 min before it was cooled in an ice bath and passed through a pad of silica gel using argon-saturated toluene/hexane (1:1) as an eluent. Evaporation of the solvents afforded the desired product (71 mg, 96%) as a yellow solid. ^{1}H NMR (500 MHz, CD_2Cl_2, ppm): δ 7.69 (d, J = 7.8 Hz, 2H), 7.62 (s, 2H), 7.40 (ddd, J = 7.8, 0.8, 0.8 Hz, 2H), 7.25 (dd, J = 8.0, 6.9 Hz, 2H), 7.22–7.12 (m, 4H), 7.01 (dd, J = 7.1, 1.2 Hz, 2H), 6.55 (dd, J = 8.1, 1.2 Hz, 2H), 6.49 (dd, J = 8.1, 7.1 Hz, 2H), 4.41 (d, J = 22.2 Hz, 2H), 4.30 (d, J = 22.2 Hz, 2H). ^{13}C NMR (126 MHz, CD_2Cl_2, ppm): δ 135.0, 133.5, 133.4, 133.2, 132.6, 130.8, 129.7, 127.30, 127.28, 126.8, 126.2, 125.4, 125.3, 125.2, 124.6, 123.5, 122.9, 35.5. HRMS (ESI) m/z: $[M - H]^+$ Calcd for $C_{36}H_{22}$ 453.1638; Found 453.1640.

Note: Elevated temperatures are required to solubilize the acid in toluene. Argon-saturated solvents for the reaction and for the purification increase the yield significantly.

Route B:

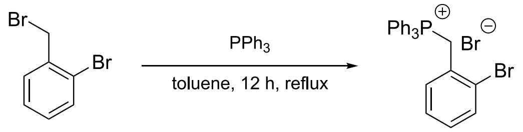

13, 99%

(2-Bromobenzyl)triphenylphosphonium bromide (13). A mixture of 2-bromobenzyl bromide (4.00 g, 16.0 mmol, 1.0 equiv), triphenyl phosphine (6.71 g, 25.6 mmol, 1.6 equiv), and toluene (90 mL) was stirred at reflux for 12 h. After cooling to room temperature, the precipitate was filtered off and extensively washed with hexanes. Drying off the precipitate under reduced pressure afforded the desired product (8.10 g, 99%) as a colorless solid (mp 182.1–183.0 °C). ^{1}H NMR (400 MHz, CD_2Cl_2, ppm): δ 7.90–7.78 (m, 3H), 7.71–7.60 (m, 12H), 7.49–7.36 (m, 2H), 7.26–7.15 (m, 2H), 5.43 (d, J = 14.2 Hz, 2H). ^{13}C NMR (101 MHz, CD_2Cl_2, ppm): δ 135.7 (d, J = 3.2 Hz), 134.7 (d, J = 9.9 Hz), 133.6 (d, J = 3.2 Hz), 133.0 (d, J = 4.9 Hz), 130.9 (d, J = 3.8 Hz), 130.6 (d, J = 12.7 Hz), 128.7 (d, J = 3.4 Hz), 128.0 (d, J = 8.6 Hz), 127.6 (d, J = 6.6 Hz), 117.5 (d, J = 86.1 Hz), 31.6 (d, J = 49.0 Hz). ^{31}P NMR (162 MHz, CD_2Cl_2, ppm): δ 22.5. IR: 3390, 3052, 2848, 2774, 1586, 1437, 1111, 754, 690, 509 cm^{-1}. HRMS (EI) m/z: $[M]^+$ Calcd for $C_{25}H_{21}BrP$ 431.05588; Found 431.05621.

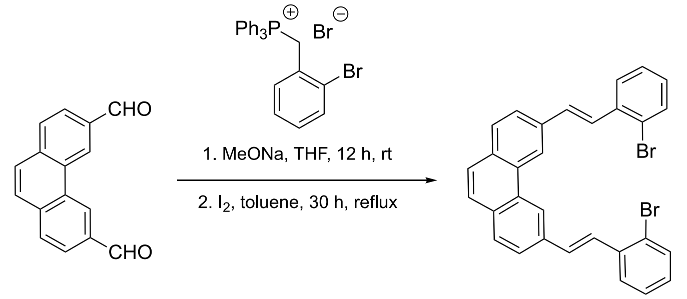

3 **14, 88%**

3,6-Bis(2-bromostyryl)phenanthrene (14). To a mixture of phenanthrene-3,6-dicarbaldehyde (**3**, 229 mg, 0.978 mmol, 1.0 equiv), (2-bromobenzyl)triphenylphosphonium bromide (**13**, 1.03 g, 2.01 mmol, 2.1 equiv), and THF (15 mL), sodium methoxide (5.4 M in MeOH, 0.35 mL, 2.5 mmol, 2.6 equiv) was added dropwise and the resulting mixture was stirred for 12 h at room temperature. Then, the reaction mixture was poured into aqueous HCl (2 M) and extracted with CH_2Cl_2. The combined organic layers were dried over anhydrous $MgSO_4$, filtered, and the solvent was evaporated under reduced pressure. The residue was purified by column chromatography over silica gel using cyclohexane/ethyl acetate (20:1) as an eluent to afford the product as a mixture of isomers. The isomeric mixture dissolved in toluene was stirred with a catalytic amount of iodine at 100 °C for 30 h before it was washed with aqueous $NaHSO_3$ (10 wt%) and extracted with CH_2Cl_2. The combined organic layers were dried over anhydrous $MgSO_4$ and filtered. After evaporation of the solvents, the desired product was afforded as a pure (E,E)-isomer (467 mg, 88%) as a

colorless solid (mp 229.8–230.4 °C). ^{1}H NMR (400 MHz, CD_2Cl_2, ppm): δ 8.84 (br s, 2H), 7.93 (m, *J* = 1.0 Hz, 4H), 7.83 (dd, *J* = 7.8, 1.7 Hz, 2H), 7.76 (s, 2H), 7.73 (d, *J* = 16.2 Hz, 2H), 7.65 (dd, *J* = 8.0, 1.2 Hz, 2H), 7.40 (d, 16.2 Hz 2H), 7.40 (ddd, *J* = 7.3, 1.3, 0.6 Hz, 2H), 7.19 (ddd, *J* = 8.0, 7.3, 1.7 Hz, 2H). ^{13}C NMR (101 MHz, CD_2Cl_2, ppm): δ 137.5, 135.8, 133.5, 132.7, 132.1, 130.8, 129.6, 129.4, 128.3, 128.2, 127.3, 127.2, 125.0, 124.6, 122.3. IR: 1463, 1020, 971, 971, 956, 857, 838, 749, 665, 566 cm^{-1}. HRMS (EI) *m/z*: [*M*]$^+$ Calcd for $C_{30}H_{20}Br_2$ 537.99263; Found 537.99215.

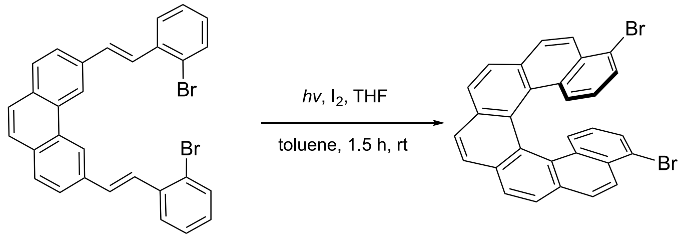

14 **15, 76%**

(±)-4,15-Dibromobenzo[1,2-*c*:4,3-*c*']diphenanthrene (15). The photocyclodehydrogenation reaction was performed using an immersion-well photoreactor system equipped with quartz cooling and immersion tubes and a 150 W medium-pressure mercury lamp. An argon-saturated solution of 3,6-bis(2-bromostyryl)phenanthrene (**14**, 200 mg, 0.370 mmol, 1.0 equiv), iodine (193 mg, 0.760 mmol, 2.1 equiv), and THF (3.1 mL, 44 mmol, 119 equiv) in toluene (750 mL) was irradiated for 1 h. The resulting solution was washed with saturated $Na_2S_2O_3$, water, and brine. The solvent was evaporated and the residue was purified by column chromatography over silica gel using cyclohexane as an eluent to afford the desired product (150 mg, 76%) as a yellow solid (mp 284.8–286.5 °C). ^{1}H NMR (400 MHz, CD_2Cl_2, ppm): δ 8.08 (s, 2H), 8.06 (d, *J* = 8.2 Hz, 2H), 7.94 (d, *J* = 8.2 Hz, 2H), 7.91 (dd, *J* = 8.8, 1.0 Hz, 2H), 7.83 (d, *J* = 8.8 Hz, 2H), 7.25 (dd, *J* = 7.5, 1.0 Hz, 2H), 7.06 (ddd, *J* = 8.5, 1.0, 1.0 Hz, 2H), 6.22 (dd, *J* = 8.5, 7.5 Hz, 2H). ^{13}C NMR (101 MHz, CD_2Cl_2, ppm): δ 132.7, 131.4, 131.0, 130.4, 129.0, 128.1, 127.8, 127.7, 127.6, 127.5, 126.0, 125.4, 124.4, 124.3, 121.8. IR: 3040, 1485, 1067, 842, 768, 698, 517 cm^{-1}. HRMS (EI) *m/z*: [*M*]$^+$ Calcd for $C_{30}H_{16}Br_2$ 533.96133; Found 533.96092.

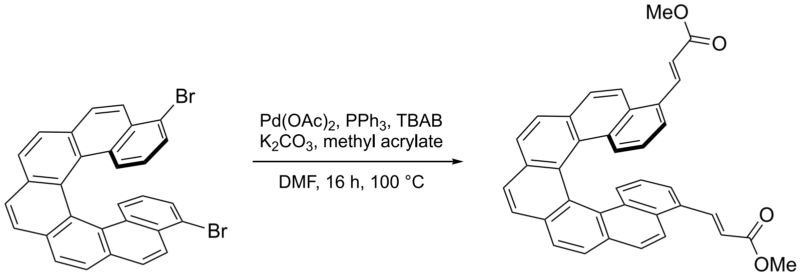

15 **16, 89%**

(±)-Dimethyl 3,3'-(benzo[1,2-*c*:4,3-*c*']diphenanthrene-4,15-diyl)(2*E*,2'*E*)-diacrylate (16). A mixture of (±)-4,15-dibromobenzo[1,2-*c*:4,3-*c*']diphenanthrene (**15**, 200 mg, 0.373

mmol, 1.0 equiv), methyl acrylate (0.27 mL, 3.0 mmol, 8.1 equiv), PPh_3 (59 mg, 0.22 mmol, 0.6 equiv), $Pd(OAc)_2$ (25 mg, 0.11 mmol, 0.3 equiv), K_2CO_3 (103 mg, 0.746 mmol, 2.0 equiv), tetrabutylammonium bromide (240 mg, 0.746 mmol, 2.0 equiv), and DMF (10 mL) was heated at 110 °C for 15 h under an argon atmosphere. After cooling to room temperature, the reaction mixture was poured into aqueous HCl (2 M) and extracted with CH_2Cl_2. The combined organic layers were washed with brine, dried over anhydrous Na_2SO_4, and filtered. After evaporation of the solvents, the residue was purified by column chromatography over silica gel using cyclohexane/ethyl acetate (4:1) as an eluent to afford the pure product (181 mg, 89%) as a pale-yellow solid (mp 201.1–204.1 °C). ^1H NMR (400 MHz, CD_2Cl_2, ppm): δ 8.21 (d, J = 15.7 Hz, 2H), 8.08 (s, 2H), 8.05 (d, J = 8.2 Hz, 2H), 7.94 (d, J = 8.2 Hz, 2H), 7.84 (dd, J = 8.7, 0.7 Hz, 2H), 7.18 (ddd, J = 8.4, 7.2, 1.0 Hz, 2H), 7.15 (ddd, J = 7.3, 1.2, 0.6 Hz, 2H), 6.37 (ddd, J = 8.4, 7.3, 0.6 Hz, 2H), 6.23 (d, J = 15.7 Hz, 2H), 3.83 (s, 6H). ^{13}C NMR (101 MHz, CD_2Cl_2, ppm): δ 167.5, 142.0, 132.6, 131.1, 130.7, 130.1, 130.0, 128.0, 127.7, 127.5, 127.4, 127.2, 126.6, 125.5, 124.1, 123.6, 122.6, 120.4, 51.9. IR: 2949, 1713, 1630, 1434, 1315, 1271, 1171, 978, 844, 517 cm^{-1}. HRMS (ESI) m/z: $[M + Na]^+$ Calcd for $C_{38}H_{26}O_4$ 569.1723; Found 569.1720.

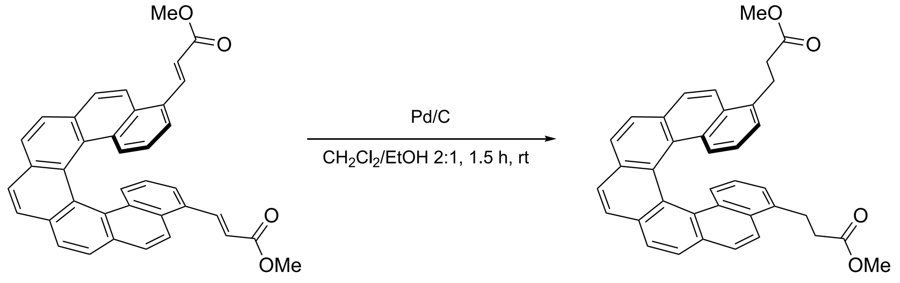

16 **5, 97%**

(±)-Dimethyl 3,3'-(benzo[1,2-*c*:4,3-*c*']diphenanthrene-4,15-diyl)dipropionate (5). (±)-Dimethyl 3,3'-(benzo[1,2-*c*:4,3-*c*']diphenanthrene-4,15-diyl)(2*E*,2'*E*)-diacrylate (**16**, 180 mg, 0.329 mmol, 1.0 equiv) and Pd/C (35 mg, 10% palladium on charcoal) were suspended in CH_2Cl_2/EtOH (40 mL, 1:1) and hydrogen gas was passed through the suspension for 30 min. The reaction mixture was then stirred at room temperature under a hydrogen atmosphere for 100 min before it was passed through celite and concentrated in vacuum to give the desired product (175 mg, 97%) as a colorless solid, which was used in the next step without further purification. ^1H NMR (400 MHz, CD_2Cl_2, ppm): δ 8.05 (s, 2H), 8.02 (d, J = 8.2 Hz, 2H), 7.93 (d, J = 8.2 Hz, 2H), 7.79 (d, J = 8.9 Hz, 2H), 7.71 (dd, J = 8.9, 0.9 Hz, 2H), 7.00 (ddd, J = 8.5, 1.2, 1.2 Hz, 2H), 6.76 (dd, J = 7.0, 1.2 Hz, 2H), 6.28 (dd, J = 8.5, 7.0 Hz, 2H), 3.72 (s, 6H), 3.24–3.02 (m, 4H), 2.59–2.43 (m, 4H). ^{13}C NMR (101 MHz, CD_2Cl_2, ppm): δ 173.6, 135.5, 132.4, 130.8, 130.1, 129.9, 128.8, 127.5, 127.24, 127.15, 126.4, 125.8, 125.3, 123.8, 123.5, 122.7, 51.9, 35.6, 28.7. IR: 2950, 1735, 1436, 1170, 844, 775, 519 cm^{-1}. HRMS (ESI) m/z: $[M + Na]^+$ Calcd for $C_{38}H_{30}O_4$ 573.2036; Found 573.2037.

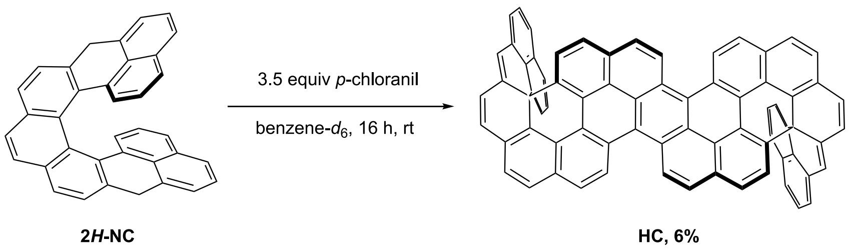

Hypercethrene (HC). To a solution of (±)-7,14-dihydrobenzo[*no*]benzo[8,9]anthra[1,2-*a*]tetraphene (2*H*-**NC**, 4.75 mg, 10.4 μmol, 1.0 equiv) in argon-saturated benzene-d_6 (0.7 mL) in an NMR tube, an argon-saturated solution of *p*-chloranil (0.05 M in benzene-d_6, 750 μL, 37.5 μmol, 3.6 equiv) was added before the tube was shaken and left at room temperature for 16 h. The crude mixture was purified by preparative TLC with toluene/cyclohexane (1:1) as an eluent and subsequently by GPC to obtain hypercethrene (292 μg, 6%) as an orange amorphous solid. ^{1}H NMR (600 MHz, $C_2D_2Cl_4$, ppm): δ 9.25 (d, J = 9.1 Hz, 2H), 8.82 (d, J = 8.8 Hz, 2H), 8.36 (d, J = 8.3 Hz, 2H), 8.04 (d, J = 8.8 Hz, 2H), 8.02 (d, J = 9.1 Hz, 2H), 8.01 (d, J = 8.0 Hz, 2H), 7.92 (d, J = 8.3 Hz, 2H), 7.694 (d, J = 8.0 Hz, 2H), 7.685 (s, 2H), 7.66 (dd, J = 7.5, 1.1 Hz, 2H), 7.60 (dd, J = 7.5, 1.1 Hz, 2H), 7.43 (dd, J = 7.5, 1.1 Hz, 2H), 7.24 (d, J = 9.4 Hz, 2H), 7.21 (dd, J = 7.9, 1.0 Hz, 2H), 7.06 (dd, J = 7.9, 1.0 Hz, 2H), 6.84 (d, J = 9.4 Hz, 2H), 6.81 (dd, J = 8.0, 7.9 Hz, 2H). ^{13}C NMR (151 MHz, $C_2D_2Cl_4$, ppm): δ 141.0, 135.5, 135.2, 133.2, 132.5, 131.9, 130.3, 130.2, 129.64, 129.57, 128.7, 128.5, 128.4, 127.4, 127.32, 127.30, 127.2, 127.0, 126.76, 126.75, 126.17, 126.16, 125.8, 125.3, 125.13, 125.12, 124.63, 124.61, 124.5, 123.8, 123.3, 122.5, 122.0, 120.8, 120.2, 50.0. HRMS (APCI) m/z: $[M + H]^+$ Calcd for $C_{72}H_{34}$ 899.27333; Found 899.27377.

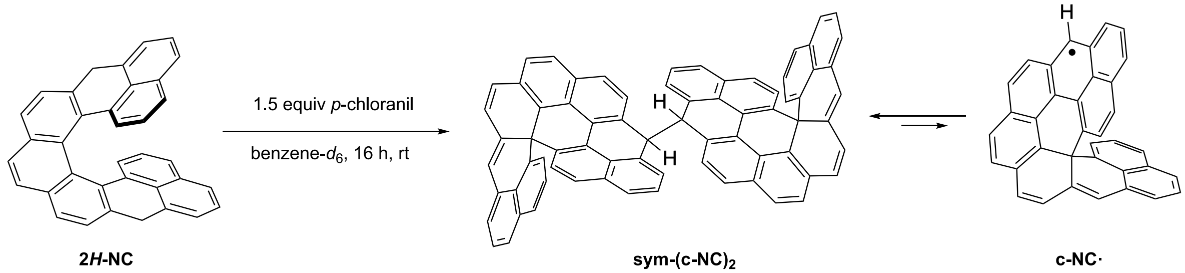

sym-(*P,S,R,M*)-(c-NC)$_2$ & sym-(*P*,R*,R*,P)-(c-NC)$_2$.** To a solution of (±)-7,14-dihydrobenzo[*no*]benzo[8,9]anthra[1,2-*a*]tetraphene (2*H*-**NC**, 2.28 mg, 5.02 μmol, 1.0 equiv) in argon-saturated benzene-d_6 (0.5 mL) in an NMR tube, an argon-saturated solution of *p*-chloranil (0.05 M in benzene-d_6, 150 μL, 7.53 μmol, 1.5 equiv) was added before the tube was shaken and left at room temperature for 16 h. The desired product was characterized by NMR and XRD but it was not isolated due to its reactivity. sym-(*P,S,R,M*)-(c-**NC**)$_2$: ^{1}H NMR (500 MHz, C_6D_6, ppm): δ 7.99 (d, J = 8.7 Hz, 1H), 7.77 (d, J = 8.1 Hz, 1H), 7.63 (dd, J = 7.3, 1.3 Hz, 1H), 7.58 (d, J = 8.3 Hz, 1H), 7.44–7.30 (m, 5H), 7.23–7.19 (m, 3H), 7.13–7.11 (m, 2H), 6.88 (d, J = 9.4 Hz, 1H), 6.56 (d, J = 9.3 Hz, 1H), 6.34 (dd, J = 8.1, 7.0 Hz, 1H), 4.76 (d, J = 7.1 Hz, 1H), 3.90 (s, 1H). ^{13}C NMR (126 MHz, C_6D_6, ppm): δ 144.0, 136.5, 136.0, 135.4,

133.6, 133.6, 133.1, 133.0, 131.4, 131.1, 131.0, 130.6, 129.9, 129.7, 129.7, 129.1, 127.9, 127.9, 127.7, 127.6, 127.4, 126.7, 126.5, 126.4, 126.3, 126.0, 126.0, 125.9, 125.6, 124.8, 124.3, 122.0, 121.7, 121.1, 58.2, 50.4. sym-(*P*,R*,R*,P**)-(c-**NC**)$_2$: ^{1}H NMR (500 MHz, C_6D_6, ppm): δ 8.06 (d, *J* = 8.8 Hz, 1H), 7.48 (d, *J* = 8.1 Hz, 1H), 7.47 (d, *J* = 8.9 Hz, 1H), 7.48–7.29 (m, 7H), 7.23–7.19 (m, 2H), 7.13–7.11 (m, 1H), 6.98 (d, *J* = 8.4 Hz, 1H), 6.95–6.92 (m, 2H), 6.84 (dd, *J* = 9.1, 0.9 Hz, 1H), 6.48 (d, *J* = 9.1 Hz, 1H), 5.31 (d, *J* = 8.3 Hz, 1H), 3.93 (s, 1H). ^{13}C NMR (126 MHz, C_6D_6, ppm): δ 143.8, 136.5, 135.5, 135.4, 134.7, 133.6, 133.3, 133.0, 131.9, 130.9, 130.8, 130.6, 129.7, 129.5, 129.4, 128.3, 127.8, 127.7, 127.7, 127.4, 127.2, 126.6, 126.4, 126.3, 126.3, 126.3, 125.9, 125.7, 125.6, 124.8, 124.8, 122.4, 121.1, 121.1, 57.7, 50.3.

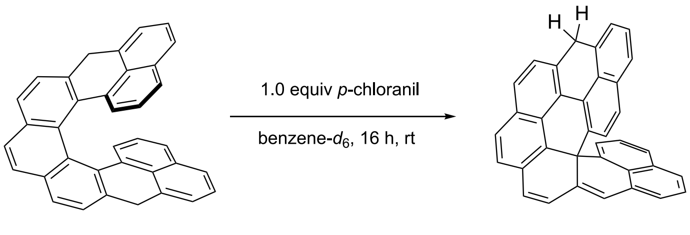

2*H*-NC c-NC

(±)-19*H*-Dibenzo[*cd,ghi*]phenaleno[2,1-*p*]perylene (c-NC). To a solution of (±)-7,14-dihydrobenzo[*no*]benzo[8,9]anthra[1,2-*a*]tetraphene (2*H*-**NC**, 2.28 mg, 5.02 μmol, 1.0 equiv) in argon-saturated benzene-d_6 (0.5 mL) in an NMR tube, an argon-saturated solution of *p*-chloranil (0.05 M in benzene-d_6, 350 μL, 17.6 μmol, 3.5 equiv) was added before the tube was shaken and left at room temperature for 16 h. Then, the residue was purified by column chromatography over silica gel with argon-saturated heptane/toluene (2:1) as an eluent to afford the desired product for NMR characterization. ^{1}H NMR (500 MHz, C_6D_6, ppm): δ 7.94 (d, *J* = 8.9 Hz, 1H), 7.68 (d, *J* = 8.3 Hz, 1H), 7.67 (d, *J* = 8.2 Hz, 1H), 7.42 (d, *J* = 8.1 Hz, 1H), 7.36 (d, *J* = 7.1 Hz, 1H), 7.30 (d, *J* = 8.1 Hz, 1H), 7.22 (s, 1H), 7.21–7.20 (m, 1H), 7.18 (d, *J* = 8.9 Hz, 1H), 7.15–7.12 (m, 3H), 7.05–7.01 (m, 1H, overlapped with toluene), 6.98 (dd, *J* = 8.1, 6.9 Hz, 1H), 6.86 (d, *J* = 6.9 Hz, 1H), 6.81 (dd, *J* = 9.2 Hz, 1H), 6.71 (dd, *J* = 7.8, 7.8 Hz, 1H), 6.47 (d, *J* = 9.2 Hz, 1H), 4.31 (d, *J* = 21.2 Hz, 1H), 3.91 (d, *J* = 21.2 Hz, 1H). ^{13}C NMR (126 MHz, C_6D_6, ppm): δ 35.0, 50.1, 121.0, 121.4, 122.2, 124.6, 124.6, 125.2, 125.4, 125.5, 126.0, 126.0, 126.2, 126.5, 126.5, 127.2, 127.4, 127.5, 127.5, 127.5, 128.0, 129.1, 129.7, 129.8, 130.5, 130.7, 130.8, 131.7, 132.3, 133.1, 133.5, 133.5, 133.5, 134.3, 136.4, 143.6.

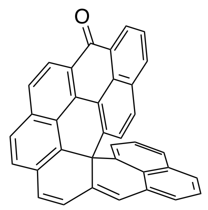

***O*-c-NC**

(±)-19*H*-Dibenzo[*cd,ghi*]phenaleno[2,1-*p*]perylen-19-one (*O*-c-NC). This compound was isolated as a byproduct from the residues of the oxidations with *p*-chloranil by column chromatography over silica gel using heptane/toluene (2:1) as an eluent to obtain the desired product as a brown solid. ^{1}H NMR (500 MHz, C_6D_6, ppm): δ 8.78 (d, *J* = 8.6 Hz, 1H), 8.62 (dd, *J* = 7.2, 1.3 Hz, 1H), 7.97 (d, *J* = 8.9 Hz, 1H), 7.67 (d, *J* = 8.7 Hz, 1H), 7.53 (d, *J* = 8.2 Hz, 1H), 7.37 (dd, *J* = 8.1, 1.2 Hz, 1H), 7.34 (d, *J* = 7.0 Hz, 1H), 7.23–7.18 (m, 3H), 7.14 (dd, *J* = 8.2, 1.2 Hz, 1H), 7.06 (dd, *J* = 7.6, 1.0 Hz, 1H), 7.06 (d *J* = 9.1 Hz, 1H), 6.97 (dd, *J* = 8.2, 1.0 Hz, 1H), 6.89 (dd, *J* = 8.1, 7.1 Hz, 1H), 6.77 (dd, *J* = 9.2, 0.8 Hz, 1H), 6.49 (dd, *J* = 8.1, 7.6 Hz, 1H), 6.40 (d, *J* = 9.2 Hz, 1H). ^{13}C NMR (126 MHz, C_6D_6, ppm): δ 183.7, 142.9, 142.0, 135.9, 134.4, 134.0, 133.7, 133.4, 133.3, 132.5, 132.0, 131.1, 131.0, 129.90, 129.85, 129.8, 129.3, 128.5 (2 overlapped signals), 128.20, 128.17, 128.16, 127.9, 127.7 (2 overlapped signals), 126.4, 126.2, 126.1, 125.4, 124.2, 124.0, 122.0, 121.8, 121.1, 120.9, 50.8. HRMS (ESI) *m*/*z*: [*M* – H]$^+$ Calcd for $C_{36}H_{18}O$ 467.1430; Found 467.1430.

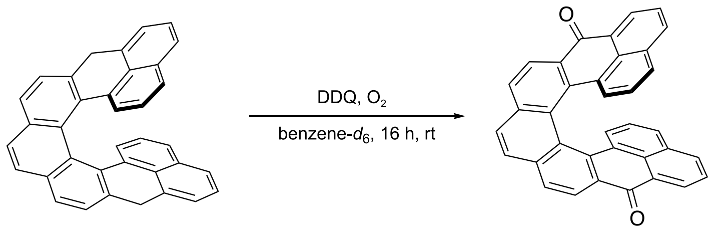

(±)-Benzo[*no*]benzo[8,9]anthra[1,2-*a*]tetraphene-7,14-dione (2*O*-NC). To a solution of (±)-7,14-dihydrobenzo[*no*]benzo[8,9]anthra[1,2-*a*]tetraphene (2*H*-**NC**, 2.65 mg, 5.84 µmol, 1.0 equiv) in argon-saturated benzene-d_6 (0.5 mL) in an NMR tube, an argon-saturated solution of 2,3-dichloro-5,6-dicyano-1,4-benzoquinone (DDQ, 0.05 M in benzene-d_6, 375 µL, 188 µmol, 3.2 equiv) was added before the tube was shaken and left at room temperature for 16 h. The crude mixture was purified by column chromatography over silica gel using CH_2Cl_2 as an eluent to obtain 2*O*-**NC** as a dark-yellow solid (1.20 mg, 43%). ^{1}H NMR (500 MHz, C_6D_6, ppm): δ 8.82 (d, *J* = 8.1 Hz, 2H), 8.68 (dd, *J* = 7.1, 1.4 Hz, 2H), 7.47 (d, *J* = 7.3 Hz, 2H), 7.46 (d, *J* = 7.9 Hz, 2H), 7.39 (s, 2H), 7.13 (dd, *J* = 8.0, 1.4 Hz, 2H), 7.07 (dd, *J* = 7.8, 7.2 Hz, 2H), 6.49 (d, *J* = 8.0 Hz, 2H), 6.15 (dd, *J* = 7.8, 7.6 Hz, 2H). ^{13}C NMR (126 MHz, C_6D_6, ppm): δ 182.5, 139.1, 136.7, 134.4, 132.3, 131.7, 129.1, 128.7, 128.6, 128.2, 128.0, 127.9, 127.7, 127.2, 126.7, 126.0, 125.7, 124.9. HRMS (ESI) *m*/*z*: [*M* + H]$^+$ Calcd for $C_{36}H_{18}O_2$ 483.1380; Found 483.1390.

S3. UV–Vis spectroscopy

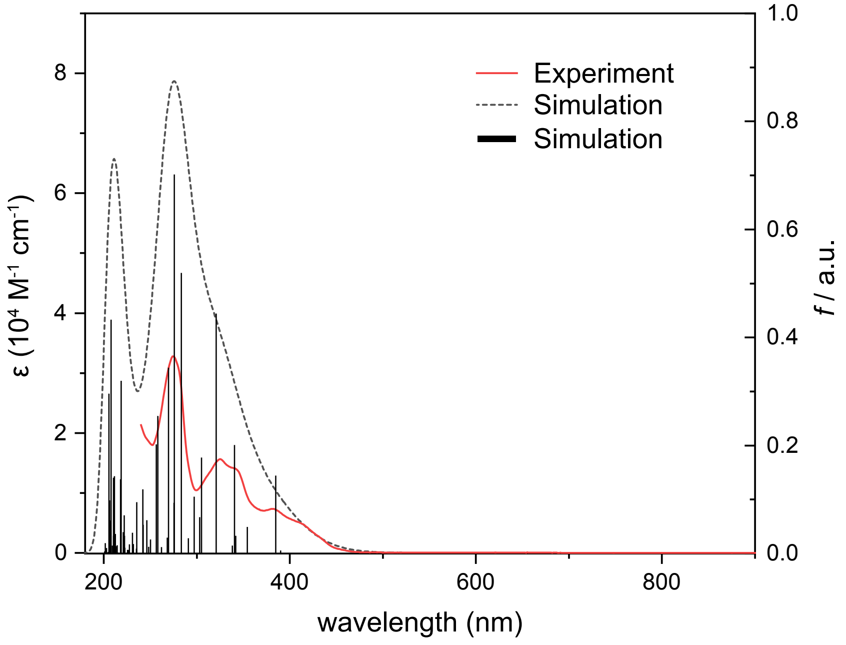

Figure S4. Comparison of the UV–Vis spectrum of (*P*)-**6** (red line) in toluene with the spectrum simulated by TD-DFT calculations (black dashed line and black vertical lines; spectrum shifted by 0.43 eV; ωB97XD/Def2SVP(toluene)). *f* = oscillator strength.

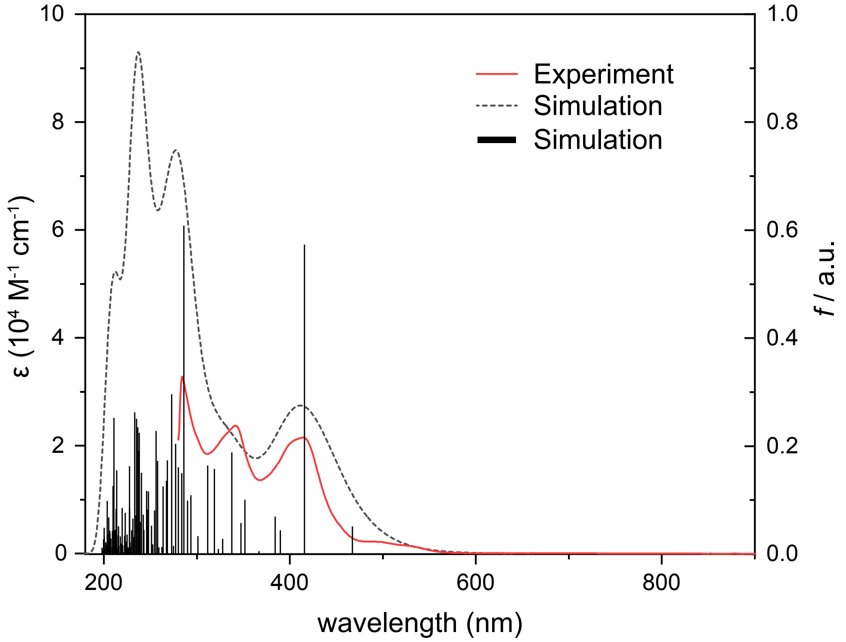

Figure S5. Comparison of the UV–Vis spectrum of (*P*)-*O*-c-**NC** (red line) in toluene with the spectrum simulated by TD-DFT calculations (black dashed line and black vertical lines; spectrum shifted by 0.32 eV; ωB97XD/Def2SVP(toluene)). *f* = oscillator strength.

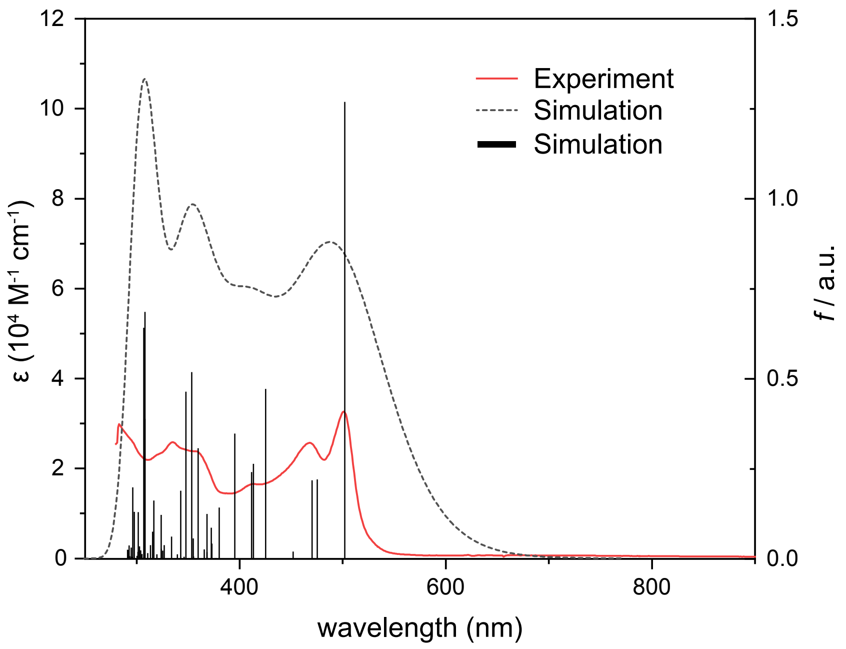

Figure S6. Comparison of the UV–Vis spectrum of (*P,P*)-**HC** (red line) in toluene with the spectrum simulated by TD-DFT calculations (black dashed line and black vertical lines; spectrum shifted by 0.40 eV; ωB97XD/Def2SVP(toluene)). *f* = oscillator strength.

S4. HPLC separation of enantiomers of (±)-6

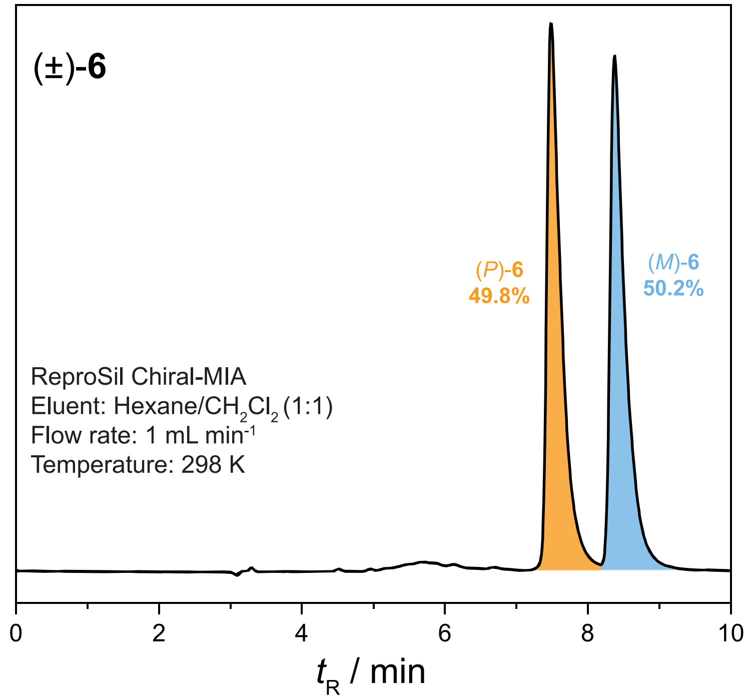

Figure S7. HPLC chromatogram for the separation of enantiomers of (±)-**6**. The absolute configurations of the enantiomers were assigned with the help of TD-DFT calculations and single crystal X-ray diffraction (see Fig. S16).

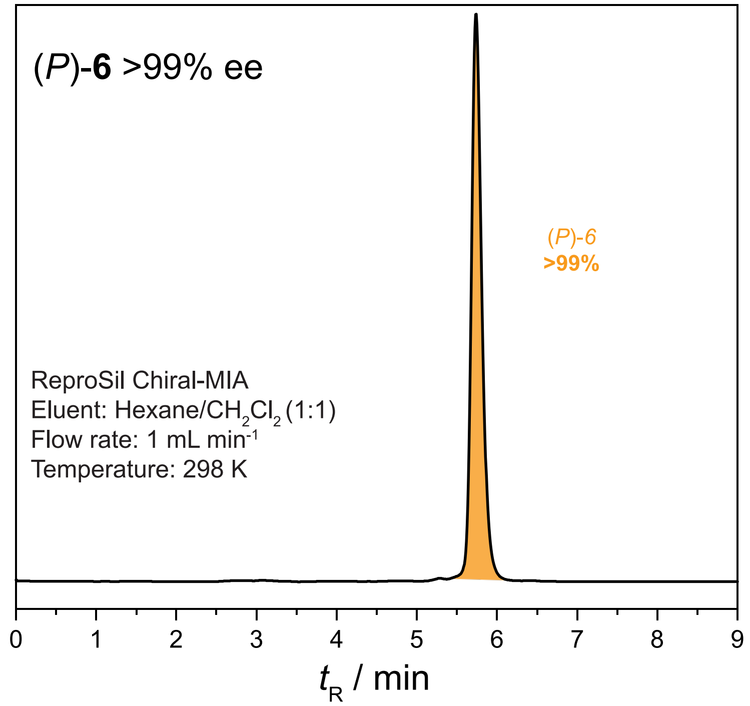

Figure S8. HPLC chromatogram for the enantiopurity check of (*P*)-**6**.

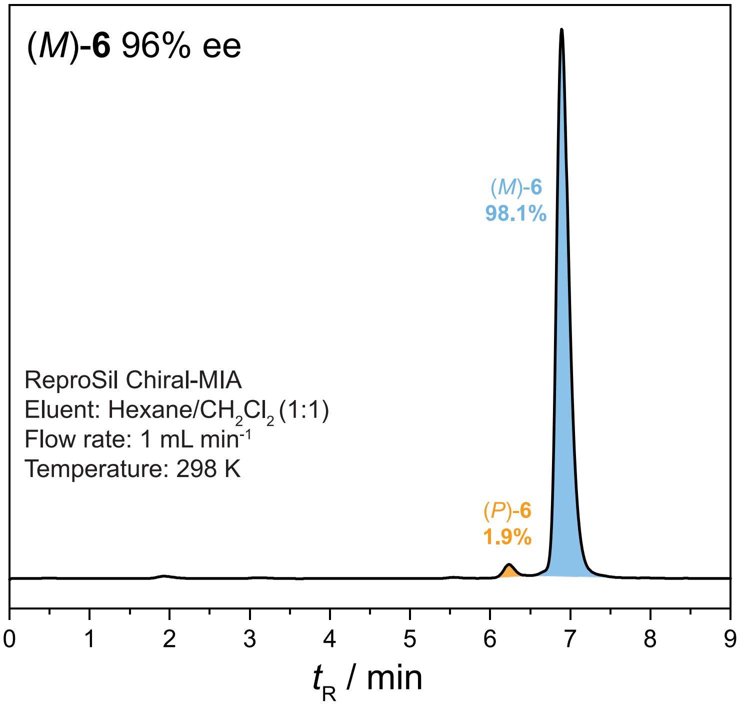

Figure S9. HPLC chromatogram for the enantiopurity check of (*M*)-**6**.

S5. Circular dichroism (CD) and circularly polarized luminescence (CPL) spectroscopy

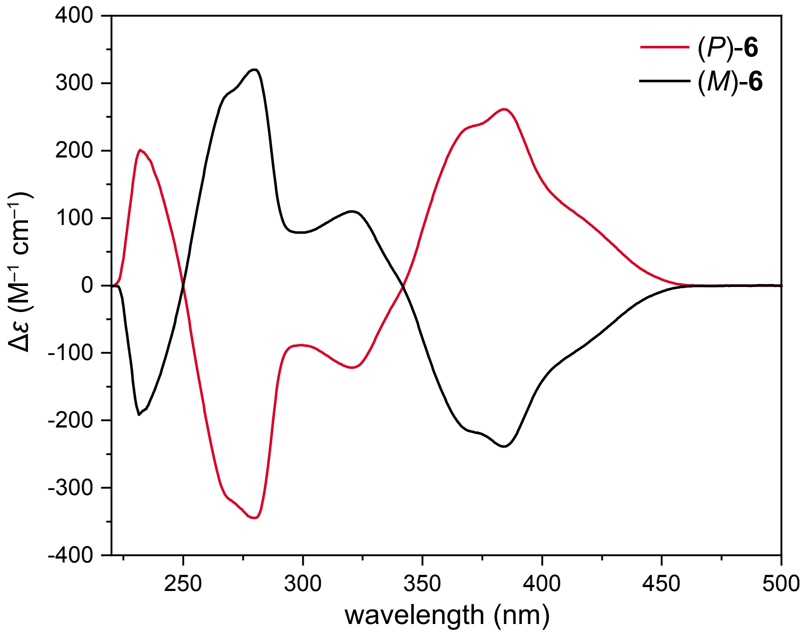

Figure S10. Measured CD spectra of (*P*)-**6** (red line) and (*M*)-**6** (black line) in toluene.

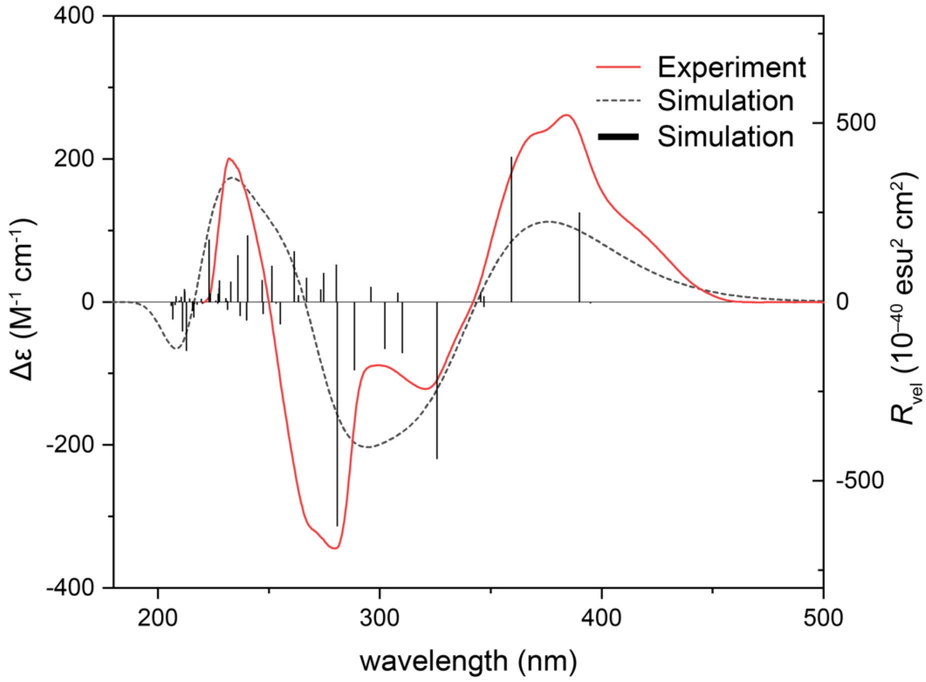

Figure S11. Comparison of the CD spectrum of (*P*)-**6** (red line) in toluene with the spectrum simulated by TD-DFT calculations (black dashed line and black vertical lines; spectrum shifted by 0.26 eV; ωB97XD/Def2SVP(toluene)).

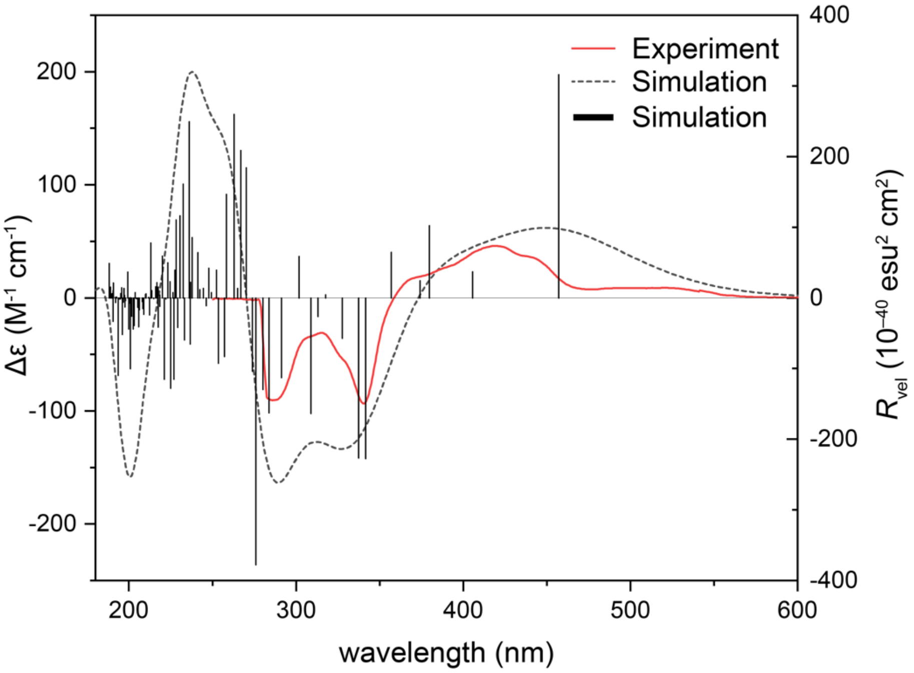

Figure S12. Comparison of the CD spectrum of (*P*)-*O*-c-**NC** (red line) in toluene with the spectrum simulated by TD-DFT calculations (black dashed line and black vertical lines; spectrum shifted by 0.34 eV; ωB97XD/Def2SVP(toluene)).

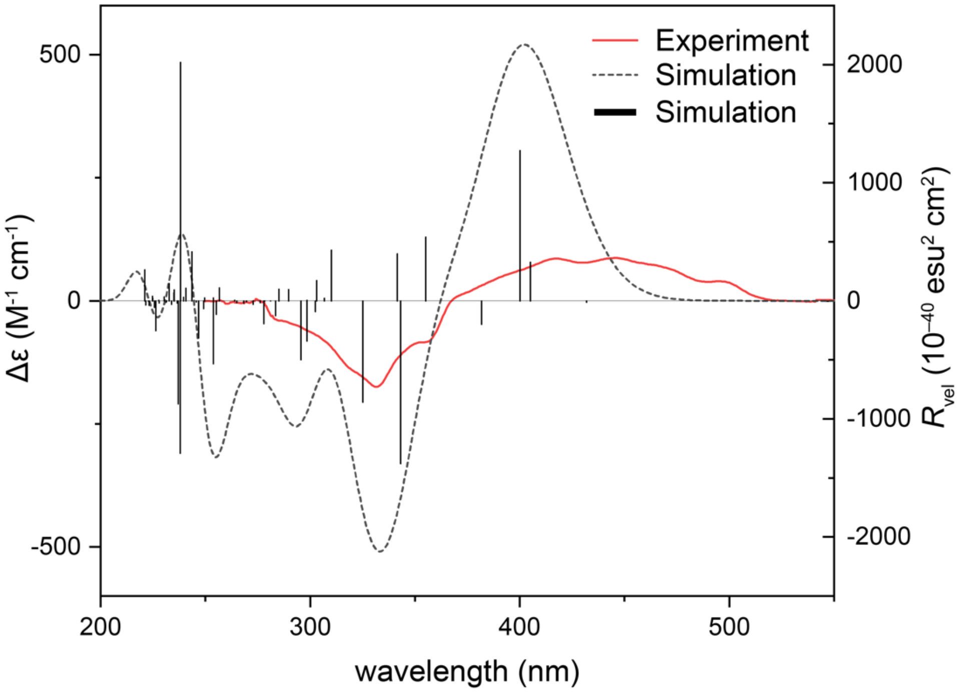

Figure S13. Comparison of the CD spectrum of (*P,P*)-**HC** (red line) in toluene with the spectrum simulated by TD-DFT calculations (black dashed line and black vertical lines; ωB97XD/Def2SVP(toluene)).

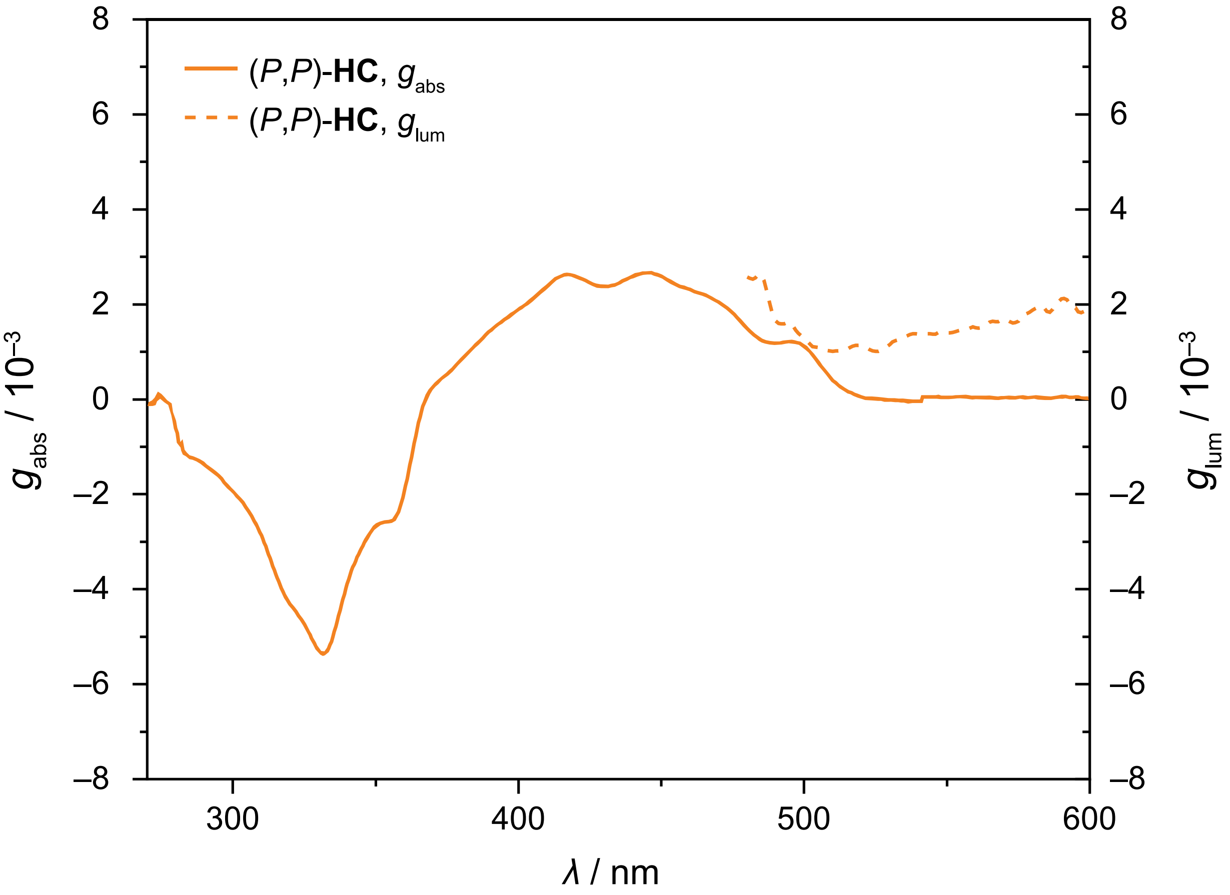

Figure S14. Absorption dissymmetry factor (g_{abs}) and luminescence dissymmetry factor (g_{lum}) of (P,P)-**HC**.

S6. CV and DPV

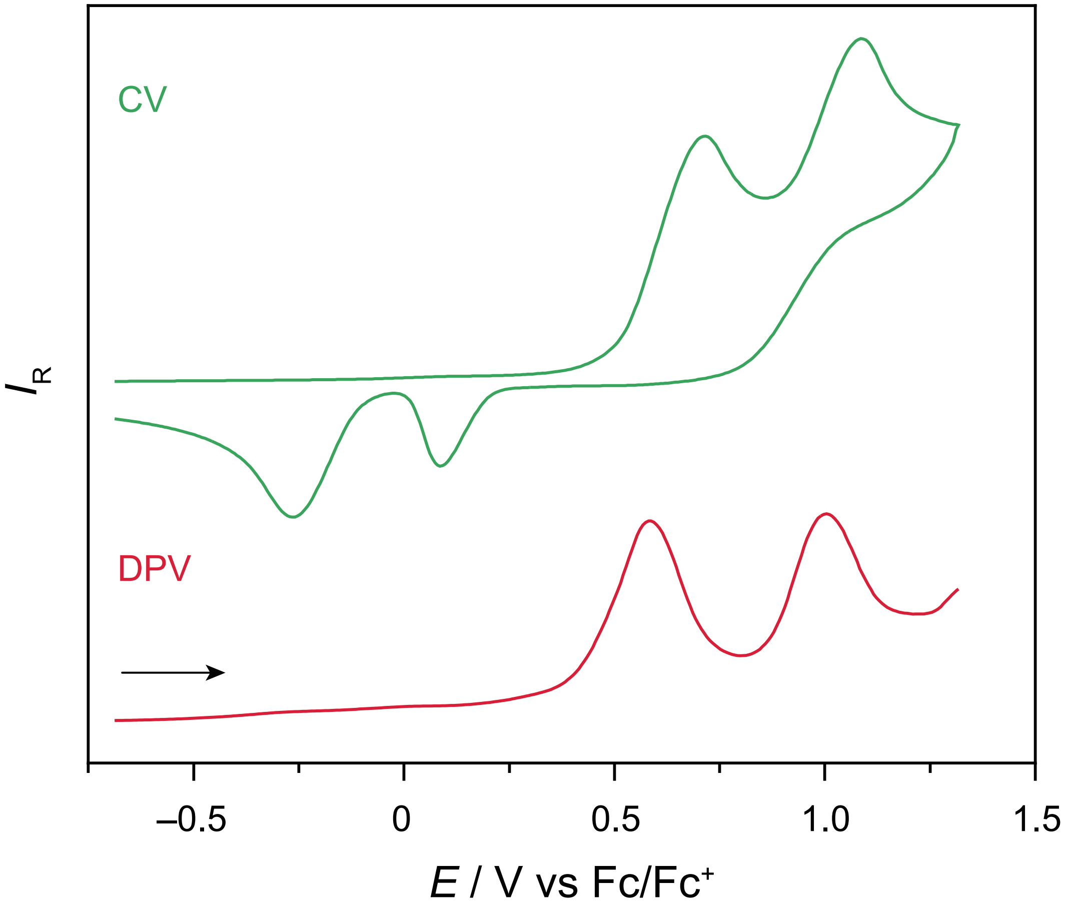

Figure S15. CV (green) and DPV (red) voltammograms versus Fc/Fc^+ of 2*H*-**NC** in CH_2Cl_2 with 0.1 M $[Bu_4N][PF_6]$ as a supporting electrolyte at a potential sweep rate of 100 mV/s. Note: With lower sweep rates, the reduction bands start to disappear. With 0.8 V as the maximum potential, only one oxidation and one reduction band are observed. The area of the DPV peaks is the same.

S7. X-ray crystallography

(*P*)-6:

Single-crystal X-ray diffraction data were collected at 160(1) K on a Rigaku OD XtaLAB Synergy, Dualflex, Pilatus 200K diffractometer using a single-wavelength X-ray source (Cu K_α radiation: λ = 1.54184 Å) from a micro-focus sealed X-ray tube and an Oxford liquid-nitrogen Cryostream cooler. The selected suitable single crystal was mounted using polybutene oil on a flexible loop fixed on a goniometer head and immediately transferred to the diffractometer. Pre-experiment, data collection, data reduction, and analytical absorption correction[1] were performed with the program suite *CrysAlisPro*.[2] Using *Olex2*,[3] the structure was solved with the *SHELXT*[4] small-molecule structure solution program and refined with the *SHELXL2018/3* program package[5] by full-matrix least-squares minimization on F^2. *PLATON*[6] was used to check the result of the X-ray analysis. For more details about the data collection and refinement parameters, see the CIF file.

Special features

There are two independent molecules in the asymmetric unit.

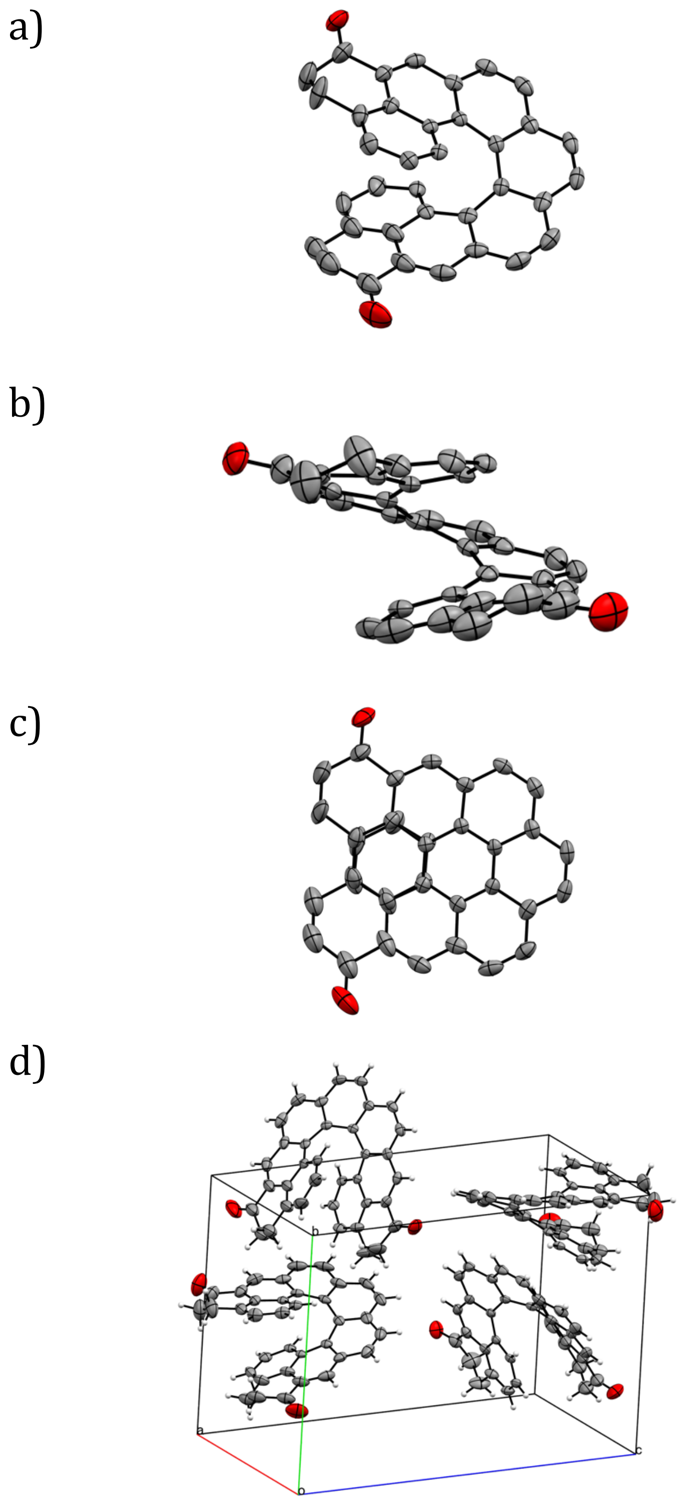

Figure S16. Crystallographic views of the solid-state structure of (*P*)-**6**: (a) perspective, (b) side, (c) top, (d) packing. All hydrogen atoms (a–c) are omitted for clarity. Thermal ellipsoids are shown at the 50% probability level. Color code: C/ gray, O / red.

(*P*)-2*H*-NC:

Single-crystal X-ray diffraction data were collected at 160(1) K on a Rigaku OD XtaLAB Synergy, Dualflex, Pilatus 200K diffractometer using a single-wavelength X-ray source (Cu K_α radiation: λ = 1.54184 Å) from a micro-focus sealed X-ray tube and an Oxford liquid-nitrogen Cryostream cooler. The selected suitable single crystal was mounted using polybutene oil on a flexible loop fixed on a goniometer head and immediately transferred to the diffractometer. Pre-experiment, data collection, data reduction, and analytical absorption correction[1] were performed with the program suite *CrysAlisPro*.[2] Using *Olex2*,[3] the structure was solved with the *SHELXT*[4] small-molecule structure solution program and refined with the *SHELXL2018/3* program package[5] by full-matrix least-squares minimization on F^2. *PLATON*[6] was used to check the result of the X-ray analysis. For more details about the data collection and refinement parameters, see the CIF file.

Special features

The absolute structure parameter is meaningless because the compound is a weak anomalous scatterer.

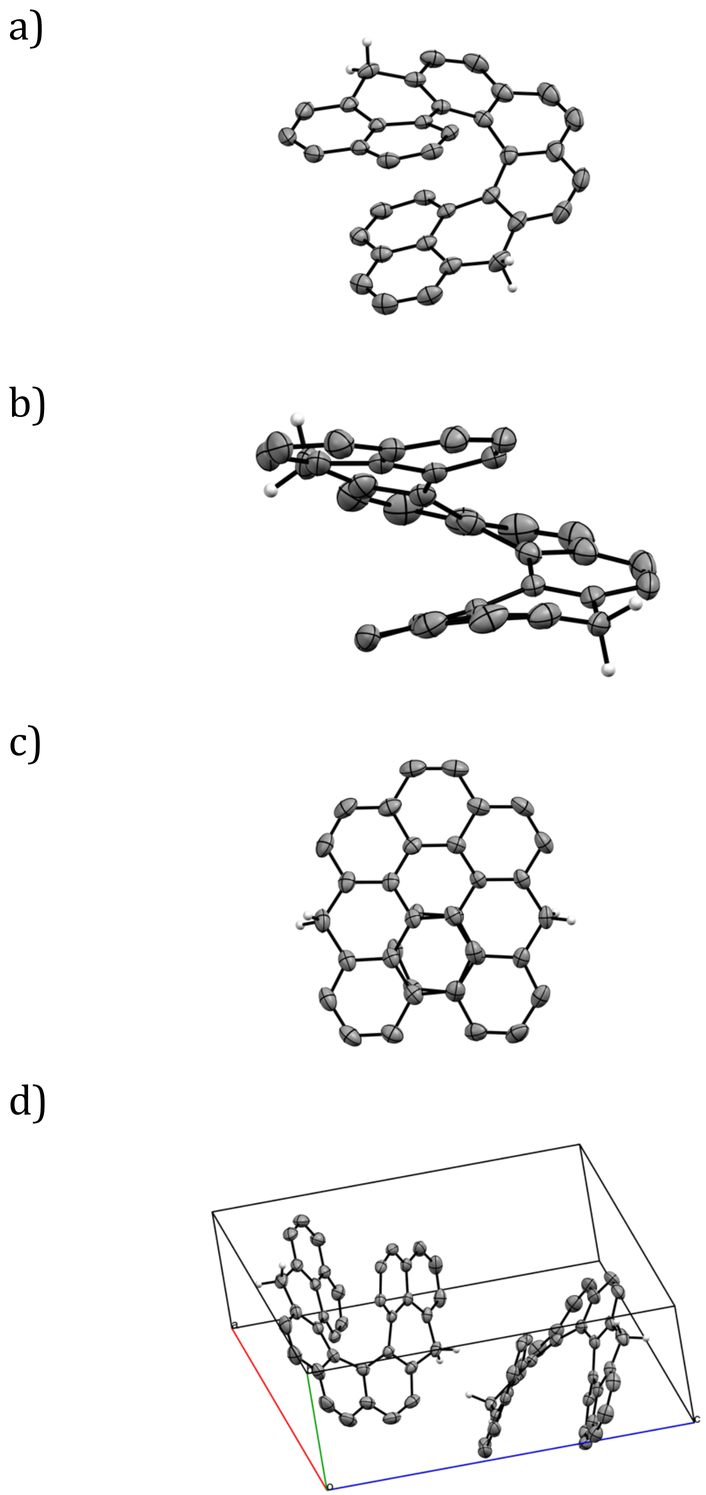

Figure S17. Crystallographic views of the solid-state structure of (*P*)-2*H*-**NC**:
(a) perspective, (b) side, (c) top, (d) packing. Some hydrogen atoms are omitted for clarity. Thermal ellipsoids are shown at the 50% probability level. Color code: C/ gray, H / white.

***O*-c-NC:**

Single-crystal X-ray diffraction data were collected at 160(1) K on a Rigaku OD XtaLAB Synergy, Dualflex, Pilatus 200K diffractometer using a single-wavelength X-ray source (Mo K_α radiation: λ = 0.71073 Å)[7] from a micro-focus sealed X-ray tube and an Oxford liquid-nitrogen Cryostream cooler. The selected suitable single crystal was mounted using polybutene oil on a flexible loop fixed on a goniometer head and immediately transferred to the diffractometer. Pre-experiment, data collection, data reduction, and analytical absorption correction[1] were performed with the program suite *CrysAlisPro*.[2] Using *Olex2*,[3] the structure was solved with the *SHELXT*[4] small-molecule structure solution program and refined with the *SHELXL2016/6* program package[5] by full-matrix least-squares minimization on F^2. *PLATON*[8] was used to check the result of the X-ray analysis. For more details about the data collection and refinement parameters, see the CIF file.

Refinement

No special features.

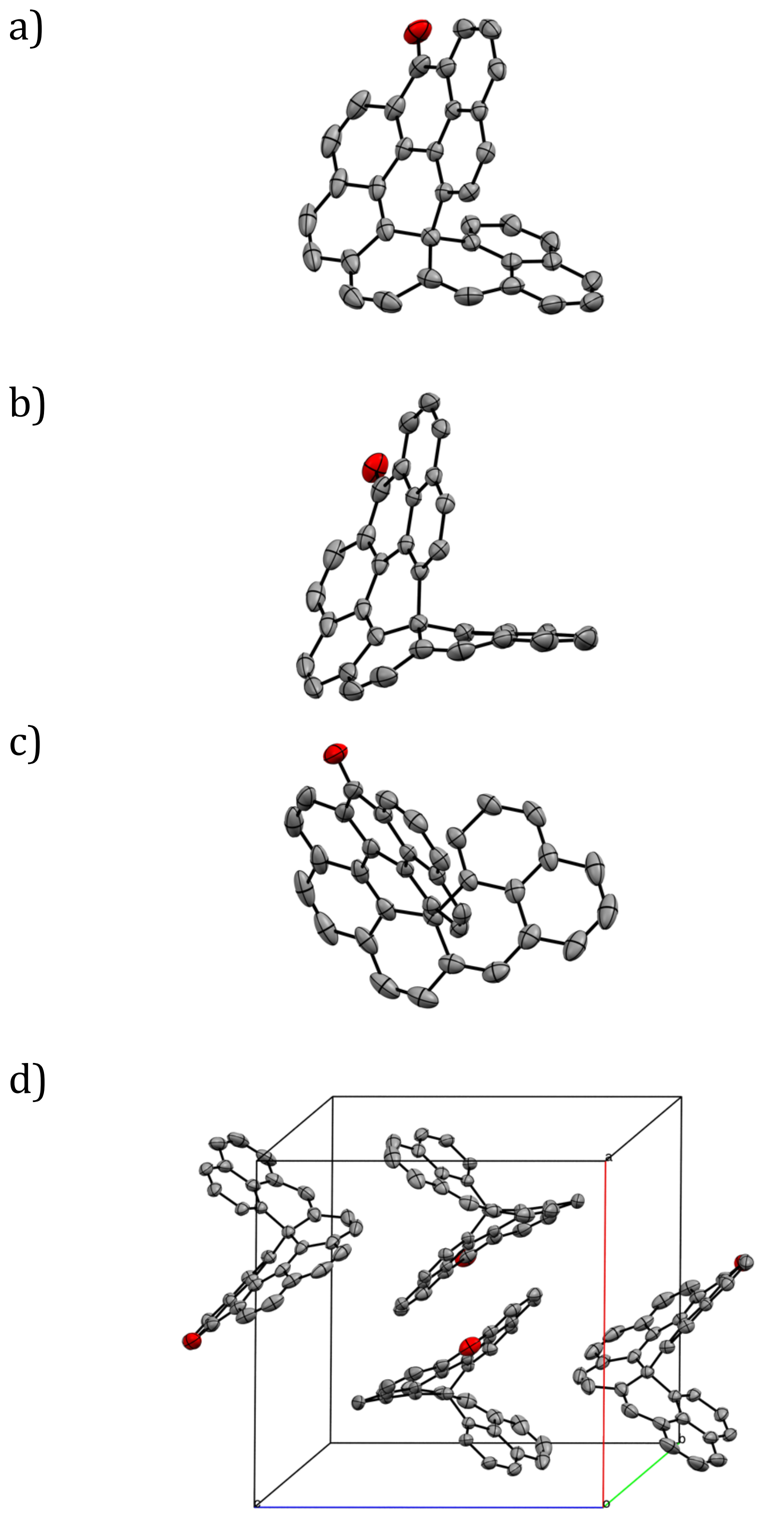

Figure S18. Crystallographic views of the solid-state structure of *O*-c-**NC**: (a) perspective, (b) side, (c) top, (d) packing. All hydrogen atoms are omitted for clarity. Thermal ellipsoids are shown at the 50% probability level. Color code: C/ gray, O / red.

sym-(*P*,*S*,*R*,*M*)-(c-NC)$_2$:

Single-crystal X-ray diffraction data were collected at 160(1) K on a Rigaku OD SuperNova/ Atlas area-detector diffractometer using Cu K_α radiation (λ = 1.54184 Å) from a micro-focus X-ray source and an Oxford Instruments Cryojet XL cooler. The selected suitable single crystal was mounted using polybutene oil on a flexible loop fixed on a goniometer head and immediately transferred to the diffractometer. Pre-experiment, data collection, data reduction, and analytical absorption correction[1] were performed with the program suite *CrysAlisPro*.[2] Using *Olex2*,[3] the structure was solved with the *SHELXT*[4] small-molecule structure solution program and refined with the *SHELXL2018/3* program package[5] by full-matrix least-squares minimization on F^2. *PLATON*[8] was used to check the result of the X-ray analysis. For more details about the data collection and refinement parameters, see the CIF file.

Special features

A solvent mask[5] was calculated as the residual electron density showed disordered solvent molecules of benzene in channels. The calculated total of 63 electrons was attributed to 1.5 solvent molecule per asymmetric unit. This was taken into account in the formula moiety and the formula sum (leading to alerts in the checkCIF report).

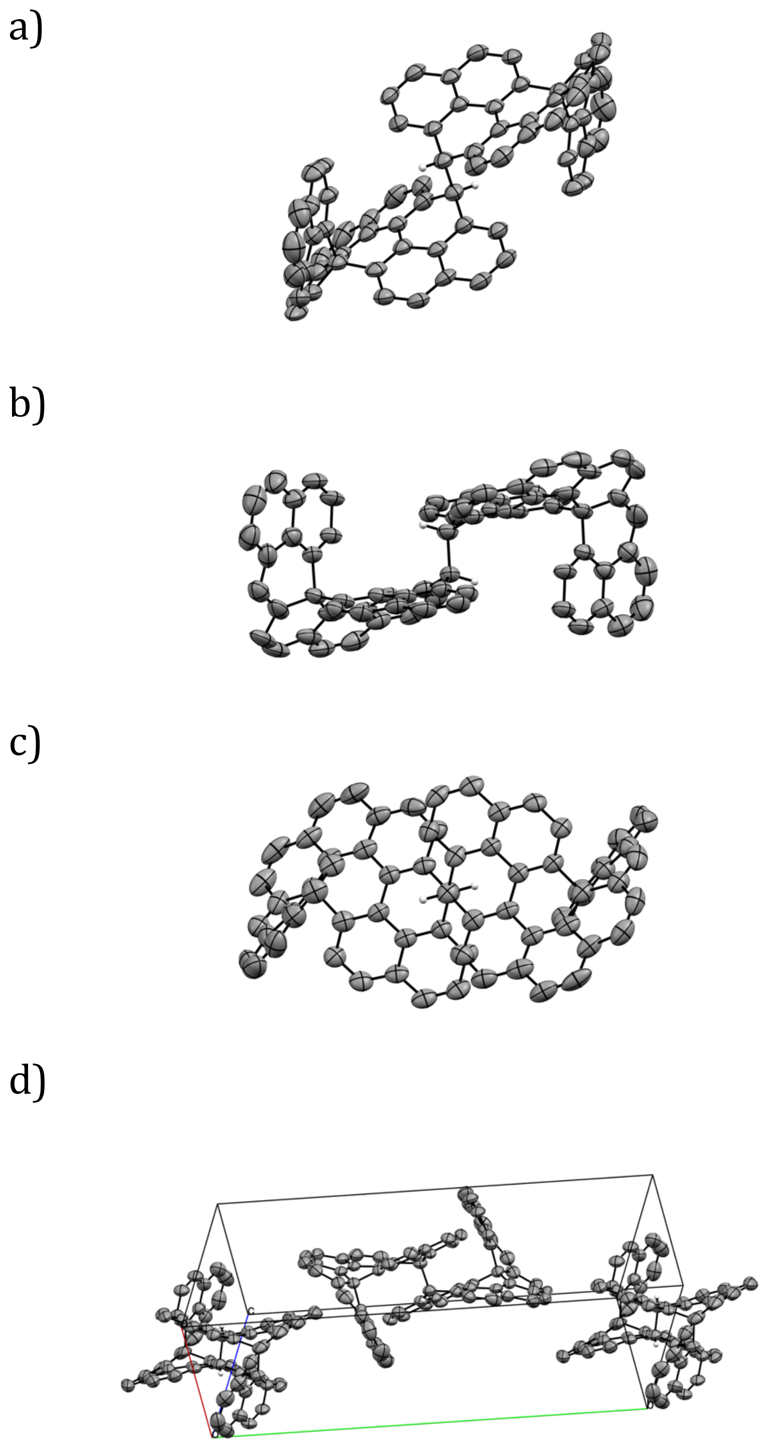

Figure S19. Crystallographic views of the solid-state structure of sym-(P,S,R,M)-(c-**NC**)$_2$: (a) perspective, (b) side, (c) top, (d) packing. Some hydrogen atoms are omitted for clarity. Thermal ellipsoids are shown at the 50% probability level. Color code: C/ gray, H / white.

Table S1. Crystal data and structure refinement for (*P*)-6 & (*P*)-2*H*-NC.

Identification code	(*P*)-**6**	(*P*)-2*H*-**NC**
Empirical formula	$C_{36}H_{22}O_2$	$C_{36}H_{22}$
Formula weight	486.53	454.53
Temperature / K	160(1)	160(1)
Crystal system	monoclinic	monoclinic
Space group	$P2_1$	$P2_1$
a / Å	9.25580(10)	10.07160(10)
b / Å	14.07710(10)	7.59150(10)
c / Å	18.8124(2)	15.64690(10)
α / °	90	90
β / °	98.9140(10)	105.0330(10)
γ / °	90	90
Volume / Å^3	2421.55(4)	1155.40(2)
Z	4	2
ρ_{calc} / g cm^{-3}	1.335	1.307
μ / mm^{-1}	0.637	0.563
F(000)	1016.0	476.0
Crystal size / mm^3	0.19 × 0.1 × 0.03	0.17 × 0.1 × 0.04
Radiation	Cu Kα (λ = 1.54184)	Cu Kα (λ = 1.54184)
2Θ range for data collection / °	4.754 to 148.968	5.848 to 148.988
Index ranges	$-10 \le h \le 11$, $-17 \le k \le 17$, $-22 \le l \le 23$	$-9 \le h \le 12$, $-9 \le k \le 9$, $-19 \le l \le 19$
Reflections collected	41713	24248
Independent reflections	9606 [R_{int} = 0.0336, R_{sigma} = 0.0304]	4721 [R_{int} = 0.0184, R_{sigma} = 0.0145]
Data/restraints/parameters	9606/217/685	4721/1/325
Goodness-of-fit on F^2	1.021	1.036
Final R indexes [$I \ge 2\sigma(I)$]	R_1 = 0.0588, wR_2 = 0.1598	R_1 = 0.0270, wR_2 = 0.0751
Final R indexes [all data]	R_1 = 0.0633, wR_2 = 0.1662	R_1 = 0.0275, wR_2 = 0.0755
Largest diff. peak / hole / e Å^{-3}	0.66/−0.51	0.15/−0.15
Flack parameter	0.01(12)	−0.4(3)

Table S2. Crystal data and structure refinement for *O*-c-NC and sym-(*P*,*S*,*R*,*M*)-(c-NC)$_2$.

Identification code	*O*-c-**NC**	sym-(*P*,*S*,*R*,*M*)-(c-**NC**)$_2$
Empirical formula	$C_{36}H_{18}O$	$C_{81}H_{47}$
Formula weight	466.50	1020.18
Temperature / K	160(1)	160(1)
Crystal system	monoclinic	monoclinic
Space group	$P2_1/c$	$P2_1$
a / Å	15.2314(4)	8.3510(2)
b / Å	9.2604(4)	24.7558(7)
c / Å	15.6156(5)	12.5731(6)
α / °	90	90
β / °	96.722(3)	100.000(3)
γ / °	90	90
Volume / Å^3	2187.44(13)	2559.82(16)
Z	4	2
ρ_{calc} / g cm^{-3}	1.417	1.324
μ / mm^{-1}	0.084	0.571
F(000)	968.0	1066.0
Crystal size / mm^3	0.32 × 0.18 × 0.18	0.11 × 0.06 × 0.03
Radiation	MoKα (λ = 0.71073)	Cu Kα (λ = 1.54184)
2Θ range for data collection / °	5.124 to 61.016	7.14 to 148.97
Index ranges	$-21 \le h \le 20$, $-12 \le k \le 13$, $-22 \le l \le 21$	$-10 \le h \le 10$, $-24 \le k \le 30$, $-15 \le l \le 15$
Reflections collected	28175	26031
Independent reflections	6670 [R_{int} = 0.0308, R_{sigma} = 0.0281]	8522 [R_{int} = 0.0324, R_{sigma} = 0.0318]
Data/restraints/parameters	6670/0/334	8522/1/649
Goodness-of-fit on F^2	1.049	1.039
Final R indexes [$I \ge 2\sigma(I)$]	R_1 = 0.0541, wR_2 = 0.1446	R_1 = 0.0610, wR_2 = 0.1688
Final R indexes [all data]	R_1 = 0.0694, wR_2 = 0.1540	R_1 = 0.0791, wR_2 = 0.1853
Largest diff. peak / hole / e Å^{-3}	0.37/−0.18	0.25/−0.18
Flack parameter		0.6(6)

S8. Assignment of ^{1}H and ^{13}C NMR resonances

Figure S20. Compound **16**.

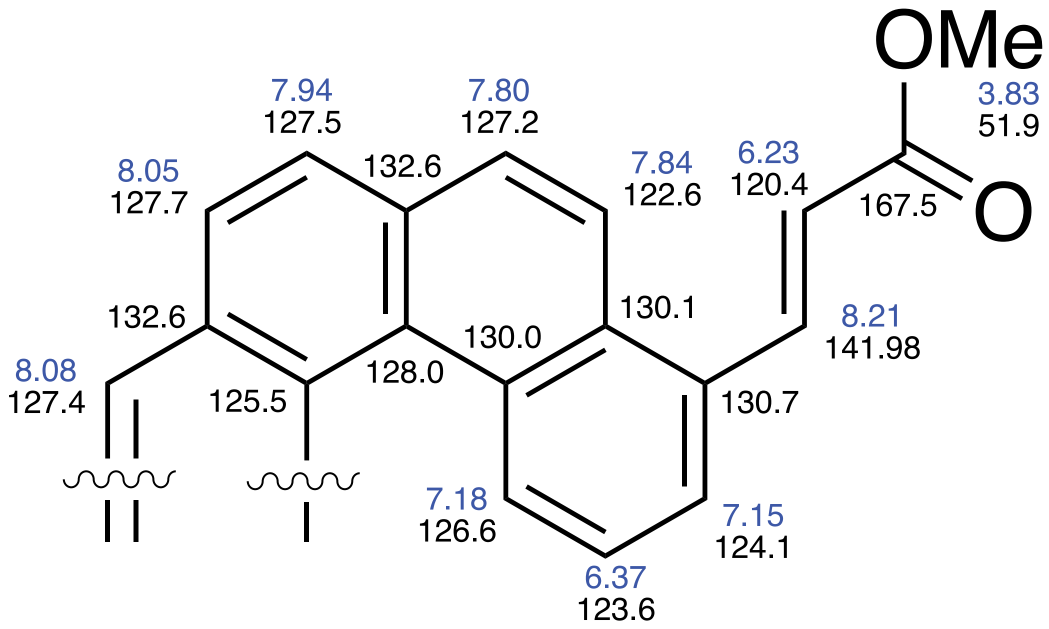

Figure S21. Compound 2*H*-**NC**.

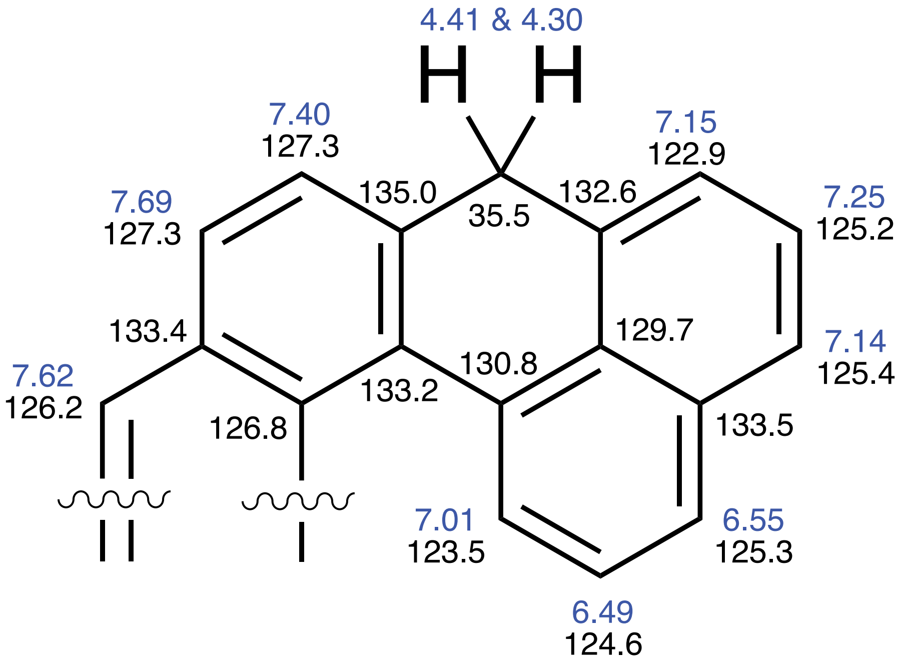

Figure S22. Compound c-**NC**.

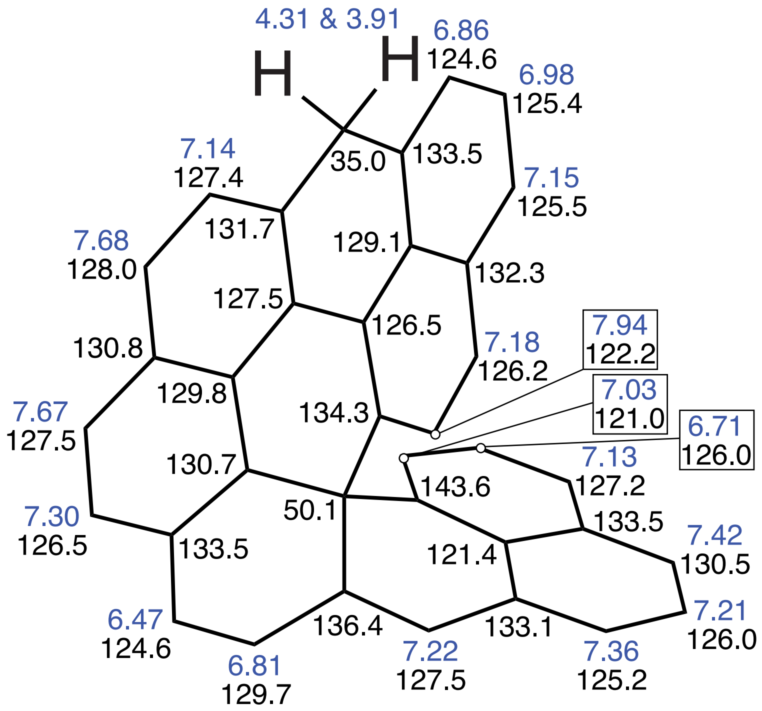

(π-bonds are omitted for clarity)

Figure S23. Compound *O*-c-**NC**.

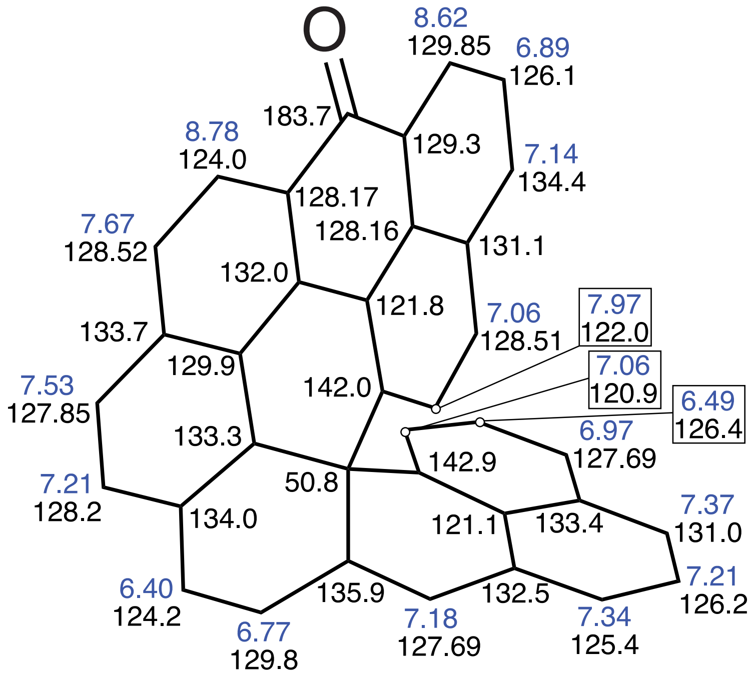

(π-bonds are omitted for clarity)

Figure S24. Compound sym-(*P*,*S*,*R*,*M*)-(c-**NC**)$_2$.

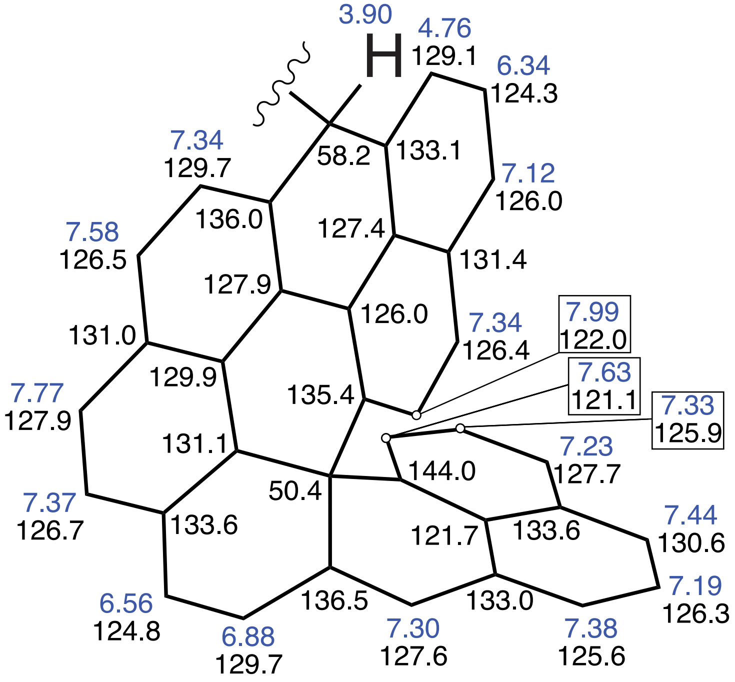

(π-bonds are omitted for clarity)

Figure S25. Compound sym-(*P**,*R**,*R**,*P**)-(c-**NC**)$_2$.

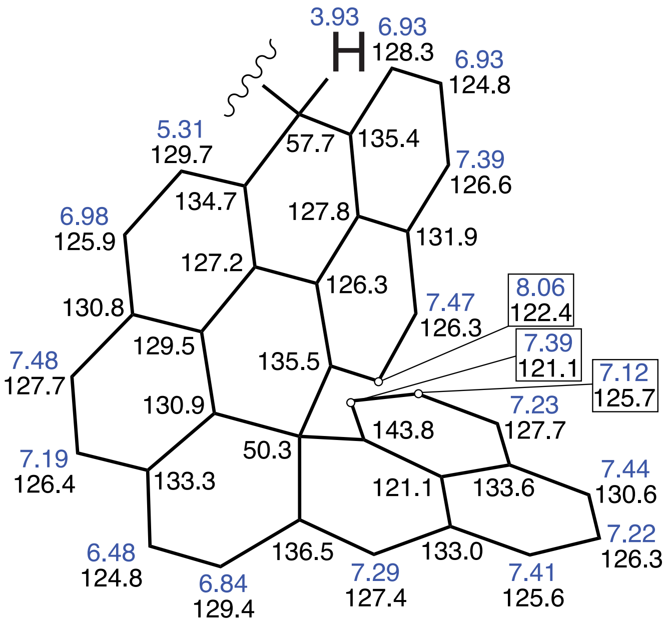

(π-bonds are omitted for clarity)

Figure S26. Hypercethrene (**HC**).

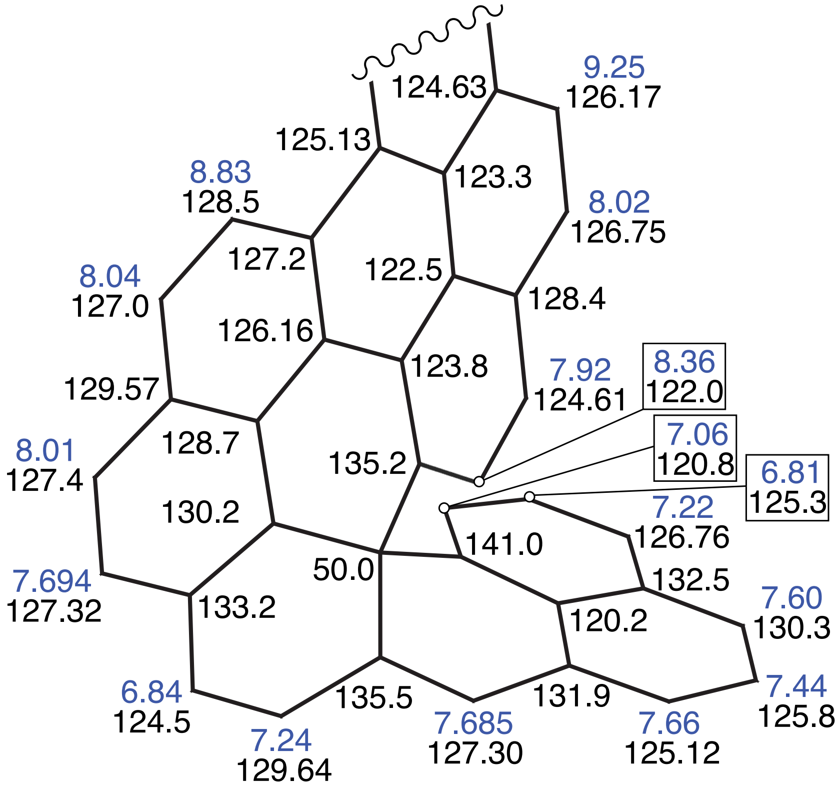

(π-bonds are omitted for clarity)

Figure S27. Compound 2*O*-**NC**.

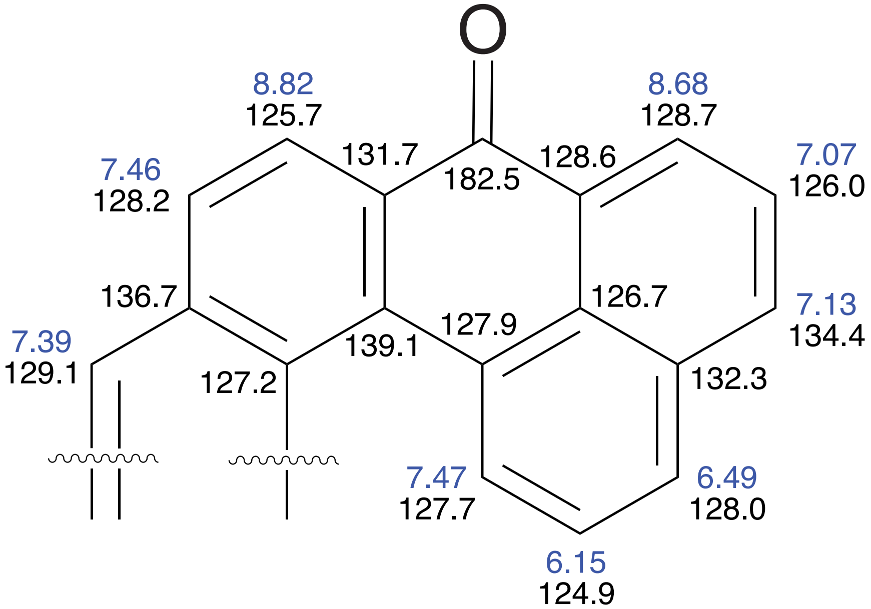

S9. Copies of NMR spectra

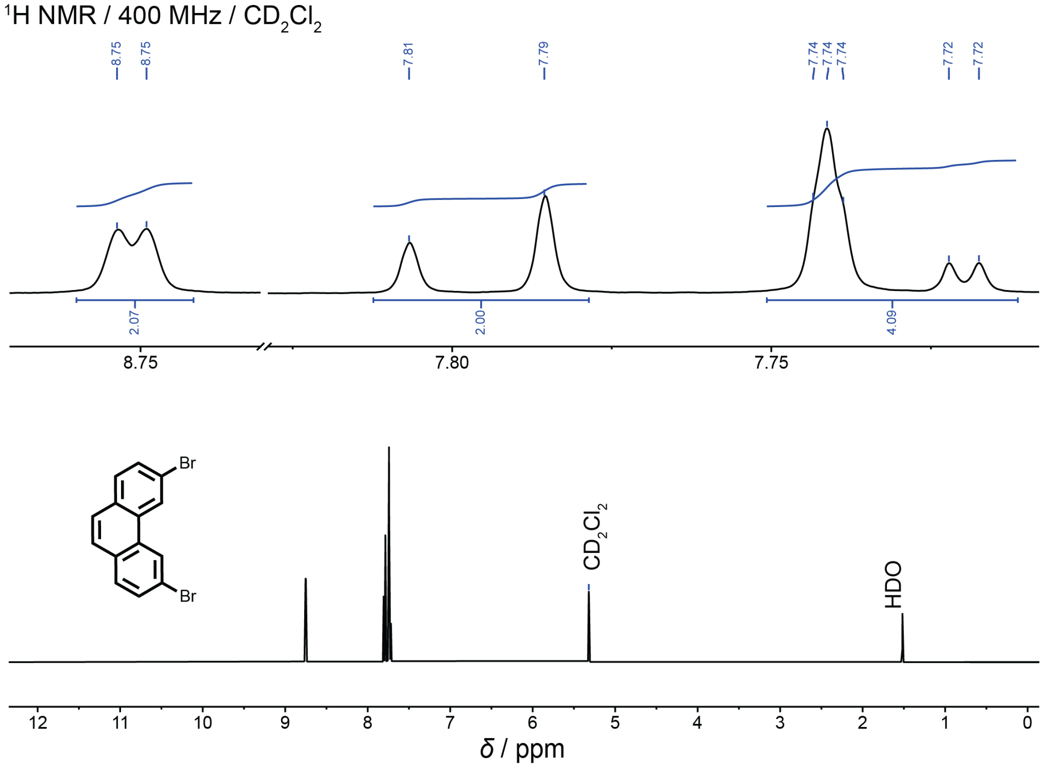

Figure S28. ^{1}H NMR spectrum of **2**.

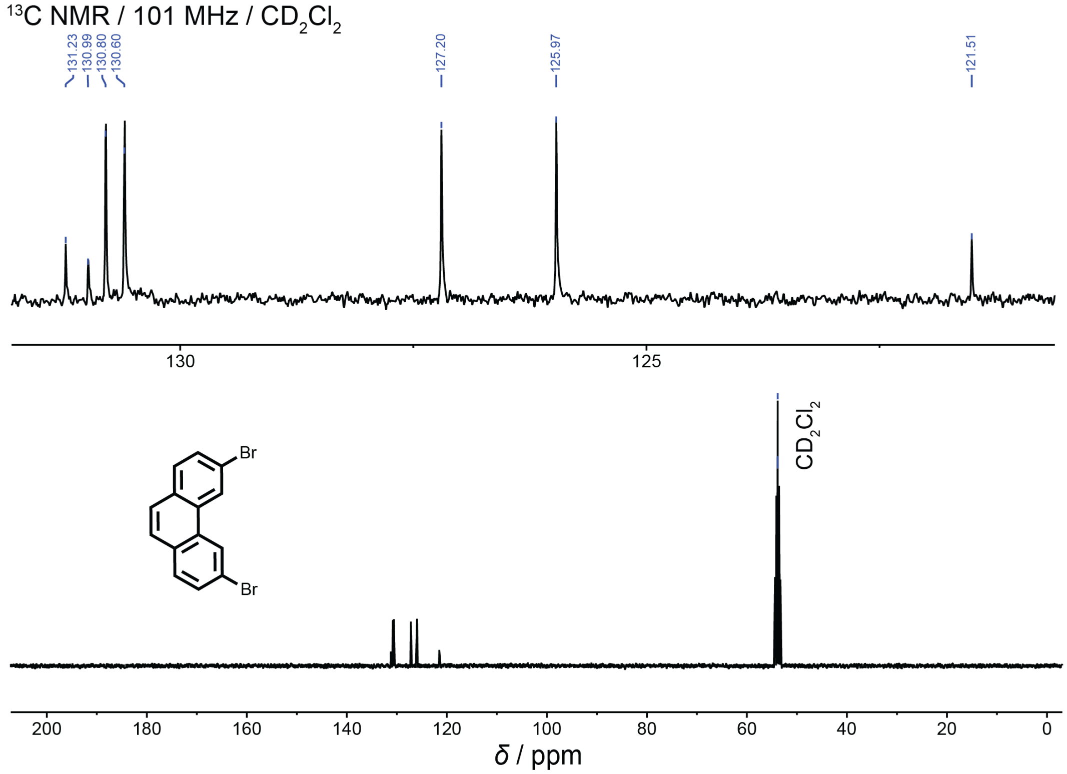

Figure S29. ^{13}C NMR spectrum of **2**.

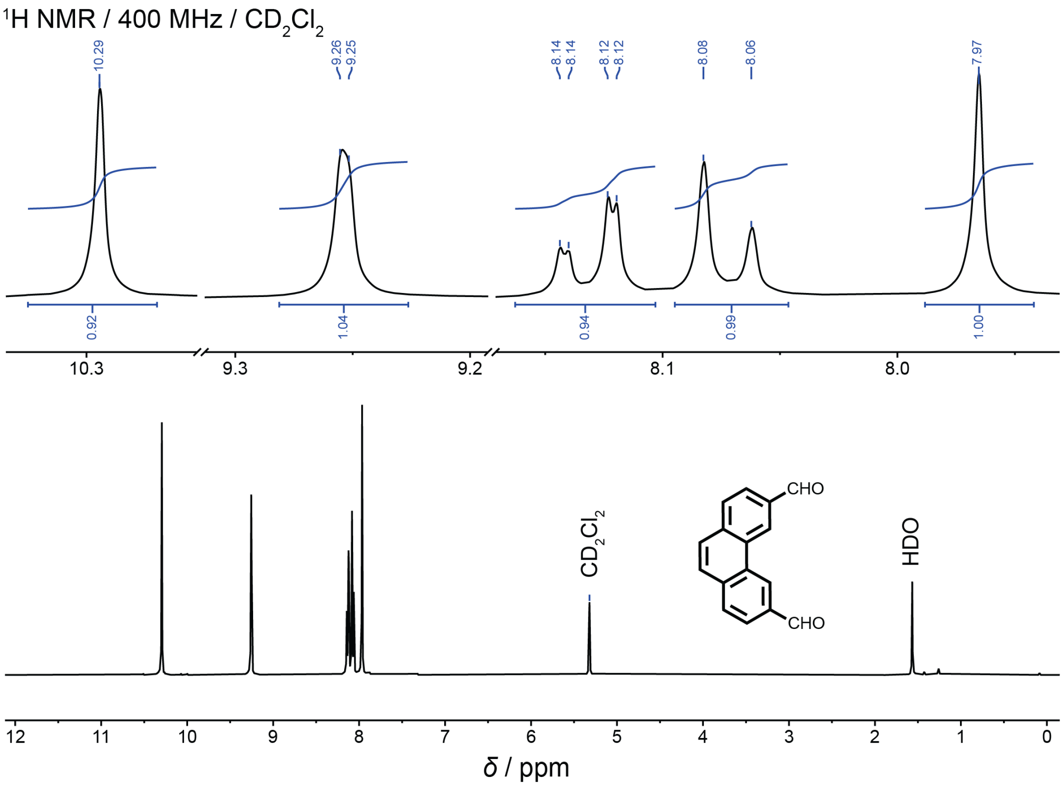

Figure S30. ^{1}H NMR spectrum of **3**.

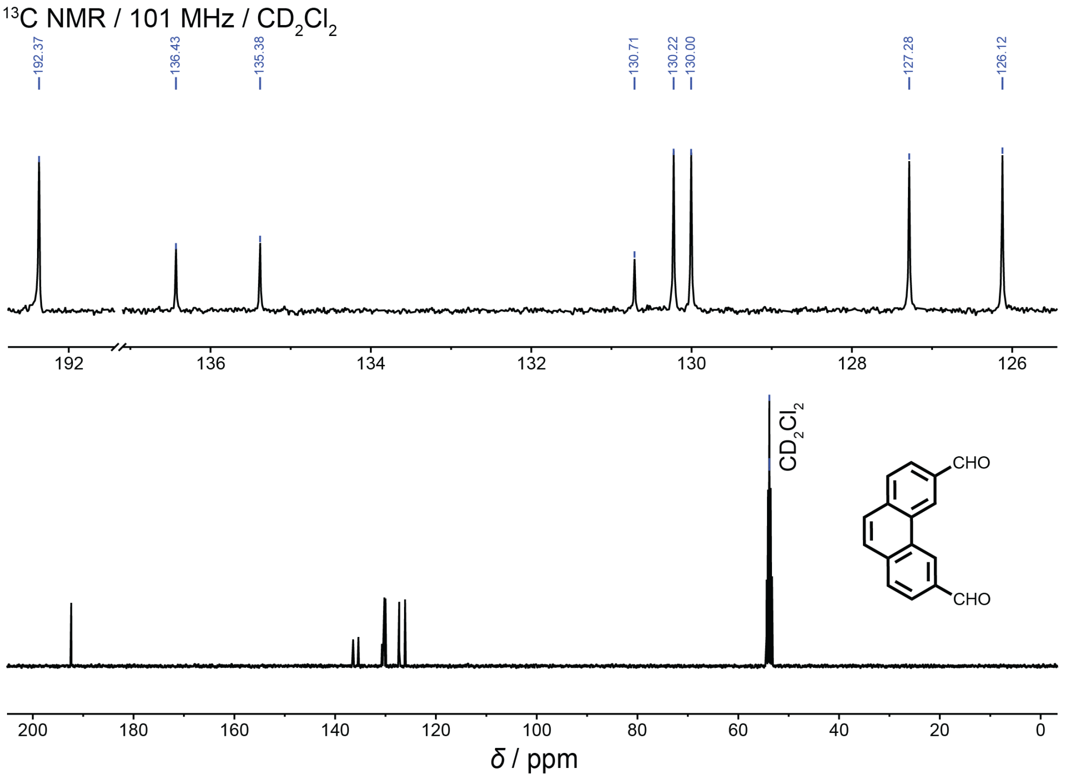

Figure S31. ^{13}C NMR spectrum of **3**.

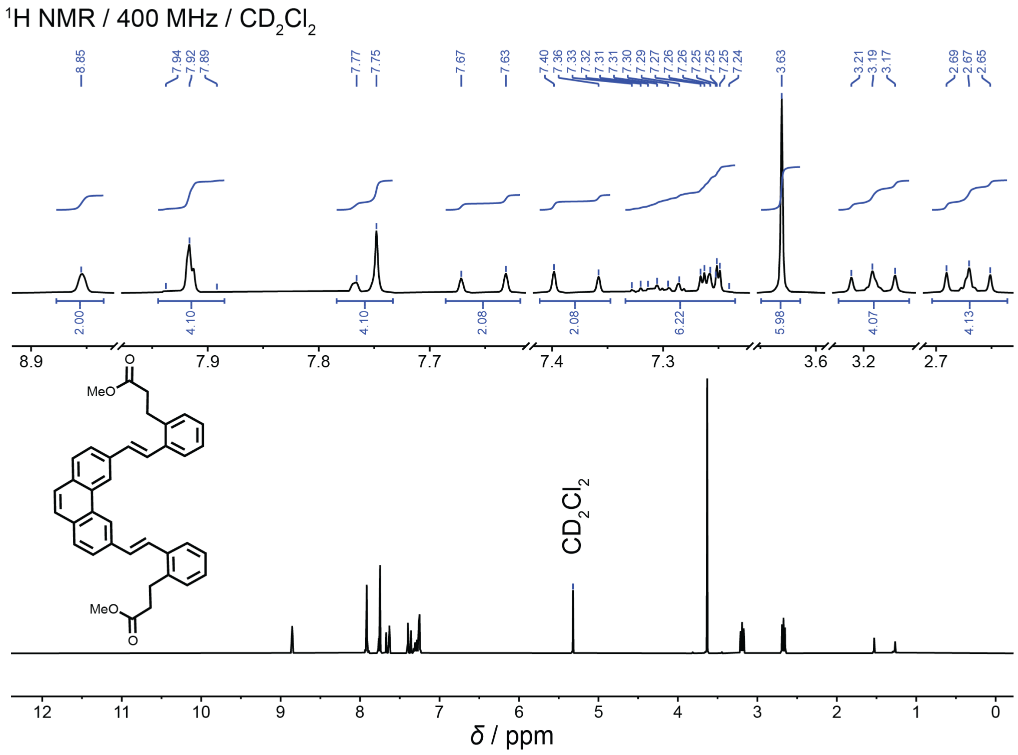

Figure S32. ^{1}H NMR spectrum of **4**.

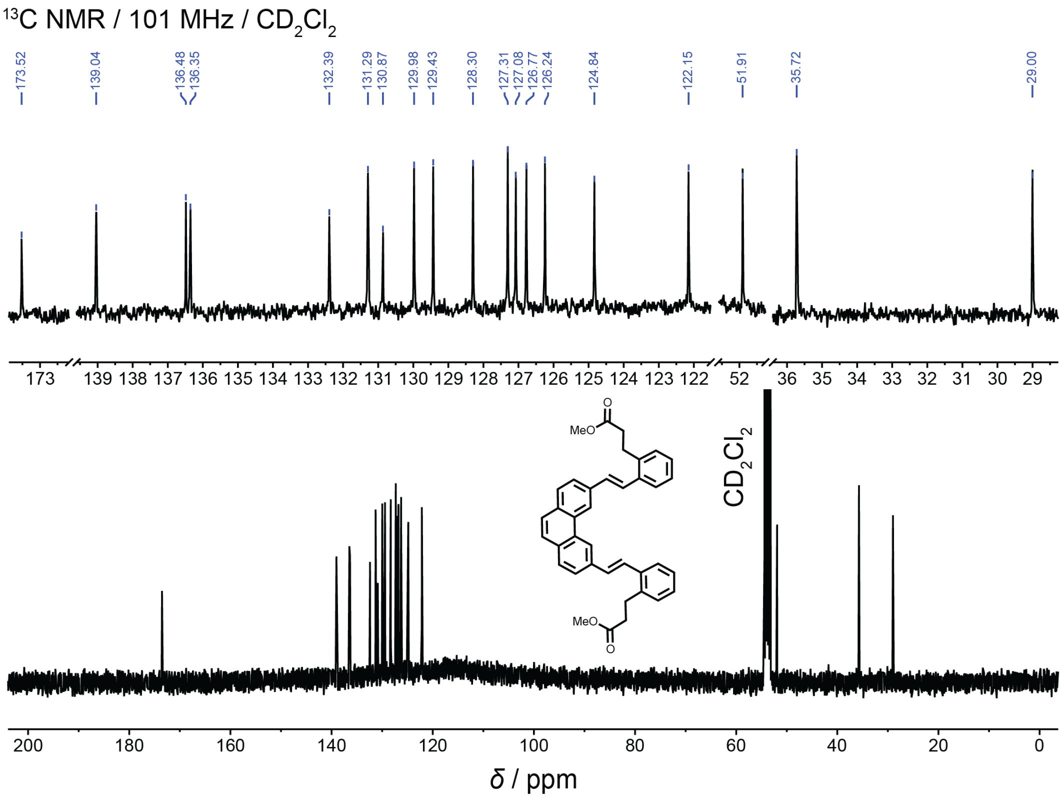

Figure S33. ^{13}C NMR spectrum of **4**.

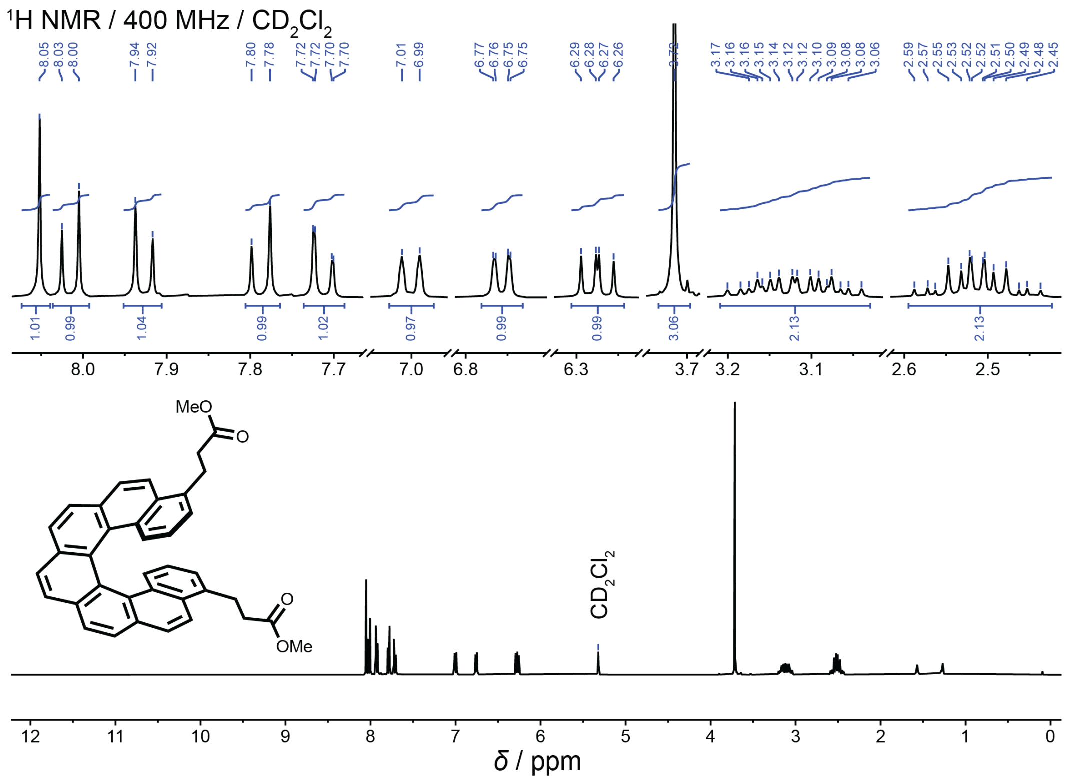

Figure S34. ^{1}H NMR spectrum of **5**.

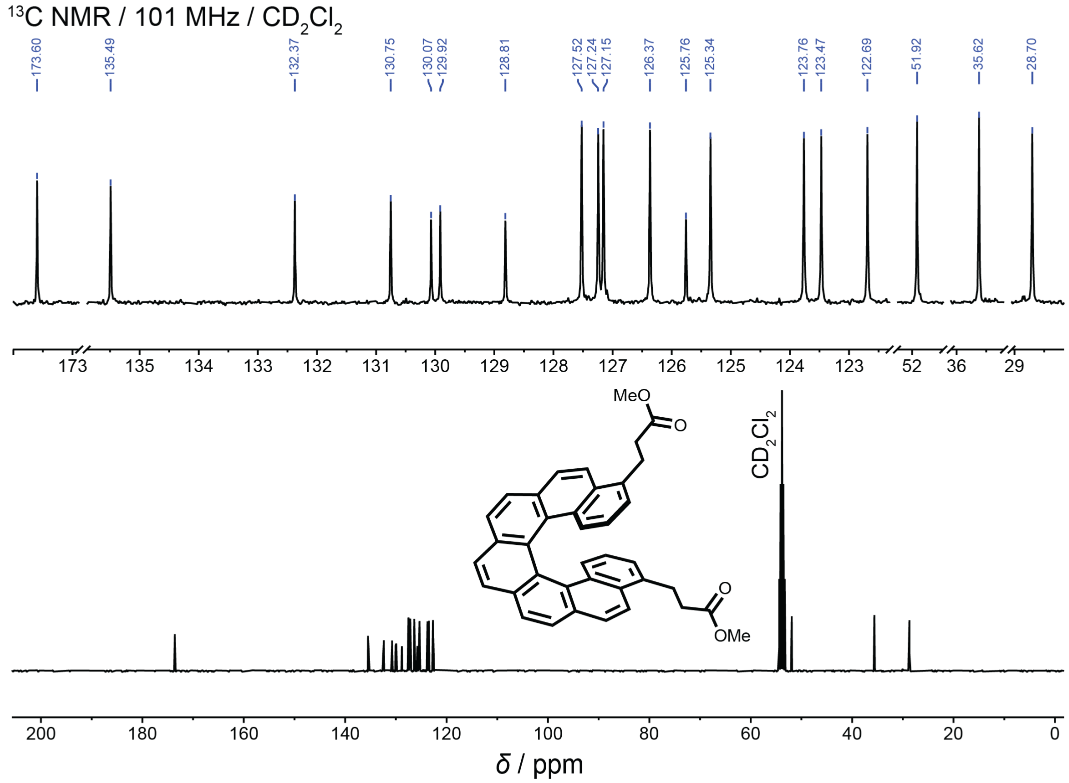

Figure S35. ^{13}C NMR spectrum of **5**.

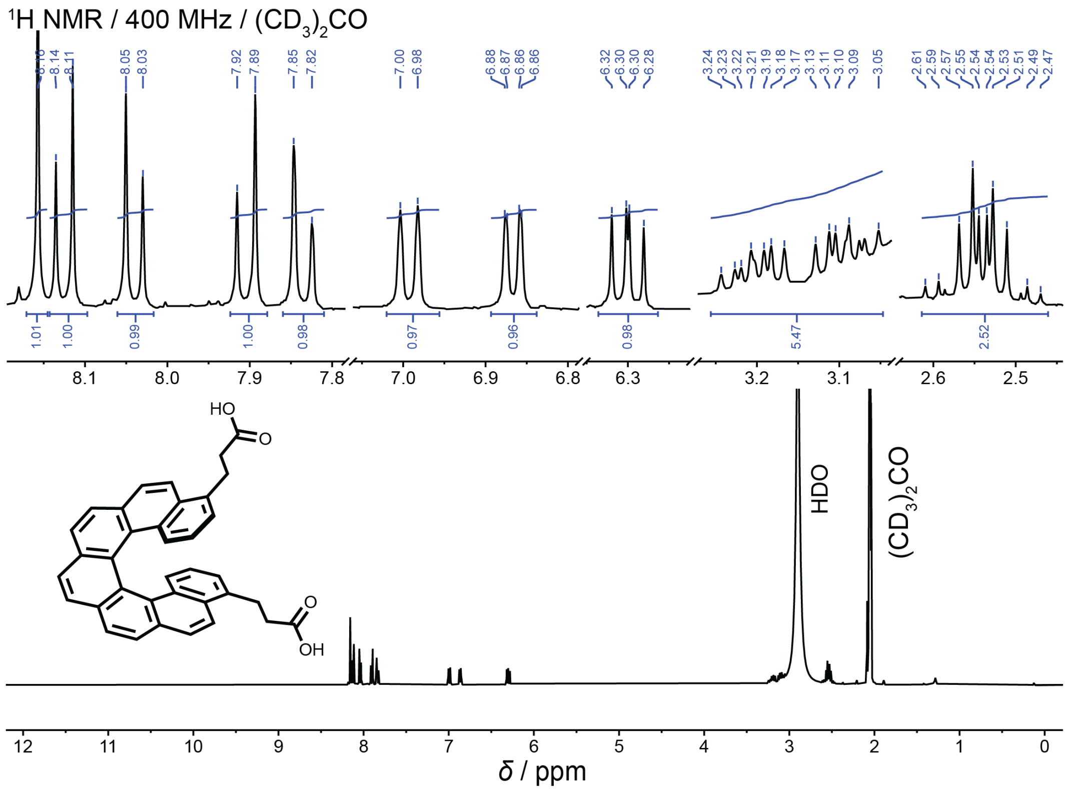

Figure S36. ^{1}H NMR spectrum of **12**.

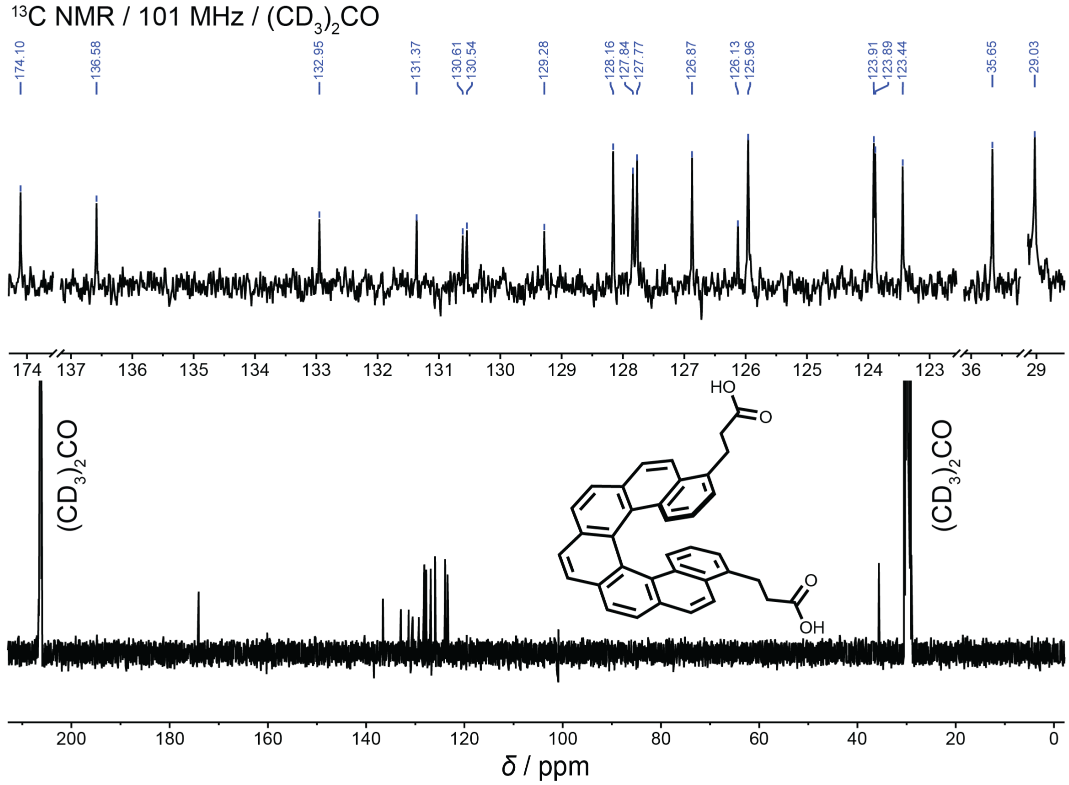

Figure S37. ^{13}C NMR spectrum of **12**.

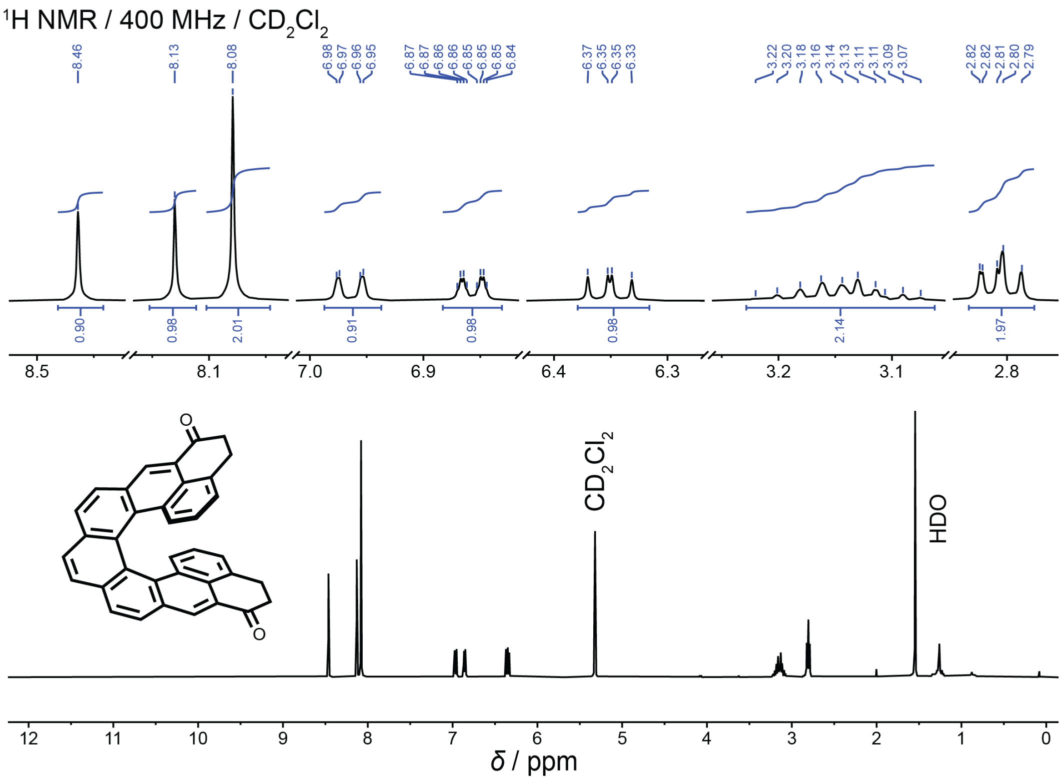

Figure S38. ^{1}H NMR spectrum of **6**.

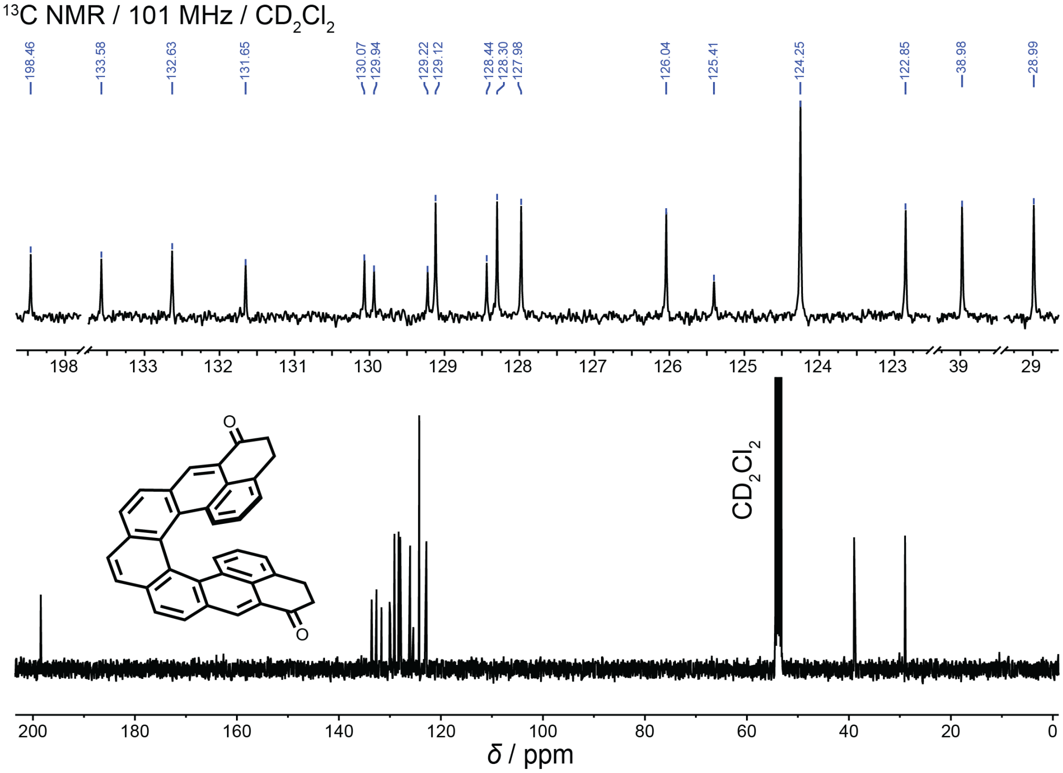

Figure S39. ^{13}C NMR spectrum of **6**.

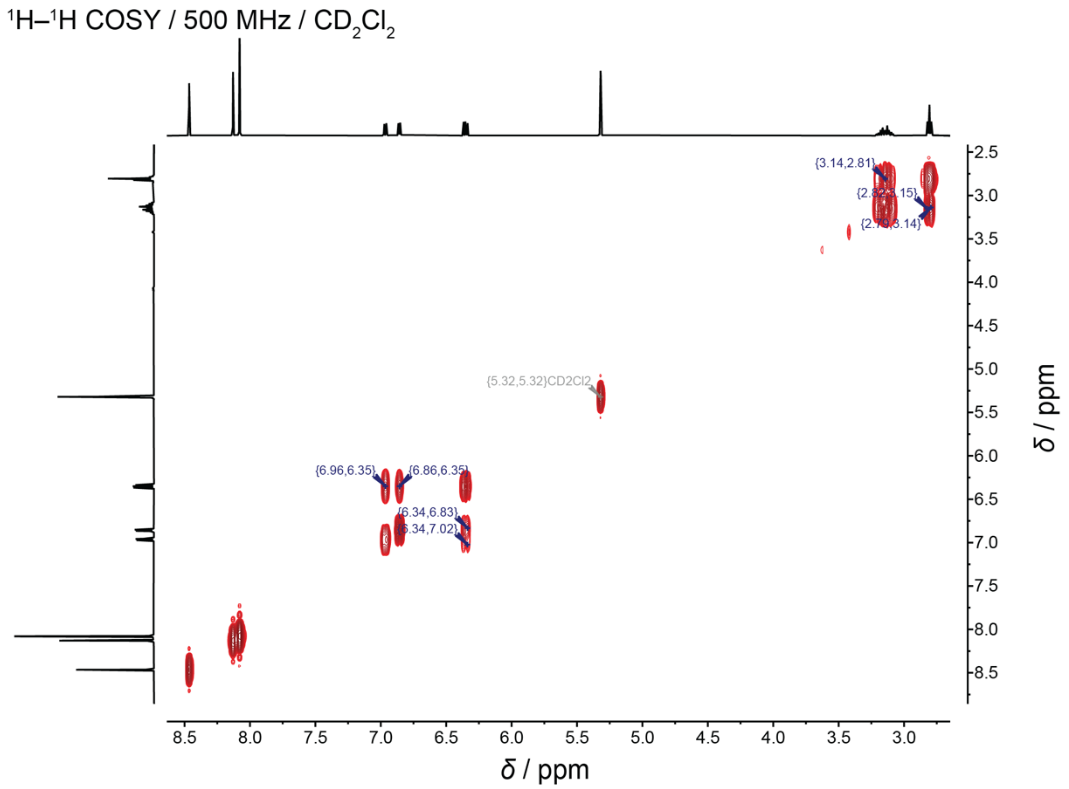

Figure S40. COSY NMR spectrum of **6**.

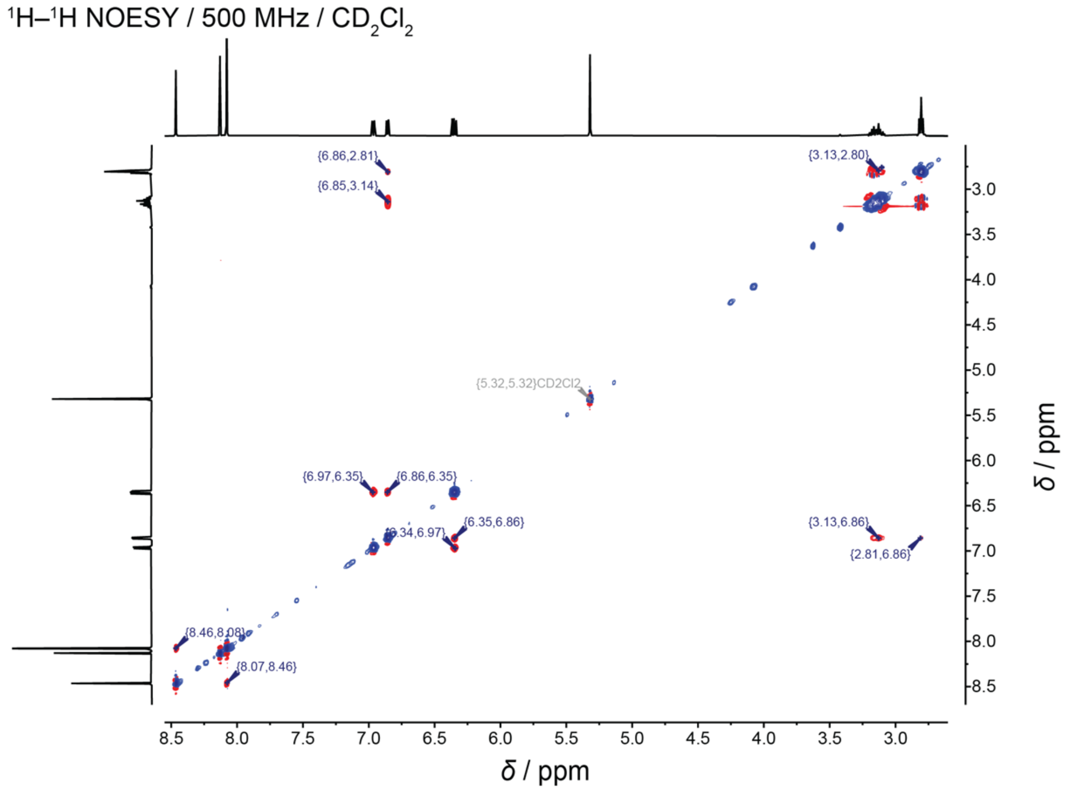

Figure S41. NOESY NMR spectrum of **6**.

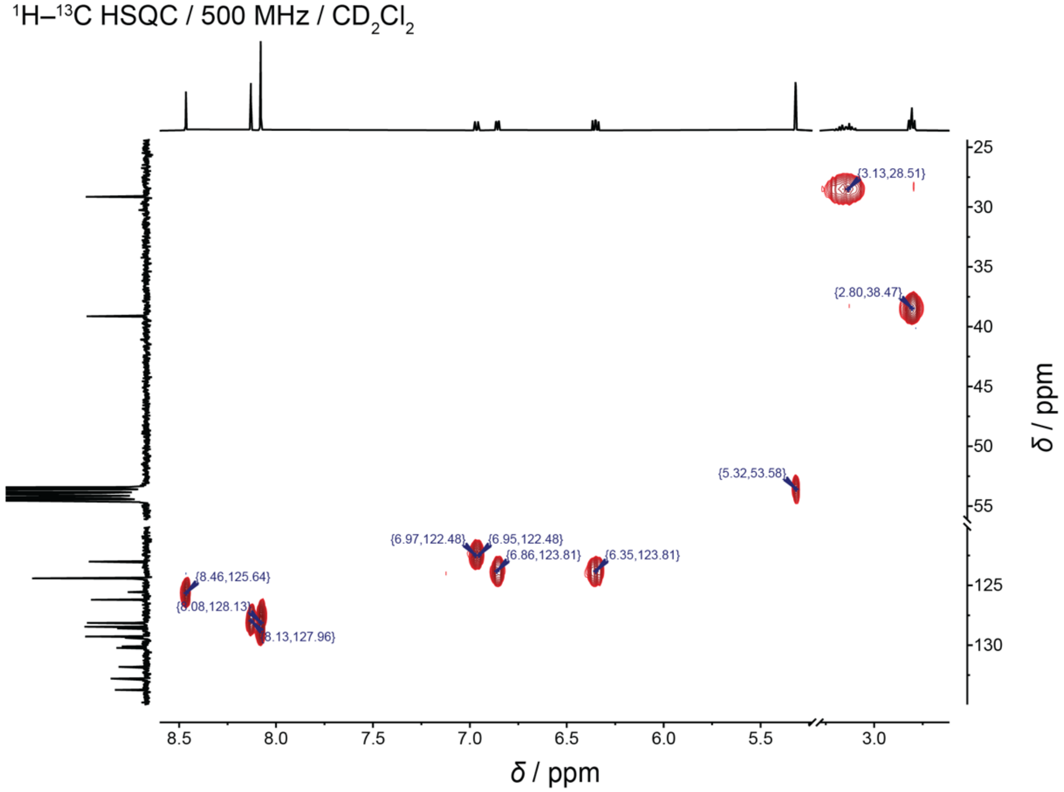

Figure S42. HSQC NMR spectrum of **6**.

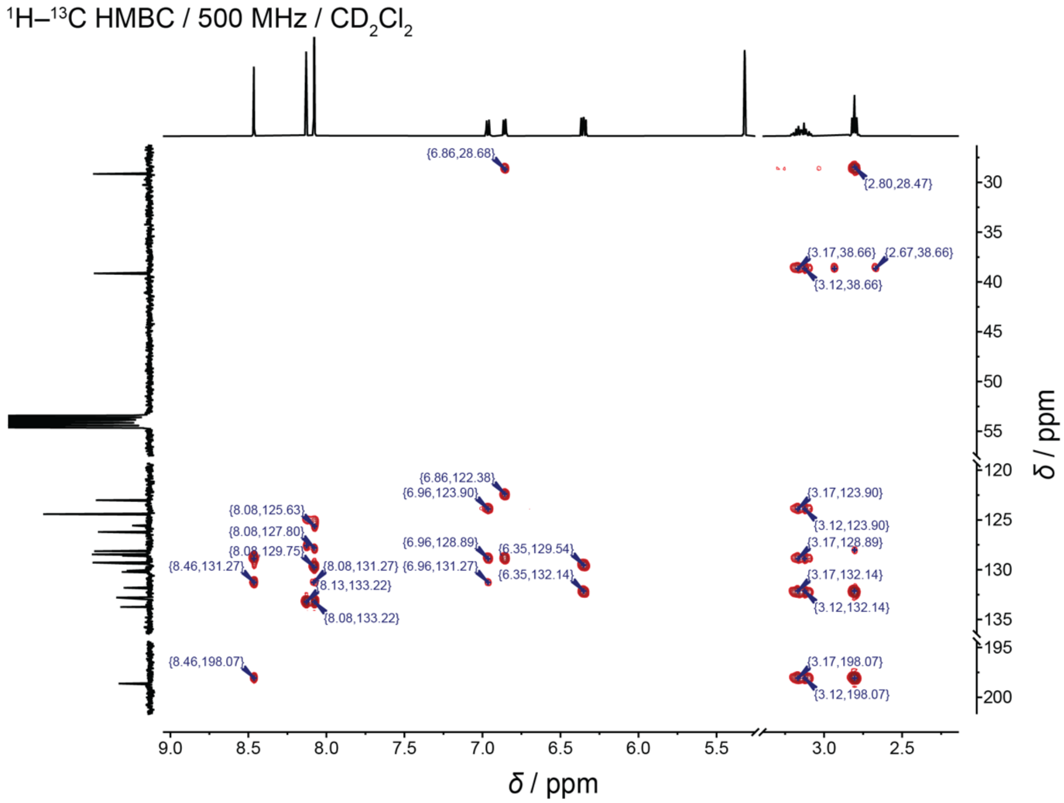

Figure S43. HMBC NMR spectrum of **6**.

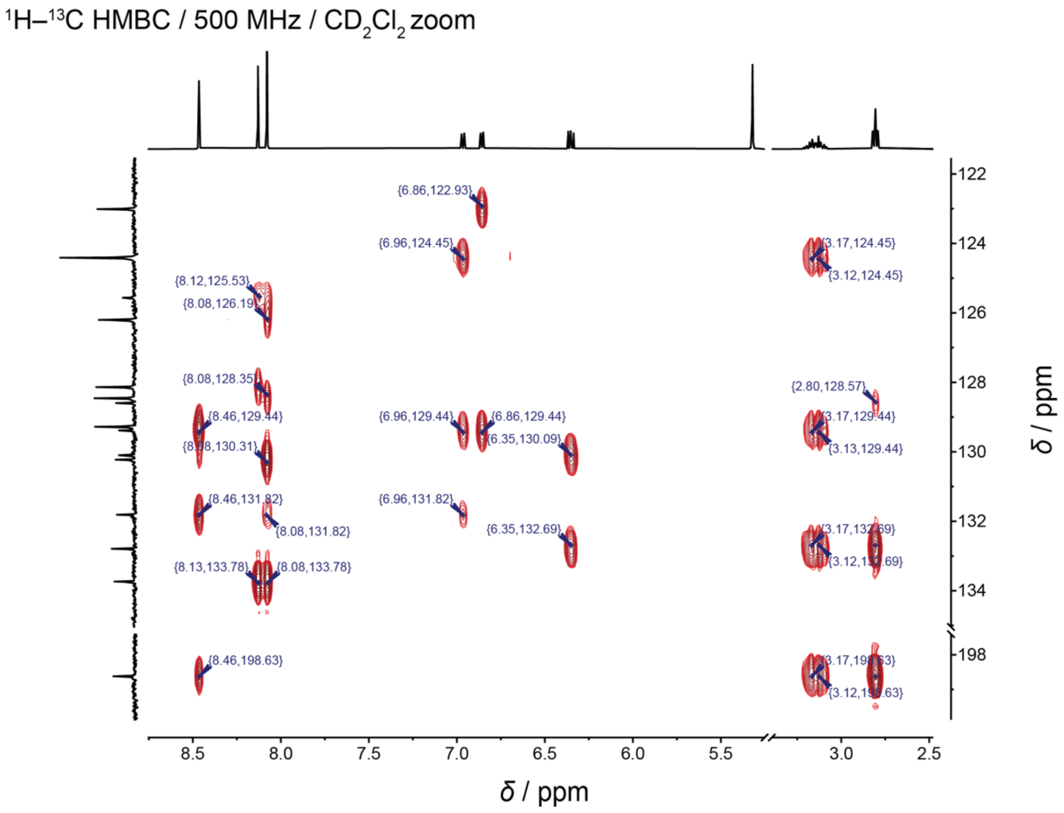

Figure S44. HMBC NMR spectrum of **6**.

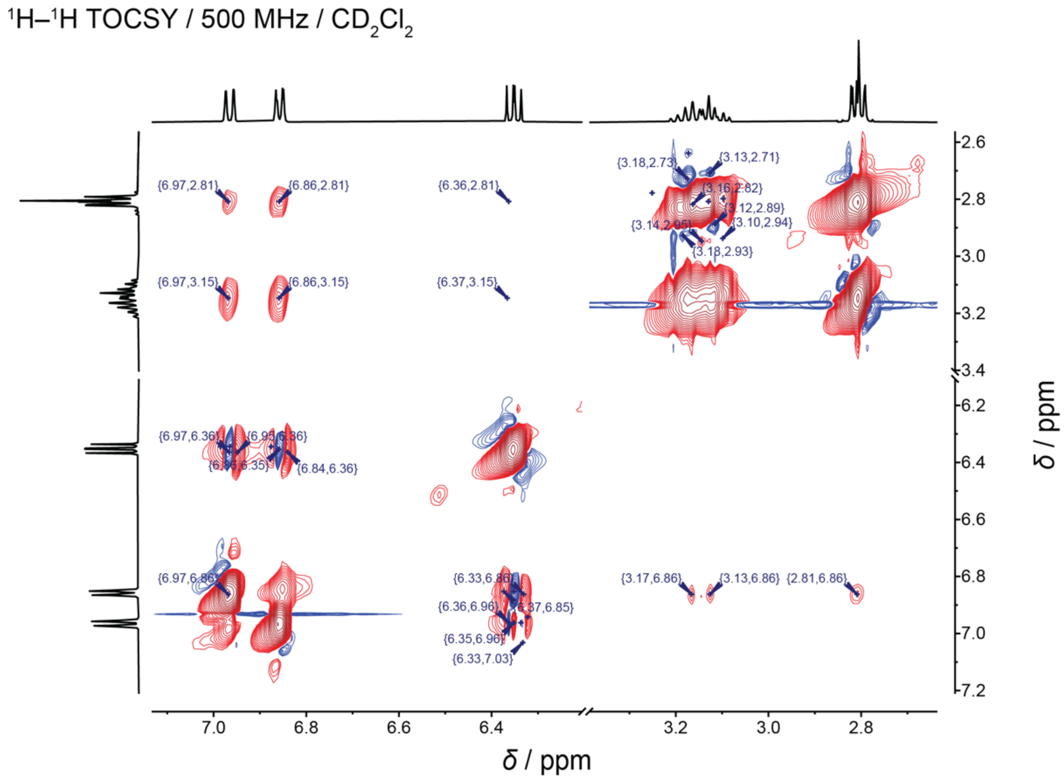

Figure S45. TOCSY NMR spectrum of **6**.

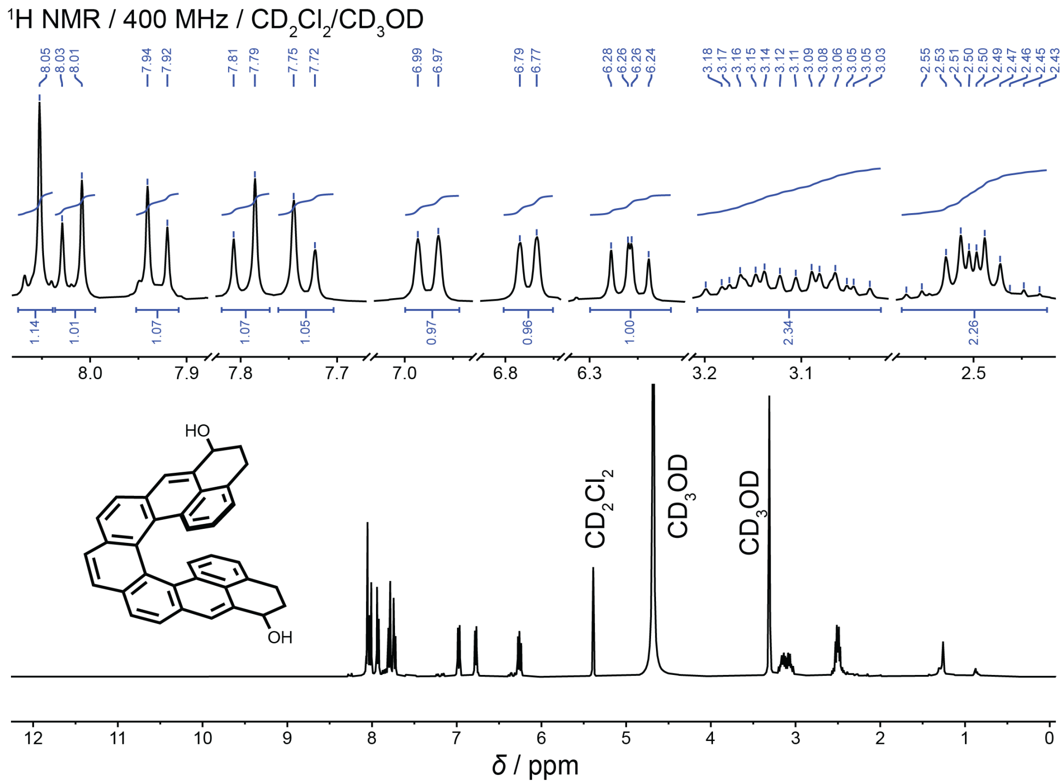

Figure S46. ^{1}H NMR spectrum of **7**.

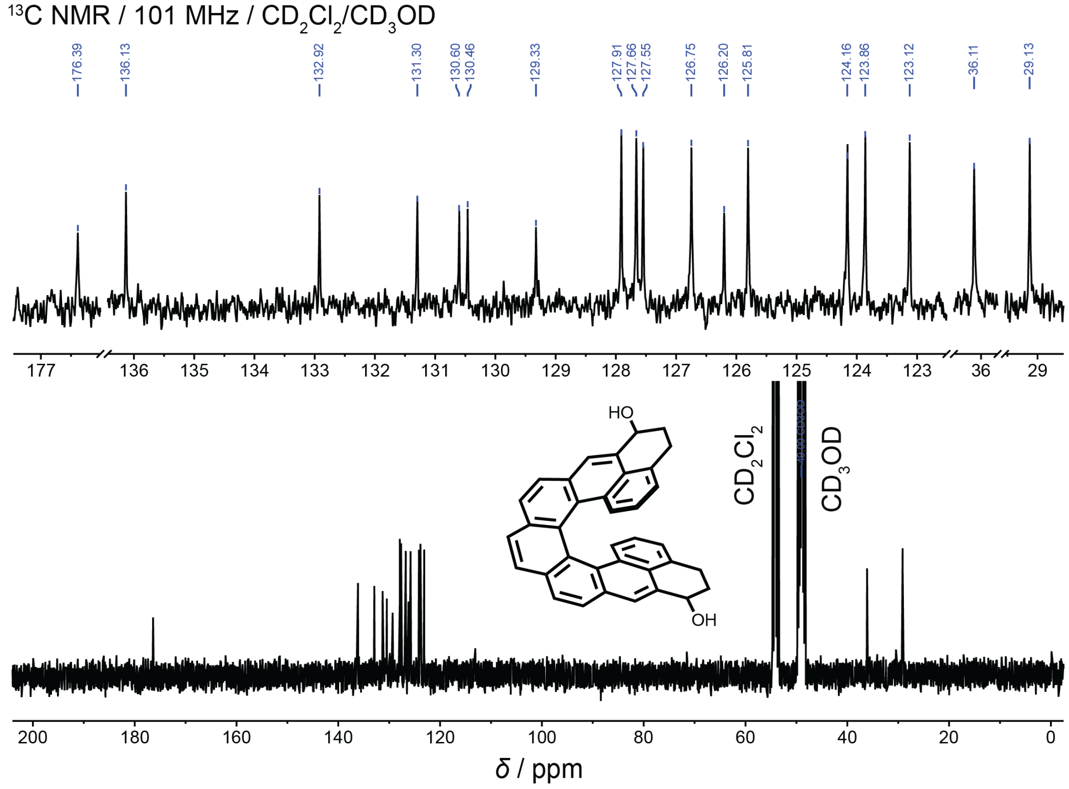

Figure S47. ^{13}C NMR spectrum of **7**.

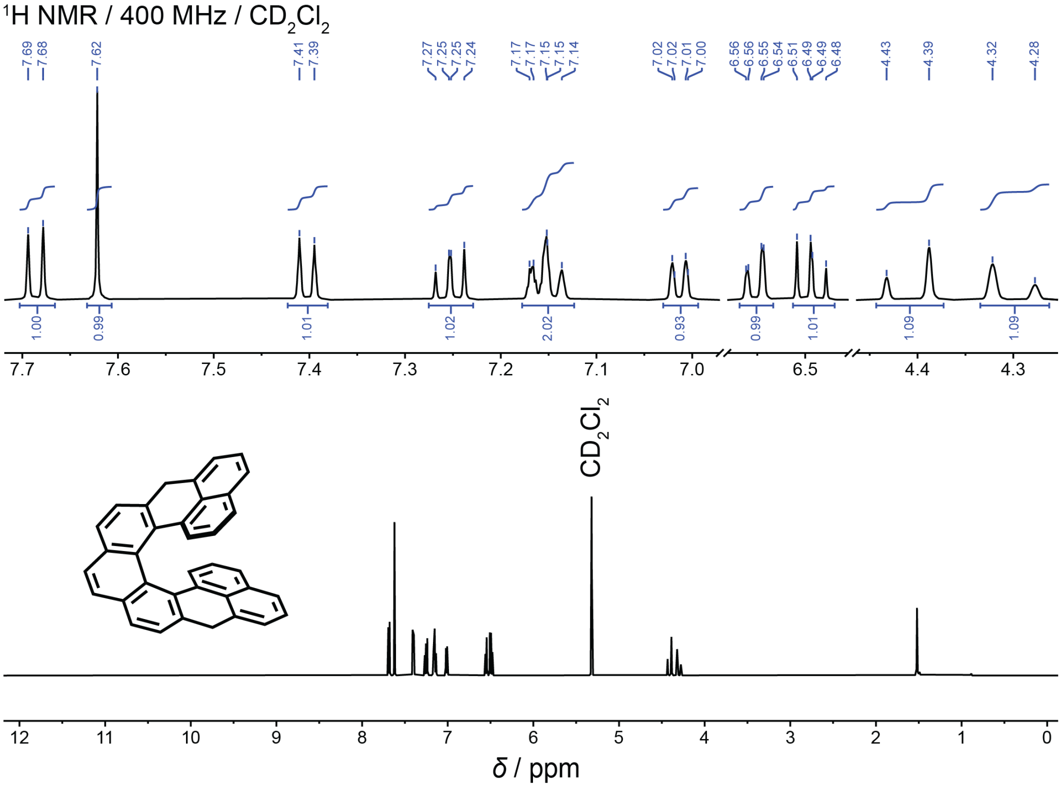

Figure S48. ^{1}H NMR spectrum of 2*H*-**NC**.

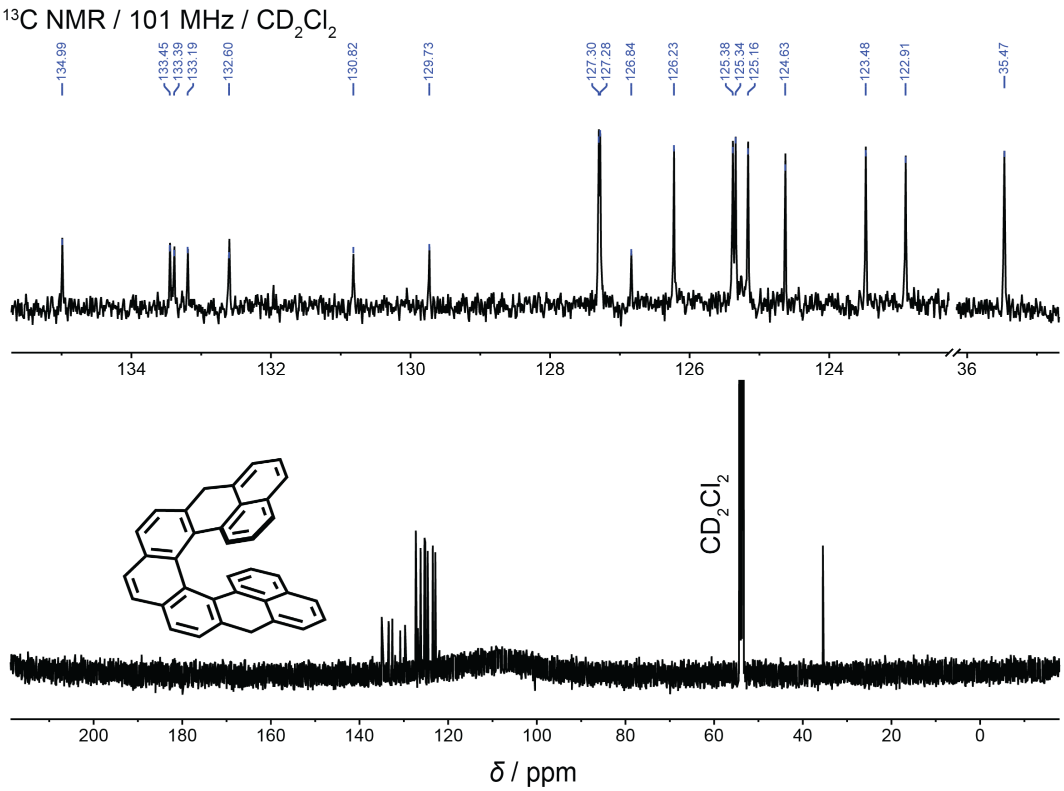

Figure S49. ^{13}C NMR spectrum of 2*H*-**NC**.

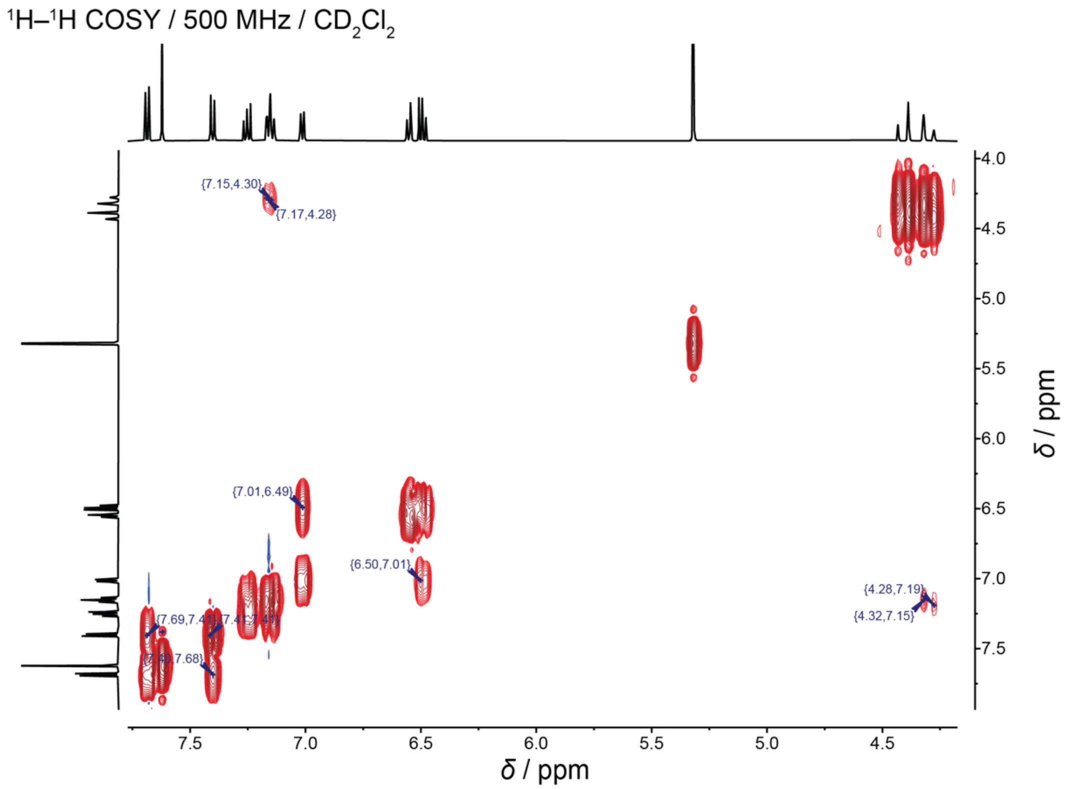

Figure S50. COSY NMR spectrum of 2*H*-**NC**.

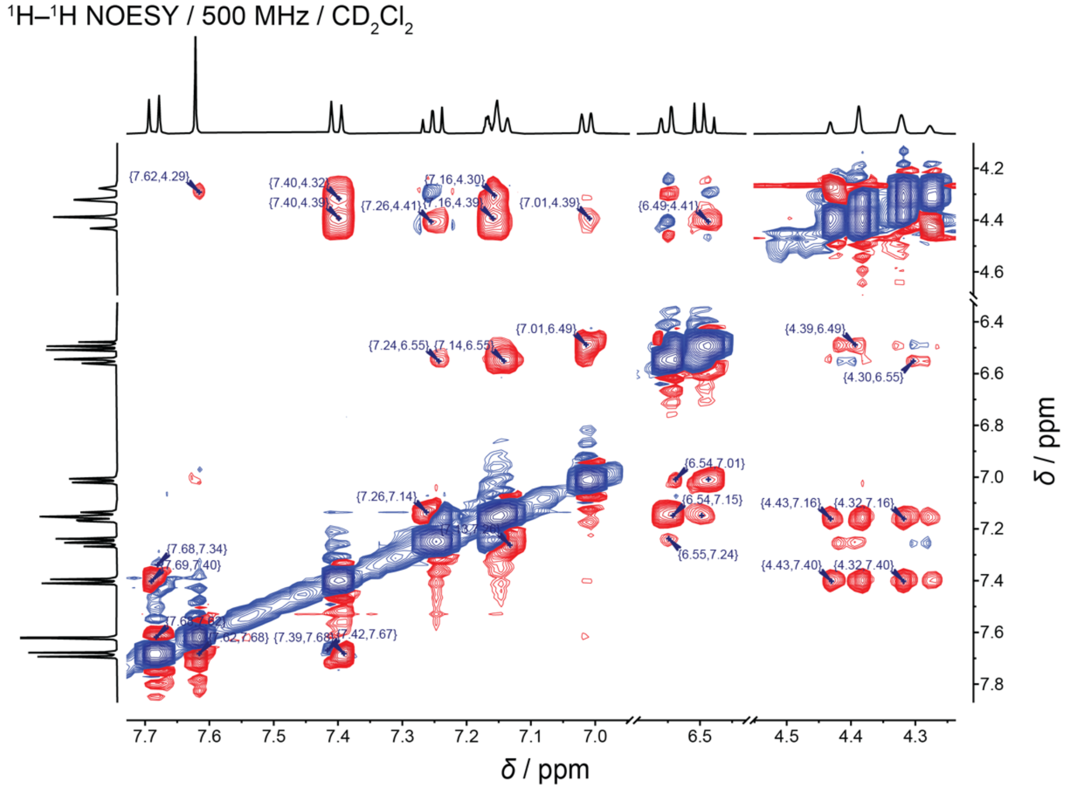

Figure S51. NOESY NMR spectrum of 2*H*-**NC**.

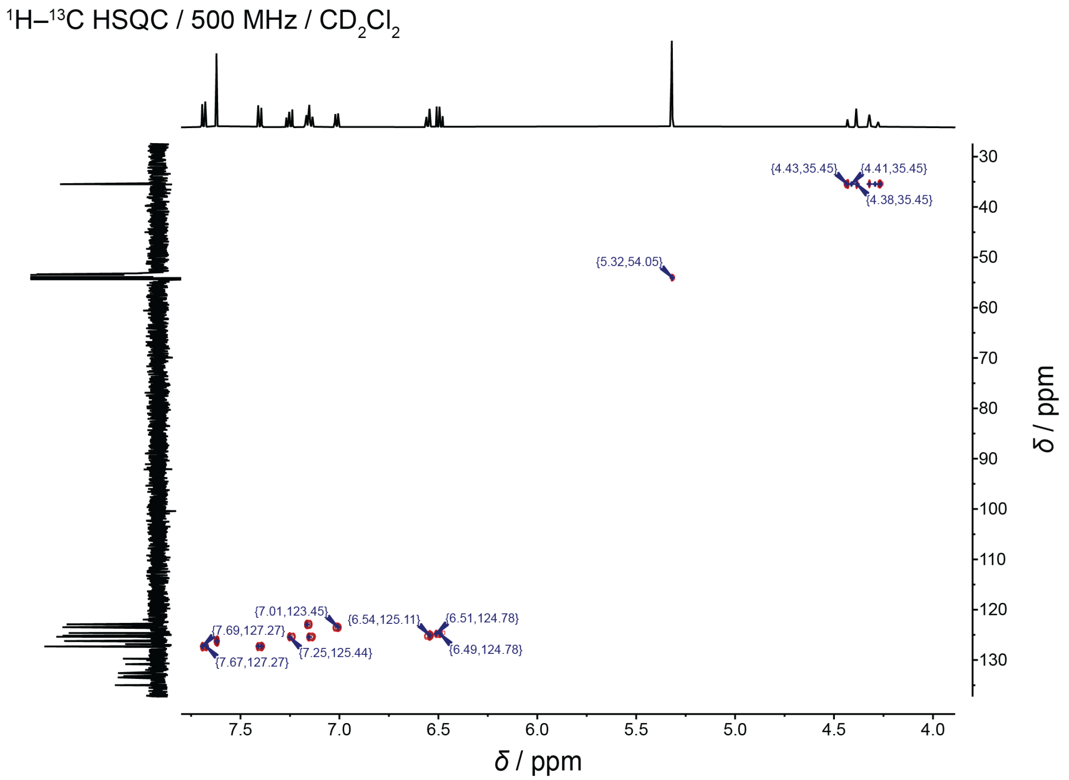

Figure S52. HSQC NMR spectrum of 2*H*-**NC**.

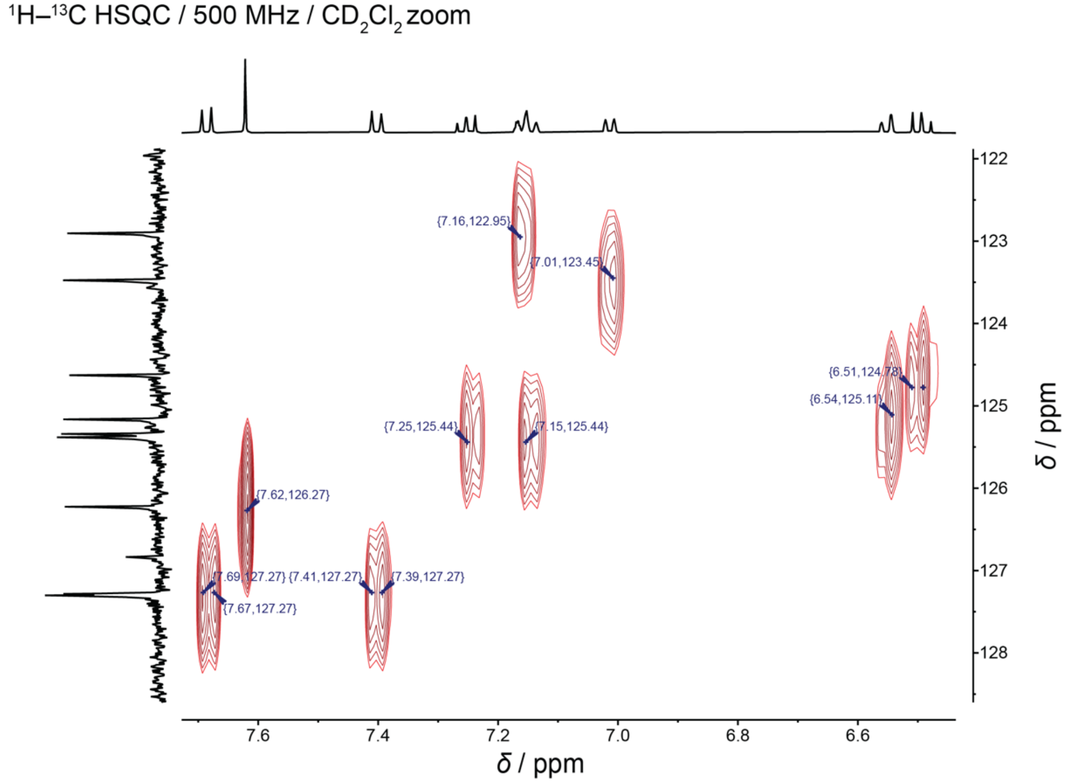

Figure S53. HSQC NMR spectrum of 2*H*-**NC**.

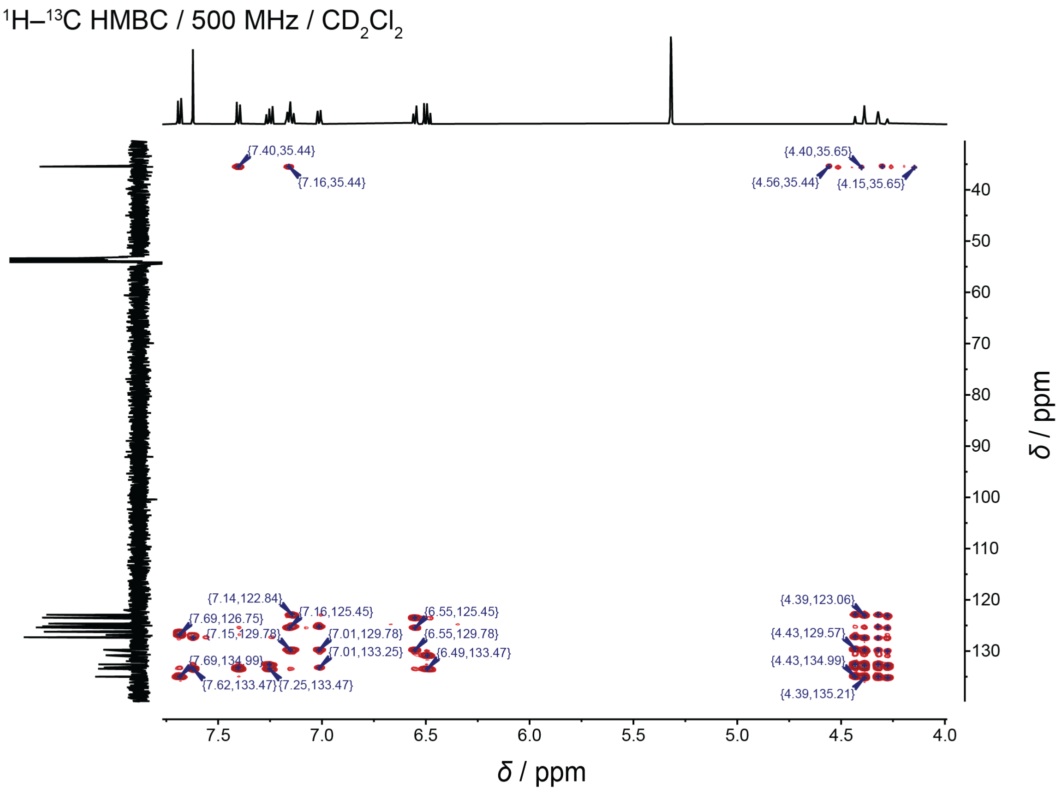

Figure S54. HMBC NMR spectrum of 2*H*-**NC**.

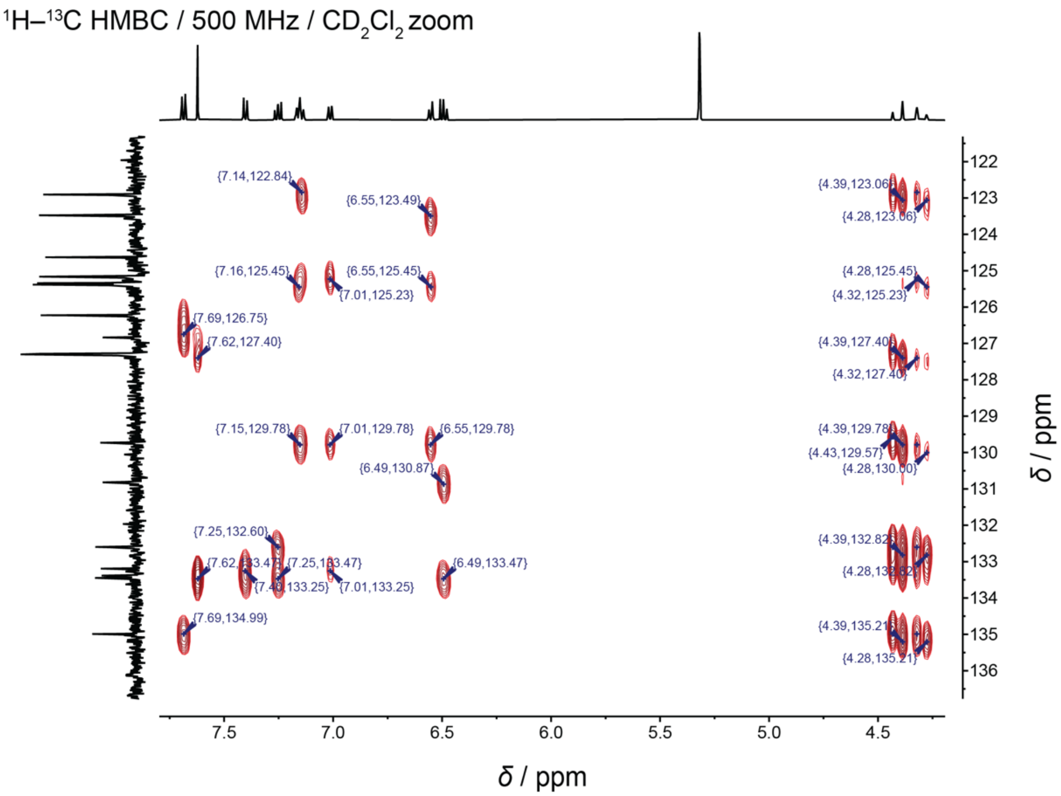

Figure S55. HMBC NMR spectrum of 2*H*-**NC**.

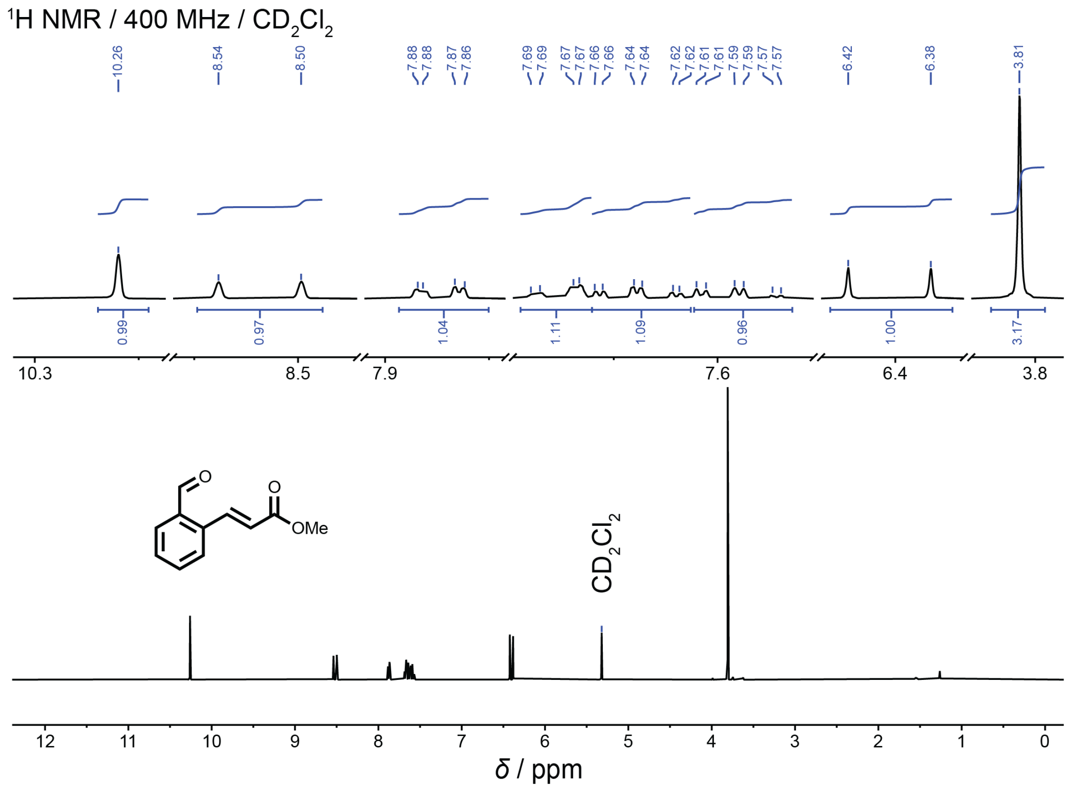

Figure S56. ^{1}H NMR spectrum of **8**.

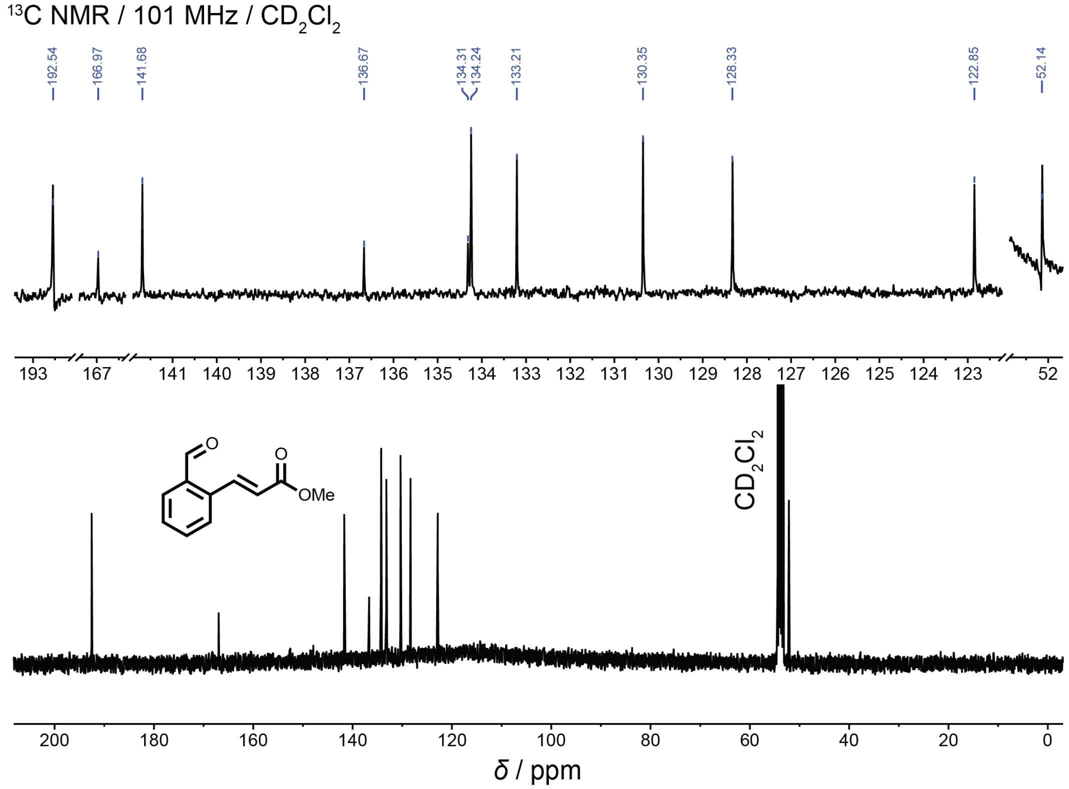

Figure S57. ^{13}C NMR spectrum of **8**.

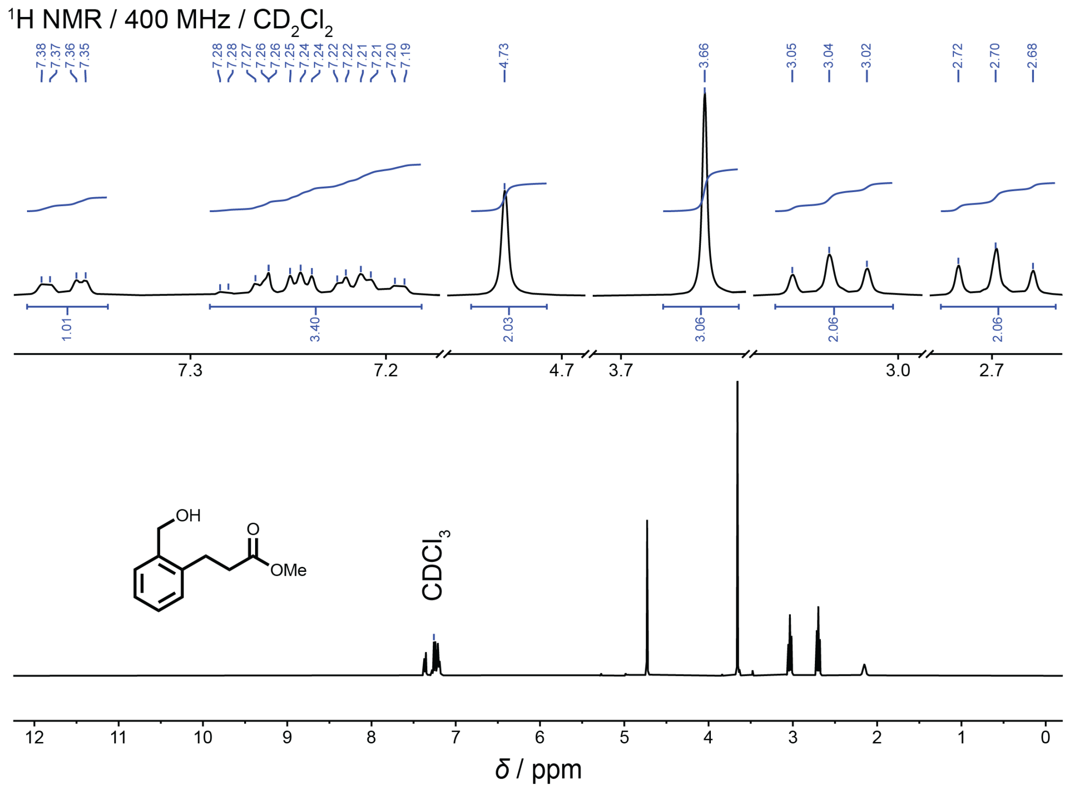

Figure S58. ^{1}H NMR spectrum of **9**.

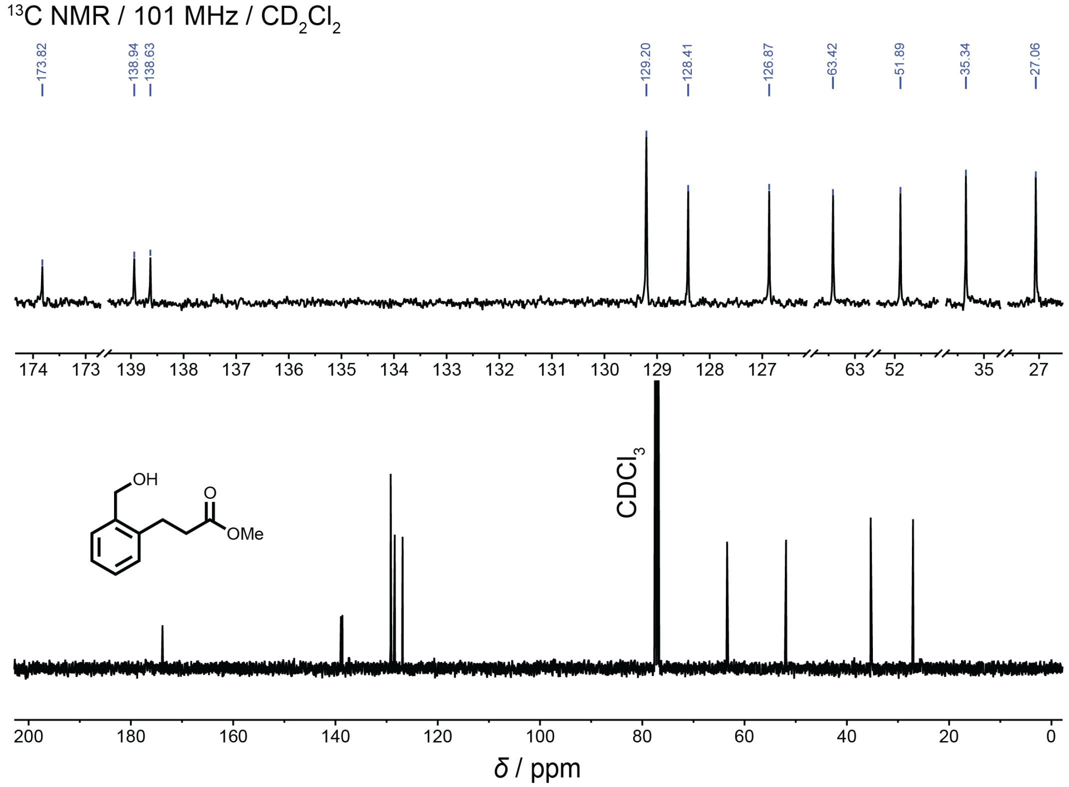

Figure S59. ^{13}C NMR spectrum of **9**.

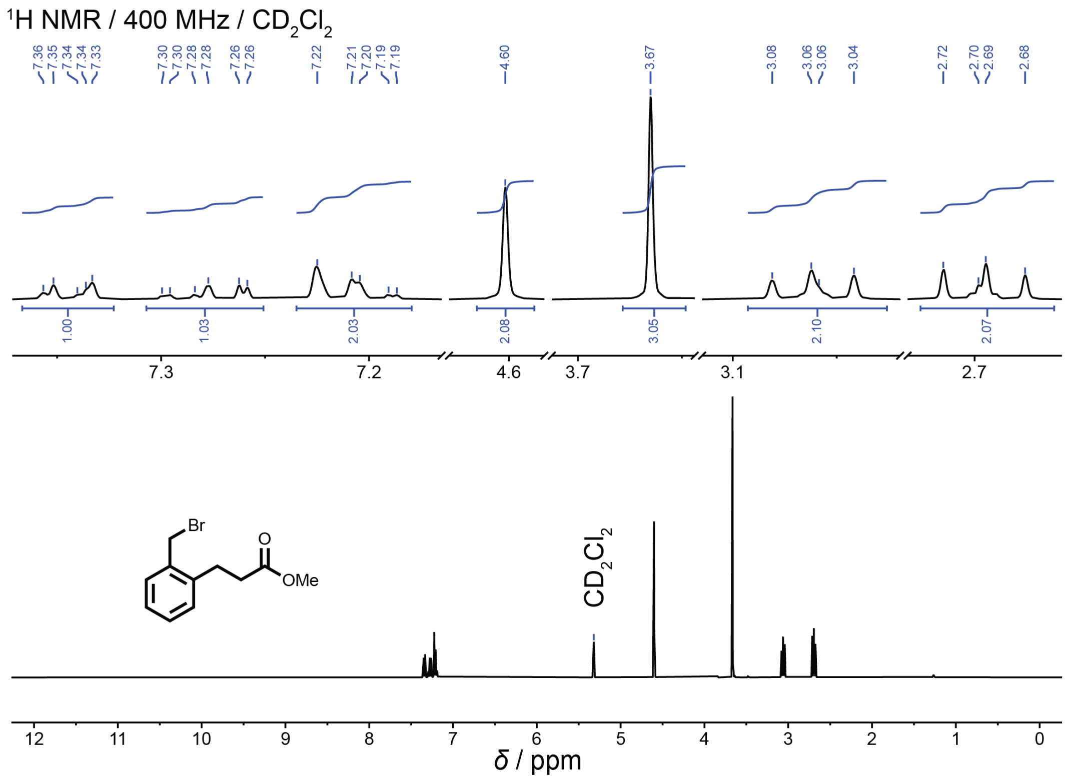

Figure S60. ^{1}H NMR spectrum of **10**.

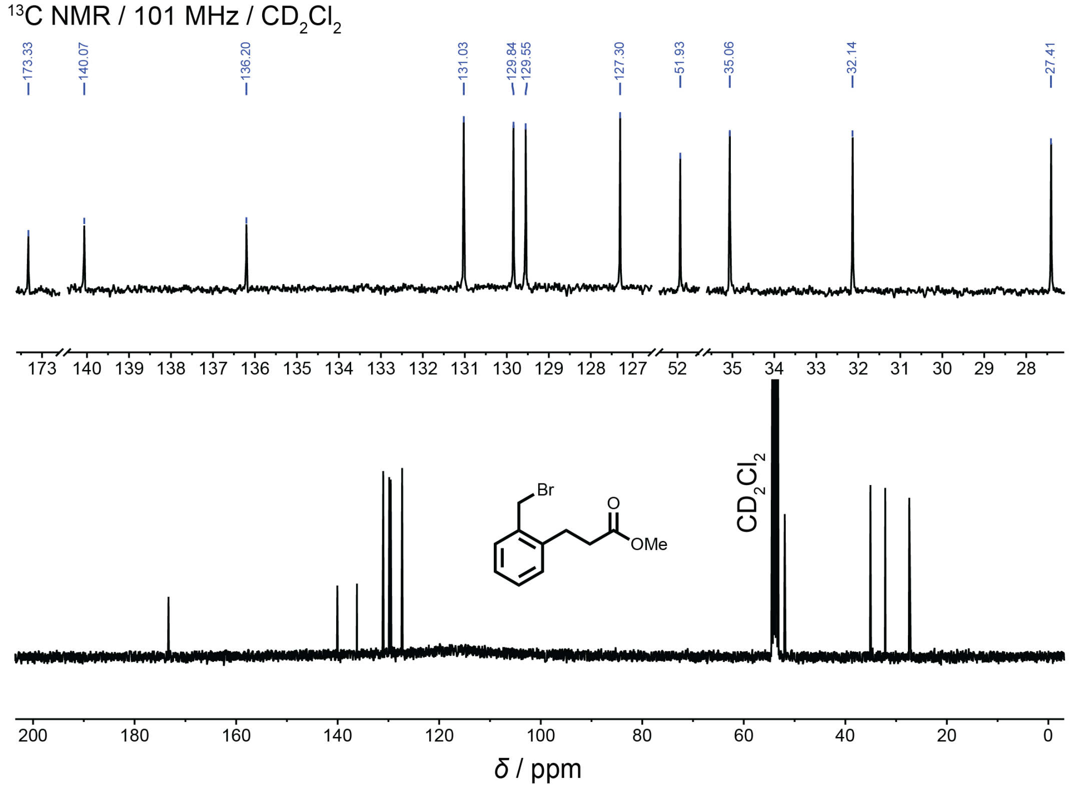

Figure S61. ^{13}C NMR spectrum of **10**.

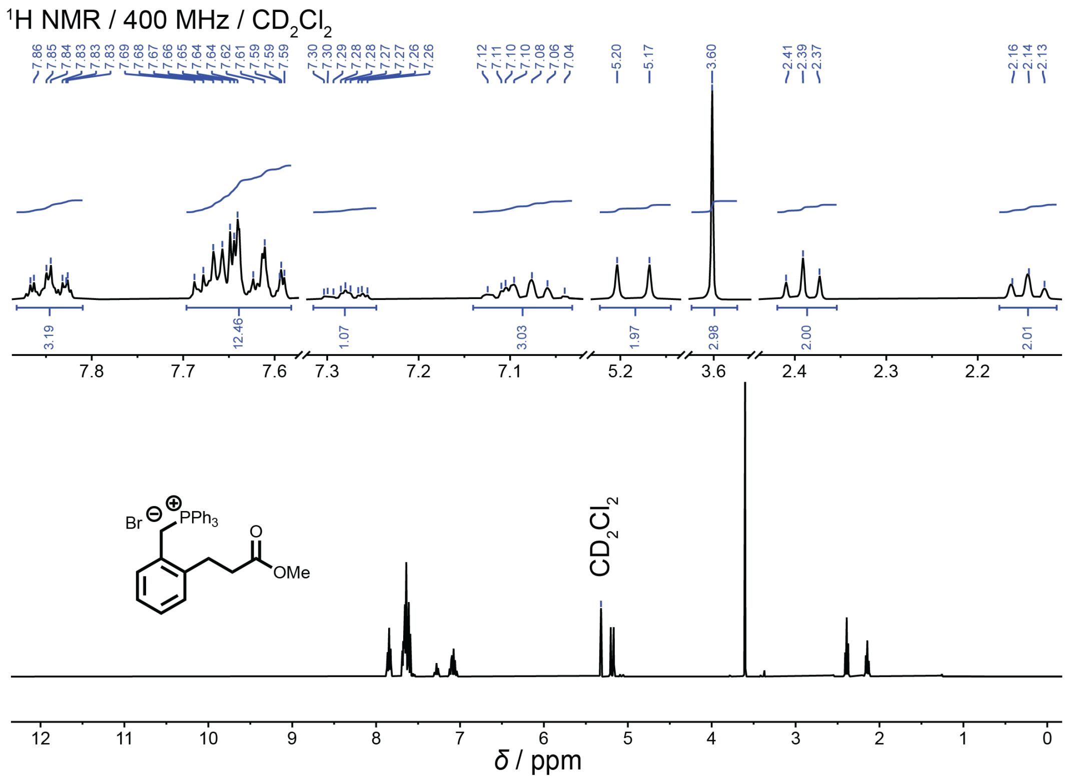

Figure S62. ^{1}H NMR spectrum of **11**.

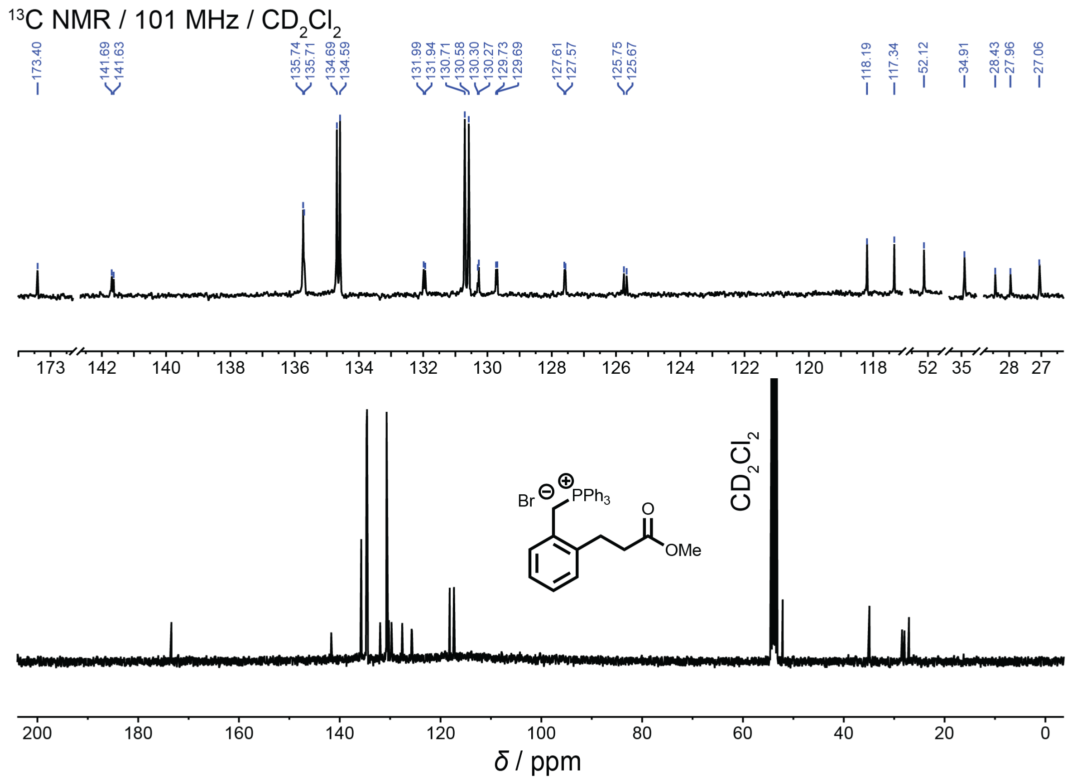

Figure S63. ^{13}C NMR spectrum of **11**.

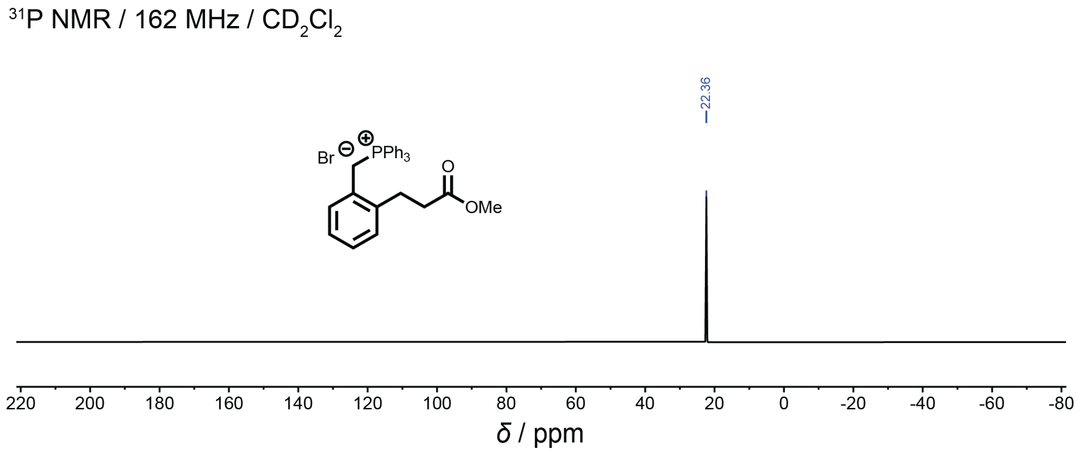

Figure S64. 31P NMR spectrum of **11**.

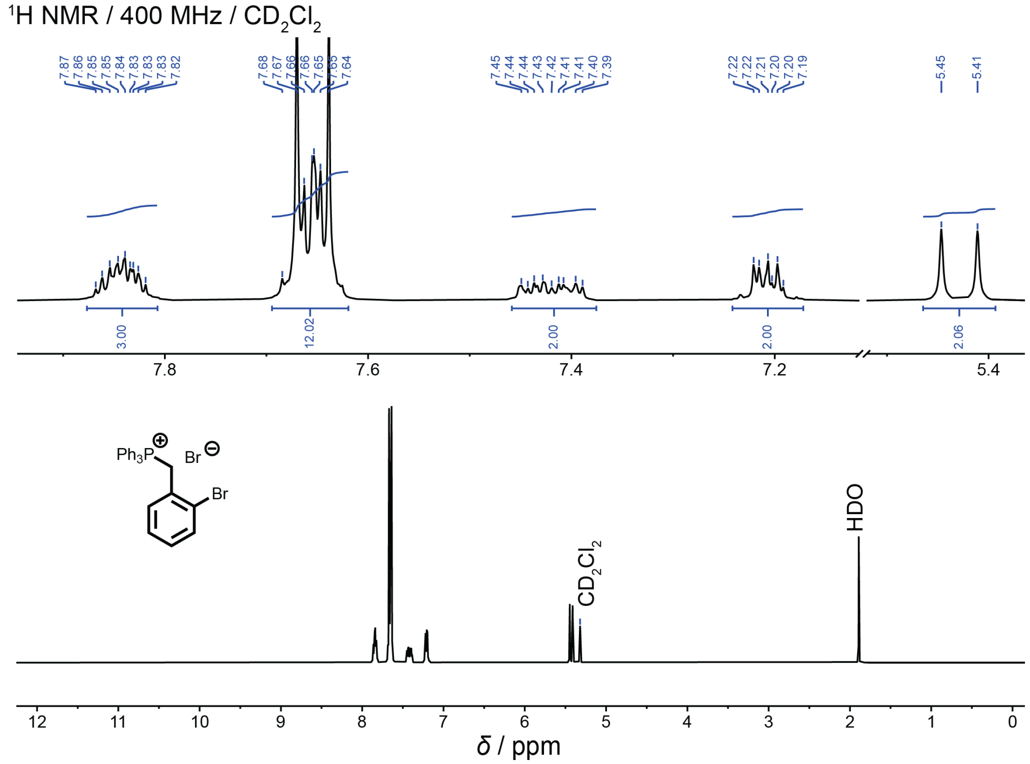

Figure S65. ^{1}H NMR spectrum of **13**.

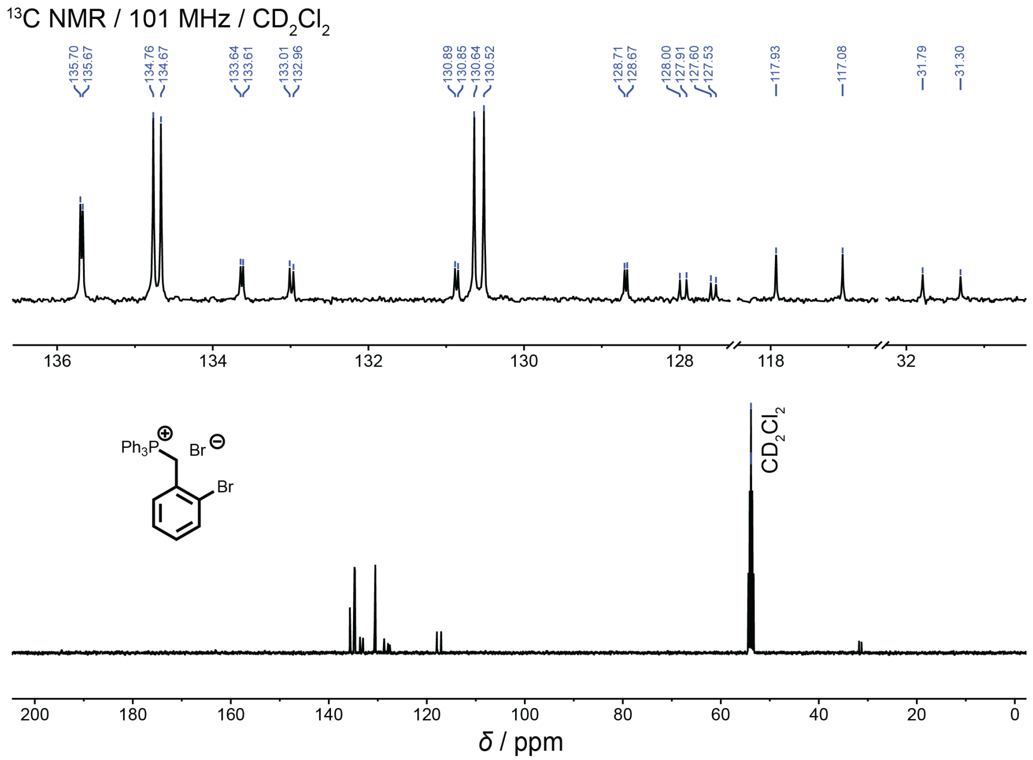

Figure S66. ^{13}C NMR spectrum of **13**.

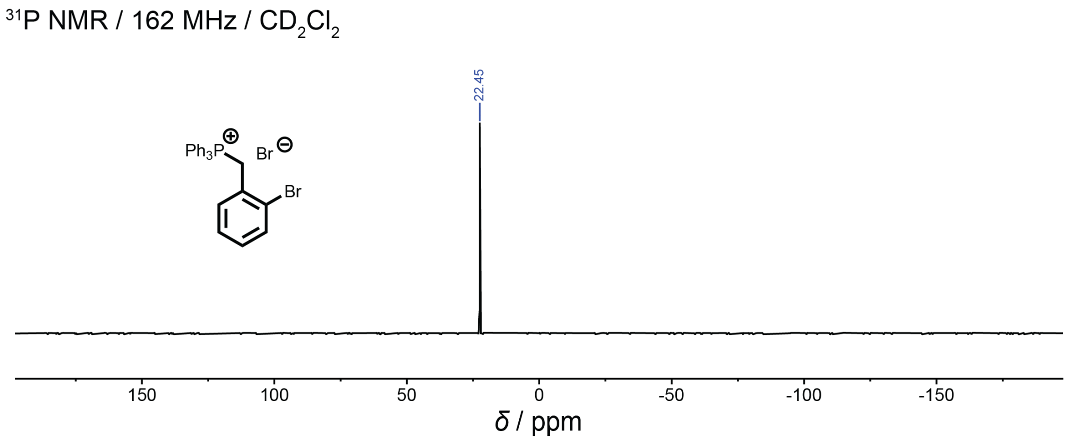

Figure S67. ^{31}P NMR spectrum of **13**.

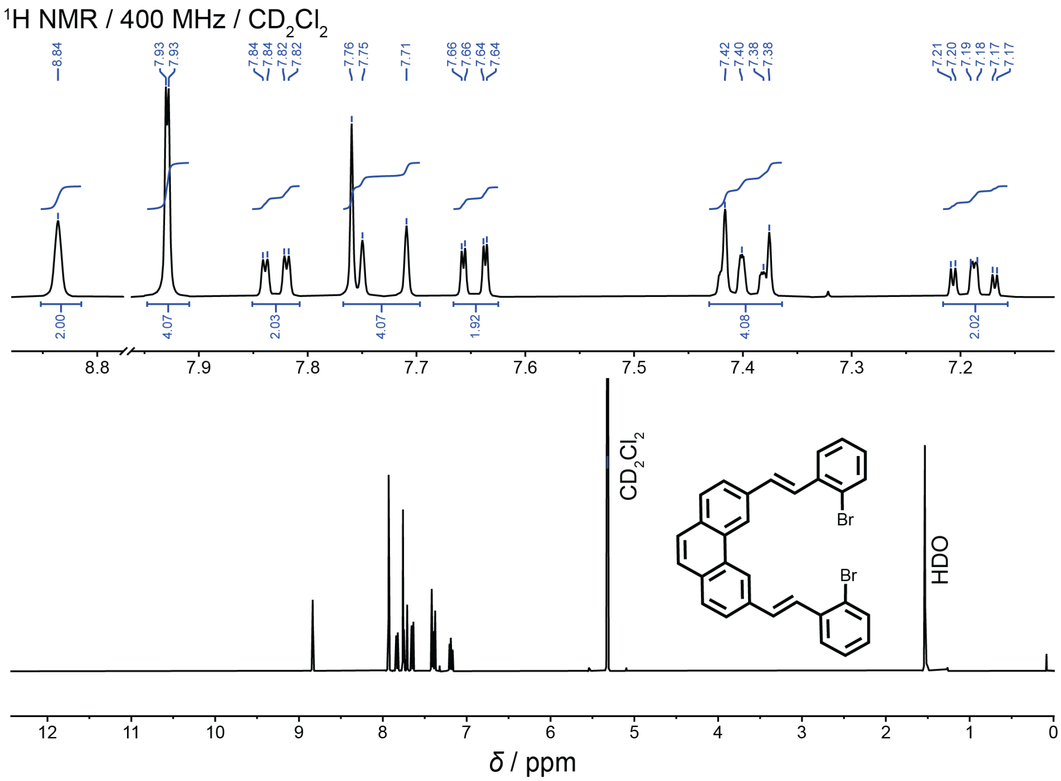

Figure S68. ^{1}H NMR spectrum of **14**.

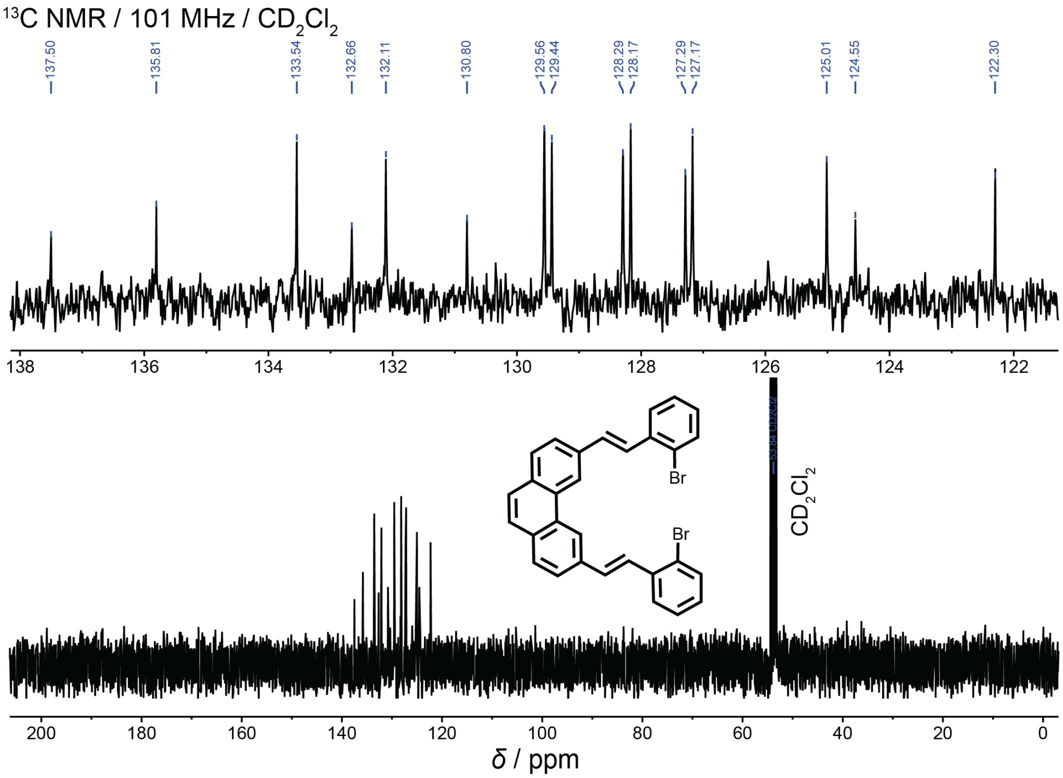

Figure S69. ^{13}C NMR spectrum of **14**.

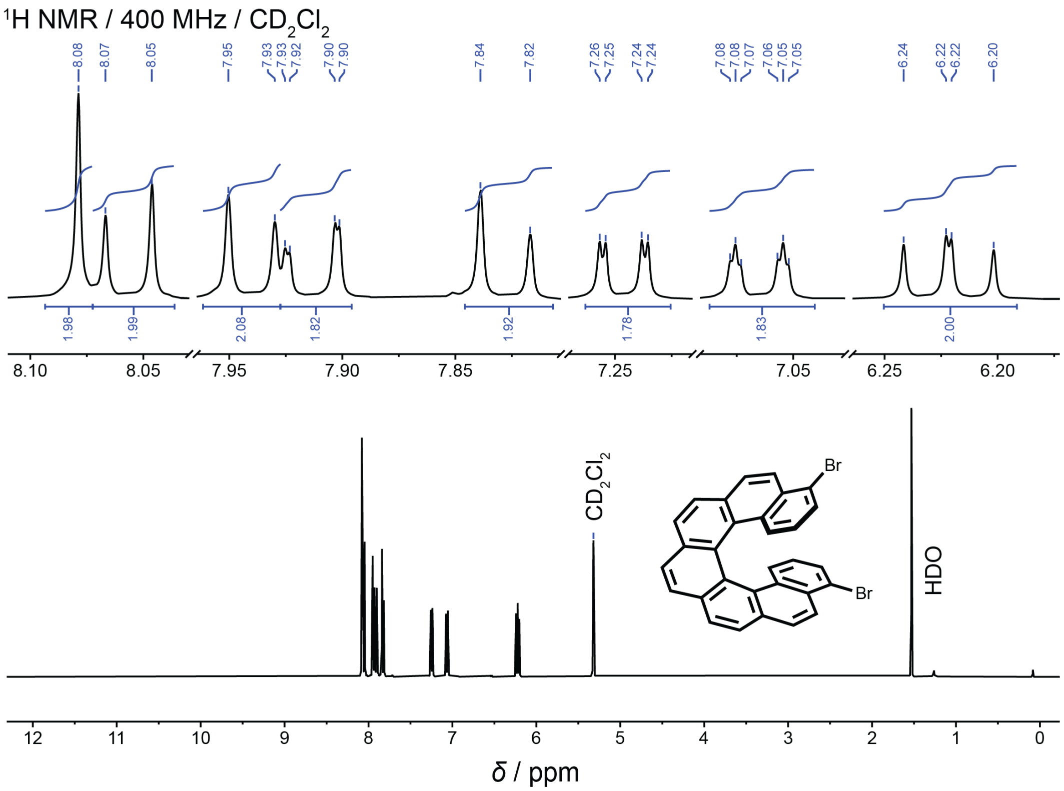

Figure S70. ^{1}H NMR spectrum of **15**.

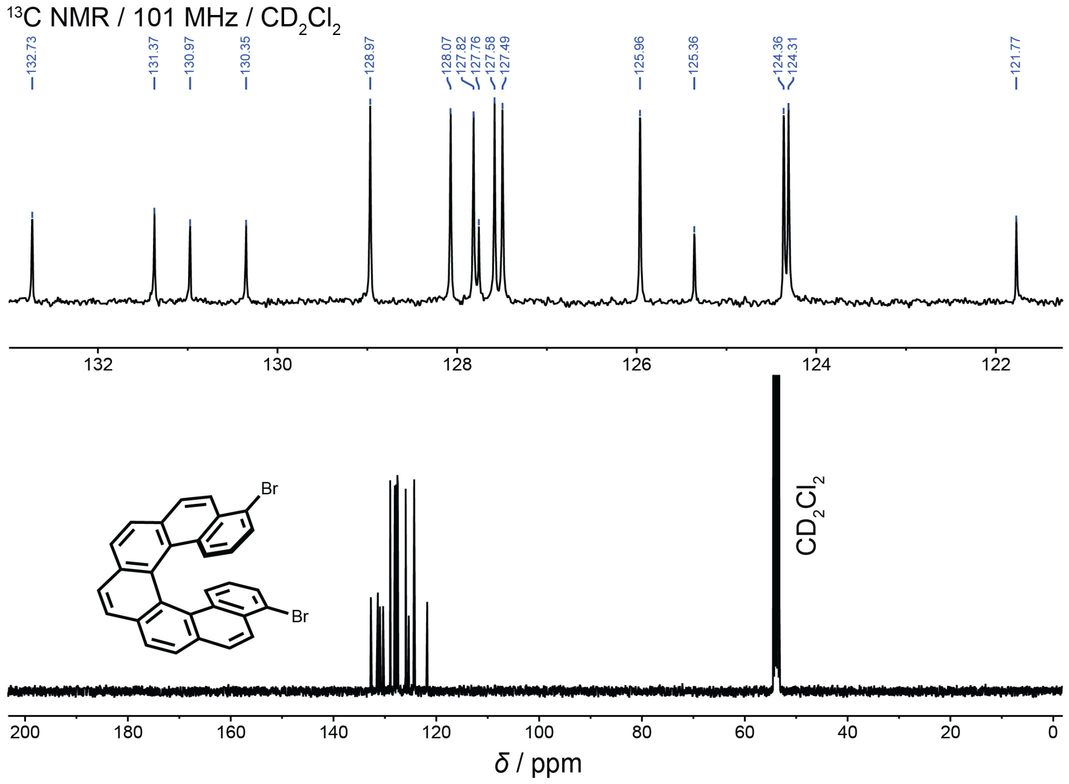

Figure S71. ^{13}C NMR spectrum of **15**.

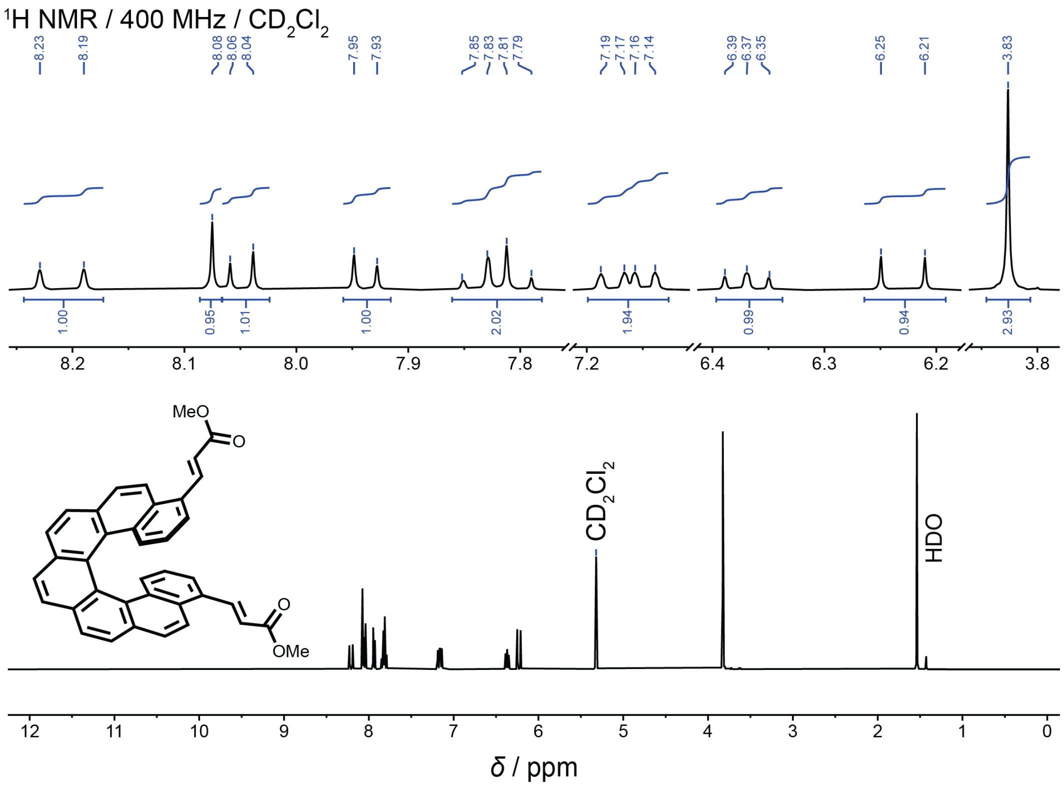

Figure S72. ^{1}H NMR spectrum of **16**.

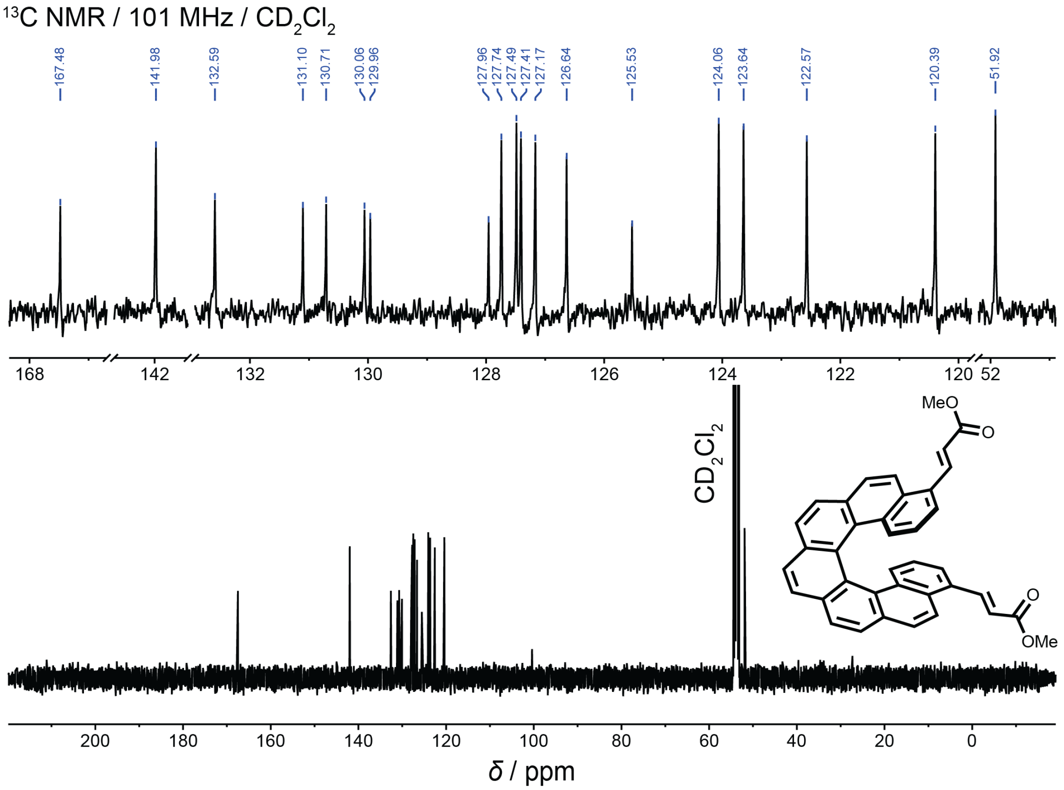

Figure S73. ^{13}C NMR spectrum of **16**.

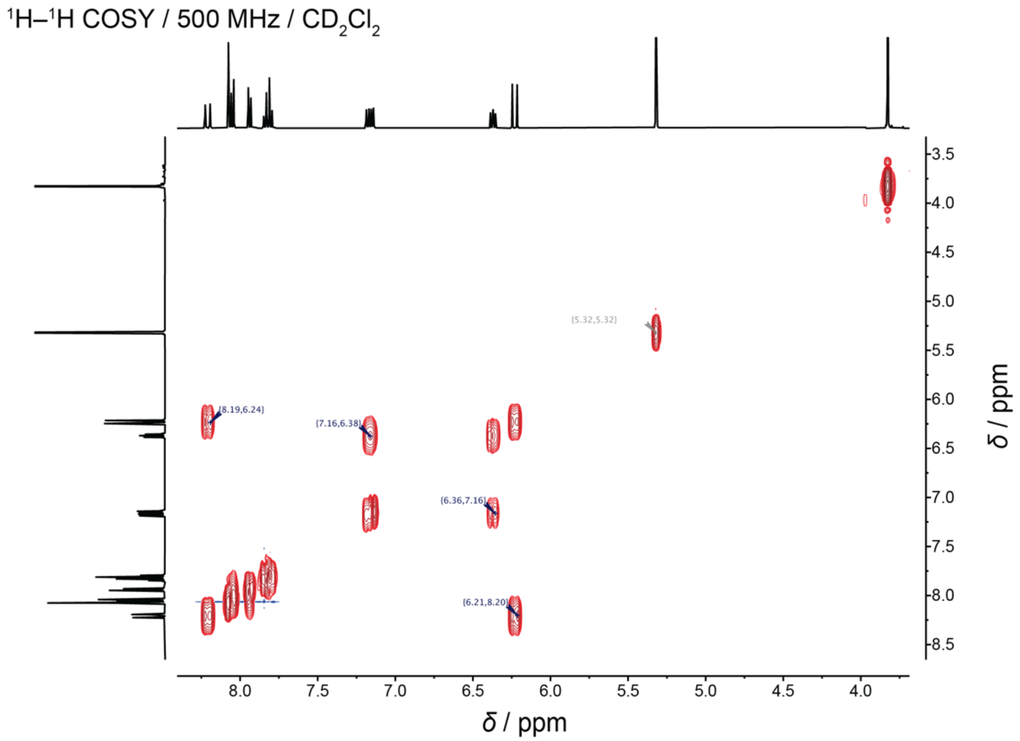

Figure S74. COSY NMR spectrum of **16**.

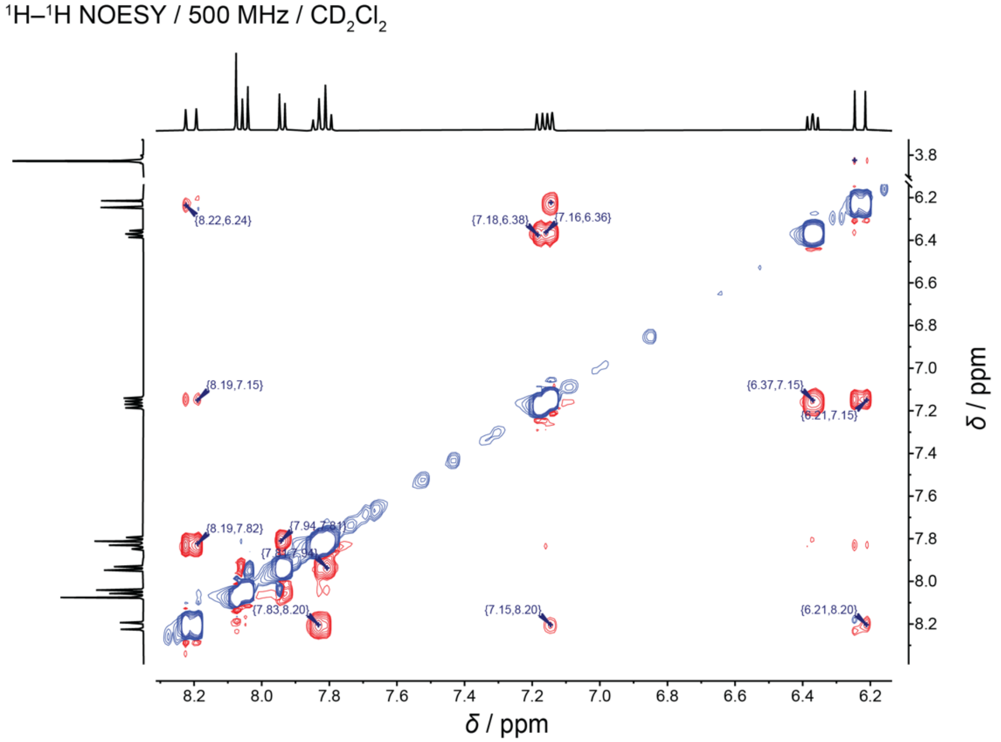

Figure S75. NOESY NMR spectrum of **16**.

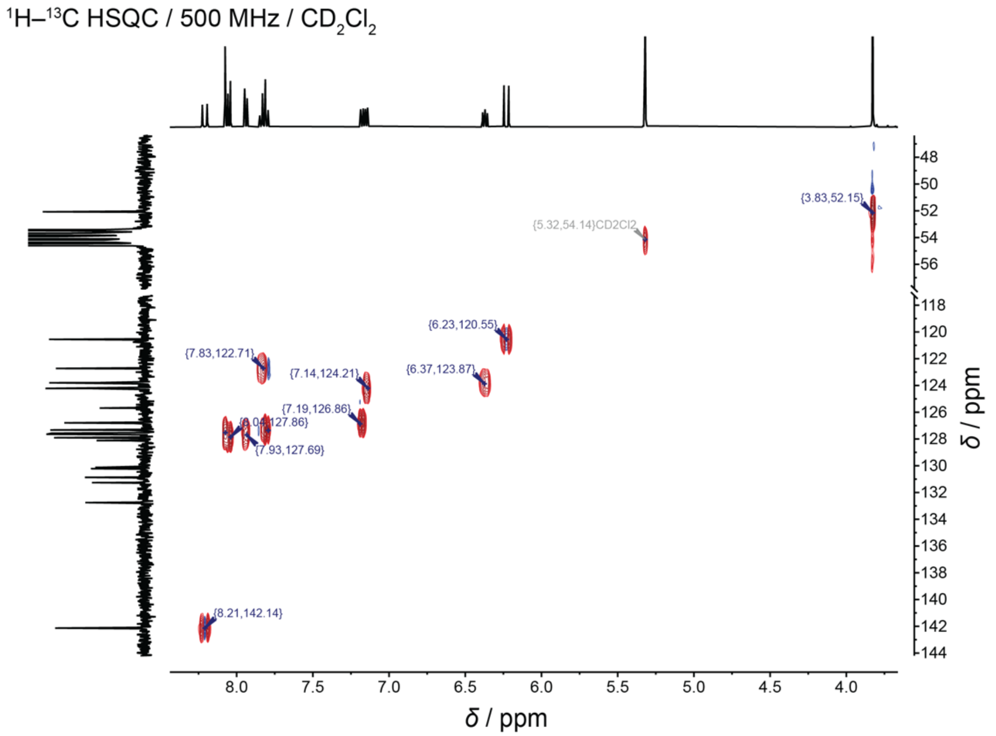

Figure S76. HSQC NMR spectrum of **16**.

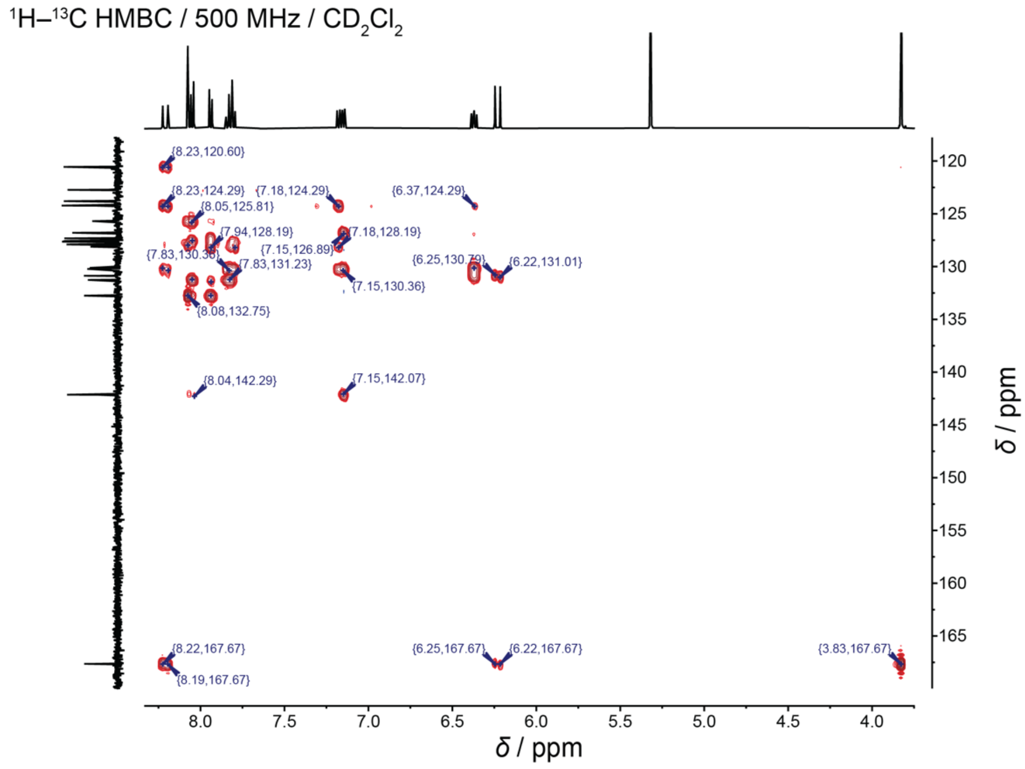

Figure S77. HMBC NMR spectrum of **16**.

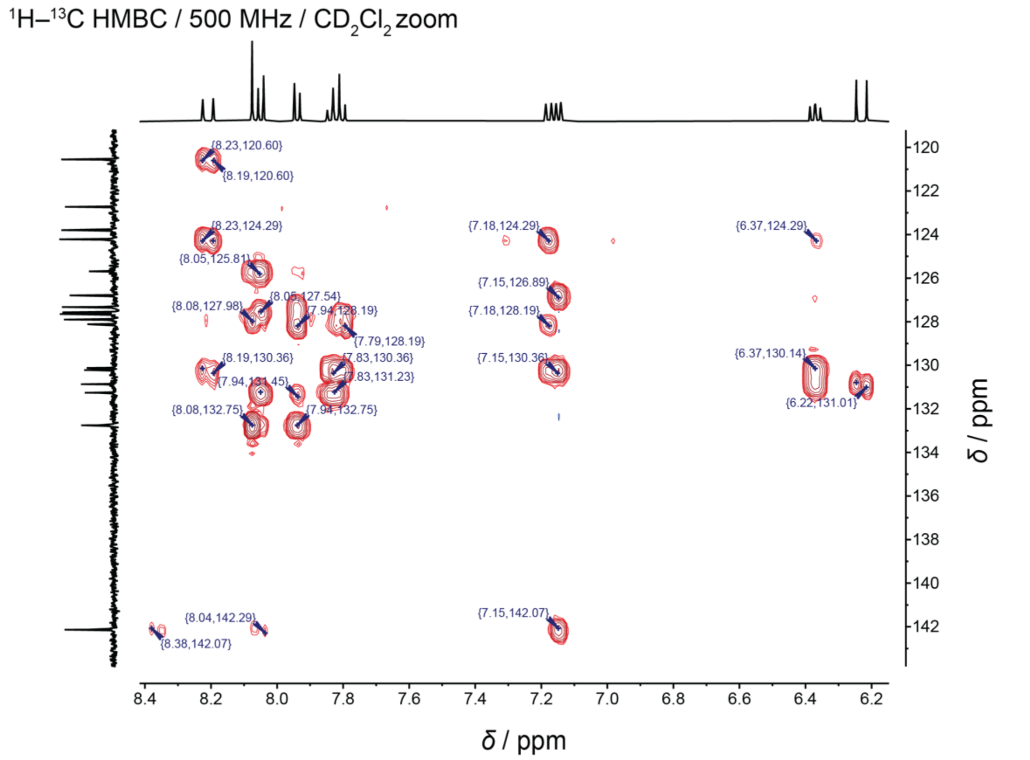

Figure S78. HMBC NMR spectrum of **16**.

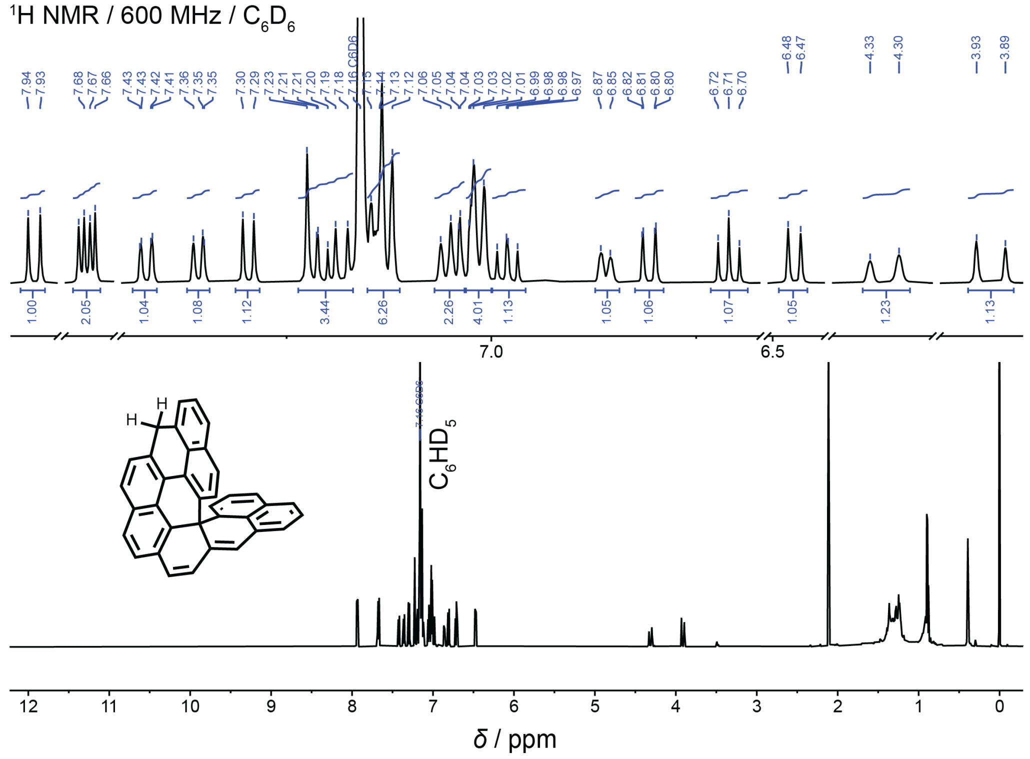

Figure S79. ^{1}H NMR spectrum of c-**NC**.

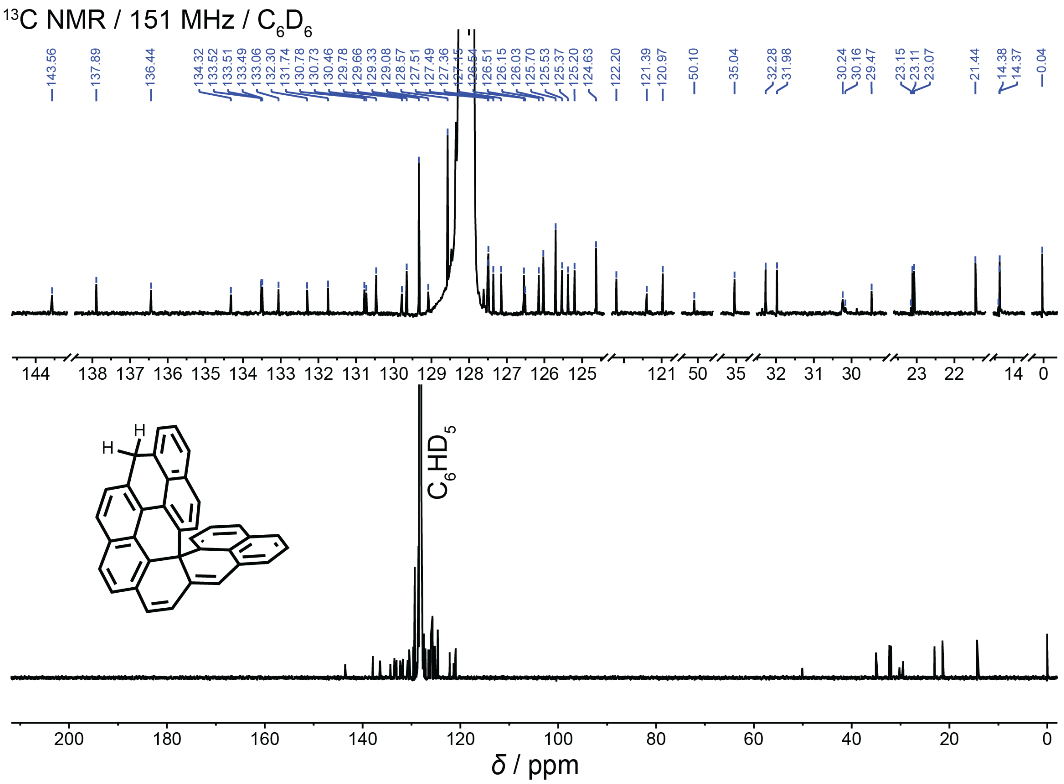

Figure S80. ^{13}C NMR spectrum of c-**NC**.

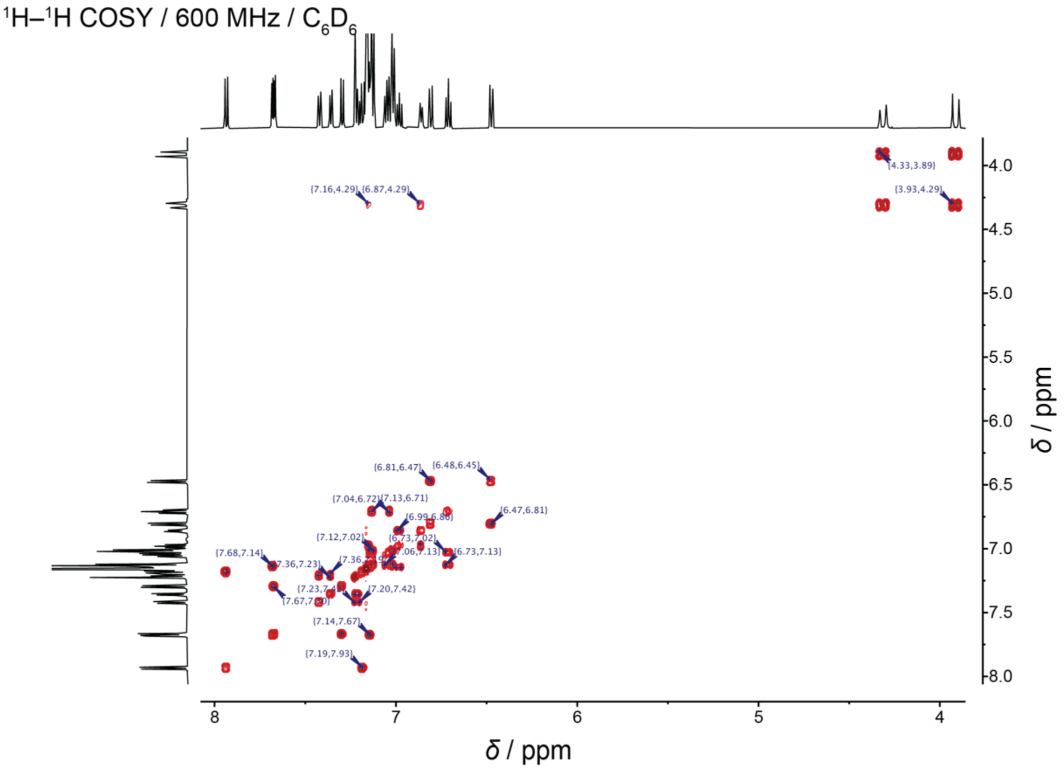

Figure S81. COSY NMR spectrum of c-**NC**.

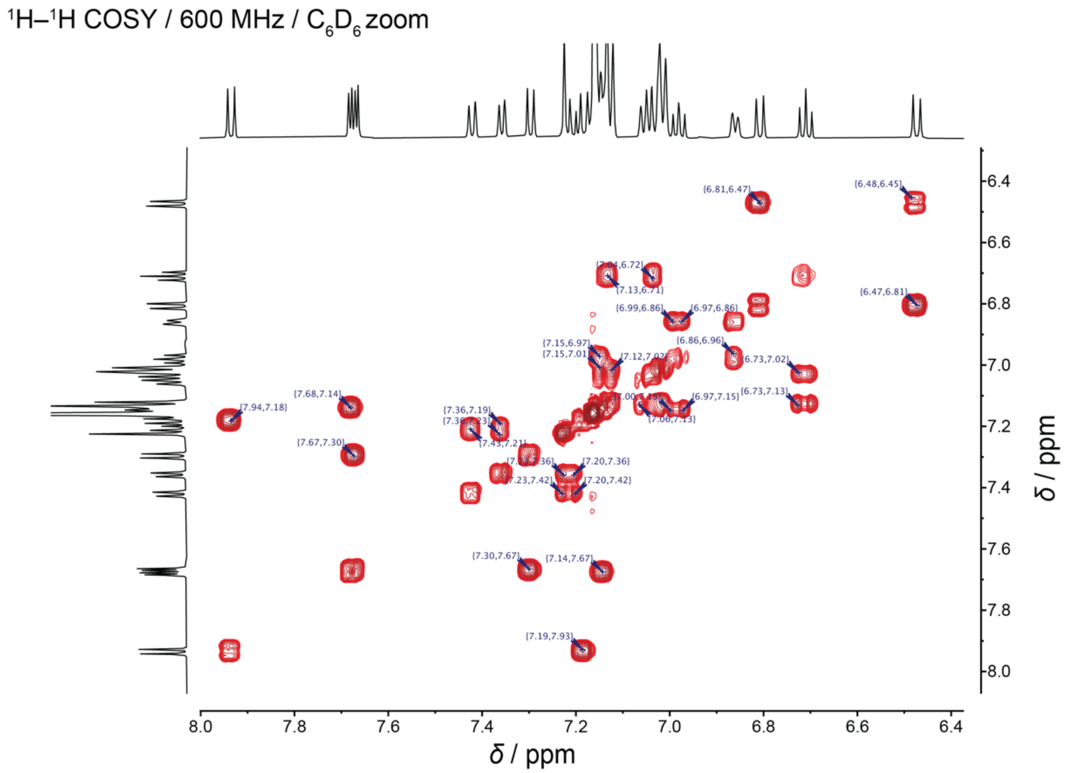

Figure S82. COSY NMR spectrum of c-**NC**.

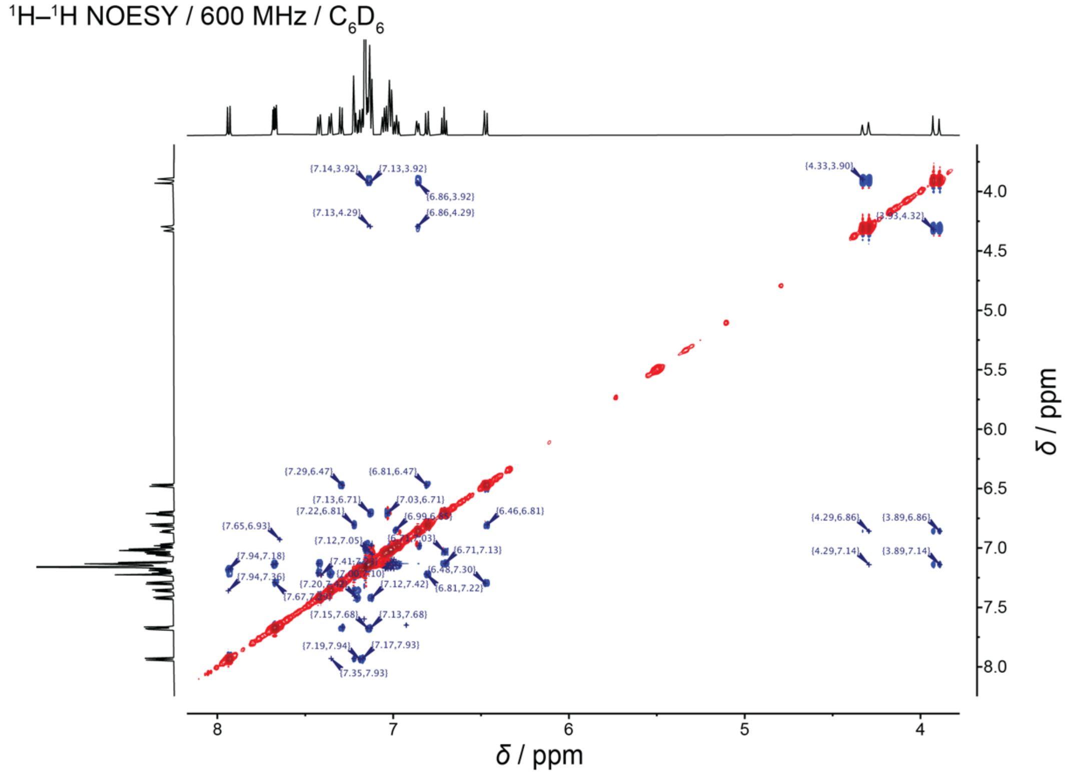

Figure S83. NOESY NMR spectrum of c-**NC**.

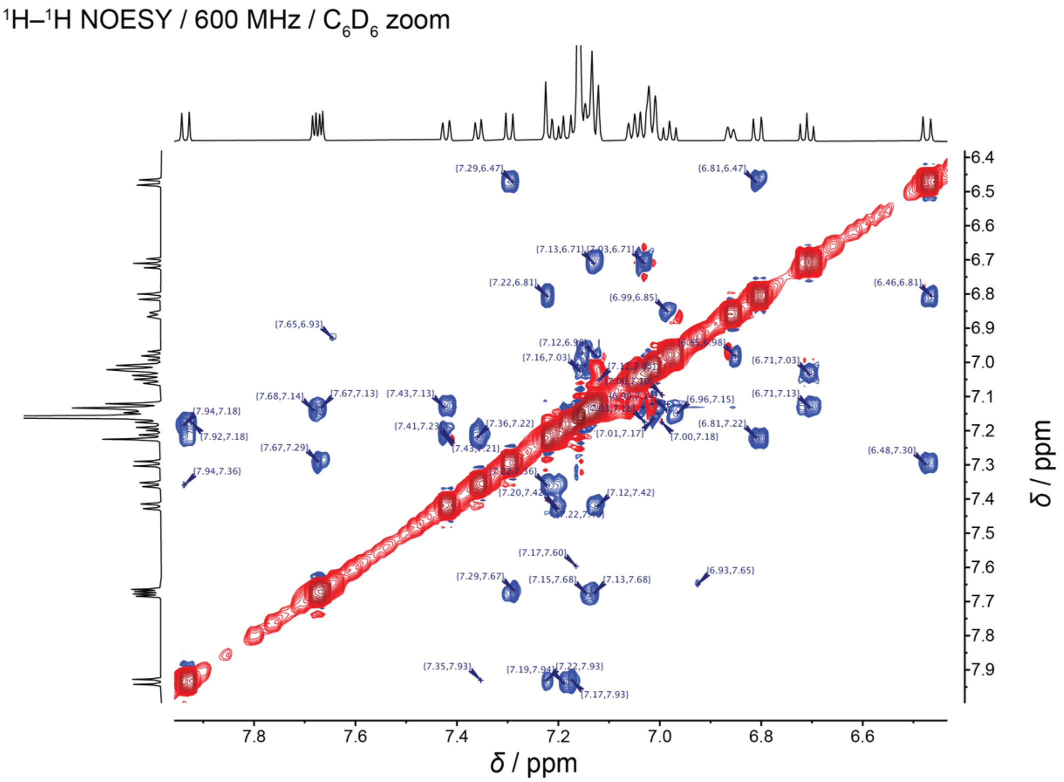

Figure S84. NOESY NMR spectrum of c-**NC**.

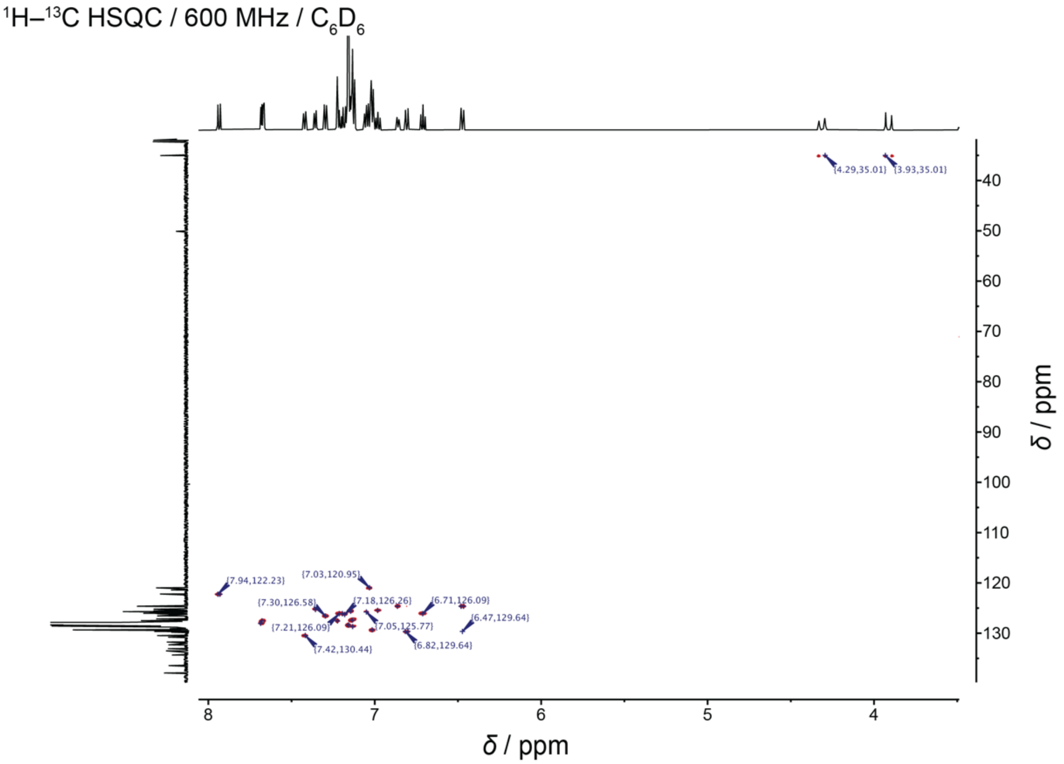

Figure S85. HSQC NMR spectrum of c-**NC**.

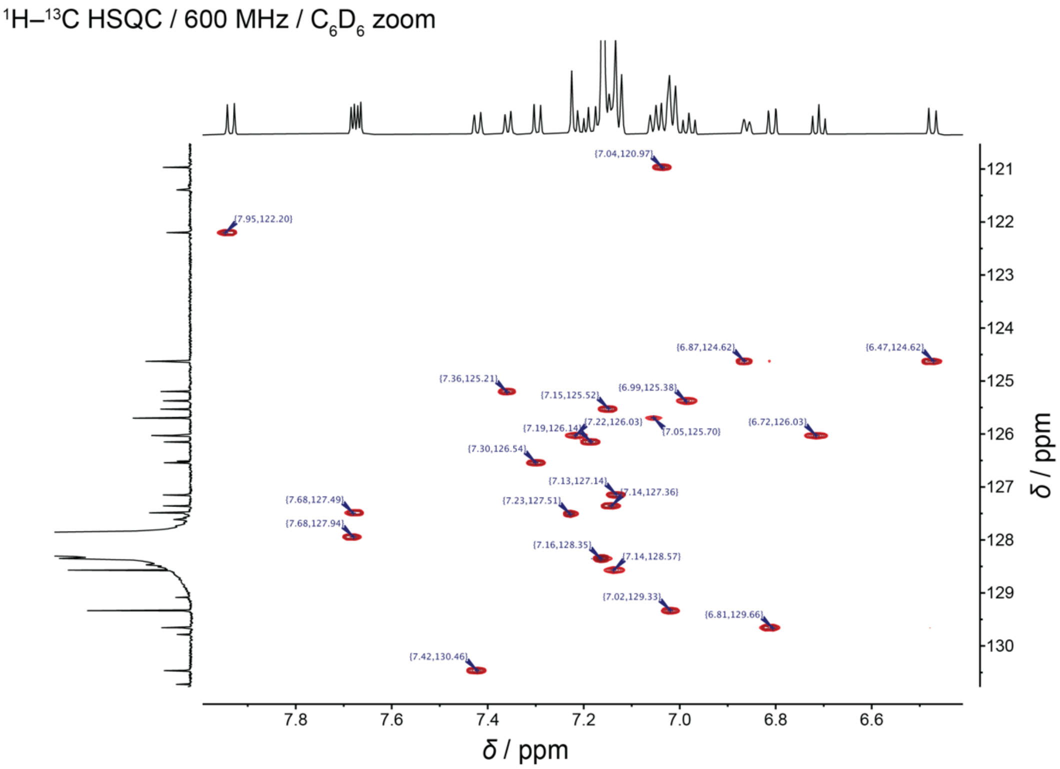

Figure S86. HSQC NMR spectrum of c-**NC**.

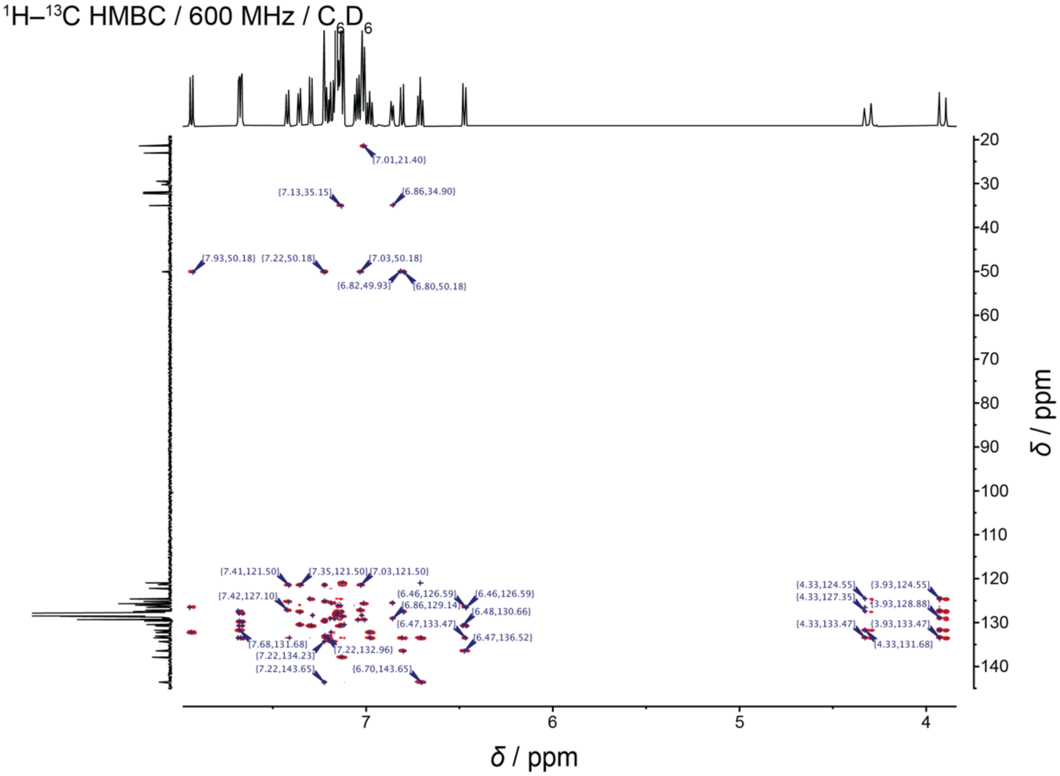

Figure S87. HMBC NMR spectrum of c-**NC**.

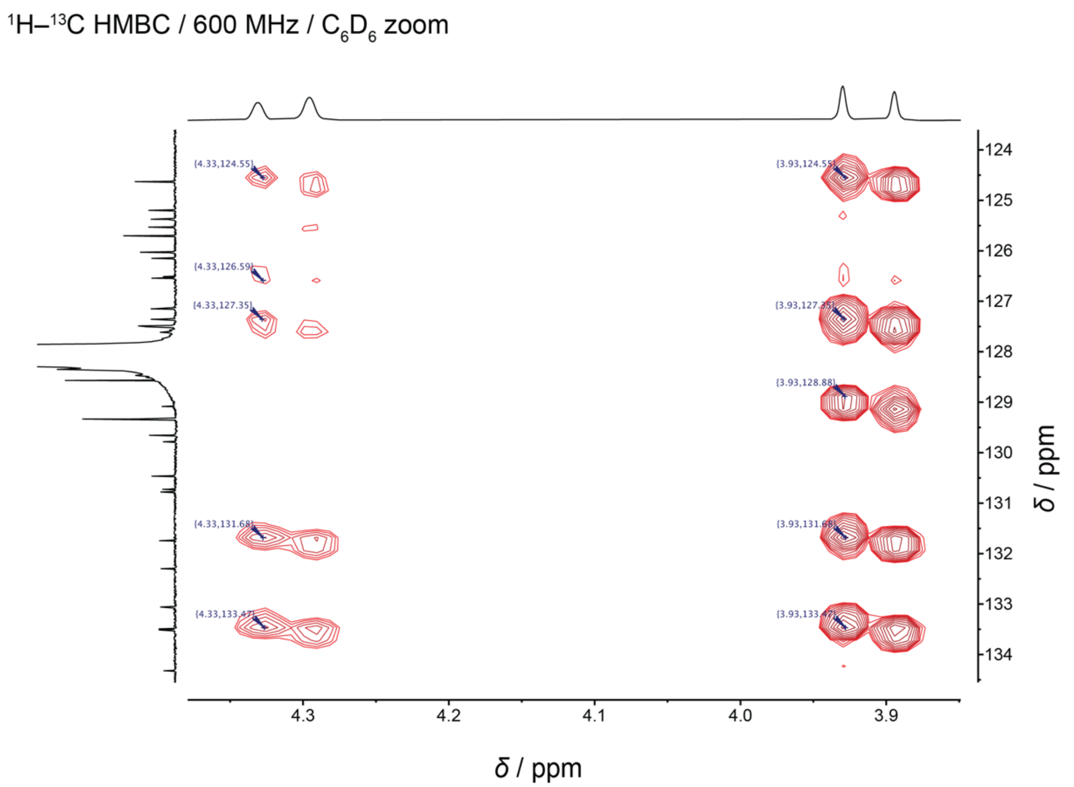

Figure S88. HMBC NMR spectrum of c-**NC**.

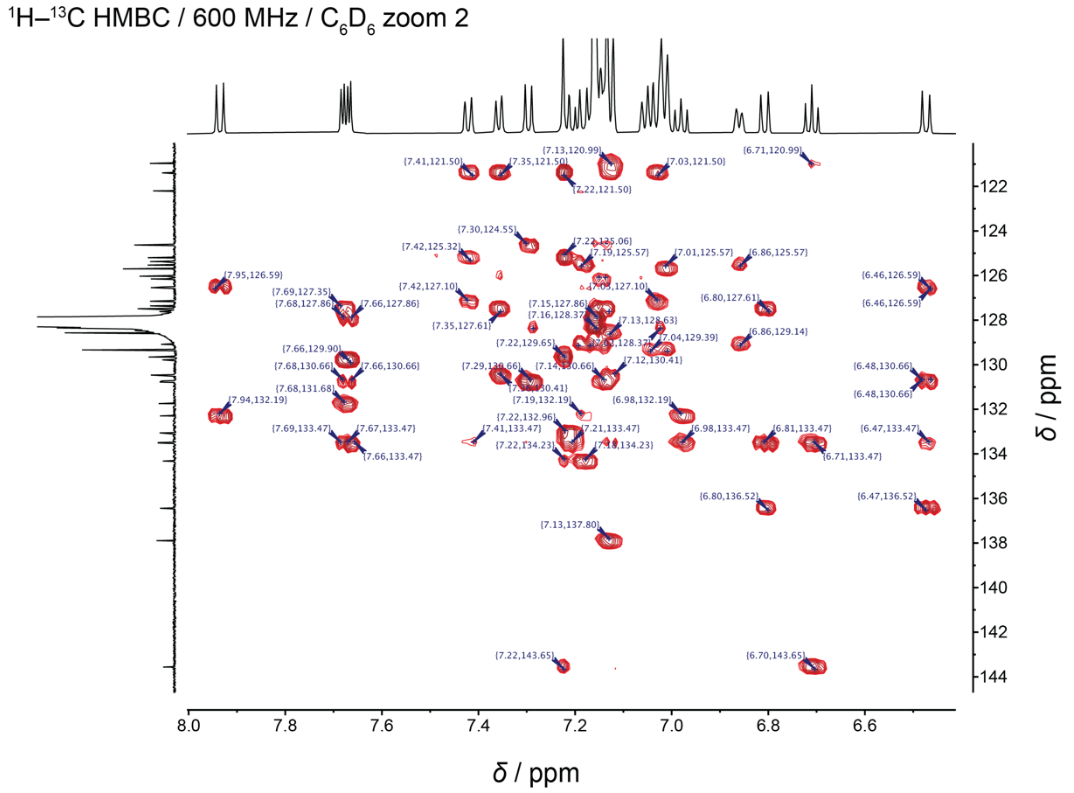

Figure S89. HMBC NMR spectrum of c-**NC**.

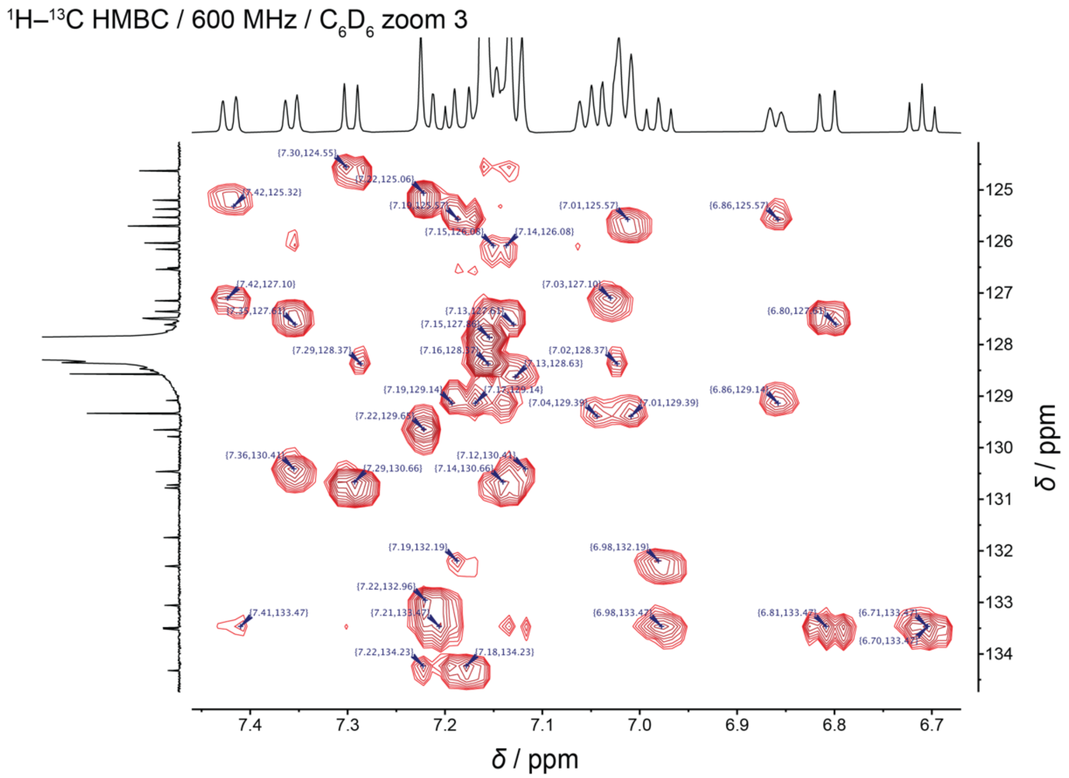

Figure S90. HMBC NMR spectrum of c-**NC.**

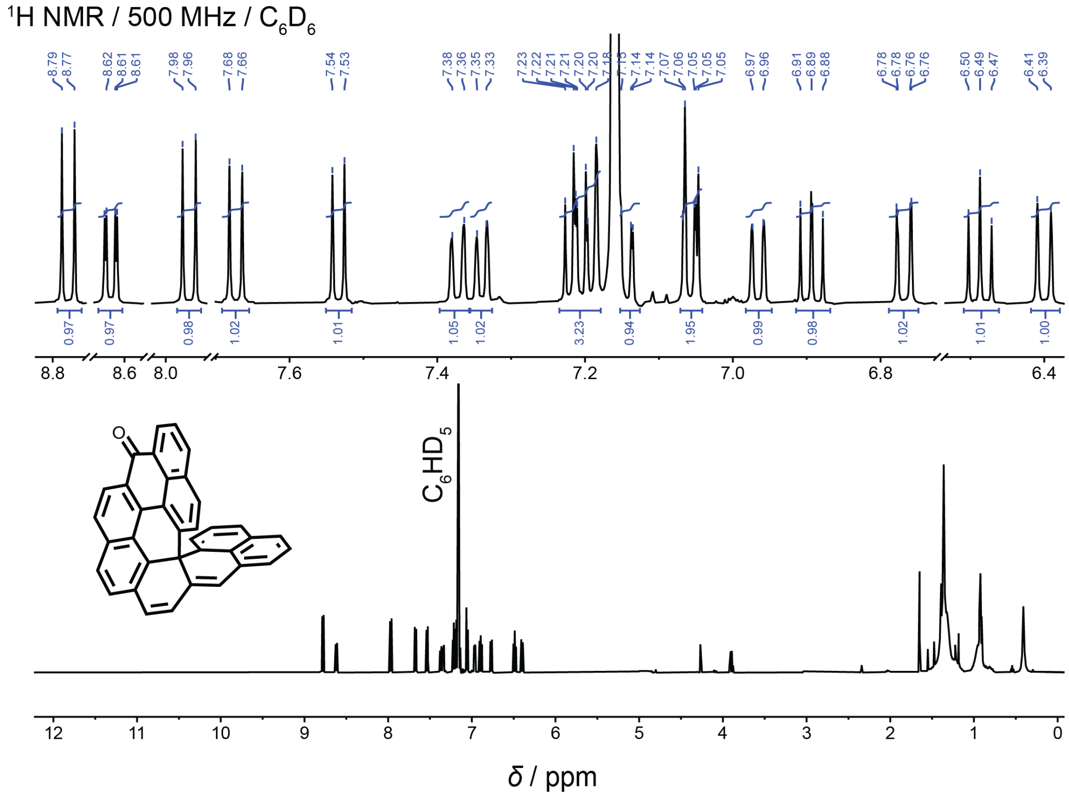

Figure S91. ¹H NMR spectrum of *O*-c-**NC.**

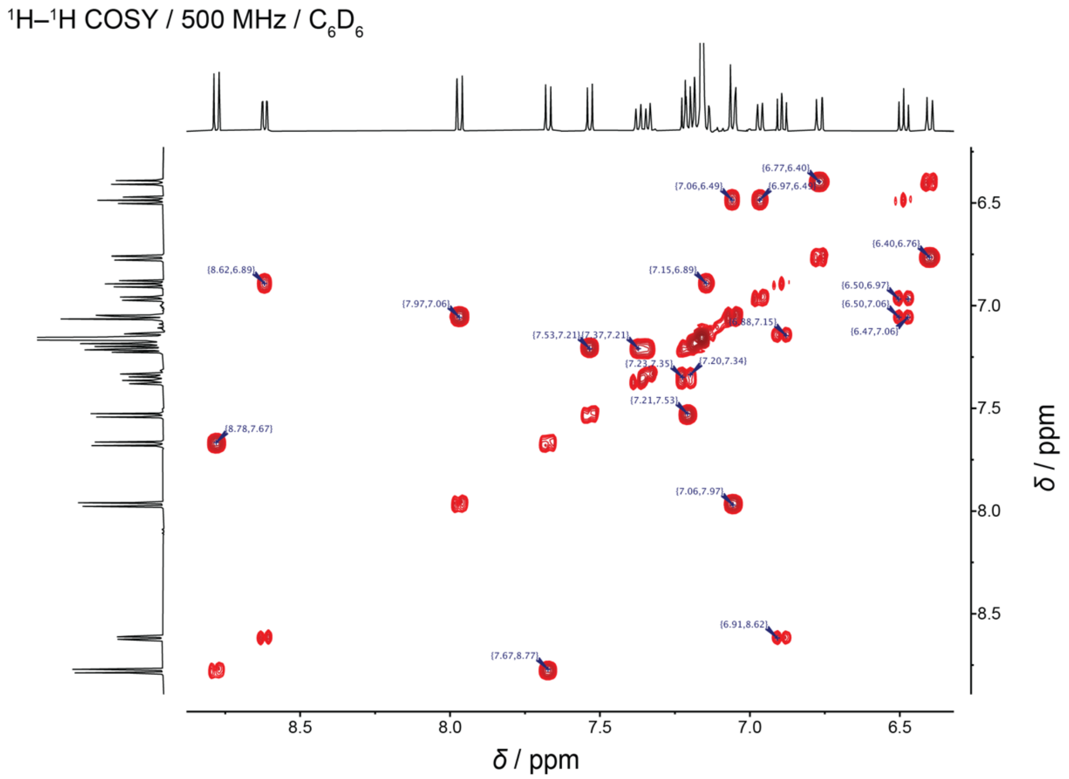

Figure S92. COSY NMR spectrum of *O*-c-**NC**.

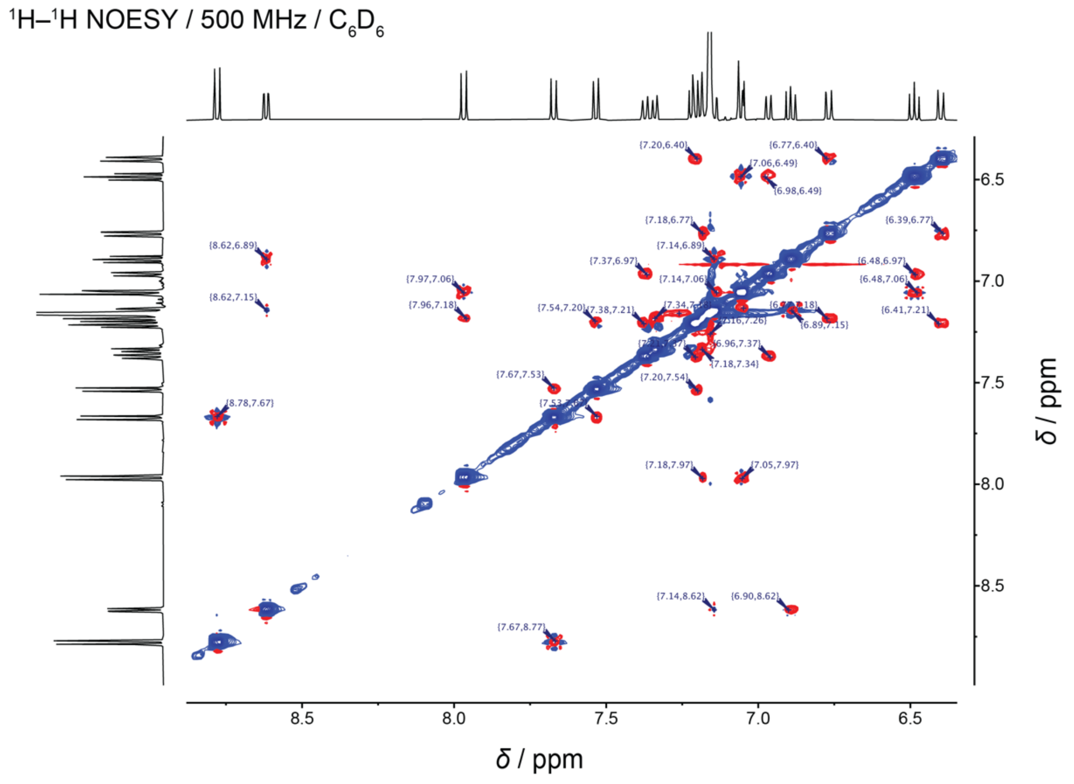

Figure S93. NOESY NMR spectrum of *O*-c-**NC**.

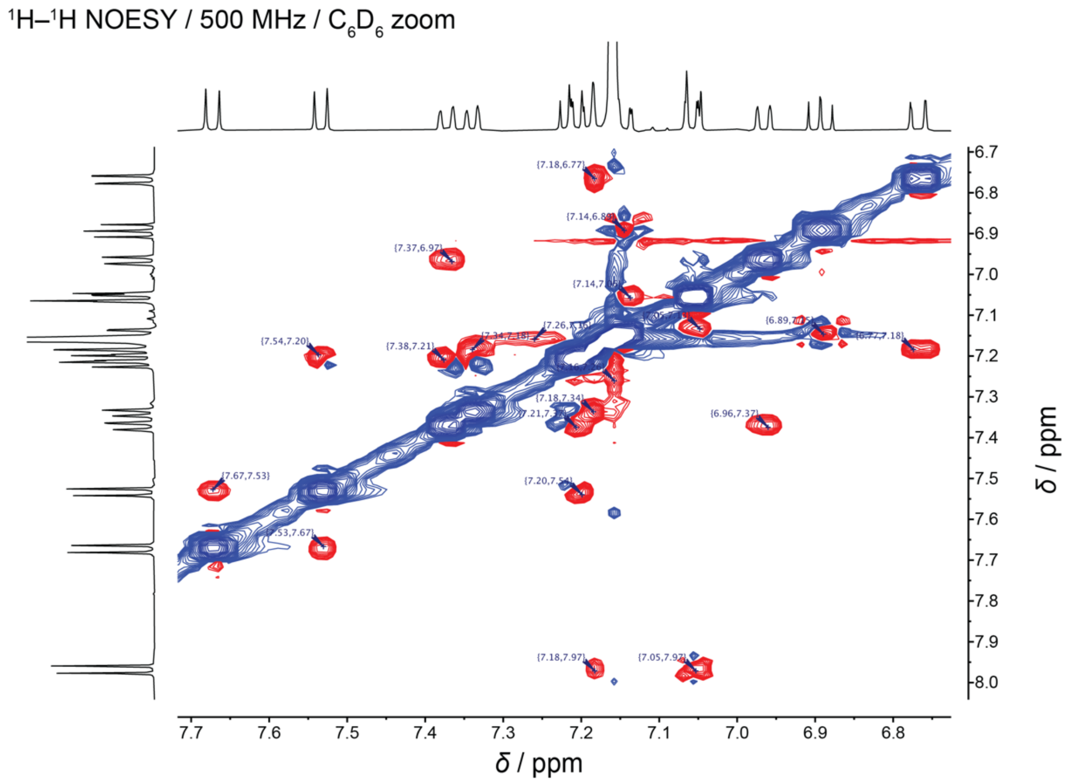

Figure S94. NOESY NMR spectrum of *O*-c-**NC**.

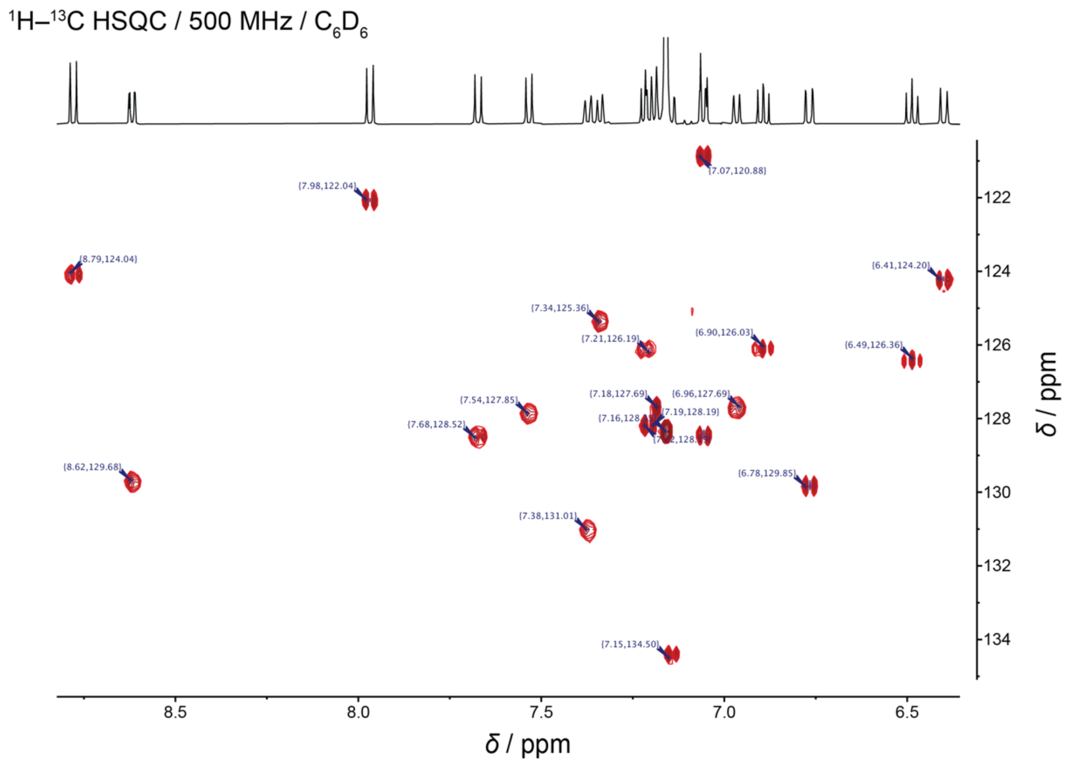

Figure S95. HSQC NMR spectrum of *O*-c-**NC**.

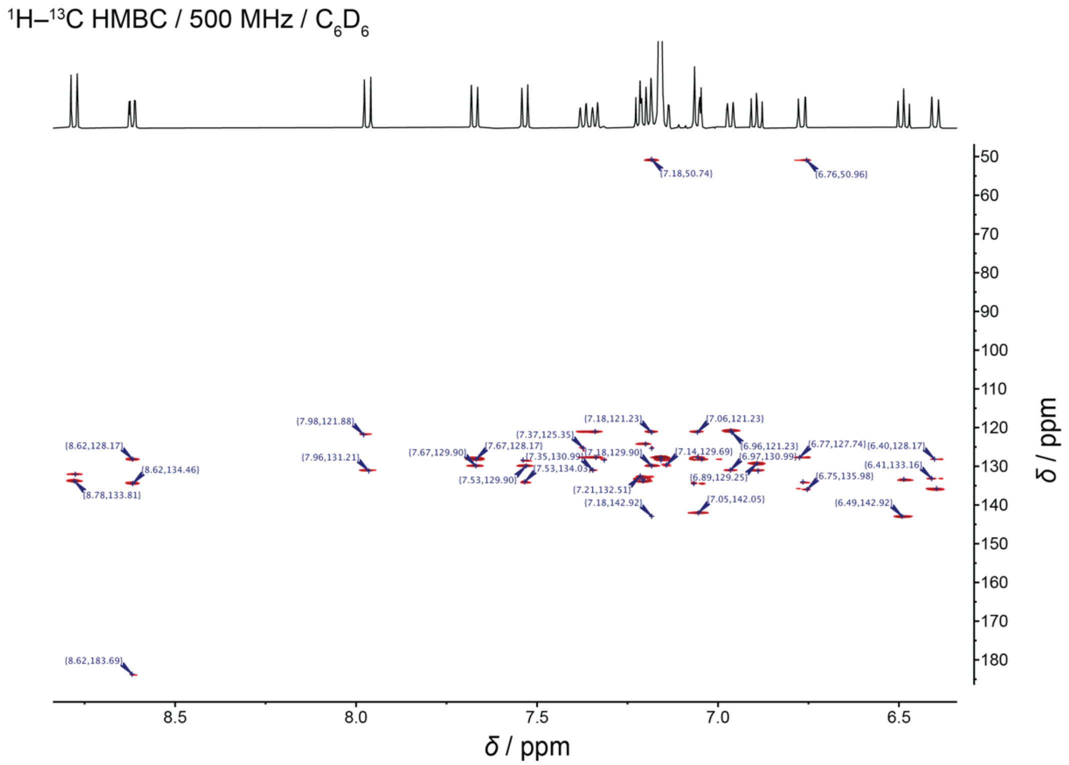

Figure S96. HMBC NMR spectrum of *O*-c-**NC**.

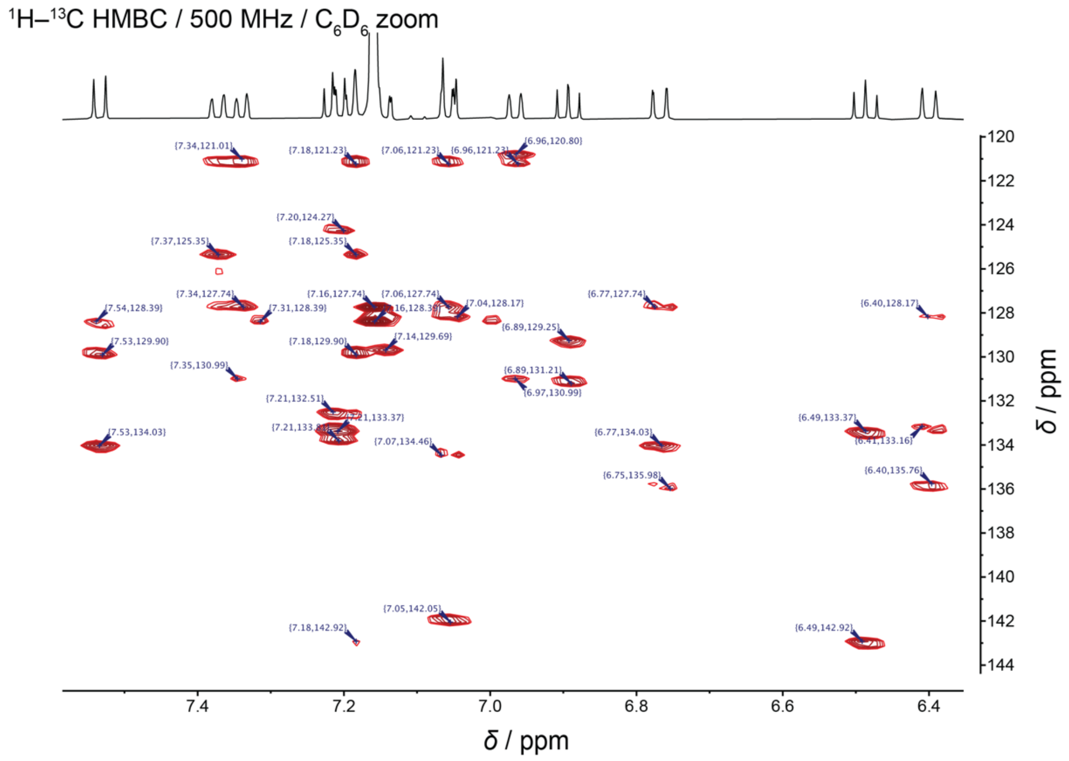

Figure S97. HMBC NMR spectrum of *O*-c-**NC**.

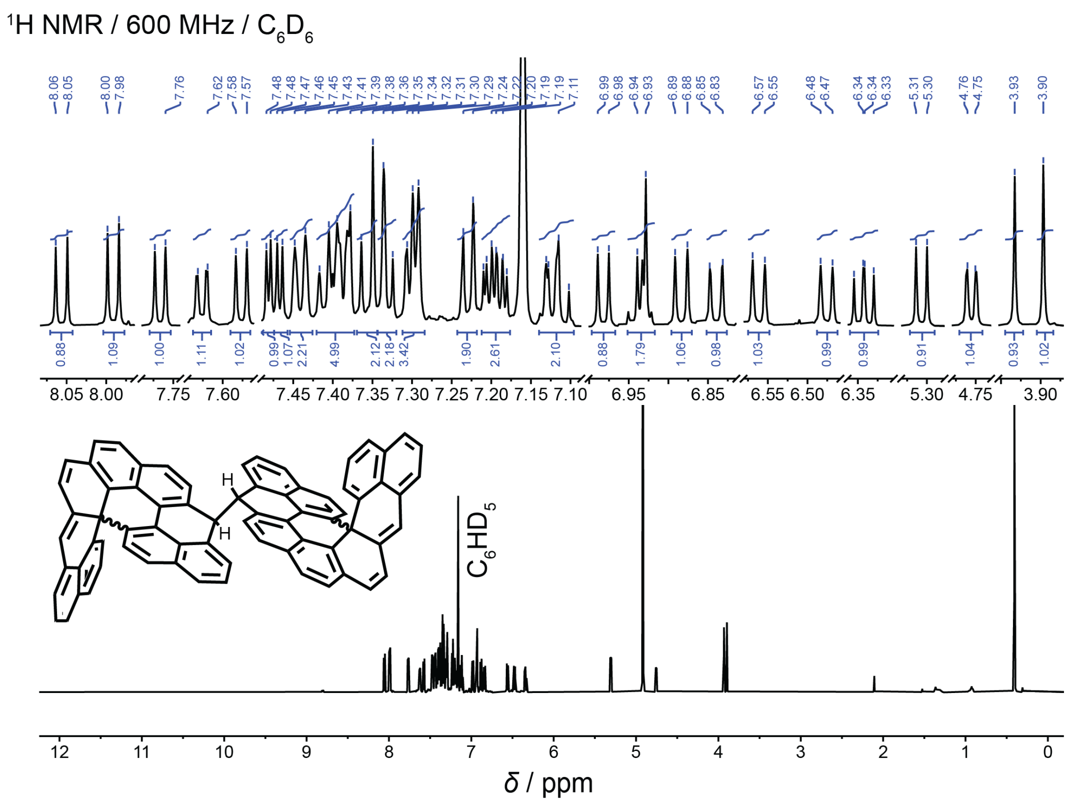

Figure S98. ^{1}H NMR spectrum of sym-(c-**NC**)$_2$.

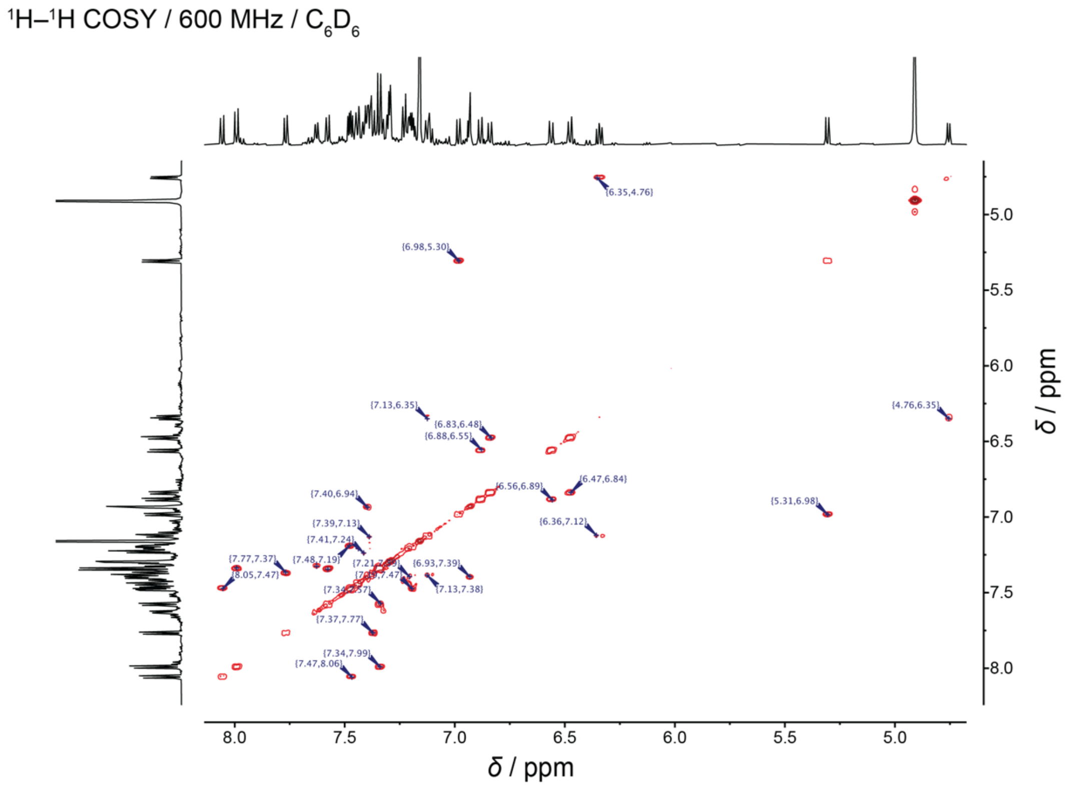

Figure S99. COSY NMR spectrum of sym-(c-**NC**)$_2$.

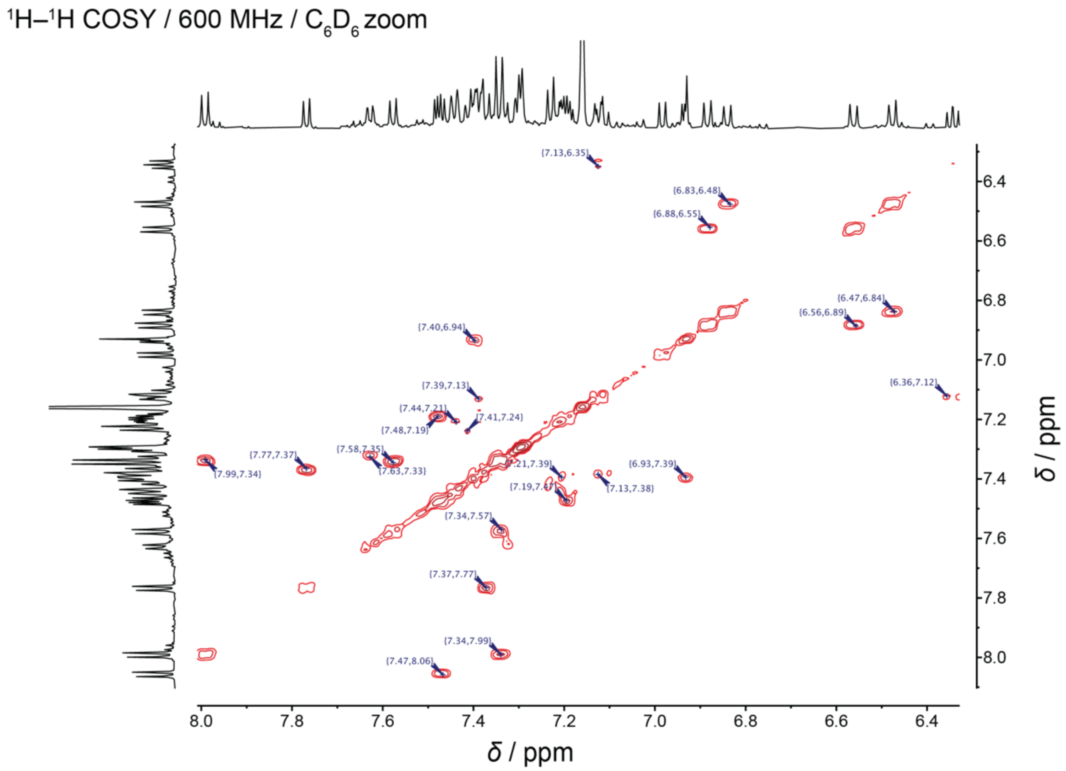

Figure S100. COSY NMR spectrum of sym-(c-**NC**)$_2$.

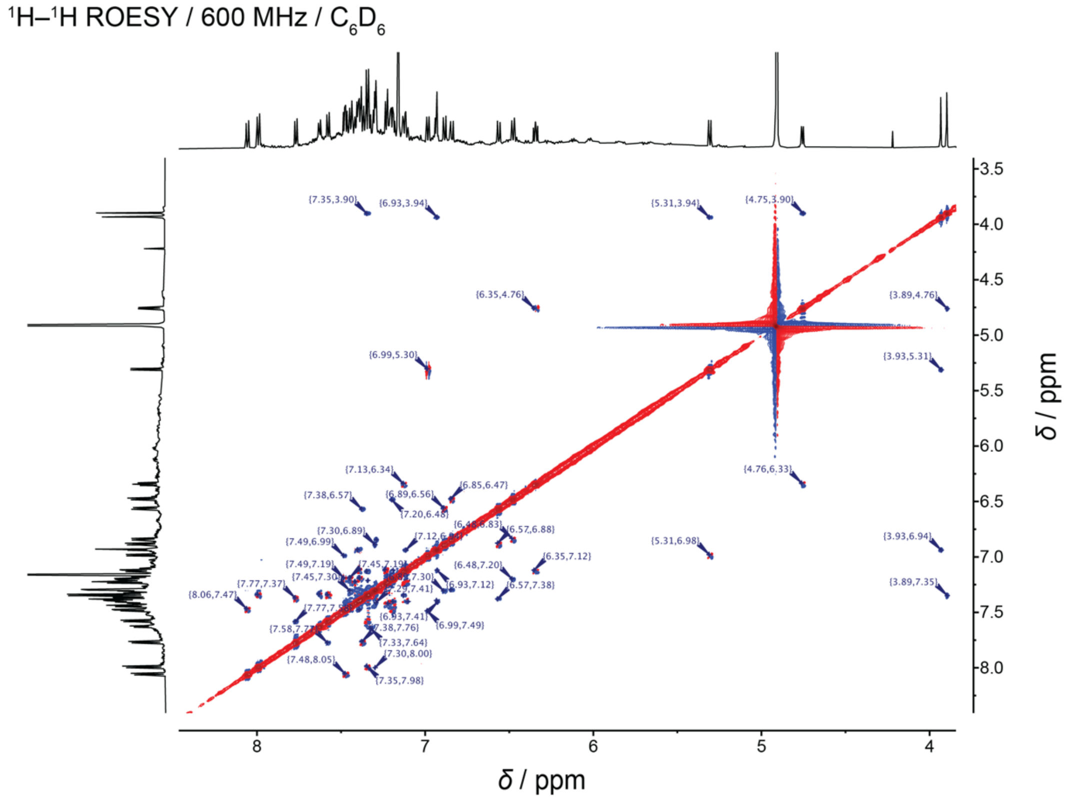

Figure S101. ROESY NMR spectrum of sym-(c-**NC**)$_2$.

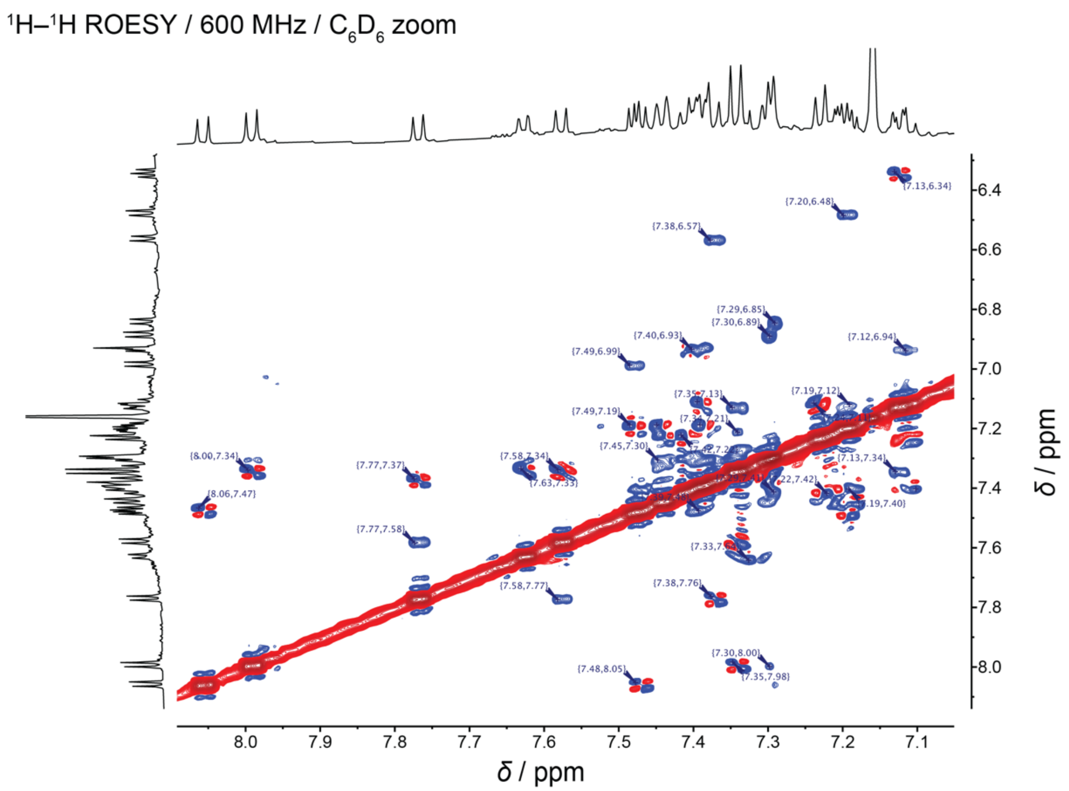

Figure S102. ROESY NMR spectrum of sym-(c-**NC**)2.

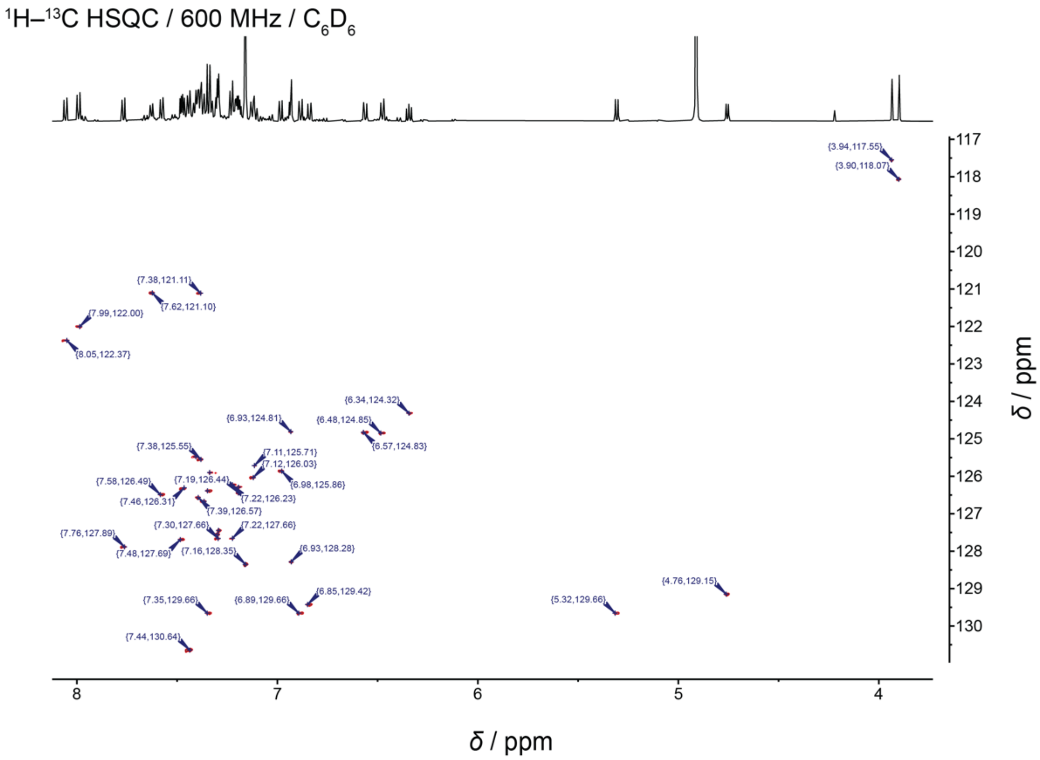

Figure S103. HSQC NMR spectrum of sym-(c-**NC**)2.

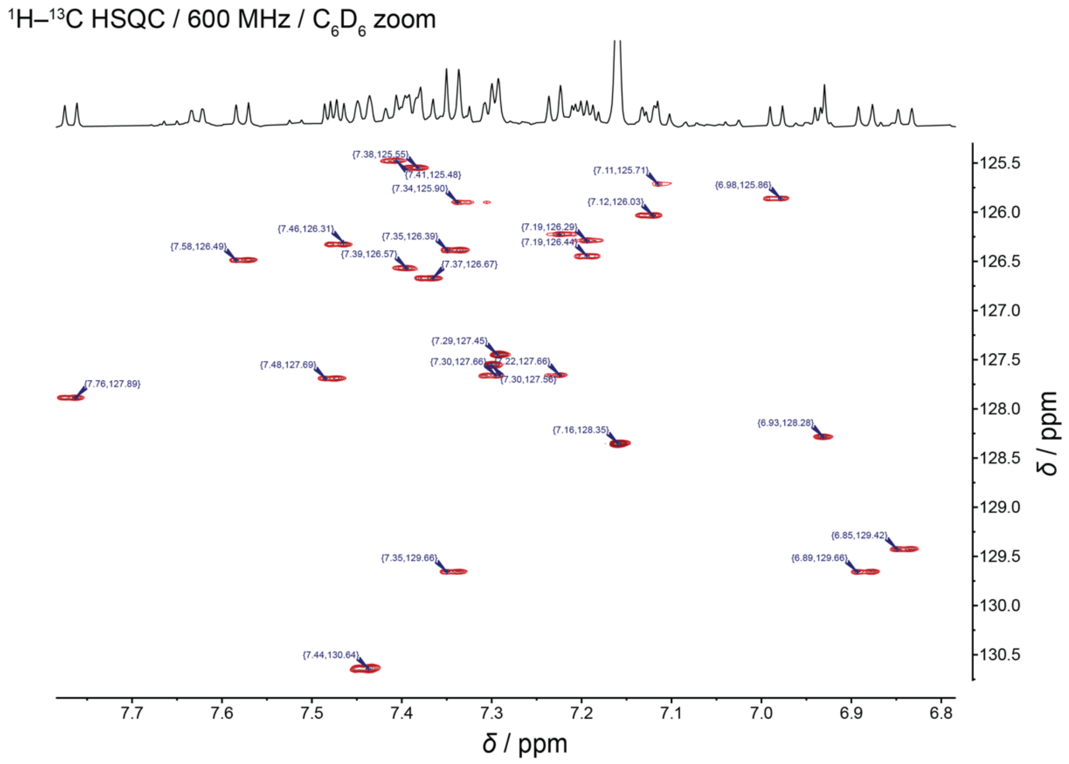

Figure S104. HSQC NMR spectrum of sym-(c-**NC**)$_2$.

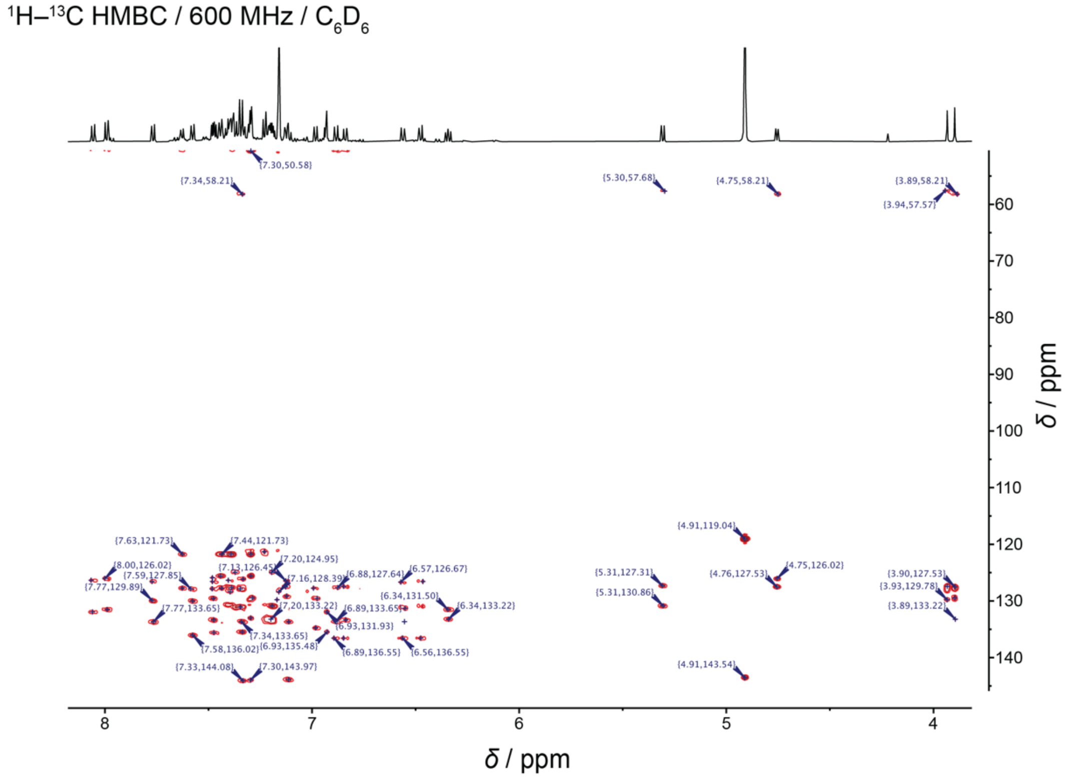

Figure S105. HMBC NMR spectrum of sym-(c-**NC**)$_2$.

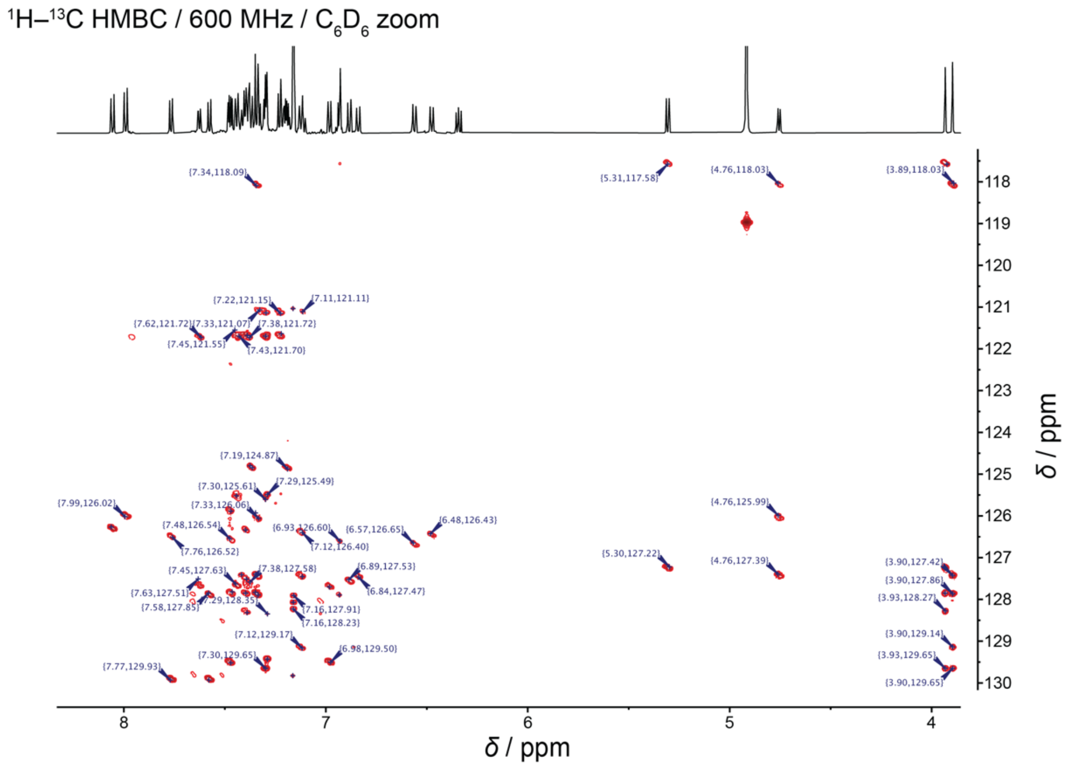

Figure S106. HMBC NMR spectrum of sym-(c-**NC**)$_2$.

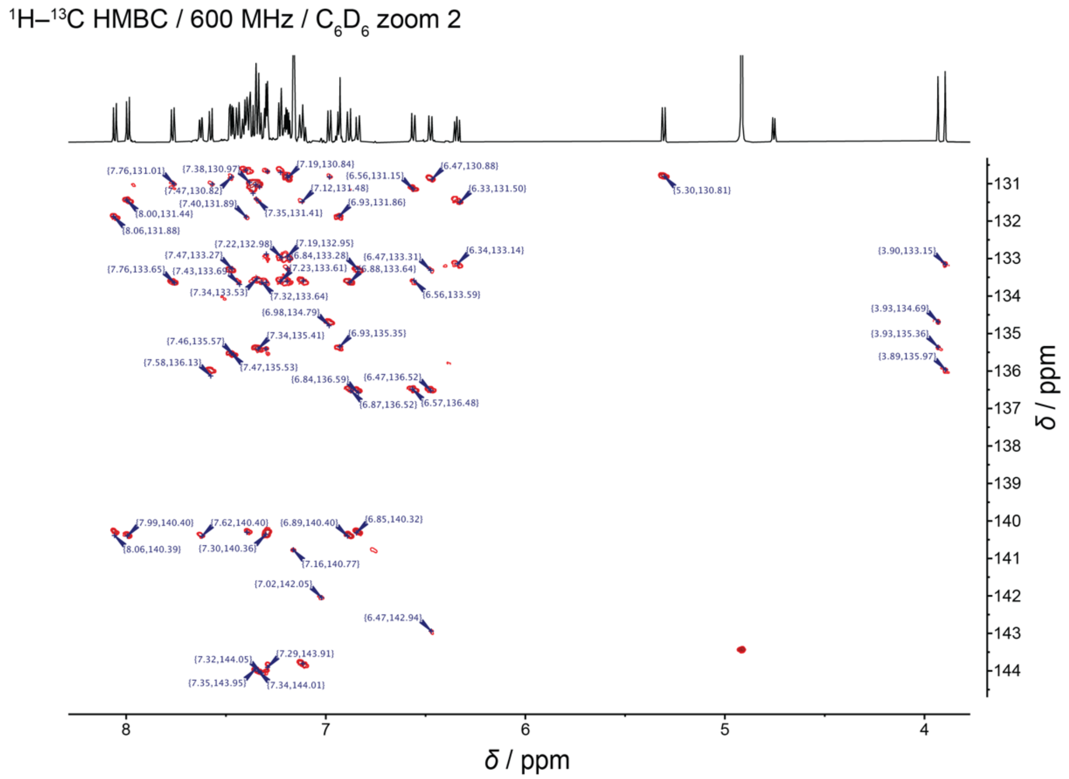

Figure S107. HMBC NMR spectrum of sym-(c-**NC**)$_2$.

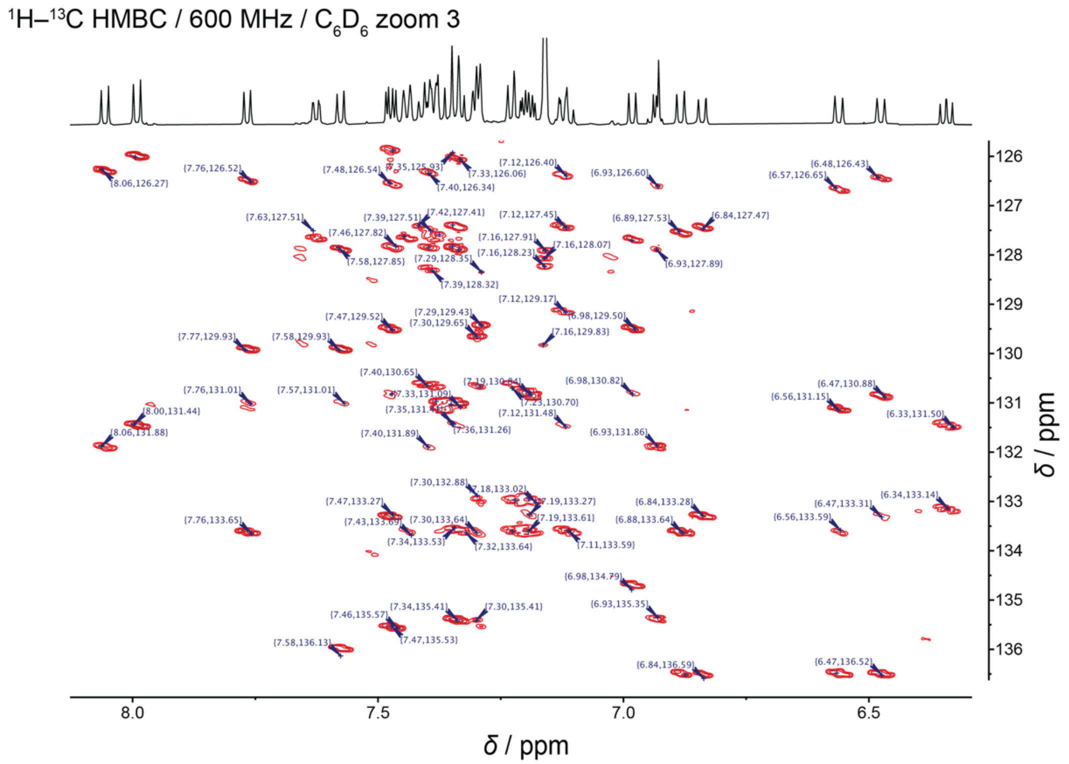

Figure S108. HMBC NMR spectrum of sym-(c-**NC**)$_2$.

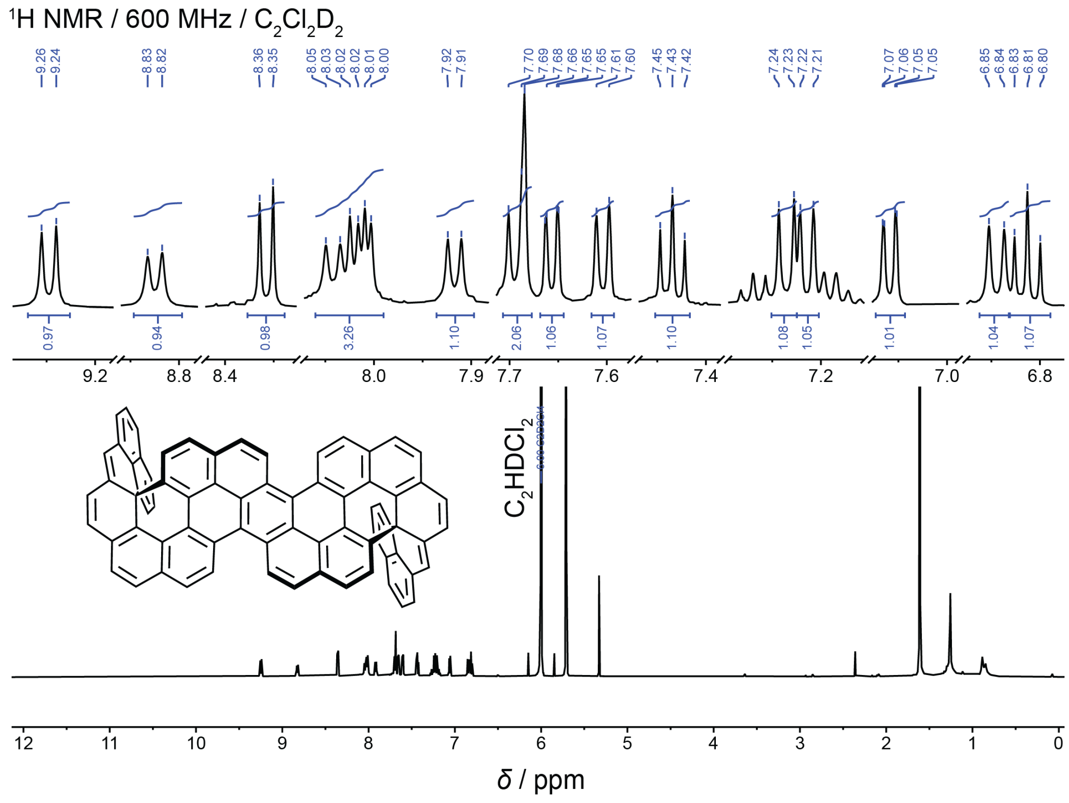

Figure S109. ^{1}H NMR spectrum of **HC**.

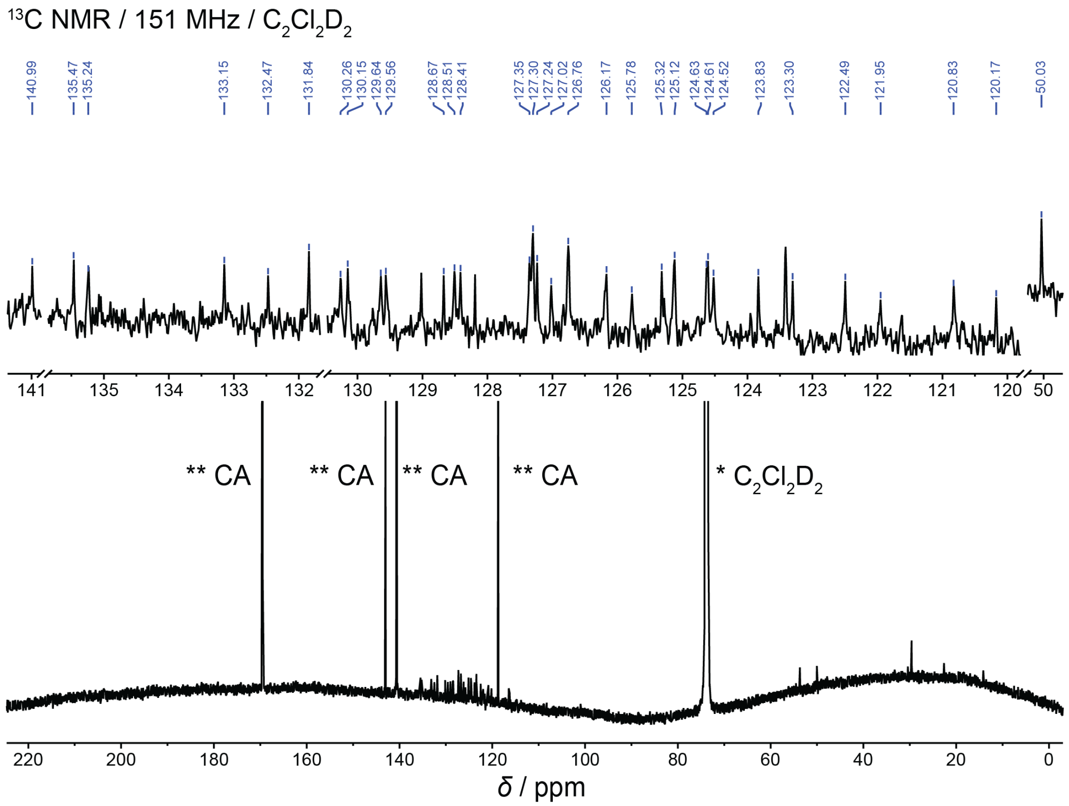

Figure S110. ^{13}C NMR spectrum of **HC**.

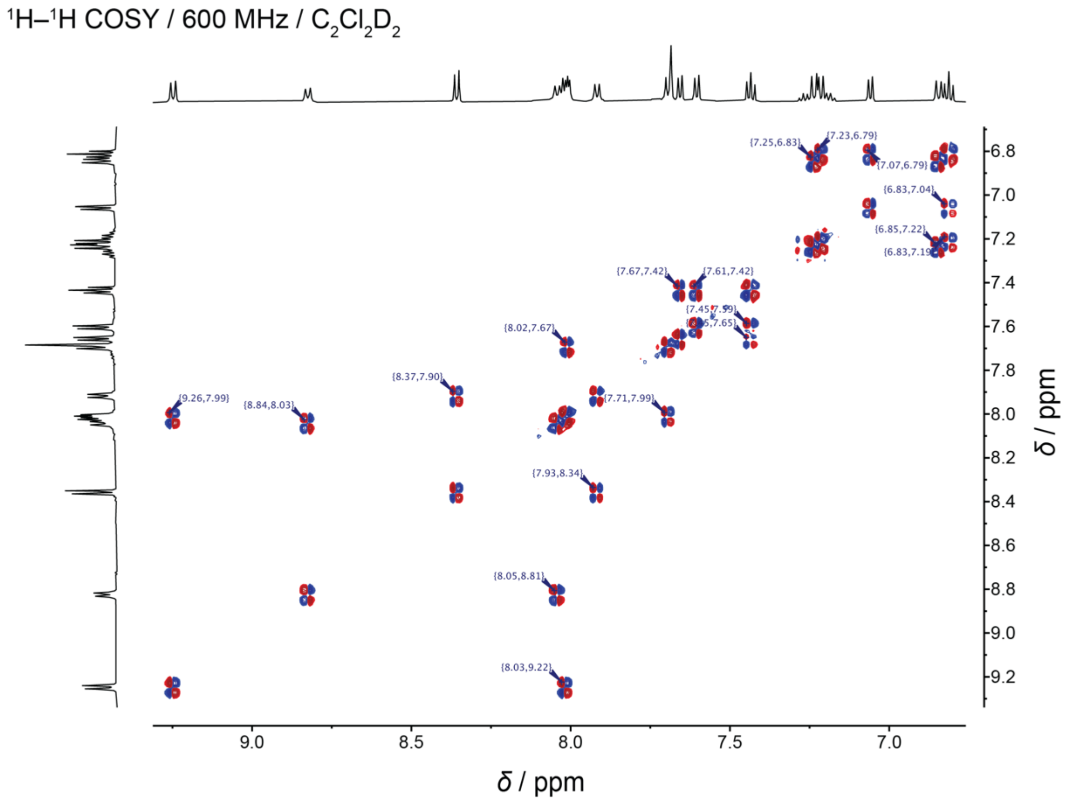

Figure S111. COSY NMR spectrum of **HC**.

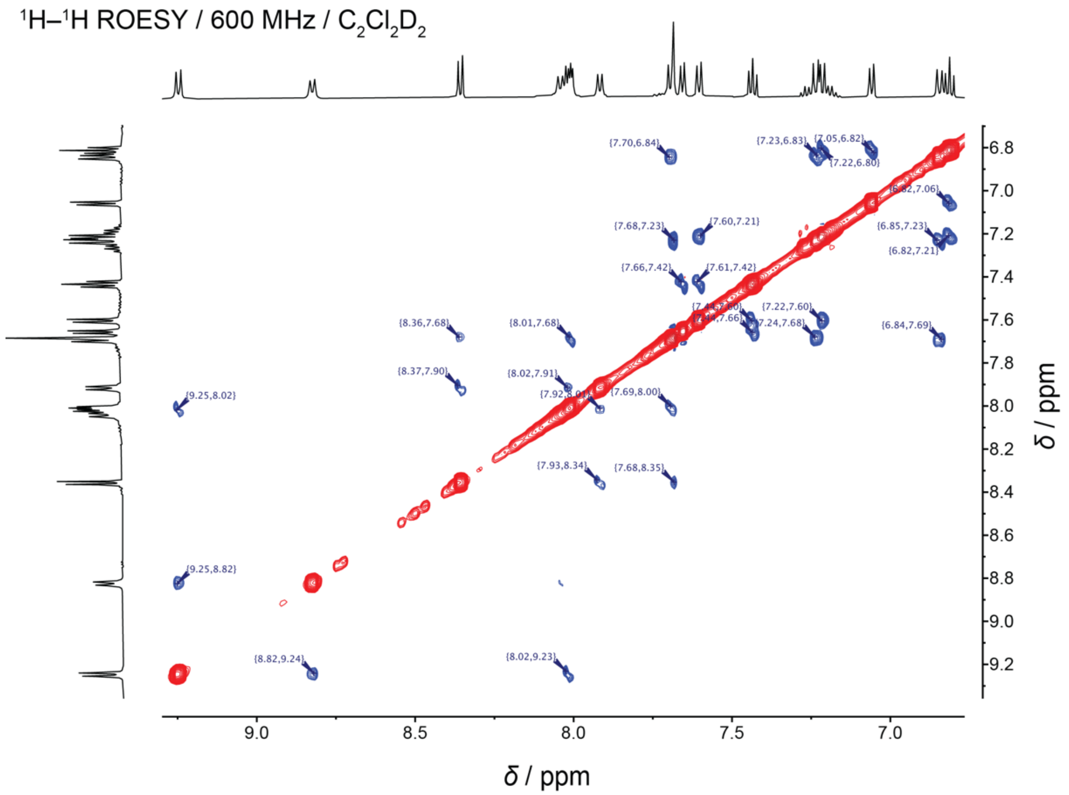

Figure S112. ROESY NMR spectrum of **HC**.

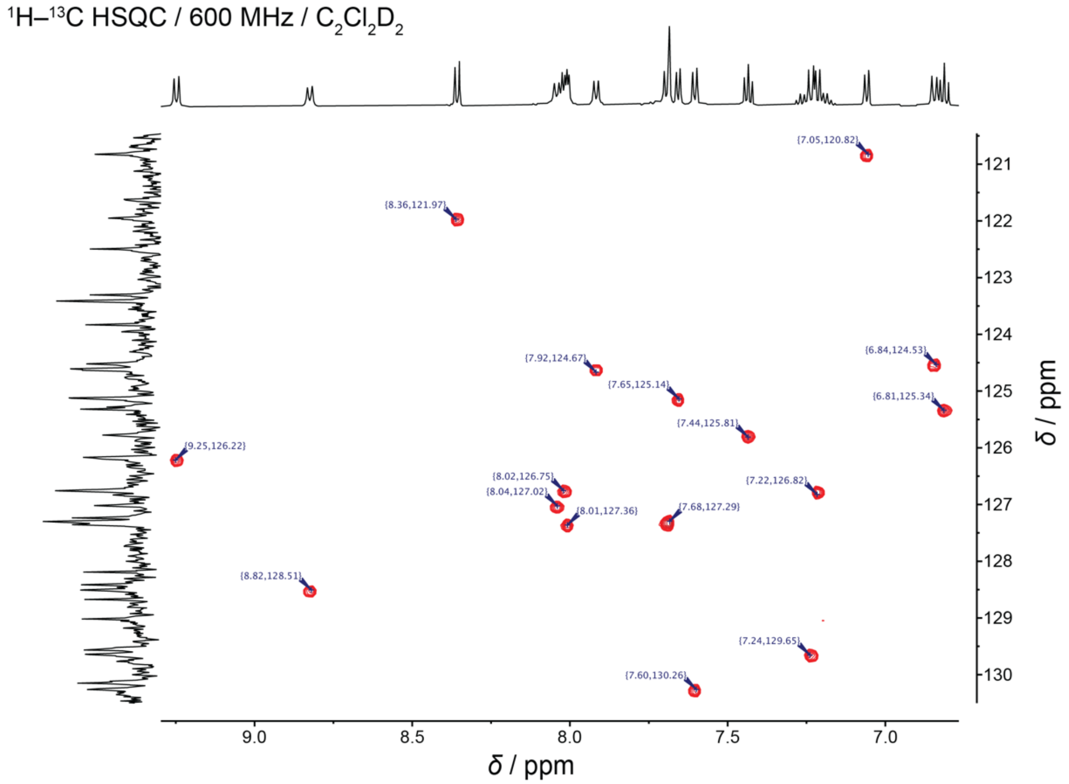

Figure S113. HSQC NMR spectrum of **HC**.

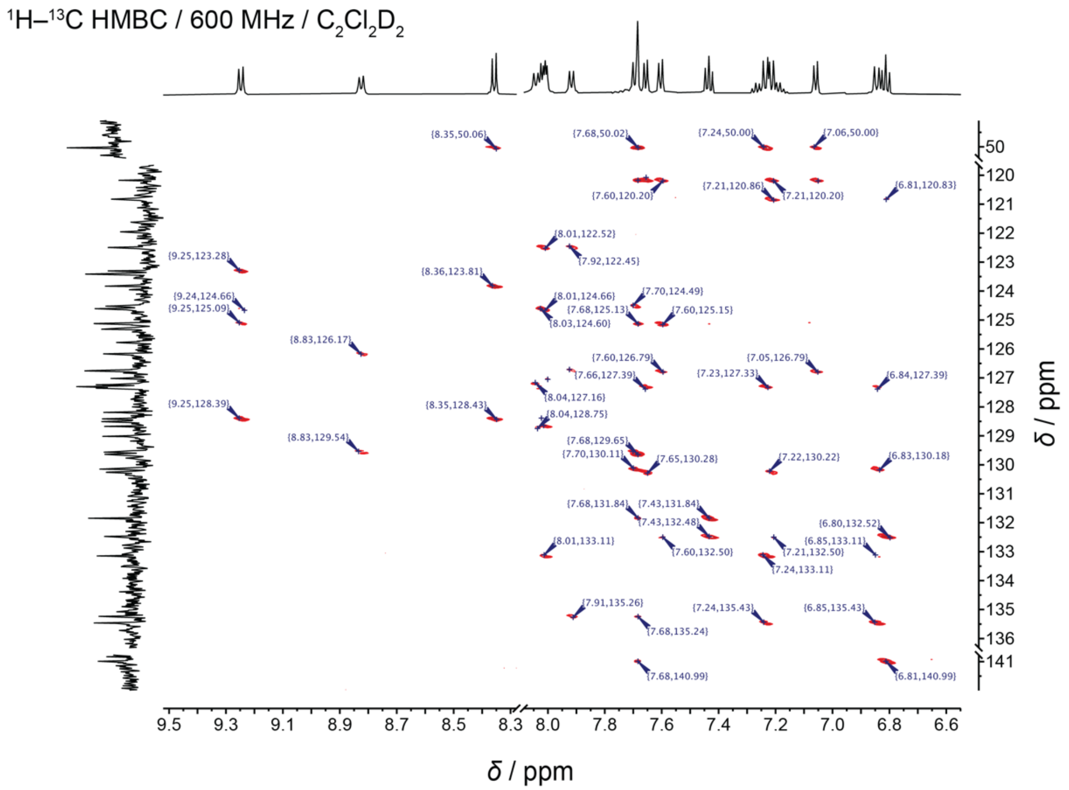

Figure S114. HMBC NMR spectrum of **HC**.

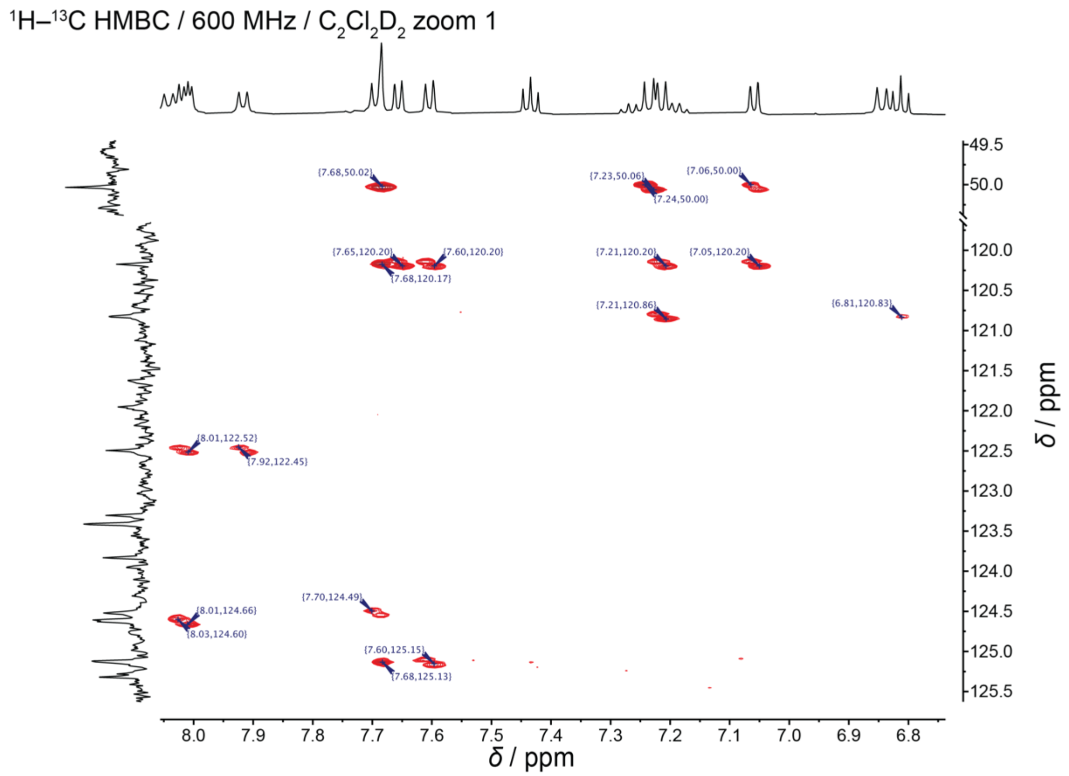

Figure S115. HMBC NMR spectrum of **HC**.

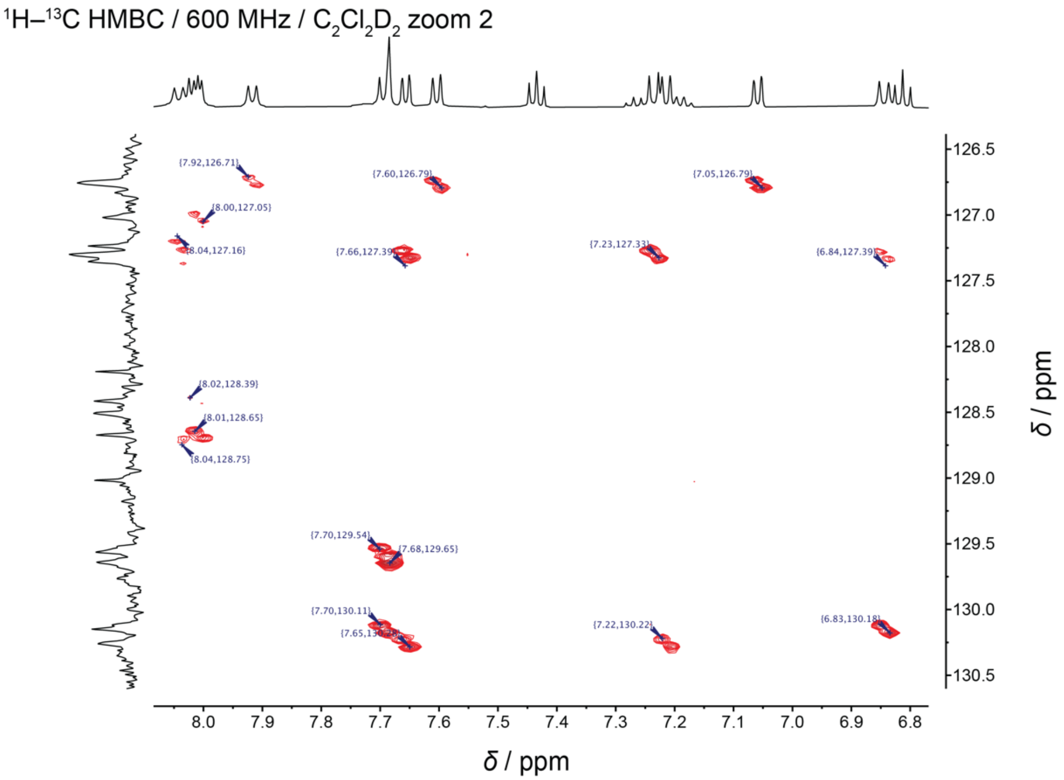

Figure S116. HMBC NMR spectrum of **HC**.

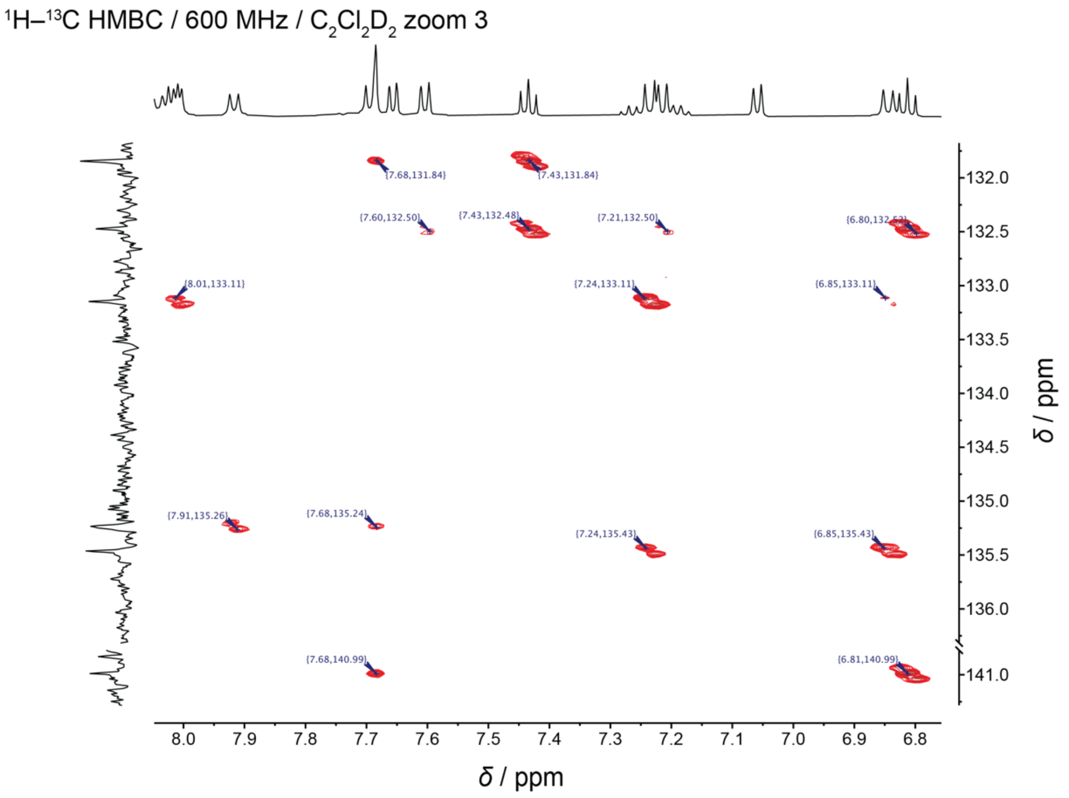

Figure S117. HMBC NMR spectrum of **HC**.

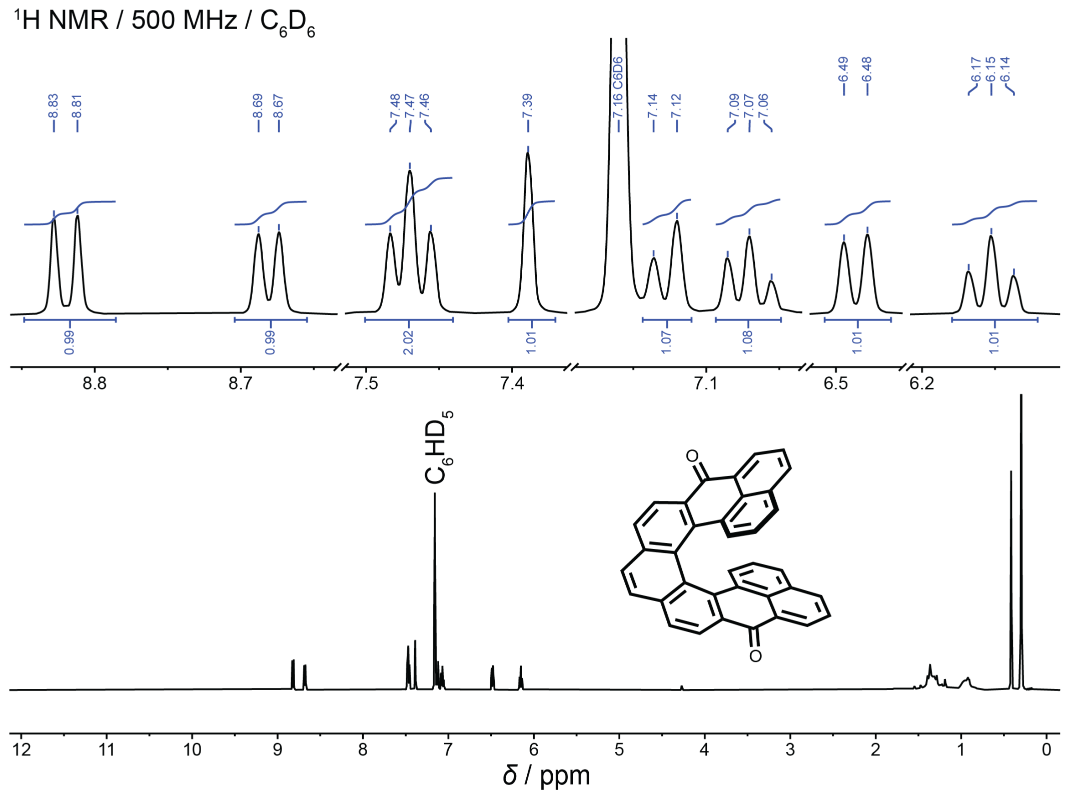

Figure S118. ^{1}H NMR spectrum of 2*O*-**NC**.

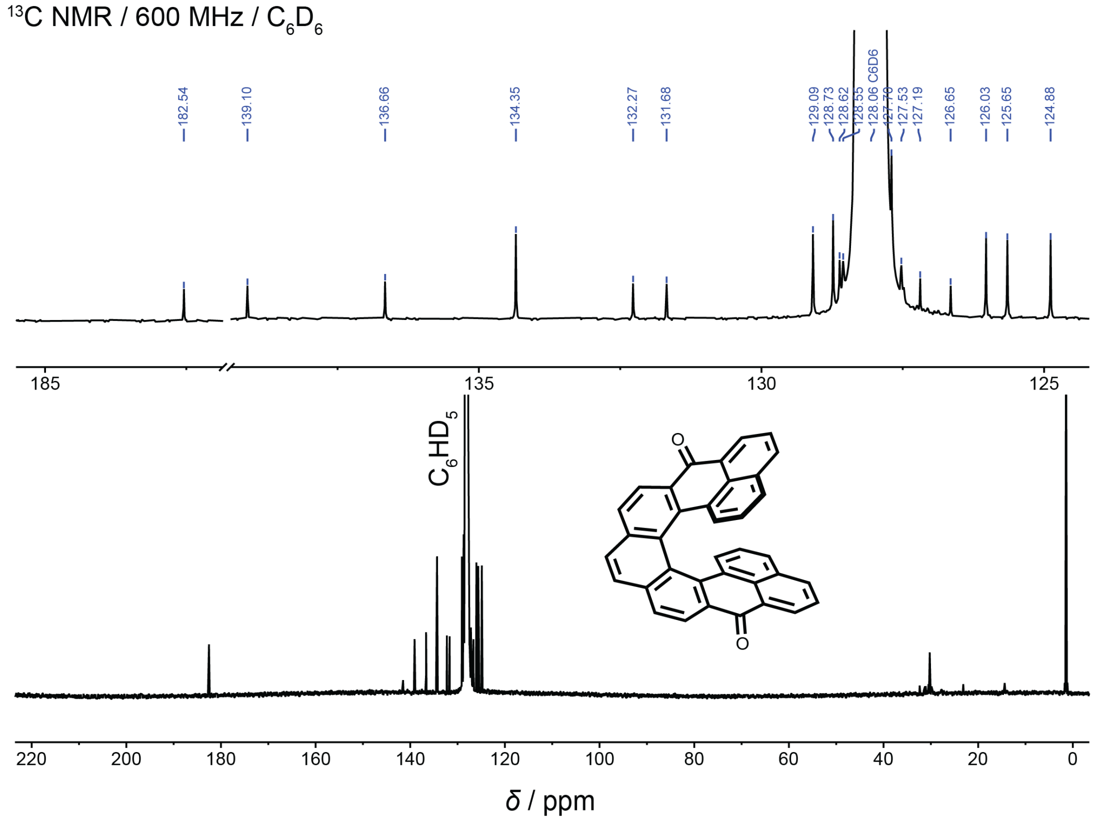

Figure S119. ^{13}C NMR spectrum of 2*O*-**NC**.

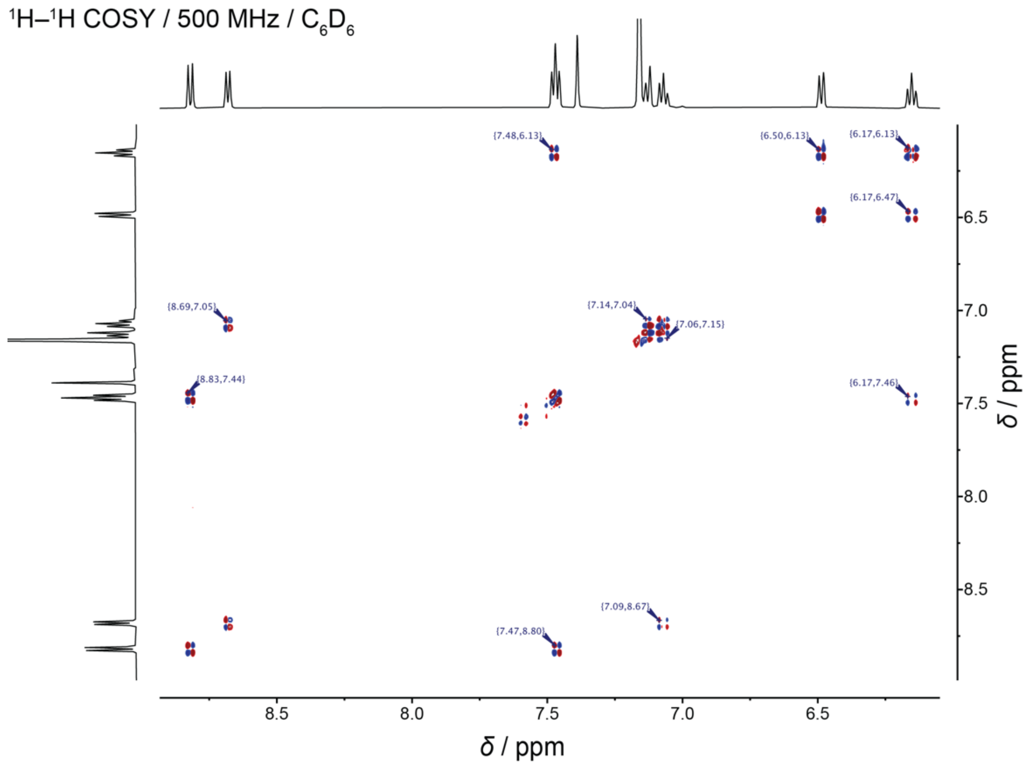

Figure S120. COSY NMR spectrum of 2*O*-**NC**.

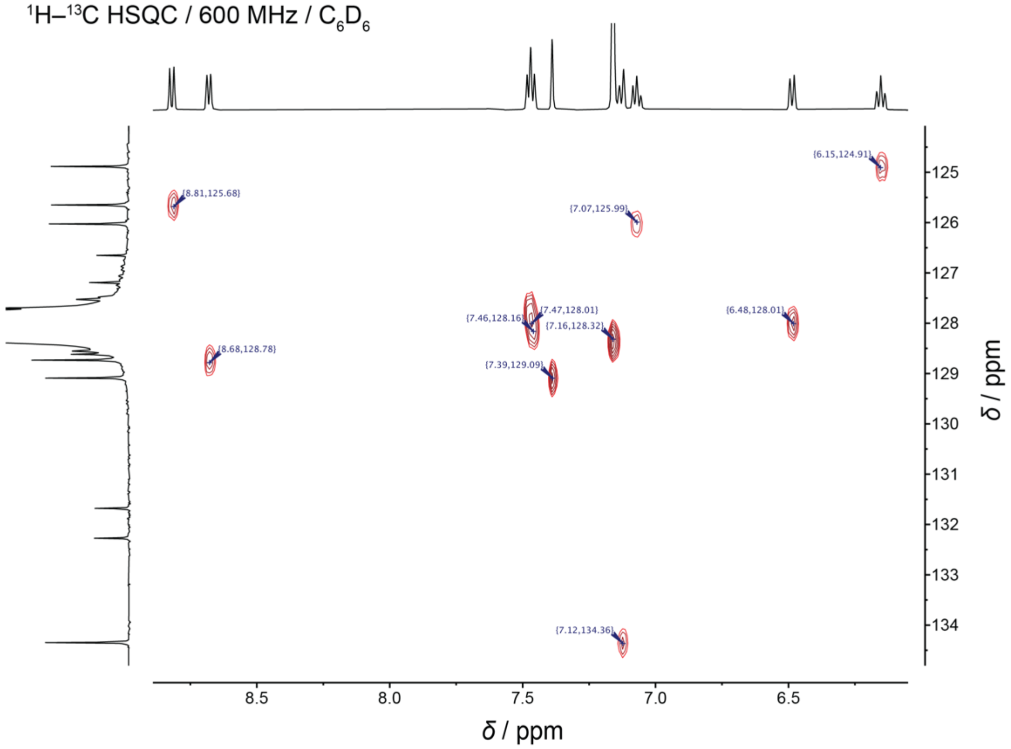

Figure S121. HSQC NMR spectrum of 2*O*-**NC**.

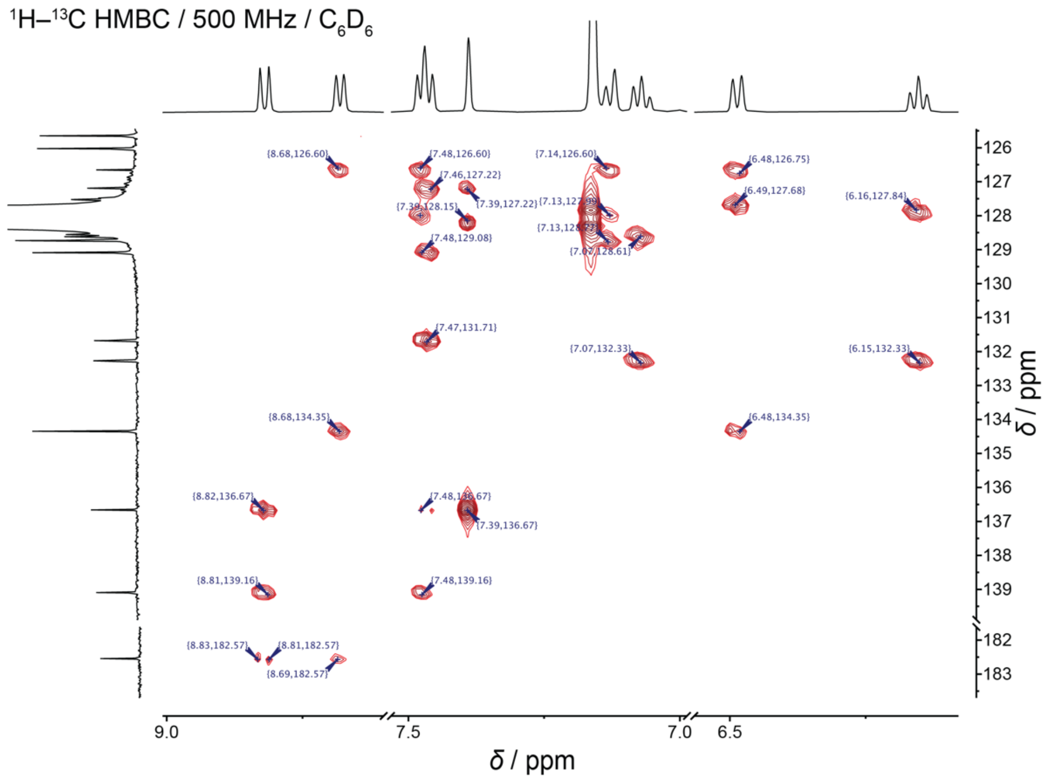

Figure S122. HMBC NMR spectrum of 2*O*-**NC**.

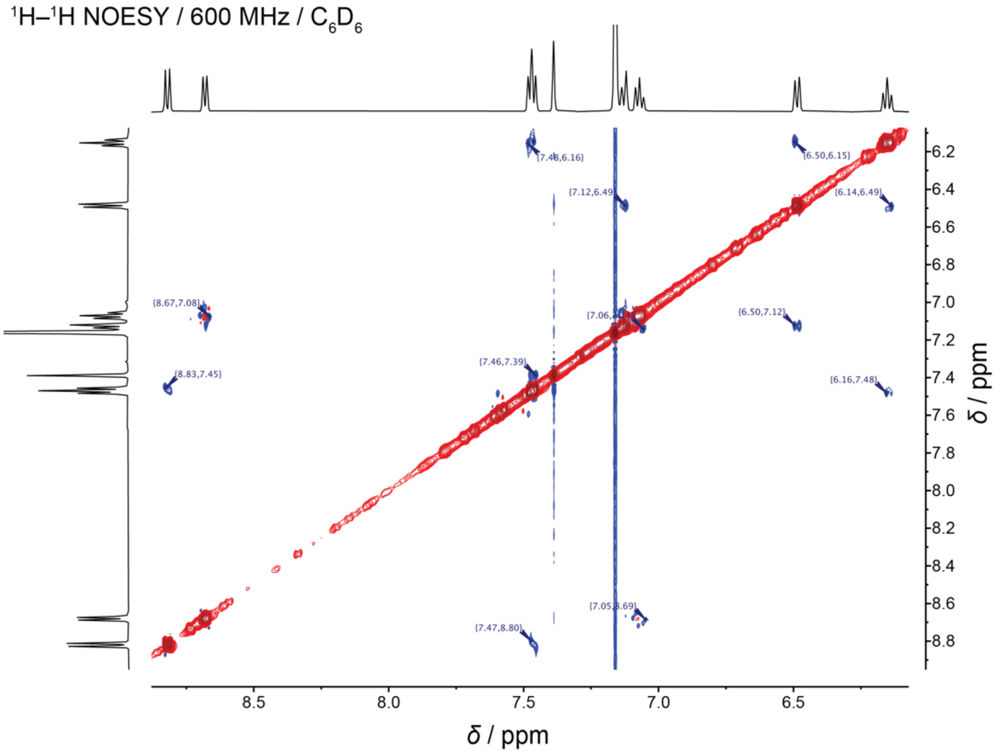

Figure S123. NOESY NMR spectrum of 2*O*-**NC**.

S10. DFT calculations

Table S3. Summary of total energies for the geometries optimized at the ωB97XD/Def2SVP level of DFT in the gas phase.

	Energies in kcal mol^{-1} including ZPVE correction	Relative energies in kcal mol^{-1} of isomeric compounds
H-**NC·**	–867619.78	16.11
c-*H*-**NC·**	–867603.67	0
**NC**-Singlet	–867239.58	6.78
**NC**-Triplet	–867238.04	8.32
c-**NC***	–867240.17	6.19
c-**NC**	–867246.36	0
c-**NC·**	–866867.01	–
sym-(*P*,*S*,*R*,*M*)-(c-**NC**)$_2$ (from XRD)	–1733766.99	0
sym-(*P*,*R*,*R*,*P*)-(c-**NC**)$_2$	–1733766.23	0.76
nonsym-(*P*,*S*,*S*,*P*)-(c-**NC**)$_2$	–1733764.56	2.43
nonsym-(*P*,*R*,*R*,*M*)-(c-**NC**)$_2$	–1733760.04	6.95
nonsym-(*P*,*R*,*R*,*P*)-(c-**NC**)$_2$	–1733752.02	15.0
nonsym-(*P*,*R*,*S*,*M*)-(c-**NC**)$_2$	–1733757.40	9.59
nonsym-(*P*,*R*,*S*,*P*)-(c-**NC**)$_2$	–1733764.56	2.43
nonsym-(*P*,*S*,*R*,*M*)-(c-**NC**)$_2$	–1733762.687	4.30
nonsym-(*P*,*S*,*R*,*P*)-(c-**NC**)$_2$	–1733759.896	7.09
nonsym-(*P*,*S*,*S*,*M*)-(c-**NC**)$_2$	–1733757.162	9.83
(*P*,*P*)-**HC**	–1732295.28	0
(*P*,*M*)-**HC**	–1732291.98	3.30

Mulliken charges and spin densities (c-NC·):

		1	2
1	C	0.015828	-0.099434
2	C	0.006083	0.093856
3	C	0.010649	0.102235
4	C	0.011478	-0.064374
5	C	-0.037934	0.085372
6	C	-0.028662	-0.068072
7	C	-0.021046	-0.102092
8	C	0.040766	0.273980
9	C	0.023778	-0.181835
10	C	-0.021257	0.102635
11	C	-0.091848	0.417608
12	C	0.053070	-0.206737
13	C	0.038828	0.122147
14	C	0.058742	-0.184580
15	C	0.041199	-0.181333
16	C	-0.037238	0.288600
17	C	-0.021108	-0.174477
18	C	0.035773	0.309563
19	C	-0.144137	-0.031326
20	C	0.047212	0.024293
21	C	0.022779	-0.037779
22	C	-0.036481	0.035022
23	C	0.089422	0.019943
24	C	0.039856	-0.003542
25	C	0.045094	0.011890
26	C	-0.076713	-0.027686
27	C	0.031907	0.004109
28	C	-0.039244	-0.009807
29	C	0.002744	0.006453
30	C	-0.070874	-0.011875
31	C	-0.032368	0.000947
32	C	0.003865	0.002126
33	C	-0.052655	0.000158
34	C	-0.046325	0.368099
35	C	0.003456	-0.206338
36	C	-0.050211	0.368877
37	H	-0.006318	-0.017845
38	H	0.006722	-0.003686
39	H	0.005566	0.002679
40	H	0.007456	0.004265
41	H	0.007570	-0.004248
42	H	0.003797	-0.012554
43	H	0.012617	0.006472
44	H	0.010392	0.001721
45	H	0.010686	-0.001428
46	H	0.005483	0.001298
47	H	0.007847	0.000478
48	H	0.019199	-0.000238
49	H	0.006501	0.000513
50	H	0.005709	-0.000045
51	H	0.018630	-0.000006
52	H	0.027980	0.000589
53	H	0.007973	-0.016092
54	H	0.020230	0.007529
55	H	0.007532	-0.016032

Sum of Mulliken charges = 0.00000 1.00000

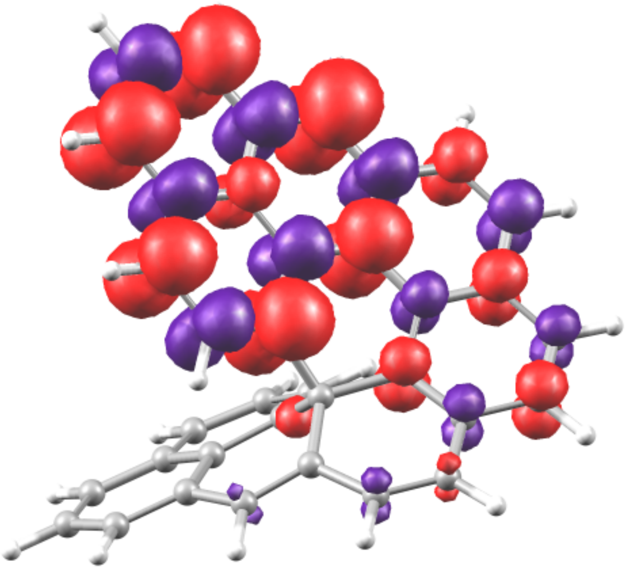

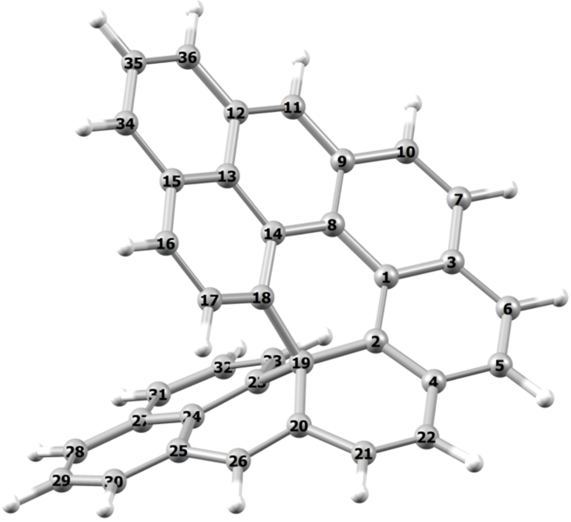

Isotropic Fermi contact couplings:

Atom		a.u.	MegaHertz	Gauss	10(-4) cm-1
1	C(13)	-0.00970	-10.90088	-3.88970	-3.63614
2	C(13)	0.00979	11.00063	3.92530	3.66941
3	C(13)	0.00694	7.80617	2.78543	2.60386
4	C(13)	-0.00456	-5.12819	-1.82987	-1.71058
5	C(13)	0.00514	5.77583	2.06096	1.92661
6	C(13)	-0.00471	-5.29554	-1.88958	-1.76640
7	C(13)	-0.00611	-6.86929	-2.45113	-2.29135
8	C(13)	0.01681	18.89260	6.74135	6.30189
9	C(13)	-0.01803	-20.27275	-7.23382	-6.76226
10	C(13)	0.00639	7.18513	2.56383	2.39670
11	C(13)	0.02493	28.02674	10.00064	9.34871
12	C(13)	-0.02082	-23.41060	-8.35348	-7.80894
13	C(13)	0.00938	10.54494	3.76270	3.51741
14	C(13)	-0.01581	-17.77342	-6.34200	-5.92858
15	C(13)	-0.01775	-19.95977	-7.12214	-6.65786
16	C(13)	0.01770	19.89758	7.09995	6.63712
17	C(13)	-0.01600	-17.99174	-6.41990	-6.00140
18	C(13)	0.02267	25.48073	9.09216	8.49946
19	C(13)	-0.00846	-9.51028	-3.39351	-3.17229
20	C(13)	0.00297	3.34178	1.19243	1.11470
21	C(13)	-0.00165	-1.85968	-0.66358	-0.62032
22	C(13)	0.00183	2.06262	0.73599	0.68802
23	C(13)	0.02138	24.04080	8.57836	8.01915
24	C(13)	-0.00042	-0.47349	-0.16895	-0.15794
25	C(13)	0.00038	0.43216	0.15420	0.14415
26	C(13)	-0.00110	-1.23746	-0.44156	-0.41277
27	C(13)	0.00063	0.70944	0.25315	0.23664
28	C(13)	-0.00024	-0.27470	-0.09802	-0.09163
29	C(13)	0.00038	0.42566	0.15189	0.14199
30	C(13)	-0.00051	-0.57441	-0.20496	-0.19160
31	C(13)	0.00006	0.06259	0.02233	0.02088
32	C(13)	0.00052	0.58134	0.20744	0.19392
33	C(13)	-0.00001	-0.01534	-0.00547	-0.00512
34	C(13)	0.02274	25.56452	9.12206	8.52741
35	C(13)	-0.01974	-22.18866	-7.91747	-7.40134
36	C(13)	0.02283	25.66656	9.15847	8.56144
37	H(1)	-0.00483	-21.58936	-7.70362	-7.20143
38	H(1)	-0.00090	-4.00647	-1.42961	-1.33642
39	H(1)	0.00048	2.13549	0.76200	0.71232
40	H(1)	0.00078	3.50199	1.24960	1.16814
41	H(1)	-0.00087	-3.90113	-1.39202	-1.30128
42	H(1)	-0.00332	-14.82755	-5.29084	-4.94594
43	H(1)	0.00139	6.22214	2.22021	2.07548
44	H(1)	0.00031	1.36751	0.48796	0.45615
45	H(1)	-0.00020	-0.90353	-0.32240	-0.30138
46	H(1)	0.00020	0.89058	0.31778	0.29707
47	H(1)	0.00009	0.41450	0.14790	0.13826
48	H(1)	-0.00004	-0.18038	-0.06436	-0.06017
49	H(1)	0.00008	0.36220	0.12924	0.12082
50	H(1)	-0.00003	-0.12804	-0.04569	-0.04271
51	H(1)	0.00004	0.19955	0.07120	0.06656
52	H(1)	0.00030	1.33125	0.47502	0.44406
53	H(1)	-0.00441	-19.69262	-7.02682	-6.56875
54	H(1)	0.00157	7.01289	2.50237	2.33925
55	H(1)	-0.00435	-19.45104	-6.94062	-6.48817

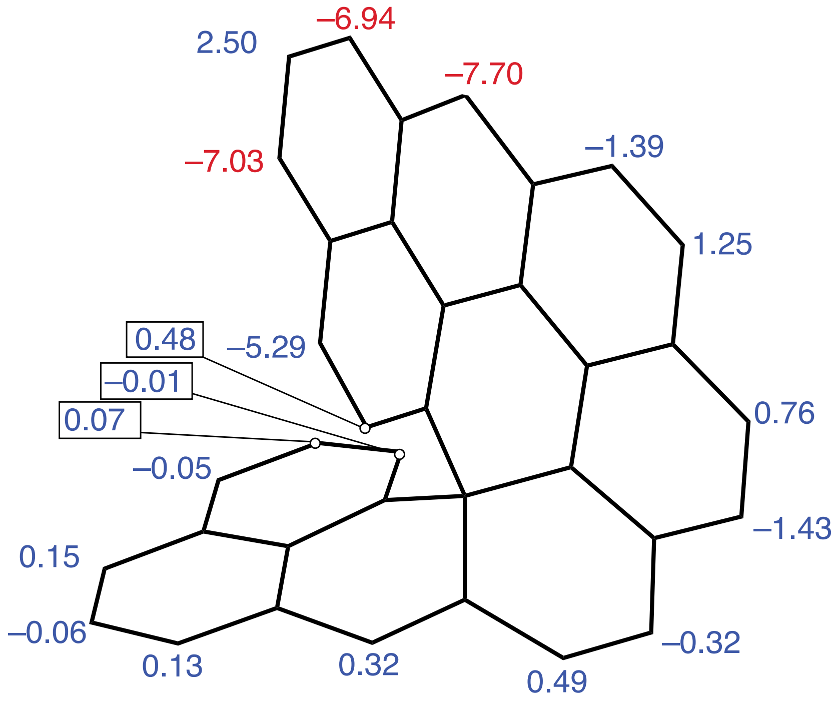

Figure S124. Calculated proton hyperfine coupling constants (in gauss) for c-**NC·**. π-Bonds are omitted for clarity. Main contributions (> 6 gauss) are depicted in red, smaller contributions (< 6 gauss) in blue.

Mulliken charges and spin densities (*H*-NC·):

		1	2
1	C	-0.027748	-0.134129
2	C	-0.024725	0.080552
3	C	0.014722	0.132636
4	C	0.021673	-0.052025
5	C	-0.013352	0.099699
6	C	-0.018877	-0.073728
7	C	-0.021583	-0.116747
8	C	0.007081	0.300396
9	C	0.047108	-0.193540
10	C	-0.015871	0.125038
11	C	-0.105925	0.451837
12	C	0.095248	-0.217682
13	C	0.028067	0.125725
14	C	0.033599	-0.183880
15	C	0.053786	-0.170594
16	C	-0.035511	0.256882
17	C	0.008558	-0.158121
18	C	-0.026647	0.272425
19	C	0.036201	-0.043716
20	C	-0.132748	0.034016
21	C	-0.027759	-0.034951
22	C	-0.029587	0.033831
23	C	0.007941	0.021539
24	C	0.044592	-0.010439
25	C	-0.077829	0.007577
26	C	0.182214	-0.003225
27	C	0.033990	0.009101
28	C	-0.042605	-0.006770
29	C	-0.000885	0.007240
30	C	-0.081874	-0.007062
31	C	-0.028059	-0.012678
32	C	-0.002251	0.010754
33	C	-0.026305	-0.017820
34	C	-0.055939	0.360798
35	C	0.004276	-0.203949
36	C	-0.061018	0.369930
37	H	0.007603	-0.004439
38	H	0.007610	0.002834
39	H	0.007382	0.005010
40	H	0.008051	-0.005181
41	H	-0.006841	-0.019451
42	H	0.002041	-0.011047
43	H	0.013418	0.006034
44	H	0.015410	-0.011585
45	H	0.005479	0.001493
46	H	0.007185	-0.001404
47	H	0.067567	0.001705
48	H	0.029892	0.000613
49	H	0.001561	0.000170
50	H	0.014869	-0.000304
51	H	-0.001243	0.000307
52	H	0.002248	0.000399
53	H	0.015250	-0.000811
54	H	0.014508	0.001300
55	H	0.004621	-0.015866
56	H	0.017143	0.007449
57	H	0.004287	-0.016145

Sum of Mulliken charges = 0.00000 1.00000

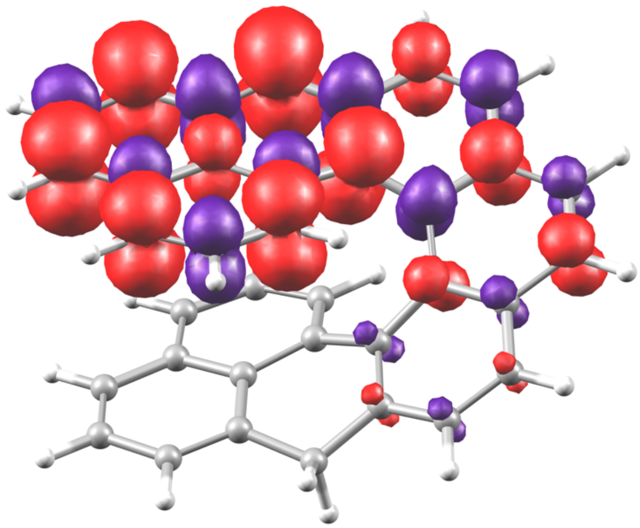

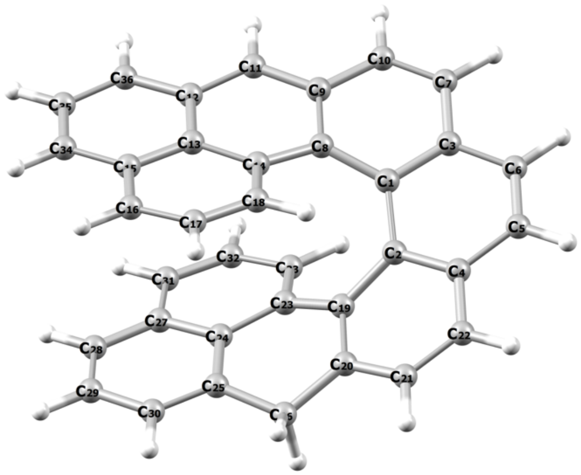

Isotropic Fermi contact couplings:

Atom		a.u.	MegaHertz	Gauss	10(-4) cm-1
1	C(13)	-0.03267	-36.72665	-13.10498	-12.25069
2	C(13)	0.01666	18.72482	6.68148	6.24593
3	C(13)	0.03005	33.78003	12.05355	11.26780
4	C(13)	-0.01398	-15.72062	-5.60951	-5.24384
5	C(13)	0.01722	19.36010	6.90817	6.45783
6	C(13)	-0.01635	-18.37974	-6.55835	-6.13082
7	C(13)	-0.02335	-26.25077	-9.36693	-8.75632
8	C(13)	0.05447	61.22951	21.84821	20.42396
9	C(13)	-0.05060	-56.88136	-20.29668	-18.97358
10	C(13)	0.02511	28.22449	10.07120	9.41468
11	C(13)	0.07689	86.43612	30.84256	28.83199
12	C(13)	-0.05319	-59.79159	-21.33513	-19.94433
13	C(13)	0.03263	36.68133	13.08881	12.23557
14	C(13)	-0.04335	-48.73372	-17.38940	-16.25582
15	C(13)	-0.04147	-46.61766	-16.63434	-15.54998
16	C(13)	0.04399	49.45521	17.64685	16.49648
17	C(13)	-0.03481	-39.13416	-13.96404	-13.05375
18	C(13)	0.04735	53.23460	18.99543	17.75715
19	C(13)	-0.00845	-9.49794	-3.38910	-3.16817
20	C(13)	0.00739	8.30576	2.96370	2.77050
21	C(13)	-0.00658	-7.39895	-2.64013	-2.46802
22	C(13)	0.00702	7.89324	2.81650	2.63290
23	C(13)	0.00430	4.83552	1.72543	1.61296
24	C(13)	-0.00239	-2.68735	-0.95891	-0.89640
25	C(13)	0.00145	1.62868	0.58115	0.54327
26	C(13)	-0.00146	-1.64500	-0.58698	-0.54871
27	C(13)	0.00179	2.01406	0.71867	0.67182
28	C(13)	-0.00097	-1.09298	-0.39000	-0.36458
29	C(13)	0.00165	1.85792	0.66295	0.61974
30	C(13)	-0.00138	-1.55134	-0.55356	-0.51747
31	C(13)	-0.00214	-2.40347	-0.85762	-0.80171
32	C(13)	0.00202	2.27123	0.81043	0.75760
33	C(13)	-0.00236	-2.64889	-0.94519	-0.88358
34	C(13)	0.06110	68.68562	24.50874	22.91106
35	C(13)	-0.04645	-52.22352	-18.63465	-17.41989
36	C(13)	0.06348	71.36509	25.46484	23.80483
37	H(1)	-0.00134	-6.00518	-2.14280	-2.00311
38	H(1)	0.00083	3.72292	1.32843	1.24183
39	H(1)	0.00153	6.83971	2.44058	2.28148
40	H(1)	-0.00160	-7.15478	-2.55300	-2.38658
41	H(1)	-0.00594	-26.55601	-9.47585	-8.85813
42	H(1)	-0.00338	-15.12235	-5.39603	-5.04427
43	H(1)	0.00179	7.99548	2.85299	2.66701
44	H(1)	-0.00361	-16.12180	-5.75266	-5.37765
45	H(1)	0.00044	1.98519	0.70837	0.66219
46	H(1)	-0.00042	-1.89900	-0.67761	-0.63344
47	H(1)	0.00079	3.55334	1.26792	1.18527
48	H(1)	0.00031	1.37818	0.49177	0.45971
49	H(1)	0.00011	0.48456	0.17290	0.16163
50	H(1)	-0.00009	-0.40889	-0.14590	-0.13639
51	H(1)	0.00009	0.41997	0.14986	0.14009
52	H(1)	0.00017	0.74349	0.26530	0.24800
53	H(1)	-0.00012	-0.52259	-0.18647	-0.17432
54	H(1)	0.00038	1.71363	0.61146	0.57160
55	H(1)	-0.00477	-21.33508	-7.61289	-7.11662
56	H(1)	0.00217	9.71979	3.46826	3.24217
57	H(1)	-0.00489	-21.86412	-7.80166	-7.29308

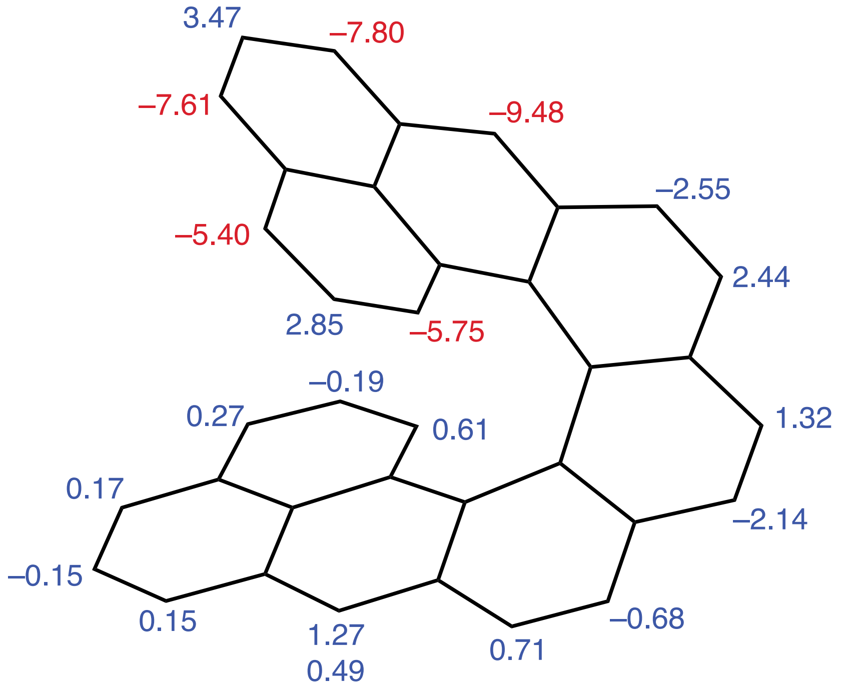

Figure S125. Calculated proton hyperfine coupling constants (in gauss) for H-**NC·**. π-Bonds are omitted for clarity. Main contributions (> 5 gauss) are depicted in red, smaller contributions (< 5 gauss) in blue.

Mulliken charges and spin densities (NC-Triplet):

		1	2
1	C	0.094665	-0.210019
2	C	0.029118	0.116369
3	C	-0.104181	0.441850
4	C	0.048760	-0.177341
5	C	-0.010336	0.280569
6	C	0.027649	-0.169647
7	C	-0.015412	0.086237
8	C	-0.022424	-0.040008
9	C	-0.018117	-0.088273
10	C	0.016253	0.066382
11	C	-0.022381	-0.040018
12	C	0.016229	0.066376
13	C	-0.019811	0.022477
14	C	-0.019820	0.022470
15	C	-0.010192	0.280530
16	C	-0.018124	-0.088262
17	C	-0.015422	0.086225
18	C	0.048733	-0.177324
19	C	0.027574	-0.169652
20	C	-0.104127	0.441849
21	C	0.094536	-0.210017
22	C	0.029196	0.116372
23	C	0.050619	-0.166590
24	C	-0.041706	0.258376
25	C	0.006988	-0.155081
26	C	-0.016325	0.268037
27	C	-0.016291	0.268065
28	C	0.007050	-0.155091
29	C	-0.041710	0.258409
30	C	0.050603	-0.166599
31	C	-0.052810	0.360247
32	C	0.004005	-0.200911
33	C	-0.058784	0.368437
34	C	-0.052805	0.360247
35	C	-0.058744	0.368427
36	C	0.003998	-0.200915
37	H	-0.006223	-0.019022
38	H	0.008890	-0.003436
39	H	0.007851	0.003910
40	H	0.008180	-0.001381
41	H	0.008182	-0.001381
42	H	0.007849	0.003909
43	H	0.008888	-0.003435
44	H	-0.006225	-0.019022
45	H	0.003654	-0.011238
46	H	0.016152	0.005486
47	H	0.015160	-0.010735
48	H	0.015158	-0.010735
49	H	0.016141	0.005483
50	H	0.003647	-0.011240
51	H	0.005544	-0.015907
52	H	0.017711	0.007310
53	H	0.004867	-0.016086
54	H	0.005539	-0.015906
55	H	0.004871	-0.016086
56	H	0.017710	0.007310

Sum of Mulliken charges = -0.00000 2.00000

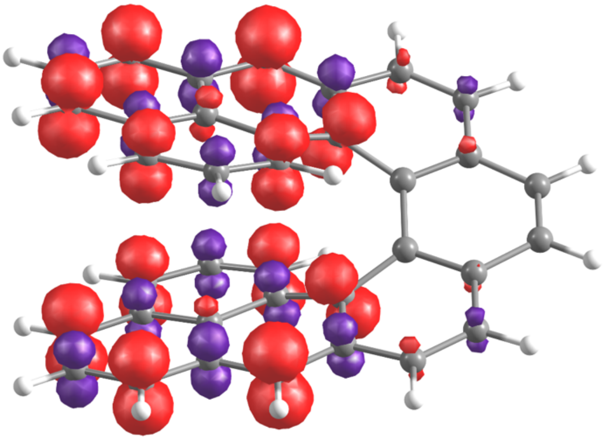

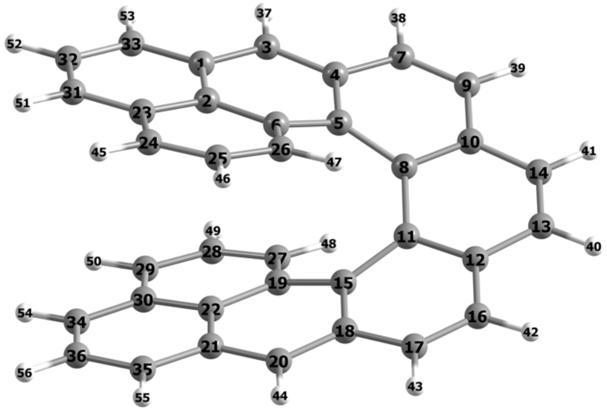

S11. Cartesian coordinates

Table S4. Cartesian coordinates.

**NC**-broken-symmetry singlet			
C	1.294826000	-2.949207000	0.204821000
C	1.242725000	-1.685266000	0.880556000
C	0.079937000	-3.558288000	-0.169600000
C	-1.145414000	-2.869423000	-0.090255000
C	-1.167879000	-1.518090000	0.348209000
C	-0.000212000	-1.017243000	1.054196000
C	-2.378850000	-3.499473000	-0.468497000
C	-2.336340000	-0.724229000	0.054050000
C	-3.552020000	-2.812676000	-0.414211000
C	-3.562973000	-1.402171000	-0.161354000
C	-2.336336000	0.724234000	-0.054046000
C	-3.562969000	1.402179000	0.161350000
C	-4.784142000	0.672918000	0.125019000
C	-4.784143000	-0.672905000	-0.125031000
C	-1.167871000	1.518090000	-0.348199000
C	-3.552013000	2.812685000	0.414203000
C	-2.378841000	3.499479000	0.468492000
C	-1.145405000	2.869426000	0.090259000
C	-0.000206000	1.017241000	-1.054187000
C	0.079946000	3.558291000	0.169596000
C	1.294835000	2.949209000	-0.204827000
C	1.242732000	1.685263000	-0.880555000
C	2.443397000	-1.107938000	1.390149000
C	2.362740000	0.076961000	2.157410000
C	1.129202000	0.633902000	2.451227000
C	-0.040649000	0.094573000	1.914101000
C	-0.040649000	-0.094577000	-1.914088000
C	1.129202000	-0.633917000	-2.451207000
C	2.362741000	-0.076977000	-2.157394000
C	2.443401000	1.107932000	-1.390148000
C	3.682876000	-1.747646000	1.118034000
C	3.726507000	-2.946927000	0.421976000
C	2.550362000	-3.560120000	-0.011404000
C	3.682882000	1.747642000	-1.118042000
C	2.550371000	3.560123000	0.011389000
C	3.726515000	2.946928000	-0.421992000
H	0.091573000	-4.576400000	-0.567470000
H	-2.361551000	-4.554357000	-0.752382000
H	-4.501472000	-3.313596000	-0.617731000
H	-5.724237000	1.215762000	0.248295000
H	-5.724240000	-1.215746000	-0.248310000
H	-4.501465000	3.313608000	0.617715000
H	-2.361542000	4.554367000	0.752367000
H	0.091583000	4.576407000	0.567456000
H	3.281371000	0.528662000	2.538266000
H	1.066629000	1.516536000	3.090705000
H	-0.998832000	0.554593000	2.153474000
H	-0.998834000	-0.554604000	-2.153446000
H	1.066626000	-1.516562000	-3.090669000
H	3.281372000	-0.528686000	-2.538244000
H	4.603984000	-1.284583000	1.479494000
H	4.688291000	-3.427558000	0.229565000
H	2.589368000	-4.523848000	-0.524697000
H	4.603988000	1.284576000	-1.479501000
H	2.589379000	4.523854000	0.524676000
H	4.688300000	3.427559000	-0.229586000

- Thermochemistry -

Temperature 298.150 Kelvin. Pressure 1.00000 Atm.

Zero-point correction= 0.442781 (Hartree/Particle)
Thermal correction to Energy= 0.465900
Thermal correction to Enthalpy= 0.466844
Thermal correction to Gibbs Free Energy= 0.392830
Sum of electronic and zero-point Energies= -1382.033942
Sum of electronic and thermal Energies= -1382.010823
Sum of electronic and thermal Enthalpies= -1382.009879
Sum of electronic and thermal Free Energies= -1382.083893

Charge = 0 Multiplicity = 1

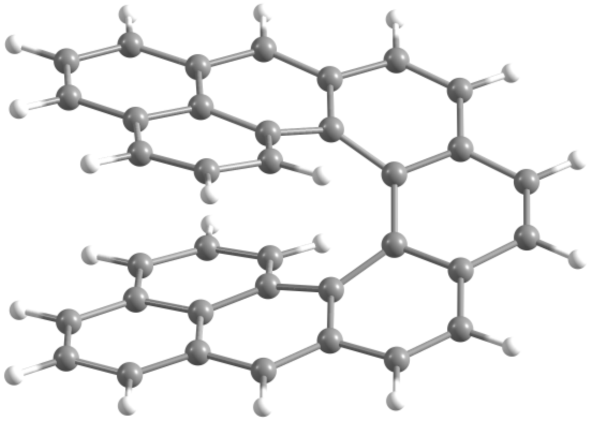

NC-restricted singlet

C	1.371147000	-2.585512000	0.356561000
C	1.231987000	-1.335750000	1.042392000
C	0.192305000	-3.327308000	0.053208000
C	-1.075129000	-2.780801000	0.189742000
C	-1.191814000	-1.402348000	0.568337000
C	-0.058992000	-0.778695000	1.240802000
C	-2.287035000	-3.495049000	-0.153148000
C	-2.336026000	-0.711614000	0.137097000
C	-3.487520000	-2.861169000	-0.192645000
C	-3.562099000	-1.427030000	-0.061252000
C	-2.336014000	0.711652000	-0.137190000
C	-3.562075000	1.427079000	0.061212000
C	-4.752952000	0.695980000	0.080896000
C	-4.752969000	-0.695912000	-0.080946000
C	-1.191791000	1.402376000	-0.568362000
C	-3.487482000	2.861212000	0.192645000
C	-2.286981000	3.495067000	0.153193000
C	-1.075086000	2.780803000	-0.189691000
C	-0.058969000	0.778703000	-1.240802000
C	0.192359000	3.327274000	-0.053087000
C	1.371192000	2.585479000	-0.356481000
C	1.232012000	1.335738000	-1.042376000
C	2.386651000	-0.643489000	1.509466000
C	2.215806000	0.523513000	2.288974000
C	0.943626000	0.967136000	2.607583000
C	-0.183481000	0.335618000	2.079900000
C	-0.183503000	-0.335664000	-2.079814000
C	0.943581000	-0.967153000	-2.607584000
C	2.215773000	-0.523479000	-2.289072000
C	2.386646000	0.643489000	-1.509525000
C	3.670730000	-1.142262000	1.157878000
C	3.792908000	-2.307114000	0.423708000
C	2.658943000	-3.035712000	0.038446000
C	3.670745000	1.142251000	-1.157961000
C	2.658994000	3.035655000	-0.038397000
C	3.792945000	2.307058000	-0.423736000
H	0.297236000	-4.341434000	-0.342589000
H	-2.217069000	-4.567172000	-0.352712000
H	-4.407890000	-3.420502000	-0.377787000
H	-5.702510000	1.229221000	0.172209000
H	-5.702543000	-1.229127000	-0.172225000
H	-4.407845000	3.420556000	0.377782000
H	-2.216980000	4.567186000	0.352763000
H	0.297294000	4.341384000	0.342754000
H	3.097841000	1.050405000	2.658642000
H	0.815329000	1.841920000	3.248162000
H	-1.176365000	0.732724000	2.298493000
H	-1.176409000	-0.732772000	-2.298322000
H	0.815274000	-1.841969000	-3.248115000
H	3.097793000	-1.050409000	-2.658723000
H	4.557728000	-0.602146000	1.495266000
H	4.784689000	-2.680092000	0.158296000
H	2.775649000	-3.981545000	-0.496422000
H	4.557723000	0.602137000	-1.495407000
H	2.775737000	3.981471000	0.496491000
H	4.784731000	2.680051000	-0.158359000

- Thermochemistry -

Temperature 298.150 Kelvin. Pressure 1.00000 Atm.

Zero-point correction= 0.445319 (Hartree/Particle)
Thermal correction to Energy= 0.468054
Thermal correction to Enthalpy= 0.468999
Thermal correction to Gibbs Free Energy= 0.396675
Sum of electronic and zero-point Energies= -1382.003577
Sum of electronic and thermal Energies= -1381.980842
Sum of electronic and thermal Enthalpies= -1381.979898
Sum of electronic and thermal Free Energies= -1382.052221

Charge = 0 Multiplicity = 1

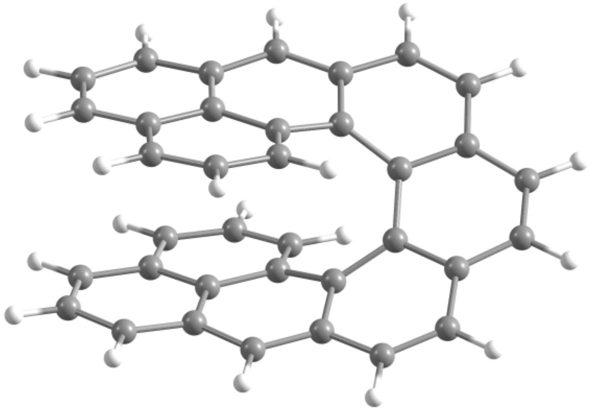

The wavefunction has an RHF -> UHF instability.

NC-triplet

C	1.292290000	-2.959368000	0.198855000
C	1.242319000	-1.696020000	0.876715000
C	0.079005000	-3.563015000	-0.178887000
C	-1.146387000	-2.868888000	-0.100022000
C	-1.166401000	-1.521848000	0.341968000
C	0.001372000	-1.025040000	1.048937000
C	-2.380451000	-3.497475000	-0.478942000
C	-2.337383000	-0.724226000	0.052658000
C	-3.552588000	-2.811191000	-0.417760000
C	-3.561626000	-1.400615000	-0.160998000
C	-2.337447000	0.723993000	-0.052555000
C	-3.561796000	1.400230000	0.161008000
C	-4.785166000	0.671270000	0.124982000
C	-4.785091000	-0.671821000	-0.124881000
C	-1.166577000	1.521732000	-0.342019000
C	-3.552987000	2.810861000	0.417453000
C	-2.380987000	3.497404000	0.478281000
C	-1.146831000	2.868957000	0.099434000
C	0.001373000	1.024860000	-1.048675000
C	0.078364000	3.563490000	0.177815000
C	1.291806000	2.959977000	-0.199632000
C	1.242182000	1.696148000	-0.876629000
C	2.444126000	-1.122959000	1.389549000
C	2.365736000	0.060461000	2.158202000
C	1.132769000	0.620651000	2.451102000
C	-0.036878000	0.086230000	1.911067000
C	-0.036501000	-0.086878000	-1.910237000
C	1.133361000	-0.621596000	-2.449512000
C	2.366190000	-0.061127000	-2.156559000
C	2.444193000	1.122946000	-1.388863000
C	3.682353000	-1.766104000	1.119216000
C	3.724247000	-2.965315000	0.421303000
C	2.548590000	-3.574204000	-0.015616000
C	3.682224000	1.766638000	-1.118976000
C	2.547930000	3.575355000	0.014342000
C	3.723760000	2.966464000	-0.422092000
H	0.087303000	-4.580055000	-0.579461000
H	-2.363898000	-4.551311000	-0.766659000
H	-4.503036000	-3.310533000	-0.620286000
H	-5.724550000	1.215214000	0.248551000
H	-5.724412000	-1.215880000	-0.248420000
H	-4.503508000	3.310068000	0.619975000
H	-2.364620000	4.551329000	0.765682000
H	0.086390000	4.580754000	0.577822000
H	3.284891000	0.508637000	2.541890000
H	1.071797000	1.501961000	3.092538000
H	-0.994056000	0.548113000	2.150426000
H	-0.993573000	-0.548878000	-2.149789000
H	1.072658000	-1.503364000	-3.090345000
H	3.285517000	-0.509536000	-2.539562000
H	4.604052000	-1.306161000	1.483053000
H	4.685328000	-3.448045000	0.230689000
H	2.585674000	-4.536874000	-0.530945000
H	4.604065000	1.306642000	-1.482385000
H	2.584733000	4.538438000	0.528917000
H	4.684698000	3.449638000	-0.231883000

- Thermochemistry -

Temperature 298.150 Kelvin. Pressure 1.00000 Atm.

Zero-point correction= 0.443257 (Hartree/Particle)
Thermal correction to Energy= 0.466311
Thermal correction to Enthalpy= 0.467255
Thermal correction to Gibbs Free Energy= 0.392372
Sum of electronic and zero-point Energies= -1382.031488
Sum of electronic and thermal Energies= -1382.008435
Sum of electronic and thermal Enthalpies= -1382.007491
Sum of electronic and thermal Free Energies= -1382.082374

Charge = 0 Multiplicity = 3

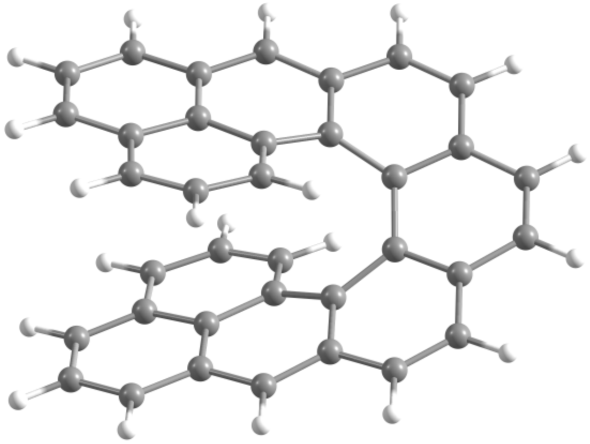

(*P*)-**6**

C	-2.541982000	0.721545000	-0.075814000
C	-2.541973000	-0.721570000	0.075824000
C	-3.762160000	1.407902000	0.116953000
C	-3.762141000	-1.407941000	-0.116960000
C	-4.988620000	-0.673664000	-0.103411000
C	-4.988629000	0.673609000	0.103404000
C	-3.749344000	2.820916000	0.329977000
C	-1.367872000	1.502586000	-0.389714000
C	-1.349747000	2.855988000	-0.000671000
C	-2.568915000	3.502536000	0.369555000
C	-0.098347000	3.541471000	0.070217000
C	1.066976000	2.929104000	-0.290183000
C	1.030894000	1.655952000	-0.965845000
C	-0.206564000	0.977171000	-1.087621000
C	2.203108000	1.120502000	-1.564744000
C	2.112604000	-0.041309000	-2.306971000
C	0.869352000	-0.664187000	-2.515532000
C	-0.269100000	-0.159150000	-1.927820000
C	-1.367855000	-1.502600000	0.389717000
C	-1.349708000	-2.855991000	0.000637000
C	-2.568865000	-3.502551000	-0.369602000
C	-3.749306000	-2.820951000	-0.330007000
C	-0.206561000	-0.977190000	1.087649000
C	1.030908000	-1.655946000	0.965857000
C	1.067016000	-2.929072000	0.290149000
C	-0.098296000	-3.541450000	-0.070271000
C	2.203109000	-1.120500000	1.564786000
C	3.501719000	-1.880695000	1.445483000
C	3.585221000	-2.695596000	0.156522000
C	2.372217000	-3.582291000	-0.026880000
C	2.112577000	0.041272000	2.307071000
C	0.869313000	0.664122000	2.515648000
C	-0.269124000	0.159093000	1.927899000
C	3.501703000	1.880730000	-1.445475000
C	3.585191000	2.695688000	-0.156549000
C	2.372164000	3.582353000	0.026840000
O	2.445205000	4.712736000	0.453323000
O	2.445280000	-4.712648000	-0.453425000
H	-5.928063000	-1.221162000	-0.208694000
H	-5.928081000	1.221096000	0.208675000
H	-4.696341000	3.331863000	0.518067000
H	-2.542390000	4.562528000	0.631818000
H	-0.051857000	4.547920000	0.493915000
H	3.010395000	-0.451086000	-2.778180000
H	0.804304000	-1.546425000	-3.155318000
H	-1.229838000	-0.634648000	-2.120493000
H	-2.542322000	-4.562537000	-0.631890000
H	-4.696295000	-3.331908000	-0.518111000
H	-0.051789000	-4.547886000	-0.493998000
H	3.594865000	-2.561601000	2.310270000
H	4.350685000	-1.184100000	1.511977000
H	4.487112000	-3.319969000	0.111959000
H	3.602317000	-2.008141000	-0.708456000
H	3.010356000	0.451040000	2.778312000
H	0.804243000	1.546329000	3.155474000
H	-1.229872000	0.634569000	2.120578000
H	3.594835000	2.561600000	-2.310291000
H	4.350682000	1.184149000	-1.511941000
H	4.487065000	3.320088000	-0.112020000
H	3.602315000	2.008269000	0.708454000

- Thermochemistry -

Temperature 298.150 Kelvin. Pressure 1.00000 Atm.

Zero-point correction= 0.478274 (Hartree/Particle)
Thermal correction to Energy= 0.504041
Thermal correction to Enthalpy= 0.504985
Thermal correction to Gibbs Free Energy= 0.424751
Sum of electronic and zero-point Energies= -1533.545586
Sum of electronic and thermal Energies= -1533.519819
Sum of electronic and thermal Enthalpies= -1533.518875
Sum of electronic and thermal Free Energies= -1533.599109

Charge = 0 Multiplicity = 1

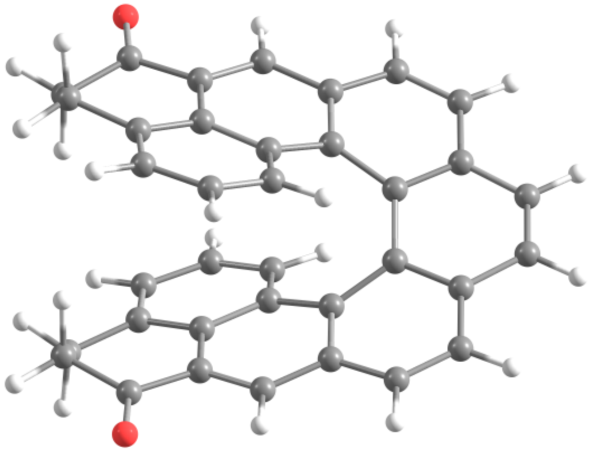

H-**NC·**

C	-2.263881000	-0.883766000	0.074008000
C	-2.391080000	0.567007000	-0.020729000
C	-3.433652000	-1.657904000	-0.102078000
C	-3.662168000	1.131339000	0.268320000
C	-4.822492000	0.291150000	0.283386000
C	-4.717279000	-1.036737000	0.004583000
C	-3.319869000	-3.058935000	-0.368837000
C	-1.027990000	-1.579242000	0.328485000
C	-0.911805000	-2.925321000	-0.109171000
C	-2.097010000	-3.649341000	-0.463574000
C	0.360700000	-3.525233000	-0.204681000
C	1.530592000	-2.839088000	0.169073000
C	1.396002000	-1.581161000	0.845660000
C	0.113107000	-0.990059000	1.013106000
C	2.556106000	-0.938483000	1.370383000
C	2.400487000	0.234663000	2.142743000
C	1.134468000	0.719703000	2.423796000
C	0.002638000	0.116158000	1.874403000
C	-1.327675000	1.461037000	-0.389662000
C	-1.428722000	2.802667000	-0.036814000
C	-2.653000000	3.322396000	0.437396000
C	-3.767871000	2.523693000	0.502980000
C	-0.115708000	1.022872000	-1.126240000
C	1.104188000	1.733228000	-0.903122000
C	1.085759000	3.010504000	-0.276889000
C	-0.239100000	3.720161000	-0.173092000
C	2.339345000	1.168253000	-1.320756000
C	3.546057000	1.815489000	-0.941419000
C	3.511921000	2.993054000	-0.235302000
C	2.275237000	3.611278000	0.067953000
C	2.318415000	-0.009141000	-2.114493000
C	1.119909000	-0.566406000	-2.488764000
C	-0.098027000	-0.058430000	-1.985412000
C	3.834274000	-1.501317000	1.106510000
C	3.956724000	-2.690771000	0.402238000
C	2.823767000	-3.371235000	-0.042926000
H	-5.797613000	0.750164000	0.462278000
H	-5.609199000	-1.661969000	-0.080893000
H	-4.232399000	-3.633040000	-0.546250000
H	-2.002838000	-4.697574000	-0.756706000
H	0.439262000	-4.538790000	-0.606558000
H	3.287750000	0.735346000	2.536153000
H	1.015685000	1.593857000	3.067021000
H	-0.981573000	0.518356000	2.110734000
H	-2.718915000	4.383876000	0.690037000
H	-4.741952000	2.944966000	0.762571000
H	-0.360909000	4.334938000	-1.085323000
H	-0.233786000	4.432725000	0.666589000
H	4.499143000	1.363944000	-1.227253000
H	4.443001000	3.482349000	0.059401000
H	2.267782000	4.591973000	0.551455000
H	3.264385000	-0.450389000	-2.436510000
H	1.101937000	-1.446156000	-3.135060000
H	-1.027758000	-0.571999000	-2.232599000
H	4.722315000	-0.985894000	1.479686000
H	4.947488000	-3.110815000	0.215022000
H	2.923982000	-4.328365000	-0.560166000

- Thermochemistry -

Temperature 298.150 Kelvin. Pressure 1.00000 Atm.

Zero-point correction= 0.456017 (Hartree/Particle)

Thermal correction to Energy= 0.479312

Thermal correction to Enthalpy= 0.480256

Thermal correction to Gibbs Free Energy= 0.405390

Sum of electronic and zero-point Energies= -1382.639840

Sum of electronic and thermal Energies= -1382.616545

Sum of electronic and thermal Enthalpies= -1382.615601

Sum of electronic and thermal Free Energies= -1382.690467

Charge = 0 Multiplicity = 2

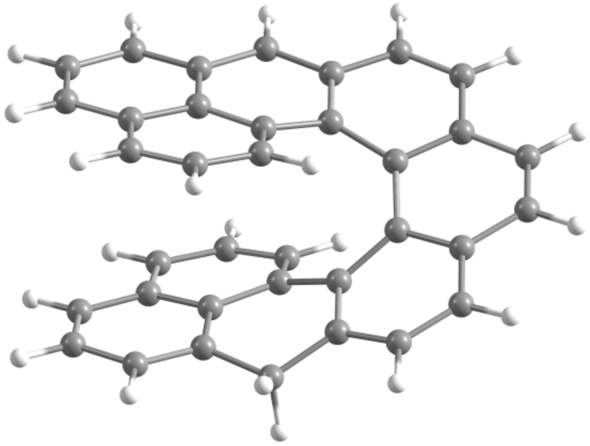

c-*H*-**NC·**

C	-2.428799000	0.400373000	-0.099614000
C	-1.628033000	1.538929000	-0.342978000
C	-3.777098000	0.539188000	0.305227000
C	-2.240616000	2.806646000	-0.363045000
C	-3.616787000	2.924924000	-0.034565000
C	-4.358155000	1.826355000	0.323340000
C	-4.505393000	-0.624967000	0.745556000
C	-1.853860000	-0.929746000	-0.182383000
C	-2.565032000	-2.046051000	0.349265000
C	-3.919110000	-1.845539000	0.810066000
C	-1.949390000	-3.292297000	0.375896000
C	-0.652196000	-3.469646000	-0.131579000
C	0.011717000	-2.364264000	-0.749582000
C	-0.612643000	-1.101522000	-0.789896000
C	1.319228000	-2.556974000	-1.310452000
C	1.938770000	-1.427188000	-1.993938000
C	1.350851000	-0.228594000	-2.080112000
C	0.010836000	0.092616000	-1.486568000
C	-0.114895000	1.364063000	-0.538747000
C	0.539463000	2.629450000	-1.066702000
C	-0.145161000	3.823190000	-1.123102000
C	-1.487489000	3.954349000	-0.759792000
C	0.627971000	1.047470000	0.779862000
C	2.060983000	1.127542000	0.772969000
C	2.776164000	1.788726000	-0.269356000
C	2.027046000	2.632674000	-1.274669000
C	2.797953000	0.544657000	1.845981000
C	4.215981000	0.520477000	1.784456000
C	4.879707000	1.087516000	0.724740000
C	4.153117000	1.746960000	-0.289572000
C	2.096469000	0.016180000	2.963192000
C	0.729206000	0.075867000	3.009221000
C	-0.004530000	0.579495000	1.909299000
C	1.927272000	-3.787287000	-1.221458000
C	1.273686000	-4.880186000	-0.594018000
C	0.018039000	-4.730889000	-0.071518000
H	-0.647457000	0.349202000	-2.340934000
H	-4.074757000	3.916740000	-0.053963000
H	-5.403850000	1.936256000	0.620664000
H	-5.541211000	-0.492634000	1.067850000
H	-4.472066000	-2.708233000	1.189182000
H	-2.483052000	-4.149905000	0.794153000
H	2.902690000	-1.595563000	-2.480738000
H	1.828745000	0.539755000	-2.683545000
H	0.405095000	4.719829000	-1.422245000
H	-1.976087000	4.929924000	-0.790205000
H	2.295487000	2.327910000	-2.300259000
H	2.387833000	3.669516000	-1.185467000
H	4.769608000	0.049484000	2.600323000
H	5.970663000	1.063613000	0.680712000
H	4.692337000	2.260456000	-1.090667000
H	2.666037000	-0.420800000	3.786859000
H	0.188685000	-0.301341000	3.879752000
H	-1.092248000	0.556026000	1.958024000
H	2.924596000	-3.925474000	-1.646201000
H	1.779287000	-5.846590000	-0.537886000
H	-0.491964000	-5.573167000	0.402044000

- Thermochemistry -

Temperature 298.150 Kelvin. Pressure 1.00000 Atm.

Zero-point correction= 0.457972 (Hartree/Particle)
Thermal correction to Energy= 0.480674
Thermal correction to Enthalpy= 0.481619
Thermal correction to Gibbs Free Energy= 0.407436
Sum of electronic and zero-point Energies= -1382.614157
Sum of electronic and thermal Energies= -1382.591455
Sum of electronic and thermal Enthalpies= -1382.590511
Sum of electronic and thermal Free Energies= -1382.664693

Charge = 0 Multiplicity = 2

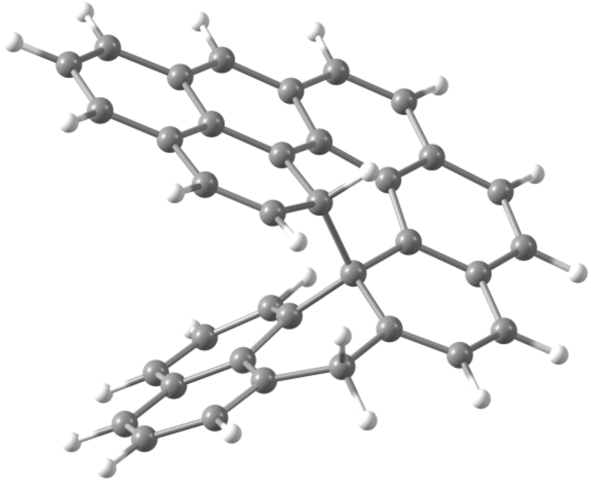

c-**NC***

C	-2.414690000	-0.279855000	0.026048000
C	-1.707974000	-1.469233000	-0.247099000
C	-3.767521000	-0.325377000	0.430261000
C	-2.427896000	-2.656410000	-0.408297000
C	-3.800078000	-2.686581000	-0.077282000
C	-4.444580000	-1.559757000	0.389799000
C	-4.397735000	0.891401000	0.890816000
C	-1.751744000	1.004445000	-0.102406000
C	-2.380333000	2.178472000	0.404272000
C	-3.727359000	2.068707000	0.919857000
C	-1.699938000	3.389447000	0.342468000
C	-0.420120000	3.473154000	-0.231929000
C	0.162414000	2.304189000	-0.814774000
C	-0.528270000	1.078108000	-0.765139000
C	1.458212000	2.391868000	-1.428757000
C	2.017849000	1.183152000	-2.028017000
C	1.372179000	0.010975000	-2.019860000
C	0.010983000	-0.176323000	-1.422890000
C	-0.189384000	-1.400637000	-0.425419000
C	0.390045000	-2.695535000	-0.986638000
C	-0.490399000	-3.776634000	-1.396916000
C	-1.786563000	-3.805908000	-1.038511000
C	0.579505000	-1.049273000	0.861307000
C	1.997749000	-1.209354000	0.822918000
C	2.610208000	-2.045509000	-0.166372000
C	1.727501000	-2.895401000	-0.949987000
C	2.814162000	-0.596659000	1.810647000
C	4.227633000	-0.702744000	1.699827000
C	4.800454000	-1.447634000	0.696354000
C	3.987289000	-2.152429000	-0.216486000
C	2.191514000	0.084100000	2.890243000
C	0.823845000	0.112895000	2.985138000
C	0.017532000	-0.448441000	1.967240000
C	2.125443000	3.594667000	-1.438396000
C	1.548116000	4.754971000	-0.858598000
C	0.311201000	4.700467000	-0.275888000
H	-0.665621000	-0.416551000	-2.268863000
H	-4.348490000	-3.625017000	-0.190204000
H	-5.492601000	-1.607933000	0.694968000
H	-5.431376000	0.836076000	1.241210000
H	-4.211162000	2.971470000	1.300546000
H	-2.169604000	4.294780000	0.736287000
H	2.996325000	1.262969000	-2.508366000
H	1.816462000	-0.851506000	-2.517448000
H	-0.035031000	-4.635293000	-1.896883000
H	-2.403679000	-4.680270000	-1.258912000
H	2.151551000	-3.802443000	-1.390981000
H	4.852472000	-0.193715000	2.437812000
H	5.887180000	-1.528218000	0.625250000
H	4.448012000	-2.809415000	-0.958630000
H	2.815644000	0.558298000	3.651398000
H	0.340815000	0.599975000	3.834598000
H	-1.064550000	-0.368829000	2.060499000
H	3.113913000	3.656816000	-1.900194000
H	2.100429000	5.696862000	-0.883224000
H	-0.136196000	5.593998000	0.165948000

- Thermochemistry -

Temperature 298.150 Kelvin. Pressure 1.00000 Atm.

Zero-point correction= 0.447772
(Hartree/Particle)
Thermal correction to Energy= 0.470099
Thermal correction to Enthalpy= 0.471043
Thermal correction to Gibbs Free Energy= 0.398549
Sum of electronic and zero-point Energies= -
1382.034880
Sum of electronic and thermal Energies= -1382.012553
Sum of electronic and thermal Enthalpies= -
1382.011609
Sum of electronic and thermal Free Energies= -
1382.084103

Charge = 0 Multiplicity = 1

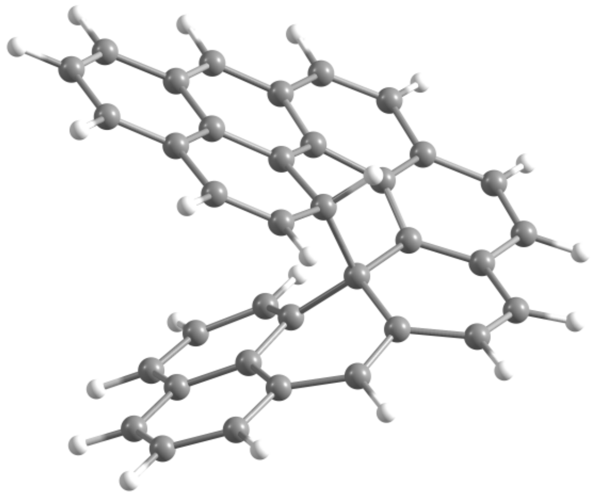

c-**NC**

C	1.461548000	1.754232000	0.122874000
C	0.225797000	2.050050000	-0.507445000
C	2.348302000	2.802626000	0.481208000
C	0.023646000	3.318540000	-1.033253000
C	0.942675000	4.359444000	-0.722920000
C	2.039834000	4.125020000	0.064317000
C	3.527038000	2.475419000	1.203070000
C	1.848887000	0.394779000	0.328859000
C	3.023267000	0.106097000	0.998568000
C	3.836859000	1.165807000	1.474201000
C	3.437661000	-1.326351000	1.201124000
C	2.897698000	-2.275032000	0.159107000
C	1.715482000	-1.929238000	-0.559129000
C	1.101882000	-0.649613000	-0.387802000
C	1.140445000	-2.869770000	-1.456496000
C	-0.052428000	-2.511743000	-2.136006000
C	-0.661261000	-1.303802000	-1.906174000
C	-0.103823000	-0.360220000	-1.011345000
C	-0.822919000	0.952319000	-0.595464000
C	-1.990152000	1.331432000	-1.504684000
C	-1.952813000	2.569590000	-2.262480000
C	-1.069362000	3.542502000	-1.973450000
C	-1.416047000	0.576340000	0.790879000
C	-2.575820000	-0.256899000	0.776170000
C	-3.376088000	-0.370829000	-0.406816000
C	-3.123730000	0.594446000	-1.466936000
C	-2.990851000	-0.924059000	1.958943000
C	-4.100053000	-1.810730000	1.897541000
C	-4.806189000	-1.973142000	0.728970000
C	-4.465422000	-1.222292000	-0.415949000
C	-2.295949000	-0.666714000	3.171813000
C	-1.265600000	0.237233000	3.192878000
C	-0.819473000	0.857006000	1.999939000
C	1.774032000	-4.126354000	-1.645812000
C	2.927231000	-4.430868000	-0.966138000
C	3.485973000	-3.501895000	-0.059009000
H	4.534475000	-1.402239000	1.251840000
H	3.067948000	-1.655255000	2.191331000
H	0.750301000	5.361427000	-1.114338000
H	2.714511000	4.940551000	0.335326000
H	4.197090000	3.279063000	1.517996000
H	4.753393000	0.924824000	2.018922000
H	-0.492816000	-3.220005000	-2.841791000
H	-1.584882000	-1.063952000	-2.430701000
H	-2.749457000	2.741359000	-2.990703000
H	-1.127264000	4.512299000	-2.473412000
H	-3.948234000	0.833184000	-2.145345000
H	-4.390551000	-2.353401000	2.800445000
H	-5.658980000	-2.654245000	0.693601000
H	-5.086769000	-1.288946000	-1.312666000
H	-2.612130000	-1.176994000	4.084737000
H	-0.757684000	0.470234000	4.131133000
H	0.028358000	1.537910000	2.054441000
H	1.329292000	-4.843611000	-2.339803000
H	3.416395000	-5.395577000	-1.117262000
H	4.395355000	-3.766630000	0.487353000

- Thermochemistry -

Temperature 298.150 Kelvin. Pressure 1.00000 Atm.

Zero-point correction= 0.447527 (Hartree/Particle)
Thermal correction to Energy= 0.470034
Thermal correction to Enthalpy= 0.470978
Thermal correction to Gibbs Free Energy= 0.397961
Sum of electronic and zero-point Energies= -1382.044732
Sum of electronic and thermal Energies= -1382.022225
Sum of electronic and thermal Enthalpies= -1382.021281
Sum of electronic and thermal Free Energies= -1382.094298

Charge = 0 Multiplicity = 1

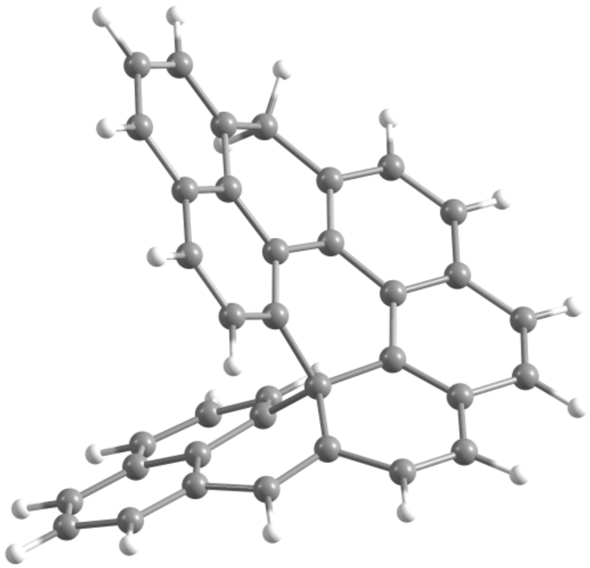

O-c-**NC**

C	1.173534000	1.931741000	0.052840000
C	-0.123714000	2.116724000	-0.488890000
C	1.962329000	3.052060000	0.424234000
C	-0.493613000	3.379700000	-0.930802000
C	0.321315000	4.498638000	-0.605341000
C	1.481877000	4.350059000	0.109150000
C	3.219466000	2.831081000	1.055717000
C	1.731783000	0.617656000	0.134487000
C	2.989074000	0.448446000	0.694818000
C	3.705490000	1.560812000	1.209670000
C	3.655325000	-0.881895000	0.691555000
C	2.999950000	-1.939726000	-0.125504000
C	1.735356000	-1.707594000	-0.737848000
C	1.042808000	-0.475957000	-0.552731000
C	1.150689000	-2.736171000	-1.522541000
C	-0.110532000	-2.492439000	-2.122942000
C	-0.784003000	-1.318048000	-1.895554000
C	-0.236464000	-0.307334000	-1.071095000
C	-1.054435000	0.917761000	-0.579638000
C	-2.302321000	1.210708000	-1.412340000
C	-2.431399000	2.476097000	-2.113912000
C	-1.647715000	3.526570000	-1.811084000
C	-1.531108000	0.430263000	0.821132000
C	-2.614209000	-0.500339000	0.835145000
C	-3.469464000	-0.644518000	-0.305122000
C	-3.360917000	0.371699000	-1.341337000
C	-2.899697000	-1.244508000	2.010811000
C	-3.931289000	-2.221877000	1.975503000
C	-4.689187000	-2.404083000	0.843100000
C	-4.480847000	-1.586902000	-0.288078000
C	-2.157119000	-0.975215000	3.191856000
C	-1.204366000	0.010302000	3.193014000
C	-0.885277000	0.710900000	2.004728000
C	1.843253000	-3.964862000	-1.680055000
C	3.059998000	-4.173465000	-1.071681000
C	3.637340000	-3.154892000	-0.287758000
O	4.700043000	-1.075866000	1.283977000
H	-0.001284000	5.491597000	-0.928140000
H	2.076264000	5.221587000	0.392553000
H	3.802275000	3.693373000	1.388078000
H	4.675331000	1.368546000	1.671329000
H	-0.554523000	-3.261882000	-2.758936000
H	-1.759888000	-1.165555000	-2.352837000
H	-3.274892000	2.591152000	-2.799105000
H	-1.833013000	4.506322000	-2.257523000
H	-4.240374000	0.558218000	-1.964812000
H	-4.120004000	-2.819491000	2.870612000
H	-5.481730000	-3.155020000	0.828208000
H	-5.145306000	-1.676355000	-1.151258000
H	-2.373355000	-1.545548000	4.098231000
H	-0.658760000	0.247557000	4.108491000
H	-0.093016000	1.456640000	2.042307000
H	1.388975000	-4.748841000	-2.291314000
H	3.580794000	-5.124957000	-1.194606000
H	4.600694000	-3.298774000	0.205877000

- Thermochemistry -

Temperature 298.150 Kelvin. Pressure 1.00000 Atm.

Zero-point correction= 0.429962 (Hartree/Particle)
Thermal correction to Energy= 0.452904
Thermal correction to Enthalpy= 0.453848
Thermal correction to Gibbs Free Energy= 0.379787
Sum of electronic and zero-point Energies= -1456.003356
Sum of electronic and thermal Energies= -1455.980413
Sum of electronic and thermal Enthalpies= -1455.979469
Sum of electronic and thermal Free Energies= -1456.053530

Charge = 0 Multiplicity = 1

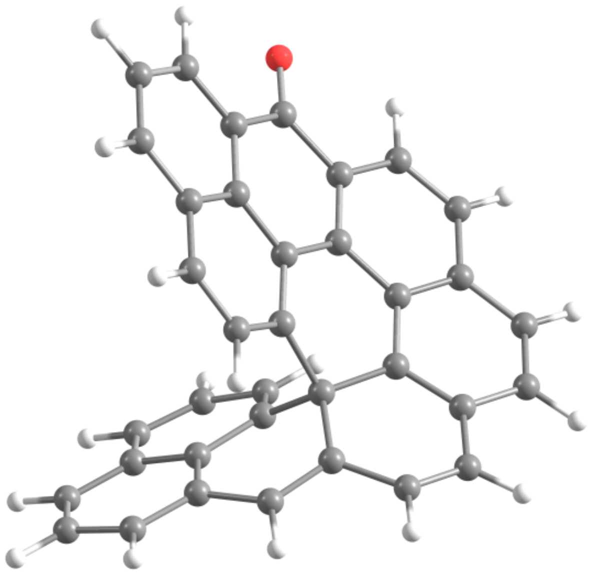

c-**NC·**

C	1.597629000	1.664883000	0.122440000
C	0.370416000	2.056595000	-0.459666000
C	2.543387000	2.642893000	0.519368000
C	0.227086000	3.365696000	-0.914672000
C	1.199537000	4.333590000	-0.564921000
C	2.299274000	3.994220000	0.191241000
C	3.728970000	2.215650000	1.208725000
C	1.931008000	0.271676000	0.220869000
C	3.147600000	-0.119770000	0.821356000
C	4.004181000	0.894982000	1.378595000
C	3.536047000	-1.472399000	0.786572000
C	2.793753000	-2.428886000	0.069621000
C	1.588511000	-2.022685000	-0.594952000
C	1.116979000	-0.690815000	-0.466010000
C	0.866085000	-2.963115000	-1.385867000
C	-0.295330000	-2.518468000	-2.056723000
C	-0.771076000	-1.227462000	-1.886303000
C	-0.115269000	-0.313379000	-1.057401000
C	-0.739503000	1.024514000	-0.586199000
C	-1.894043000	1.511998000	-1.459254000
C	-1.790551000	2.777882000	-2.164911000
C	-0.857873000	3.689555000	-1.835069000
C	-1.335567000	0.622512000	0.795802000
C	-2.556134000	-0.119126000	0.771985000
C	-3.384420000	-0.125493000	-0.396836000
C	-3.075690000	0.854715000	-1.428002000
C	-2.997655000	-0.804299000	1.934785000
C	-4.173641000	-1.600108000	1.862923000
C	-4.912509000	-1.659580000	0.705403000
C	-4.535305000	-0.890864000	-0.416411000
C	-2.260267000	-0.657549000	3.140583000
C	-1.159371000	0.158353000	3.175816000
C	-0.691441000	0.794704000	2.000759000
C	1.340342000	-4.297198000	-1.481018000
C	2.492183000	-4.690588000	-0.809320000
C	3.214115000	-3.778548000	-0.044545000
H	4.466884000	-1.774646000	1.273394000
H	1.059944000	5.364335000	-0.899775000
H	3.019954000	4.757289000	0.494620000
H	4.421309000	2.976619000	1.577138000
H	4.918415000	0.582212000	1.888668000
H	-0.837880000	-3.215583000	-2.699469000
H	-1.689187000	-0.932204000	-2.392522000
H	-2.580536000	3.025196000	-2.878463000
H	-0.867399000	4.683599000	-2.288469000
H	-3.890648000	1.177311000	-2.082870000
H	-4.487145000	-2.156162000	2.749817000
H	-5.815973000	-2.271298000	0.661792000
H	-5.175424000	-0.874160000	-1.302191000
H	-2.596754000	-1.182954000	4.037484000
H	-0.612296000	0.304414000	4.109438000
H	0.210665000	1.401017000	2.064862000
H	0.783562000	-5.013939000	-2.089067000
H	2.838649000	-5.723356000	-0.887502000
H	4.125102000	-4.092207000	0.470516000

- Thermochemistry -

Temperature 298.150 Kelvin. Pressure 1.00000 Atm.

Zero-point correction= 0.434590 (Hartree/Particle)
Thermal correction to Energy= 0.456838
Thermal correction to Enthalpy= 0.457782
Thermal correction to Gibbs Free Energy= 0.384832
Sum of electronic and zero-point Energies= -1381.440221
Sum of electronic and thermal Energies= -1381.417973
Sum of electronic and thermal Enthalpies= -1381.417029
Sum of electronic and thermal Free Energies= -1381.489979

Charge = 0 Multiplicity = 2

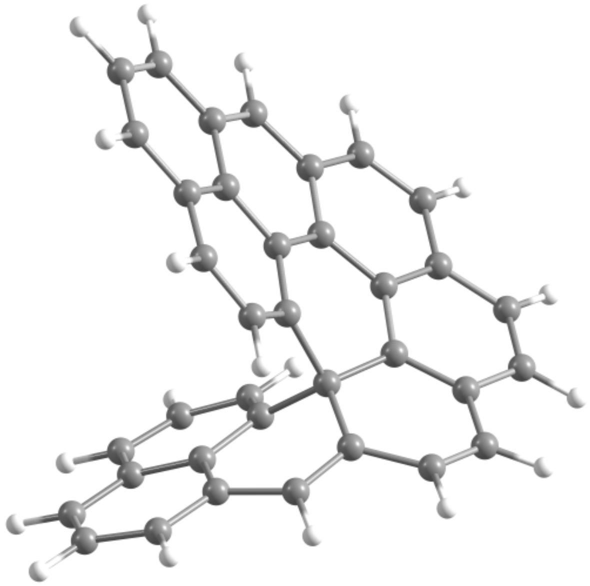

sym-(P,R,R,P)-(c-**NC**)$_2$

C	1.391765000	-1.288867000	1.563042000
C	2.277305000	-2.053469000	0.771379000
C	0.575157000	-1.918538000	2.534383000

- Thermochemistry -

Temperature 298.150 Kelvin. Pressure 1.00000 Atm.

C	2.135232000	-3.437406000	0.746489000	
C	1.294675000	-4.068164000	1.696068000	Zero-point correction= 0.876016 (Hartree/Particle)
C	0.576550000	-3.331387000	2.607179000	
C	-0.220221000	-1.099287000	3.397767000	Thermal correction to Energy= 0.921029
C	1.271653000	0.124217000	1.342815000	Thermal correction to Enthalpy= 0.921973
C	0.439060000	0.891097000	2.164391000	Thermal correction to Gibbs Free Energy= 0.802924
C	-0.260497000	0.253462000	3.239690000	Sum of electronic and zero-point Energies= -2762.929105
C	0.187555000	2.256850000	1.830853000	
C	0.643915000	2.798679000	0.661685000	Sum of electronic and thermal Energies= -2762.884091
C	1.565783000	2.047181000	-0.148383000	Sum of electronic and thermal Enthalpies= -2762.883147
C	1.898248000	0.717745000	0.196648000	
C	2.181264000	2.655400000	-1.276757000	Sum of electronic and thermal Free Energies= -2763.002197
C	3.003972000	1.890763000	-2.081239000	
C	3.295041000	0.554265000	-1.760943000	
C	2.810846000	-0.023820000	-0.604952000	Charge = 0 Multiplicity = 1
C	3.373177000	-1.338102000	-0.003949000	
C	4.050650000	-2.251693000	-1.023636000	
C	3.550322000	-3.599467000	-1.241399000	
C	2.748400000	-4.195164000	-0.339491000	
C	4.484199000	-0.789893000	0.942911000	
C	5.708357000	-0.382400000	0.329035000	
C	6.046111000	-0.820110000	-0.992534000	
C	5.241094000	-1.893927000	-1.556891000	
C	6.637461000	0.413910000	1.049570000	
C	7.811769000	0.872573000	0.392093000	
C	8.080561000	0.505431000	-0.904787000	
C	7.210894000	-0.372122000	-1.586783000	
C	6.373810000	0.714993000	2.413063000	
C	5.250257000	0.217202000	3.019667000	
C	4.300369000	-0.530740000	2.282865000	
C	1.930710000	4.072751000	-1.533686000	
C	0.982758000	4.750921000	-0.874809000	
C	0.076405000	4.094286000	0.123503000	
C	-1.174218000	0.309951000	-1.818831000	
C	-1.454908000	-1.005964000	-1.374417000	
C	-0.301448000	0.511222000	-2.919542000	
C	-0.608156000	-2.034810000	-1.760927000	
C	0.345826000	-1.811523000	-2.791512000	
C	0.436197000	-0.594830000	-3.415531000	
C	-0.178327000	1.825138000	-3.440440000	
C	-1.665223000	1.431546000	-1.085590000	
C	-1.319955000	2.710807000	-1.489880000	
C	-0.695530000	2.892135000	-2.751455000	
C	-1.387441000	3.855961000	-0.514090000	
C	-2.369954000	3.636981000	0.606258000	
C	-2.732995000	2.313571000	0.987063000	
C	-2.382292000	1.190238000	0.172903000	
C	-3.456232000	2.114270000	2.194445000	
C	-3.730080000	0.783819000	2.603989000	
C	-3.377464000	-0.277824000	1.815193000	
C	-2.754499000	-0.090577000	0.556830000	
C	-2.669822000	-1.231885000	-0.493553000	
C	-2.698369000	-2.640303000	0.099958000	
C	-1.570495000	-3.534174000	-0.092482000	
C	-0.650237000	-3.301923000	-1.044064000	
C	-4.004542000	-1.023938000	-1.272818000	
C	-5.196377000	-1.508035000	-0.650459000	
C	-5.130691000	-2.476520000	0.403403000	
C	-3.847476000	-3.126404000	0.622767000	
C	-6.469428000	-1.083316000	-1.116866000	
C	-7.637549000	-1.518104000	-0.433066000	

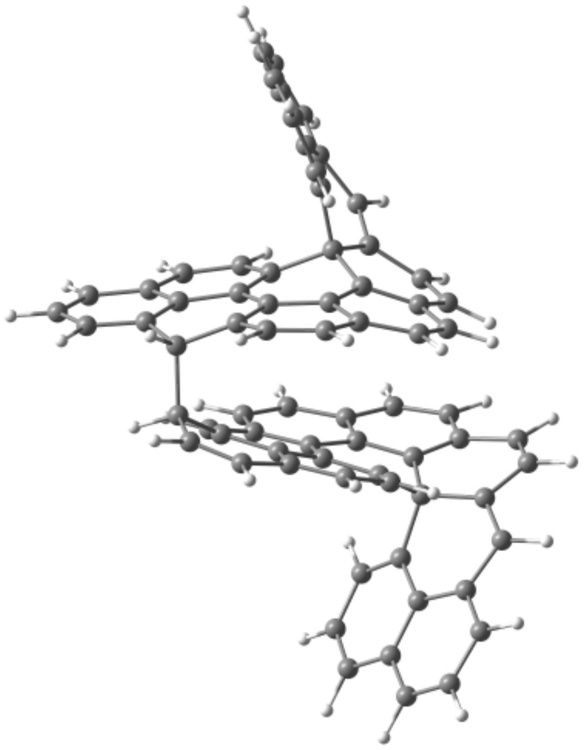

C	-7.548504000	-2.387986000	0.627381000	
C	-6.292956000	-2.894940000	1.024027000	
C	-6.538419000	-0.250301000	-2.265601000	
C	-5.389473000	0.106927000	-2.921821000	
C	-4.120752000	-0.274752000	-2.423234000	
C	-3.858337000	3.237103000	2.962152000	
C	-3.558494000	4.510327000	2.541627000	
C	-2.805669000	4.704805000	1.364082000	
H	-1.634785000	4.792973000	-1.038767000	
H	-0.100542000	4.789477000	0.958820000	
H	1.222539000	-5.158874000	1.691171000	
H	-0.040204000	-3.827436000	3.360397000	
H	-0.816487000	-1.582239000	4.175626000	
H	-0.894310000	0.866329000	3.884528000	
H	-0.482007000	2.831604000	2.474646000	
H	3.459947000	2.339144000	-2.967539000	
H	3.959929000	-0.008210000	-2.414331000	
H	3.974771000	-4.170282000	-2.071282000	
H	2.496058000	-5.254547000	-0.434554000	
H	5.708804000	-2.534491000	-2.310758000	
H	8.504168000	1.515481000	0.941094000	
H	8.986802000	0.860782000	-1.399652000	
H	7.473999000	-0.727822000	-2.586305000	
H	7.086894000	1.327100000	2.970385000	
H	5.060570000	0.416733000	4.076309000	
H	3.401497000	-0.870941000	2.794414000	
H	2.541414000	4.567636000	-2.293724000	
H	0.805292000	5.807241000	-1.097000000	
H	0.994223000	-2.638602000	-3.090928000	
H	1.127405000	-0.443669000	-4.247626000	
H	0.391420000	1.983272000	-4.359109000	
H	-0.544114000	3.905777000	-3.125251000	
H	-4.236499000	0.614534000	3.557197000	
H	-3.596463000	-1.287609000	2.155849000	
H	-1.569413000	-4.474467000	0.463280000	
H	0.121667000	-4.040558000	-1.267989000	
H	-3.854382000	-4.123363000	1.073433000	
H	-8.610900000	-1.156437000	-0.773512000	
H	-8.452251000	-2.718790000	1.143489000	
H	-6.237320000	-3.650187000	1.812225000	
H	-7.515816000	0.077951000	-2.627175000	
H	-5.439127000	0.715345000	-3.827270000	
H	-3.234739000	0.065817000	-2.955709000	
H	-4.417246000	3.072137000	3.886509000	
H	-3.885346000	5.374881000	3.123273000	
H	-2.537352000	5.721132000	1.061264000	
nonsym-(*P*,*S*,*S*,*P*)-(c-**NC**)$_2$				
C	1.391765000	-1.288867000	1.563042000	- Thermochemistry -<br>-------------------<br>Temperature 298.150 Kelvin. Pressure 1.00000 Atm.<br><br>Zero-point correction= 0.876016 (Hartree/Particle)<br>Thermal correction to Energy= 0.921029<br>Thermal correction to Enthalpy= 0.921973<br>Thermal correction to Gibbs Free Energy= 0.802924<br>Sum of electronic and zero-point Energies= -2762.929105<br>Sum of electronic and thermal Energies= -2762.884091
C	2.277305000	-2.053469000	0.771379000	
C	0.575157000	-1.918538000	2.534383000	
C	2.135232000	-3.437406000	0.746489000	
C	1.294675000	-4.068164000	1.696068000	
C	0.576550000	-3.331387000	2.607179000	
C	-0.220221000	-1.099287000	3.397767000	
C	1.271653000	0.124217000	1.342815000	
C	0.439060000	0.891097000	2.164391000	
C	-0.260497000	0.253462000	3.239690000	
C	0.187555000	2.256850000	1.830853000	
C	0.643915000	2.798679000	0.661685000	
C	1.565783000	2.047181000	-0.148383000	

C	1.898248000	0.717745000	0.196648000
C	2.181264000	2.655400000	-1.276757000
C	3.003972000	1.890763000	-2.081239000
C	3.295041000	0.554265000	-1.760943000
C	2.810846000	-0.023820000	-0.604952000
C	3.373177000	-1.338102000	-0.003949000
C	4.050650000	-2.251693000	-1.023636000
C	3.550322000	-3.599467000	-1.241399000
C	2.748400000	-4.195164000	-0.339491000
C	4.484199000	-0.789893000	0.942911000
C	5.708357000	-0.382400000	0.329035000
C	6.046111000	-0.820110000	-0.992534000
C	5.241094000	-1.893927000	-1.556891000
C	6.637461000	0.413910000	1.049570000
C	7.811769000	0.872573000	0.392093000
C	8.080561000	0.505431000	-0.904787000
C	7.210894000	-0.372122000	-1.586783000
C	6.373810000	0.714993000	2.413063000
C	5.250257000	0.217202000	3.019667000
C	4.300369000	-0.530740000	2.282865000
C	1.930710000	4.072751000	-1.533686000
C	0.982758000	4.750921000	-0.874809000
C	0.076405000	4.094286000	0.123503000
C	-1.174218000	0.309951000	-1.818831000
C	-1.454908000	-1.005964000	-1.374417000
C	-0.301448000	0.511222000	-2.919542000
C	-0.608156000	-2.034810000	-1.760927000
C	0.345826000	-1.811523000	-2.791512000
C	0.436197000	-0.594830000	-3.415531000
C	-0.178327000	1.825138000	-3.440440000
C	-1.665223000	1.431546000	-1.085590000
C	-1.319955000	2.710807000	-1.489880000
C	-0.695530000	2.892135000	-2.751455000
C	-1.387441000	3.855961000	-0.514090000
C	-2.369954000	3.636981000	0.606258000
C	-2.732995000	2.313571000	0.987063000
C	-2.382292000	1.190238000	0.172903000
C	-3.456232000	2.114270000	2.194445000
C	-3.730080000	0.783819000	2.603989000
C	-3.377464000	-0.277824000	1.815193000
C	-2.754499000	-0.090577000	0.556830000
C	-2.669822000	-1.231885000	-0.493553000
C	-2.698369000	-2.640303000	0.099958000
C	-1.570495000	-3.534174000	-0.092482000
C	-0.650237000	-3.301923000	-1.044064000
C	-4.004542000	-1.023938000	-1.272818000
C	-5.196377000	-1.508035000	-0.650459000
C	-5.130691000	-2.476520000	0.403403000
C	-3.847476000	-3.126404000	0.622767000
C	-6.469428000	-1.083316000	-1.116866000
C	-7.637549000	-1.518104000	-0.433066000
C	-7.548504000	-2.387986000	0.627381000
C	-6.292956000	-2.894940000	1.024027000
C	-6.538419000	-0.250301000	-2.265601000
C	-5.389473000	0.106927000	-2.921821000
C	-4.120752000	-0.274752000	-2.423234000
C	-3.858337000	3.237103000	2.962152000
C	-3.558494000	4.510327000	2.541627000
C	-2.805669000	4.704805000	1.364082000
H	-1.634785000	4.792973000	-1.038767000
H	-0.100542000	4.789477000	0.958820000

Sum of electronic and thermal Enthalpies= -2762.883147

Sum of electronic and thermal Free Energies= -2763.002197

Charge = 0 Multiplicity = 1

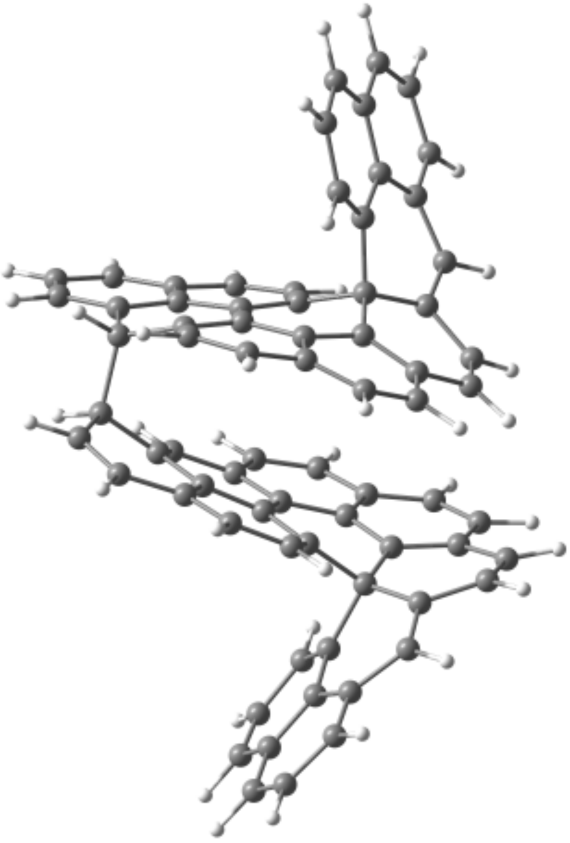

H	1.222539000	-5.158874000	1.691171000	
H	-0.040204000	-3.827436000	3.360397000	
H	-0.816487000	-1.582239000	4.175626000	
H	-0.894310000	0.866329000	3.884528000	
H	-0.482007000	2.831604000	2.474646000	
H	3.459947000	2.339144000	-2.967539000	
H	3.959929000	-0.008210000	-2.414331000	
H	3.974771000	-4.170282000	-2.071282000	
H	2.496058000	-5.254547000	-0.434554000	
H	5.708804000	-2.534491000	-2.310758000	
H	8.504168000	1.515481000	0.941094000	
H	8.986802000	0.860782000	-1.399652000	
H	7.473999000	-0.727822000	-2.586305000	
H	7.086894000	1.327100000	2.970385000	
H	5.060570000	0.416733000	4.076309000	
H	3.401497000	-0.870941000	2.794414000	
H	2.541414000	4.567636000	-2.293724000	
H	0.805292000	5.807241000	-1.097000000	
H	0.994223000	-2.638602000	-3.090928000	
H	1.127405000	-0.443669000	-4.247626000	
H	0.391420000	1.983272000	-4.359109000	
H	-0.544114000	3.905777000	-3.125251000	
H	-4.236499000	0.614534000	3.557197000	
H	-3.596463000	-1.287609000	2.155849000	
H	-1.569413000	-4.474467000	0.463280000	
H	0.121667000	-4.040558000	-1.267989000	
H	-3.854382000	-4.123363000	1.073433000	
H	-8.610900000	-1.156437000	-0.773512000	
H	-8.452251000	-2.718790000	1.143489000	
H	-6.237320000	-3.650187000	1.812225000	
H	-7.515816000	0.077951000	-2.627175000	
H	-5.439127000	0.715345000	-3.827270000	
H	-3.234739000	0.065817000	-2.955709000	
H	-4.417246000	3.072137000	3.886509000	
H	-3.885346000	5.374881000	3.123273000	
H	-2.537352000	5.721132000	1.061264000	

nonsym-(*P*,*S*,*S*,*M*)-(c-**NC**)$_2$

C	3.760557000	1.089908000	1.798686000	- Thermochemistry -
C	3.946359000	-0.310907000	1.734425000	-------------------
C	4.198287000	1.817954000	2.931714000	Temperature 298.150 Kelvin. Pressure 1.00000 Atm.
C	4.275944000	-0.998473000	2.899265000	
C	4.663581000	-0.271343000	4.053084000	Zero-point correction= 0.876074
C	4.690083000	1.103101000	4.049426000	(Hartree/Particle)
C	4.077114000	3.245442000	2.920394000	Thermal correction to Energy= 0.921104
C	3.007522000	1.759611000	0.774193000	Thermal correction to Enthalpy= 0.922049
C	2.808507000	3.143980000	0.845386000	Thermal correction to Gibbs Free Energy= 0.802310
C	3.435326000	3.884315000	1.901856000	Sum of electronic and zero-point Energies= -
C	1.827076000	3.755462000	0.007443000	2762.917316
C	1.010731000	3.003186000	-0.790118000	Sum of electronic and thermal Energies= -2762.872286
C	1.274845000	1.598651000	-0.947277000	Sum of electronic and thermal Enthalpies= -
C	2.278679000	0.973030000	-0.179051000	2762.871342
C	0.502378000	0.828324000	-1.861802000	Sum of electronic and thermal Free Energies= -
C	0.664597000	-0.541787000	-1.884261000	2762.991080
C	1.633726000	-1.167765000	-1.081450000	
C	2.481026000	-0.433043000	-0.280279000	Charge = 0 Multiplicity = 1
C	3.745993000	-1.014495000	0.400398000	
C	3.717966000	-2.534395000	0.544068000	
C	3.735232000	-3.144557000	1.862203000	
C	4.098713000	-2.445566000	2.952908000	
C	4.858057000	-0.658197000	-0.628657000	

C	4.933836000	-1.477504000	-1.796757000	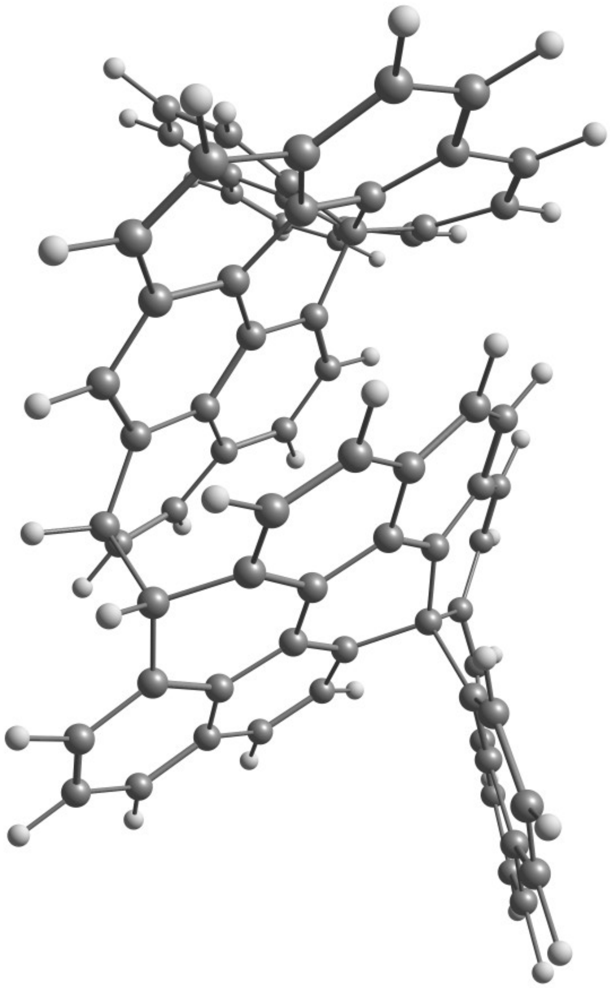
C	4.315567000	-2.769280000	-1.825654000	
C	3.859960000	-3.310787000	-0.553809000	
C	5.681329000	-1.045844000	-2.924264000	
C	5.675621000	-1.840641000	-4.103124000	
C	5.007823000	-3.041439000	-4.138498000	
C	4.355392000	-3.522947000	-2.983610000	
C	6.427658000	0.160107000	-2.834110000	
C	6.439610000	0.872373000	-1.663363000	
C	5.648532000	0.467251000	-0.561090000	
C	-0.404414000	1.529908000	-2.767490000	
C	-0.732944000	2.809647000	-2.564882000	
C	-0.289587000	3.549641000	-1.331510000	
C	-1.246483000	-0.020567000	1.149621000	
C	-1.697696000	-1.310019000	0.773042000	
C	-0.154669000	0.112068000	2.049397000	
C	-0.862738000	-2.396766000	0.999243000	
C	0.250946000	-2.258314000	1.869799000	
C	0.551466000	-1.051834000	2.446889000	
C	0.223835000	1.416041000	2.462788000	
C	-1.787263000	1.135711000	0.509523000	
C	-1.234943000	2.376065000	0.773705000	
C	-0.314154000	2.514237000	1.843919000	
C	-1.451565000	3.516947000	-0.185587000	
C	-2.780961000	3.438037000	-0.884970000	
C	-3.308141000	2.146995000	-1.186246000	
C	-2.802861000	0.973770000	-0.537569000	
C	-4.348503000	2.034098000	-2.143039000	
C	-4.760531000	0.733149000	-2.534051000	
C	-4.248514000	-0.379316000	-1.919609000	
C	-3.321601000	-0.274324000	-0.851051000	
C	-3.064459000	-1.456565000	0.126430000	
C	-3.259018000	-2.843041000	-0.491576000	
C	-2.141889000	-3.772121000	-0.564146000	
C	-1.068056000	-3.620295000	0.231904000	
C	-4.208940000	-1.235072000	1.163334000	
C	-5.515084000	-1.685333000	0.799674000	
C	-5.696696000	-2.627966000	-0.263650000	
C	-4.504531000	-3.289315000	-0.772076000	
C	-6.651158000	-1.247679000	1.532618000	
C	-7.946820000	-1.649108000	1.106589000	
C	-8.104110000	-2.496945000	0.036468000	
C	-6.972619000	-3.013567000	-0.629664000	
C	-6.458456000	-0.432964000	2.680027000	
C	-5.189815000	-0.101216000	3.078847000	
C	-4.064248000	-0.496843000	2.317786000	
C	-4.918710000	3.211584000	-2.695305000	
C	-4.467956000	4.450527000	-2.309407000	
C	-3.381158000	4.560317000	-1.412288000	
H	-1.359047000	4.470546000	0.355926000	
H	-0.170491000	4.616713000	-1.575141000	
H	4.952708000	-0.824449000	4.950099000	
H	5.034234000	1.654051000	4.927931000	
H	4.482200000	3.812810000	3.761914000	
H	3.320771000	4.971099000	1.919802000	
H	1.629769000	4.824693000	0.132099000	
H	0.046972000	-1.149497000	-2.549666000	
H	1.730519000	-2.251163000	-1.130108000	
H	3.580729000	-4.225146000	1.921930000	
H	4.226445000	-2.944387000	3.916624000	
H	3.798558000	-4.399078000	-0.457904000	

H	6.221764000	-1.484182000	-4.979925000	
H	5.011527000	-3.645298000	-5.048275000	
H	3.900553000	-4.516793000	-2.992515000	
H	7.004296000	0.495030000	-3.699588000	
H	7.040008000	1.780365000	-1.577468000	
H	5.655229000	1.086305000	0.334555000	
H	-0.808183000	0.978327000	-3.620007000	
H	-1.411778000	3.319328000	-3.251363000	
H	0.871947000	-3.133019000	2.076820000	
H	1.389126000	-0.958712000	3.141610000	
H	1.001528000	1.532315000	3.221651000	
H	0.031936000	3.512553000	2.117762000	
H	-5.508855000	0.626417000	-3.322929000	
H	-4.591357000	-1.362675000	-2.235528000	
H	-2.271333000	-4.678475000	-1.160958000	
H	-0.294937000	-4.391414000	0.280165000	
H	-4.631563000	-4.269525000	-1.241439000	
H	-8.817552000	-1.278408000	1.652964000	
H	-9.103672000	-2.801715000	-0.280523000	
H	-7.101825000	-3.749322000	-1.427579000	
H	-7.330211000	-0.095664000	3.245739000	
H	-5.034339000	0.496376000	3.979469000	
H	-3.078658000	-0.175677000	2.650041000	
H	-5.727069000	3.115034000	-3.424193000	
H	-4.924742000	5.354469000	-2.718040000	
H	-2.980711000	5.548399000	-1.168522000	
nonsym-(*P*,*S*,*R*,*P*)-(c-**NC**)$_2$				
C	3.674021000	-2.472260000	-0.678727000	- Thermochemistry -
C	5.025439000	-2.055098000	-0.679055000	-------------------
C	3.346791000	-3.846649000	-0.585648000	Temperature 298.150 Kelvin. Pressure 1.00000 Atm.
C	6.024385000	-3.003402000	-0.874210000	
C	5.692668000	-4.381575000	-0.829647000	Zero-point correction= 0.876126
C	4.396721000	-4.794818000	-0.628328000	(Hartree/Particle)
C	1.966599000	-4.219982000	-0.487314000	Thermal correction to Energy= 0.921469
C	2.627904000	-1.507939000	-0.869669000	Thermal correction to Enthalpy= 0.922413
C	1.288212000	-1.911268000	-0.847631000	Thermal correction to Gibbs Free Energy= 0.800468
C	0.978111000	-3.286008000	-0.586709000	Sum of electronic and zero-point Energies= -
C	0.269707000	-0.949239000	-1.119630000	2762.921673
C	0.574024000	0.322828000	-1.512859000	Sum of electronic and thermal Energies= -2762.876330
C	1.947143000	0.692649000	-1.703725000	Sum of electronic and thermal Enthalpies= -
C	2.971621000	-0.178614000	-1.285789000	2762.875386
C	2.275858000	1.917399000	-2.353831000	Sum of electronic and thermal Free Energies= -
C	3.609613000	2.235569000	-2.528853000	2762.997331
C	4.628667000	1.419294000	-1.998470000	
C	4.330352000	0.242598000	-1.344054000	Charge = 0 Multiplicity = 1
C	5.331744000	-0.576941000	-0.490435000	
C	6.794105000	-0.237797000	-0.762325000	
C	7.694512000	-1.258771000	-1.269723000	
C	7.370667000	-2.564292000	-1.228214000	
C	4.994158000	-0.076918000	0.944554000	
C	5.474702000	1.223192000	1.288703000	
C	6.540958000	1.825438000	0.544884000	
C	7.278010000	0.962268000	-0.368142000	
C	4.943267000	1.906264000	2.414127000	
C	5.376518000	3.233327000	2.684486000	
C	6.343582000	3.827054000	1.909018000	
C	6.953036000	3.106810000	0.858948000	
C	4.011765000	1.232065000	3.249444000	
C	3.666168000	-0.067106000	2.979722000	
C	4.147520000	-0.720763000	1.819210000	

C	1.181426000	2.727036000	-2.893848000
C	-0.099474000	2.465541000	-2.597041000
C	-0.473832000	1.403221000	-1.600064000
C	-2.518491000	-0.462674000	1.986930000
C	-3.848142000	-0.935850000	2.133313000
C	-1.480578000	-0.968761000	2.811878000
C	-4.177804000	-1.683098000	3.255075000
C	-3.141989000	-2.158309000	4.107887000
C	-1.826760000	-1.861349000	3.861744000
C	-0.151564000	-0.521466000	2.583661000
C	-2.234563000	0.585846000	1.061767000
C	-0.933013000	1.014399000	0.875569000
C	0.114993000	0.425709000	1.626835000
C	-0.656428000	2.070665000	-0.160721000
C	-1.764479000	3.095982000	-0.209854000
C	-3.103120000	2.668333000	0.031927000
C	-3.365736000	1.355350000	0.528425000
C	-4.181328000	3.564497000	-0.191492000
C	-5.501930000	3.096036000	0.029408000
C	-5.736931000	1.805771000	0.436923000
C	-4.670646000	0.910078000	0.686388000
C	-4.868018000	-0.585259000	1.058816000
C	-6.297436000	-0.932197000	1.470252000
C	-6.569812000	-1.437096000	2.804038000
C	-5.581184000	-1.882205000	3.601431000
C	-4.592246000	-1.305747000	-0.288219000
C	-5.622417000	-1.213532000	-1.272300000
C	-6.962203000	-0.877082000	-0.891388000
C	-7.271235000	-0.926537000	0.531134000
C	-5.346169000	-1.533994000	-2.627327000
C	-6.366527000	-1.351383000	-3.600001000
C	-7.624739000	-0.939997000	-3.228179000
C	-7.933624000	-0.740704000	-1.865622000
C	-4.066007000	-2.053604000	-2.961485000
C	-3.136238000	-2.271124000	-1.977512000
C	-3.394974000	-1.888360000	-0.637583000
C	-3.899545000	4.894730000	-0.603734000
C	-2.601251000	5.308006000	-0.772923000
C	-1.531364000	4.402695000	-0.579025000
H	0.298878000	2.568749000	0.066001000
H	-1.448334000	0.958948000	-1.859604000
H	6.490866000	-5.117967000	-0.951309000
H	4.159803000	-5.858807000	-0.553613000
H	1.718157000	-5.275162000	-0.349734000
H	-0.071647000	-3.582637000	-0.518450000
H	-0.774472000	-1.234786000	-0.990914000
H	3.881189000	3.147935000	-3.065523000
H	5.665449000	1.740494000	-2.094719000
H	8.699038000	-0.945700000	-1.565307000
H	8.103104000	-3.326430000	-1.504959000
H	8.313535000	1.230566000	-0.597784000
H	4.935952000	3.769223000	3.528724000
H	6.672683000	4.845204000	2.126841000
H	7.781837000	3.554771000	0.304819000
H	3.602032000	1.751148000	4.119071000
H	2.992305000	-0.605255000	3.649711000
H	3.806567000	-1.734811000	1.615714000
H	1.439227000	3.543989000	-3.573256000
H	-0.899875000	3.071575000	-3.025746000
H	-3.414245000	-2.777254000	4.966411000
H	-1.037235000	-2.263564000	4.500993000

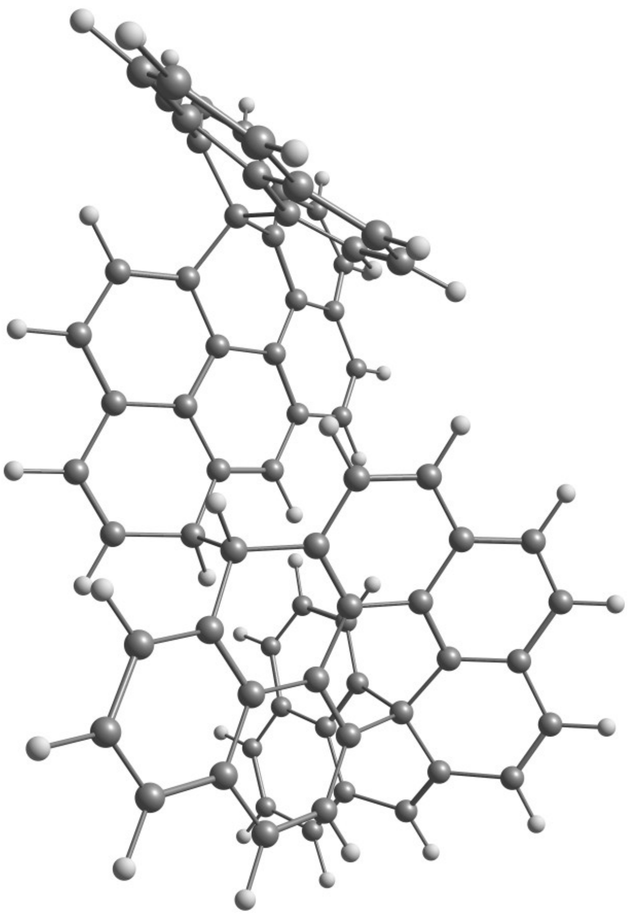

H	0.658045000	-0.933542000	3.189701000
H	1.144841000	0.750583000	1.454442000
H	-6.340123000	3.778486000	-0.130396000
H	-6.762688000	1.474784000	0.591771000
H	-7.616081000	-1.564970000	3.092754000
H	-5.811824000	-2.364231000	4.554511000
H	-8.306116000	-1.131357000	0.820958000
H	-6.137915000	-1.557814000	-4.648396000
H	-8.402972000	-0.807016000	-3.982527000
H	-8.960056000	-0.507530000	-1.571322000
H	-3.847557000	-2.305612000	-4.001787000
H	-2.171056000	-2.717371000	-2.228049000
H	-2.618190000	-2.036222000	0.112340000
H	-4.730592000	5.584179000	-0.771293000
H	-2.386594000	6.336586000	-1.070696000
H	-0.504957000	4.735516000	-0.750518000

nonsym-(*P*,*S*,*R*,*M*)-(c-**NC**)$_2$

- Thermochemistry -

Temperature 298.150 Kelvin. Pressure 1.00000 Atm.

Zero-point correction= 0.876973 (Hartree/Particle)
Thermal correction to Energy= 0.921977
Thermal correction to Enthalpy= 0.922921
Thermal correction to Gibbs Free Energy= 0.804004
Sum of electronic and zero-point Energies= -2762.926121
Sum of electronic and thermal Energies= -2762.881117
Sum of electronic and thermal Enthalpies= -2762.880173
Sum of electronic and thermal Free Energies= -2762.999090

Charge = 0 Multiplicity = 1

C	-2.228394000	1.670493000	1.185868000
C	-3.206818000	1.988122000	0.218506000
C	-1.816992000	2.641422000	2.131891000
C	-3.556535000	3.321594000	0.021929000
C	-3.136406000	4.295512000	0.961840000
C	-2.332190000	3.953841000	2.023833000
C	-0.880357000	2.262984000	3.147831000
C	-1.601370000	0.378293000	1.174638000
C	-0.678844000	0.045494000	2.169276000
C	-0.354698000	1.007163000	3.182385000
C	-0.041283000	-1.226472000	2.132282000
C	-0.164905000	-2.077281000	1.064765000
C	-1.048875000	-1.702943000	-0.010608000
C	-1.816292000	-0.516512000	0.077550000
C	-1.220433000	-2.554108000	-1.140814000
C	-2.035274000	-2.142228000	-2.176808000
C	-2.803210000	-0.966307000	-2.076729000
C	-2.769604000	-0.197103000	-0.934330000
C	-3.847379000	0.858885000	-0.573073000
C	-4.646046000	1.368766000	-1.770702000
C	-4.629577000	2.782631000	-2.108399000
C	-4.227918000	3.706264000	-1.215521000
C	-4.815436000	0.011818000	0.306649000
C	-5.693689000	-0.878154000	-0.383818000
C	-5.957172000	-0.707441000	-1.781600000
C	-5.526521000	0.545830000	-2.384805000
C	-6.367123000	-1.907912000	0.324741000
C	-7.172604000	-2.830938000	-0.397020000
C	-7.349649000	-2.698462000	-1.753542000
C	-6.767679000	-1.612050000	-2.441272000
C	-6.228941000	-1.974215000	1.737508000
C	-5.476041000	-1.038119000	2.397493000
C	-4.761757000	-0.047414000	1.681304000
C	-0.610647000	-3.881819000	-1.120988000
C	0.129891000	-4.304293000	-0.092053000
C	0.539894000	-3.429799000	1.056491000
C	4.101153000	-1.393335000	-1.430066000
C	4.604563000	-0.075937000	-1.575132000
C	4.444918000	-2.394996000	-2.373483000
C	5.661175000	0.147150000	-2.447673000
C	6.039885000	-0.873389000	-3.363515000
C	5.404883000	-2.087608000	-3.373747000
C	3.862700000	-3.682027000	-2.234109000

C	3.334387000	-1.737640000	-0.273290000
C	2.824336000	-3.014289000	-0.135446000
C	3.062981000	-3.970251000	-1.158427000
C	2.126423000	-3.429795000	1.138415000
C	2.621913000	-2.652237000	2.333445000
C	3.102583000	-1.322186000	2.161432000
C	3.354360000	-0.798500000	0.856397000
C	3.337269000	-0.511459000	3.303691000
C	3.771687000	0.824307000	3.104427000
C	3.977067000	1.317477000	1.841296000
C	3.779929000	0.511446000	0.693702000
C	3.971003000	1.037766000	-0.754421000
C	4.782626000	2.331785000	-0.822249000
C	6.070286000	2.357032000	-1.492466000
C	6.434925000	1.378861000	-2.341041000
C	2.528697000	1.361434000	-1.234340000
C	1.960314000	2.589590000	-0.782511000
C	2.782844000	3.618528000	-0.217909000
C	4.217811000	3.492241000	-0.418070000
C	0.577745000	2.848052000	-0.977254000
C	0.020612000	4.049982000	-0.468515000
C	0.807943000	4.979059000	0.168089000
C	2.200986000	4.778762000	0.259800000
C	-0.207392000	1.908805000	-1.694779000
C	0.372359000	0.774629000	-2.197668000
C	1.739447000	0.497927000	-1.963493000
C	3.137457000	-1.060739000	4.597842000
C	2.744691000	-2.369399000	4.745255000
C	2.486809000	-3.164452000	3.606372000
H	2.373090000	-4.490949000	1.308042000
H	0.240545000	-3.941102000	1.989103000
H	-3.455007000	5.331770000	0.823331000
H	-2.033867000	4.705984000	2.757792000
H	-0.584392000	3.008205000	3.889843000
H	0.368411000	0.725877000	3.951972000
H	0.572078000	-1.498770000	2.989336000
H	-2.142935000	-2.775245000	-3.061074000
H	-3.481217000	-0.710723000	-2.889863000
H	-5.096560000	3.085273000	-3.048961000
H	-4.347560000	4.771831000	-1.426273000
H	-6.072339000	0.899180000	-3.264839000
H	-7.658505000	-3.643490000	0.148704000
H	-7.972493000	-3.411093000	-2.298201000
H	-6.983505000	-1.464155000	-3.502532000
H	-6.746558000	-2.764719000	2.286127000
H	-5.395668000	-1.064051000	3.486152000
H	-4.140217000	0.649255000	2.241686000
H	-0.835689000	-4.555318000	-1.952055000
H	0.509829000	-5.329932000	-0.082833000
H	6.845369000	-0.667176000	-4.072730000
H	5.672150000	-2.850329000	-4.108924000
H	4.092696000	-4.452950000	-2.973413000
H	2.656499000	-4.976922000	-1.048159000
H	3.951908000	1.459308000	3.975126000
H	4.320236000	2.343547000	1.720198000
H	6.673201000	3.263644000	-1.396930000
H	7.347269000	1.467416000	-2.935637000
H	4.812206000	4.411043000	-0.420297000
H	-1.049245000	4.220384000	-0.594000000
H	0.363472000	5.895367000	0.562583000
H	2.837517000	5.566695000	0.671029000

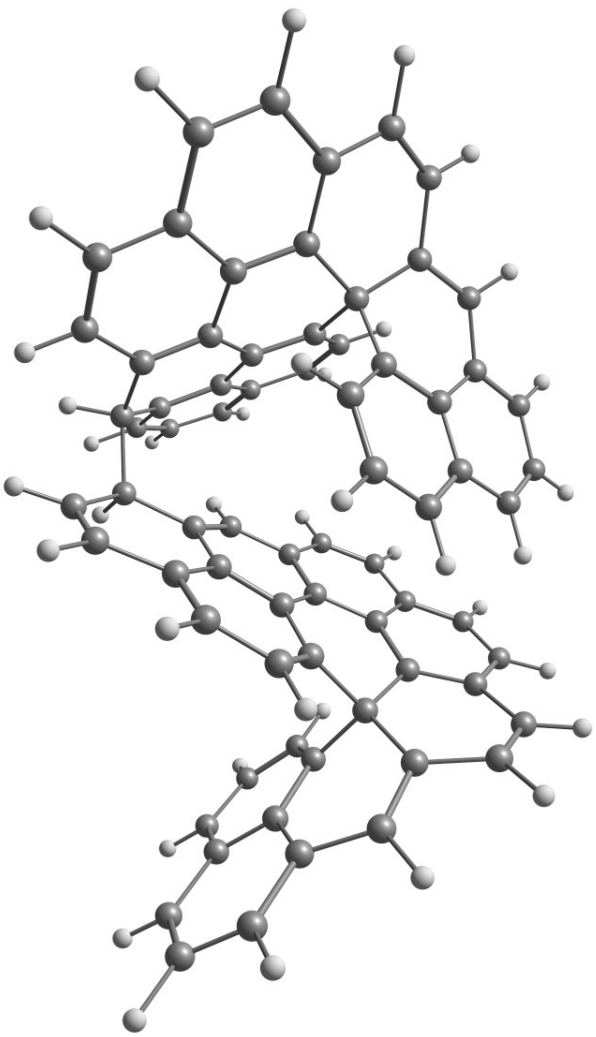

H	-1.270510000	2.109598000	-1.846399000
H	-0.226047000	0.054769000	-2.759278000
H	2.149254000	-0.427039000	-2.363797000
H	3.318093000	-0.431672000	5.472983000
H	2.614480000	-2.796094000	5.741981000
H	2.131001000	-4.190226000	3.738650000

nonsym-(*P*,*R*,*S*,*P*)-(c-**NC**)$_2$

C	-1.391855000	1.288833000	1.562904000
C	-2.277458000	2.053430000	0.771315000
C	-0.575200000	1.918489000	2.534213000
C	-2.135392000	3.437378000	0.746441000
C	-1.294763000	4.068119000	1.695963000
C	-0.576573000	3.331330000	2.607019000
C	0.220197000	1.099232000	3.397580000
C	-1.271752000	-0.124255000	1.342655000
C	-0.439142000	-0.891142000	2.164216000
C	0.260443000	-0.253513000	3.239513000
C	-0.187649000	-2.256883000	1.830673000
C	-0.644005000	-2.798676000	0.661478000
C	-1.565850000	-2.047174000	-0.148579000
C	-1.898363000	-0.717767000	0.196508000
C	-2.181307000	-2.655373000	-1.276991000
C	-3.004009000	-1.890727000	-2.081452000
C	-3.295119000	-0.554232000	-1.761107000
C	-2.811006000	0.023798000	-0.605062000
C	-3.373362000	1.338052000	-0.003987000
C	-4.050889000	2.251676000	-1.023622000
C	-3.550587000	3.599455000	-1.241364000
C	-2.748619000	4.195149000	-0.339493000
C	-4.484348000	0.789811000	0.942903000
C	-5.708568000	0.382394000	0.329086000
C	-6.046376000	0.820123000	-0.992462000
C	-5.241351000	1.893921000	-1.556847000
C	-6.637669000	-0.413888000	1.049656000
C	-7.812018000	-0.872517000	0.392231000
C	-8.080856000	-0.505366000	-0.904637000
C	-7.211197000	0.372169000	-1.586663000
C	-6.373976000	-0.714988000	2.413133000
C	-5.250379000	-0.217248000	3.019696000
C	-4.300486000	0.530644000	2.282856000
C	-1.930660000	-4.072706000	-1.533967000
C	-0.982718000	-4.750861000	-0.875047000
C	-0.076417000	-4.094238000	0.123314000
C	1.174398000	-0.309852000	-1.818807000
C	1.455134000	1.006048000	-1.374375000
C	0.301634000	-0.511076000	-2.919535000
C	0.608400000	2.034918000	-1.760850000
C	-0.345589000	1.811676000	-2.791442000
C	-0.435987000	0.595005000	-3.415492000
C	0.178497000	-1.824973000	-3.440456000
C	1.665312000	-1.431459000	-1.085544000
C	1.320048000	-2.710712000	-1.489880000
C	0.695691000	-2.891995000	-2.751492000
C	1.387457000	-3.855894000	-0.514108000
C	2.369851000	-3.636911000	0.606336000
C	2.732908000	-2.313528000	0.987179000
C	2.382334000	-1.190179000	0.172981000
C	3.456100000	-2.114259000	2.194593000
C	3.730104000	-0.783823000	2.604092000
C	3.377629000	0.277833000	1.815255000

- Thermochemistry -

Temperature 298.150 Kelvin. Pressure 1.00000 Atm.

Zero-point correction= 0.876014 (Hartree/Particle)
Thermal correction to Energy= 0.921028
Thermal correction to Enthalpy= 0.921972
Thermal correction to Gibbs Free Energy= 0.802919
Sum of electronic and zero-point Energies= -2762.929107
Sum of electronic and thermal Energies= -2762.884093
Sum of electronic and thermal Enthalpies= -2762.883149
Sum of electronic and thermal Free Energies= -2763.002202

Charge = 0 Multiplicity = 1

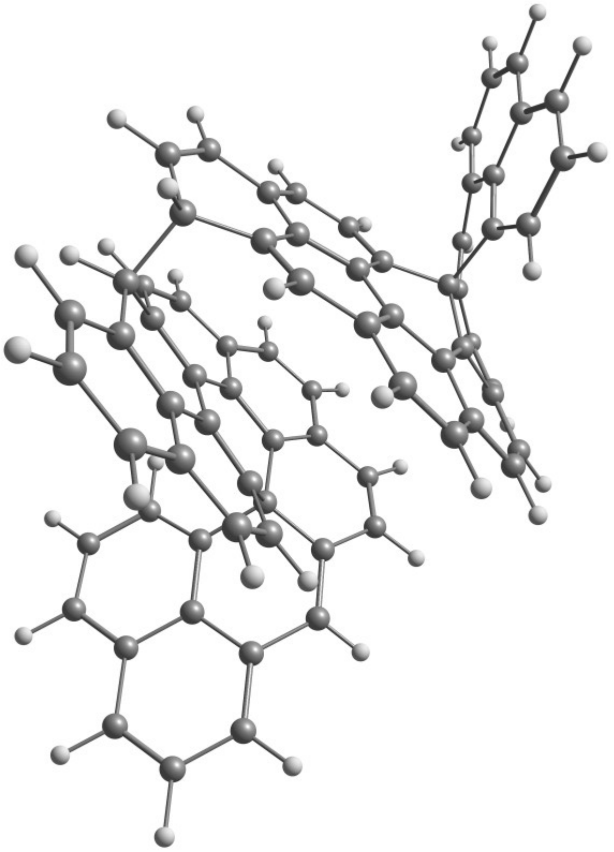

C	2.754644000	0.090605000	0.556901000	
C	2.670044000	1.231908000	-0.493487000	
C	2.698647000	2.640323000	0.100031000	
C	1.570765000	3.534209000	-0.092348000	
C	0.650490000	3.302003000	-1.043923000	
C	4.004756000	1.023886000	-1.272733000	
C	5.196610000	1.507926000	-0.650374000	
C	5.130970000	2.476451000	0.403453000	
C	3.847781000	3.126388000	0.622815000	
C	6.469646000	1.083130000	-1.116761000	
C	7.637783000	1.517888000	-0.432969000	
C	7.548778000	2.387818000	0.627442000	
C	6.293252000	2.894850000	1.024060000	
C	6.538605000	0.250089000	-2.265471000	
C	5.389642000	-0.107086000	-2.921698000	
C	4.120941000	0.274676000	-2.423141000	
C	3.858053000	-3.237100000	2.962352000	
C	3.558158000	-4.510317000	2.541822000	
C	2.805444000	-4.704762000	1.364209000	
H	0.100464000	-4.789431000	0.958658000	
H	1.634885000	-4.792885000	-1.038787000	
H	-1.222616000	5.158828000	1.691072000	
H	0.040218000	3.827371000	3.360214000	
H	0.816520000	1.582190000	4.175391000	
H	0.894269000	-0.866390000	3.884329000	
H	0.481889000	-2.831680000	2.474463000	
H	-3.459959000	-2.339077000	-2.967779000	
H	-3.959997000	0.008246000	-2.414499000	
H	-3.975100000	4.170293000	-2.071201000	
H	-2.496289000	5.254532000	-0.434574000	
H	-5.709086000	2.534508000	-2.310681000	
H	-8.504409000	-1.515408000	0.941260000	
H	-8.987125000	-0.860693000	-1.399466000	
H	-7.474334000	0.727876000	-2.586174000	
H	-7.087074000	-1.327052000	2.970486000	
H	-5.060672000	-0.416778000	4.076333000	
H	-3.401581000	0.870795000	2.794371000	
H	-2.541290000	-4.567595000	-2.294058000	
H	-0.805178000	-5.807163000	-1.097274000	
H	-0.993988000	2.638769000	-3.090810000	
H	-1.127212000	0.443867000	-4.247577000	
H	-0.391243000	-1.983098000	-4.359131000	
H	0.544275000	-3.905618000	-3.125339000	
H	4.236521000	-0.614570000	3.557306000	
H	3.596808000	1.287604000	2.155843000	
H	1.569716000	4.474490000	0.463437000	
H	-0.121419000	4.040644000	-1.267803000	
H	3.854727000	4.123342000	1.073493000	
H	8.611114000	1.156148000	-0.773393000	
H	8.452538000	2.718600000	1.143539000	
H	6.237647000	3.650134000	1.812223000	
H	7.515983000	-0.078242000	-2.627024000	
H	5.439279000	-0.715522000	-3.827136000	
H	3.234917000	-0.065848000	-2.955626000	
H	4.416871000	-3.072159000	3.886769000	
H	3.884905000	-5.374877000	3.123515000	
H	2.537119000	-5.721075000	1.061355000	
nonsym-(*P*,*R*,*S*,*M*)-(c-**NC**)$_2$				
C	5.590155000	-1.579101000	0.487451000	- Thermochemistry -
C	6.121337000	-0.508431000	-0.269372000	-------------------

C	6.454086000	-2.469957000	1.169600000
C	7.475883000	-0.518067000	-0.587669000
C	8.337952000	-1.436283000	0.063828000
C	7.849886000	-2.345042000	0.972410000
C	5.878922000	-3.495739000	1.987808000
C	4.177998000	-1.845609000	0.457573000
C	3.654604000	-2.924598000	1.182169000
C	4.531770000	-3.699636000	2.010838000
C	2.281507000	-3.278543000	1.011121000
C	1.500172000	-2.674684000	0.065321000
C	2.063650000	-1.643329000	-0.762077000
C	3.360278000	-1.152885000	-0.497172000
C	1.318134000	-1.121513000	-1.857183000
C	1.862315000	-0.093319000	-2.599594000
C	3.116833000	0.456170000	-2.274142000
C	3.868838000	-0.041047000	-1.230651000
C	5.186637000	0.603758000	-0.724005000
C	5.837076000	1.520945000	-1.758688000
C	7.151537000	1.206511000	-2.290837000
C	7.962596000	0.330092000	-1.670944000
C	4.744716000	1.501420000	0.469598000
C	4.147009000	2.758475000	0.149547000
C	4.256393000	3.304729000	-1.170748000
C	5.268104000	2.715756000	-2.037327000
C	3.504636000	3.524314000	1.158375000
C	2.818068000	4.714800000	0.791620000
C	2.835852000	5.161839000	-0.507947000
C	3.595014000	4.477282000	-1.482290000
C	3.568258000	3.077871000	2.505724000
C	4.233419000	1.918567000	2.812412000
C	4.807892000	1.121348000	1.792828000
C	0.044376000	-1.752516000	-2.204361000
C	-0.528378000	-2.663234000	-1.409827000
C	0.027712000	-2.991607000	-0.052998000
C	-4.413849000	-2.309920000	-0.186301000
C	-5.328310000	-1.468375000	-0.871945000
C	-4.689898000	-3.697432000	-0.060960000
C	-6.304801000	-2.046718000	-1.672839000
C	-6.548948000	-3.444262000	-1.582994000
C	-5.809346000	-4.238052000	-0.745892000
C	-3.801459000	-4.500192000	0.703789000
C	-3.162090000	-1.786443000	0.271636000
C	-2.261117000	-2.629043000	0.898973000
C	-2.631149000	-3.970308000	1.180712000
C	-0.818335000	-2.227252000	1.074967000
C	-0.582499000	-0.746712000	0.967098000
C	-1.476035000	0.053000000	0.198349000
C	-2.784522000	-0.421939000	-0.125656000
C	-1.082746000	1.363472000	-0.185364000
C	-1.971753000	2.119723000	-0.990992000
C	-3.232837000	1.658855000	-1.270345000
C	-3.685821000	0.413016000	-0.772474000
C	-5.192314000	0.036108000	-0.717466000
C	-6.067195000	0.805265000	-1.706834000
C	-6.793113000	0.094518000	-2.745140000
C	-6.993561000	-1.233252000	-2.669372000
C	-5.576012000	0.513966000	0.714758000
C	-5.769657000	1.918133000	0.891578000
C	-6.011503000	2.770052000	-0.234336000
C	-6.315399000	2.118545000	-1.499540000
C	-5.793462000	2.477253000	2.197000000

Temperature 298.150 Kelvin. Pressure 1.00000 Atm.

Zero-point correction= 0.876147 (Hartree/Particle)
Thermal correction to Energy= 0.921379
Thermal correction to Enthalpy= 0.922323
Thermal correction to Gibbs Free Energy= 0.801398
Sum of electronic and zero-point Energies= -2762.917692
Sum of electronic and thermal Energies= -2762.872460
Sum of electronic and thermal Enthalpies= -2762.871516
Sum of electronic and thermal Free Energies= -2762.992441

Charge = 0 Multiplicity = 1

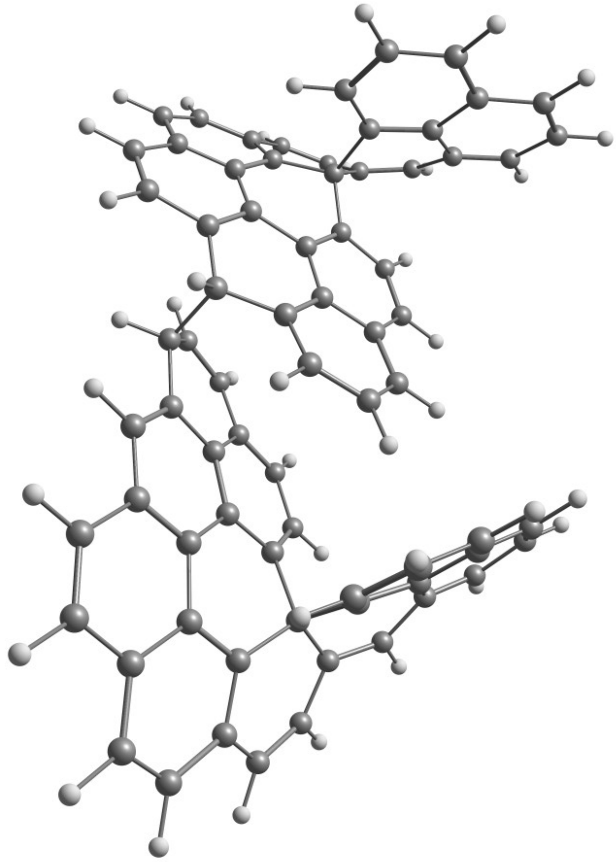

C	-5.908068000	3.886701000	2.342949000	
C	-6.059455000	4.697592000	1.243378000	
C	-6.147425000	4.132801000	-0.046653000	
C	-5.721278000	1.608263000	3.318636000	
C	-5.660746000	0.251889000	3.130927000	
C	-5.582470000	-0.297016000	1.828340000	
C	0.165419000	1.874078000	0.257489000	
C	0.947580000	1.133844000	1.107839000	
C	0.567194000	-0.175045000	1.466849000	
H	-0.434865000	-2.600834000	2.038258000	
H	-0.121303000	-4.063784000	0.150938000	
H	9.406025000	-1.407440000	-0.165407000	
H	8.527201000	-3.020954000	1.499777000	
H	6.545529000	-4.137373000	2.569241000	
H	4.106407000	-4.504746000	2.615026000	
H	1.865793000	-4.077172000	1.633226000	
H	1.309125000	0.305561000	-3.453070000	
H	3.493699000	1.285940000	-2.870227000	
H	7.518933000	1.810684000	-3.124101000	
H	8.995167000	0.197495000	-2.002749000	
H	5.701350000	3.353028000	-2.814054000	
H	2.282558000	5.272215000	1.563828000	
H	2.304687000	6.075907000	-0.781321000	
H	3.694557000	4.899052000	-2.485655000	
H	3.083578000	3.669079000	3.286002000	
H	4.303233000	1.582373000	3.848838000	
H	5.293479000	0.189206000	2.077380000	
H	-0.422293000	-1.469332000	-3.151030000	
H	-1.473291000	-3.129866000	-1.697076000	
H	-7.350259000	-3.876356000	-2.187472000	
H	-6.032165000	-5.302942000	-0.645916000	
H	-4.044089000	-5.552503000	0.870110000	
H	-1.932240000	-4.604906000	1.732778000	
H	-1.654065000	3.100027000	-1.354262000	
H	-3.908769000	2.277961000	-1.857751000	
H	-7.291571000	0.689604000	-3.514459000	
H	-7.644820000	-1.740107000	-3.385498000	
H	-6.910163000	2.677047000	-2.228578000	
H	-5.885330000	4.314458000	3.348246000	
H	-6.150417000	5.778752000	1.367017000	
H	-6.354232000	4.774579000	-0.906887000	
H	-5.739205000	2.034603000	4.324430000	
H	-5.644656000	-0.422244000	3.989815000	
H	-5.493773000	-1.377359000	1.728666000	
H	0.483503000	2.870720000	-0.059697000	
H	1.886075000	1.534266000	1.484284000	
H	1.237057000	-0.767734000	2.093989000	

nonsym-(*P*,*R*,*R*,*P*)-(c-**NC**)$_2$

C	4.698961000	-1.816191000	-1.524783000	- Thermochemistry -
C	5.901443000	-1.303686000	-0.983375000	-------------------
C	4.676789000	-3.088083000	-2.146894000	Temperature 298.150 Kelvin. Pressure 1.00000 Atm.
C	7.104982000	-1.918830000	-1.313506000	
C	7.087096000	-3.172505000	-1.976181000	Zero-point correction= 0.875843
C	5.901822000	-3.773020000	-2.328925000	(Hartree/Particle)
C	3.422158000	-3.610901000	-2.600179000	Thermal correction to Energy= 0.921155
C	3.514806000	-1.004424000	-1.534880000	Thermal correction to Enthalpy= 0.922100
C	2.319931000	-1.510681000	-2.059535000	Thermal correction to Gibbs Free Energy= 0.800147
C	2.284907000	-2.861653000	-2.535673000	Sum of electronic and zero-point Energies= -
C	1.173738000	-0.661407000	-2.124460000	2762.909122
C	1.246978000	0.661281000	-1.791793000	Sum of electronic and thermal Energies= -2762.863809

C	2.514062000	1.228064000	-1.428028000	Sum of electronic and thermal Enthalpies= -2762.862865
C	3.619104000	0.386323000	-1.197309000	
C	2.667653000	2.642049000	-1.345235000	Sum of electronic and thermal Free Energies= -2762.984818
C	3.904949000	3.154346000	-1.002139000	
C	4.972209000	2.304444000	-0.650972000	
C	4.836958000	0.932582000	-0.701863000	Charge = 0 Multiplicity = 1
C	5.834667000	-0.079291000	-0.082072000	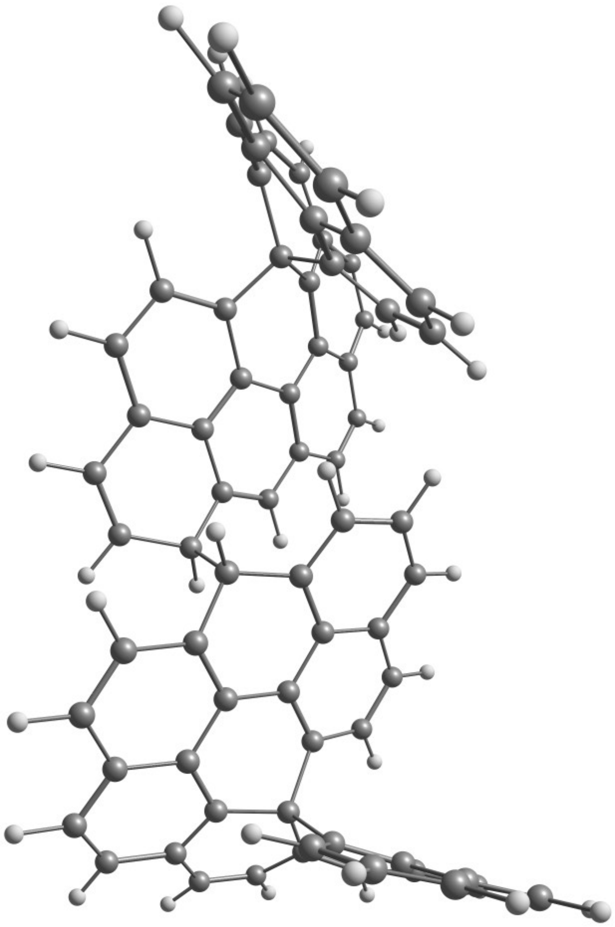
C	7.210554000	0.512832000	0.210019000	
C	8.388751000	-0.023828000	-0.448789000	
C	8.360788000	-1.214327000	-1.075111000	
C	5.155592000	-0.381090000	1.286132000	
C	5.274233000	0.630949000	2.287164000	
C	6.291585000	1.635133000	2.191001000	
C	7.343722000	1.415623000	1.208344000	
C	4.424595000	0.614035000	3.424381000	
C	4.504256000	1.679580000	4.362624000	
C	5.433522000	2.680655000	4.209991000	
C	6.353643000	2.637739000	3.140317000	
C	3.536901000	-0.481344000	3.601655000	
C	3.533281000	-1.512868000	2.698524000	
C	4.333794000	-1.458747000	1.531214000	
C	1.535242000	3.478965000	-1.745116000	
C	0.314844000	2.959947000	-1.942836000	
C	0.017232000	1.516699000	-1.637219000	
C	-4.263337000	2.128325000	-0.717423000	
C	-5.450473000	1.425371000	-1.049784000	
C	-4.223865000	3.543187000	-0.835481000	
C	-6.423622000	2.072659000	-1.799897000	
C	-6.356264000	3.482821000	-1.968813000	
C	-5.325100000	4.209990000	-1.433674000	
C	-3.059894000	4.225370000	-0.391490000	
C	-3.075193000	1.408965000	-0.377336000	
C	-1.904221000	2.104370000	-0.121258000	
C	-1.940207000	3.520619000	-0.030023000	
C	-0.579608000	1.383758000	-0.140881000	
C	-0.699788000	-0.068814000	0.219955000	
C	-1.882082000	-0.769296000	-0.156795000	
C	-3.085984000	-0.057768000	-0.461490000	
C	-1.881868000	-2.189718000	-0.146610000	
C	-3.046985000	-2.861956000	-0.595966000	
C	-4.191952000	-2.164457000	-0.883870000	
C	-4.256274000	-0.758037000	-0.728090000	
C	-5.610466000	-0.009563000	-0.579692000	
C	-6.798264000	-0.707672000	-1.243696000	
C	-7.530774000	-0.045088000	-2.310088000	
C	-7.436524000	1.283834000	-2.493623000	
C	-5.822768000	-0.089862000	0.963926000	
C	-6.303394000	-1.329551000	1.486563000	
C	-6.936013000	-2.289102000	0.631583000	
C	-7.298281000	-1.843252000	-0.705271000	
C	-6.217363000	-1.599169000	2.878751000	
C	-6.630287000	-2.870086000	3.364085000	
C	-7.164913000	-3.808077000	2.513576000	
C	-7.349947000	-3.502296000	1.148595000	
C	-5.736794000	-0.583624000	3.747980000	
C	-5.391122000	0.642669000	3.242809000	
C	-5.428188000	0.889115000	1.849508000	
C	-0.734369000	-2.881406000	0.324729000	
C	0.346715000	-2.182650000	0.801272000	
C	0.366068000	-0.771253000	0.740063000	
H	0.145267000	1.882177000	0.521662000	

H	-0.778485000	1.144223000	-2.301168000	
H	8.038356000	-3.661869000	-2.199428000	
H	5.898728000	-4.757513000	-2.802783000	
H	3.397067000	-4.618900000	-3.021431000	
H	1.334528000	-3.264428000	-2.893580000	
H	0.214108000	-1.098526000	-2.414749000	
H	4.054505000	4.236655000	-0.980557000	
H	5.910598000	2.747138000	-0.318401000	
H	9.333897000	0.505824000	-0.305349000	
H	9.280456000	-1.659069000	-1.462830000	
H	8.318422000	1.876166000	1.395163000	
H	3.821473000	1.681646000	5.215750000	
H	5.489879000	3.492642000	4.937944000	
H	7.145220000	3.388697000	3.076277000	
H	2.883566000	-0.502366000	4.476997000	
H	2.887959000	-2.378680000	2.861098000	
H	4.261735000	-2.274729000	0.813206000	
H	1.721947000	4.542506000	-1.917020000	
H	-0.504792000	3.595566000	-2.281570000	
H	-7.154631000	3.982838000	-2.522702000	
H	-5.306279000	5.298866000	-1.520669000	
H	-3.052801000	5.318051000	-0.386068000	
H	-1.031696000	4.050171000	0.263853000	
H	-3.037555000	-3.951955000	-0.671308000	
H	-5.081713000	-2.709345000	-1.193824000	
H	-8.272057000	-0.632011000	-2.858113000	
H	-8.087789000	1.795602000	-3.206255000	
H	-8.126934000	-2.355639000	-1.203207000	
H	-6.525044000	-3.084643000	4.430376000	
H	-7.482416000	-4.779808000	2.897424000	
H	-7.851778000	-4.220366000	0.494991000	
H	-5.669640000	-0.787689000	4.819215000	
H	-5.056237000	1.438826000	3.910873000	
H	-5.105628000	1.864834000	1.490799000	
H	-0.737750000	-3.974072000	0.325017000	
H	1.220341000	-2.712735000	1.184604000	
H	1.271084000	-0.238452000	1.045952000	

nonsym-(*P*,*R*,*R*,*M*)-(c-**NC**)$_2$

C	-4.091114000	2.416809000	-0.018479000	- Thermochemistry -
C	-5.376336000	1.878354000	-0.261759000	-------------------
C	-3.944839000	3.758555000	0.409375000	Temperature 298.150 Kelvin. Pressure 1.00000 Atm.
C	-6.463083000	2.741972000	-0.355932000	
C	-6.308444000	4.098598000	0.027002000	Zero-point correction= 0.876049
C	-5.095398000	4.581190000	0.458599000	(Hartree/Particle)
C	-2.634612000	4.236247000	0.739328000	Thermal correction to Energy= 0.921423
C	-2.921520000	1.632386000	-0.298010000	Thermal correction to Enthalpy= 0.922368
C	-1.647904000	2.153345000	-0.040625000	Thermal correction to Gibbs Free Energy= 0.799906
C	-1.533500000	3.455573000	0.547230000	Sum of electronic and zero-point Energies= -
C	-0.497823000	1.387889000	-0.400428000	2762.921895
C	-0.612296000	0.213779000	-1.090366000	Sum of electronic and thermal Energies= -2762.876520
C	-1.907218000	-0.240409000	-1.507237000	Sum of electronic and thermal Enthalpies= -
C	-3.063416000	0.405784000	-1.028507000	2762.875576
C	-2.025405000	-1.315332000	-2.435749000	Sum of electronic and thermal Free Energies= -
C	-3.289716000	-1.717107000	-2.824294000	2762.998038
C	-4.443458000	-1.146590000	-2.250759000	
C	-4.349929000	-0.125260000	-1.328640000	Charge = 0 Multiplicity = 1
C	-5.515711000	0.373465000	-0.436817000	
C	-6.896067000	-0.036484000	-0.941452000	
C	-7.865532000	0.979639000	-1.313153000	
C	-7.708766000	2.265521000	-0.948950000	

C	-5.249153000	-0.407360000	0.883400000	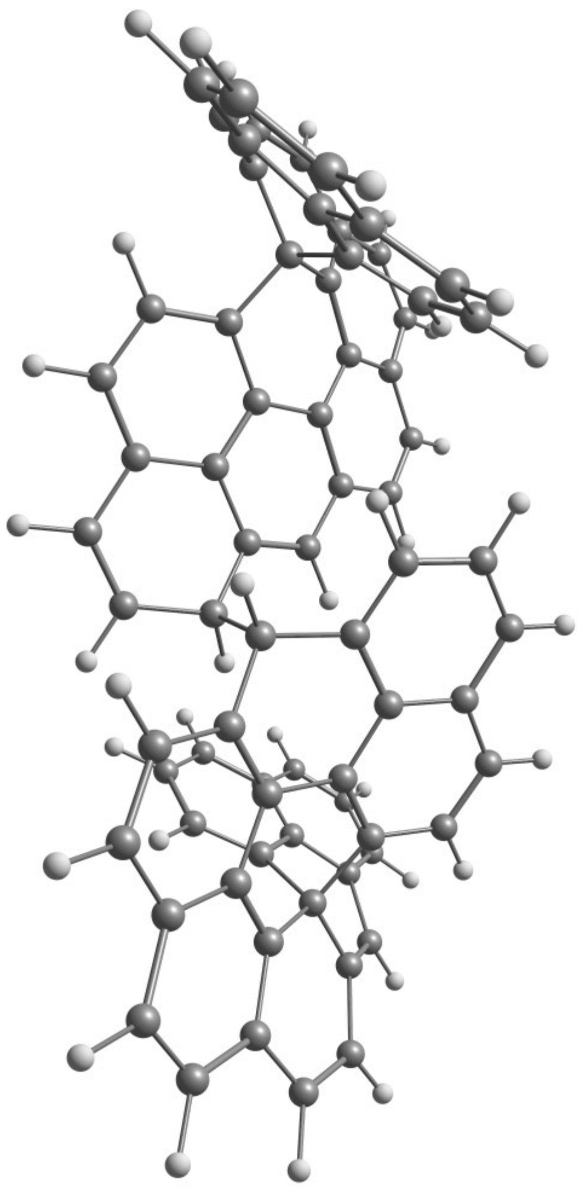
C	-5.599583000	-1.792042000	0.880081000	
C	-6.514576000	-2.309277000	-0.093569000	
C	-7.264655000	-1.336278000	-0.876014000	
C	-5.094364000	-2.659118000	1.884350000	
C	-5.388720000	-4.048267000	1.809836000	
C	-6.203725000	-4.540332000	0.818543000	
C	-6.797210000	-3.662090000	-0.113773000	
C	-4.329830000	-2.106512000	2.947316000	
C	-4.118474000	-0.753087000	3.006752000	
C	-4.567387000	0.098363000	1.967807000	
C	-0.798503000	-1.877524000	-3.004675000	
C	0.410001000	-1.559356000	-2.519365000	
C	0.563572000	-0.701491000	-1.294216000	
C	4.481326000	-2.346881000	-0.094840000	
C	5.664965000	-1.607670000	0.153336000	
C	4.547488000	-3.690892000	-0.541769000	
C	6.876254000	-2.278729000	0.226484000	
C	6.938483000	-3.647729000	-0.161303000	
C	5.821630000	-4.319633000	-0.587778000	
C	3.335370000	-4.346762000	-0.890141000	
C	3.213342000	-1.748461000	0.154104000	
C	2.049621000	-2.404280000	-0.201252000	
C	2.125676000	-3.706053000	-0.755281000	
C	0.749210000	-1.663377000	-0.026538000	
C	0.742280000	-0.866824000	1.253242000	
C	1.952489000	-0.231180000	1.652357000	
C	3.176089000	-0.547172000	0.994174000	
C	1.943791000	0.728117000	2.698127000	
C	3.162630000	1.379029000	3.020960000	
C	4.316144000	1.116510000	2.319505000	
C	4.335500000	0.156933000	1.281726000	
C	5.546343000	-0.102852000	0.344976000	
C	6.847238000	0.533941000	0.822525000	
C	7.989814000	-0.302653000	1.145175000	
C	8.040877000	-1.598850000	0.784359000	
C	5.102517000	0.643658000	-0.943230000	
C	5.172931000	2.068435000	-0.888682000	
C	6.012306000	2.717229000	0.074671000	
C	6.967945000	1.881055000	0.788487000	
C	4.459868000	2.854339000	-1.832028000	
C	4.480557000	4.269669000	-1.704360000	
C	5.231720000	4.875229000	-0.724388000	
C	6.027514000	4.098237000	0.143954000	
C	3.762897000	2.199000000	-2.883956000	
C	3.825253000	0.834201000	-3.001578000	
C	4.495290000	0.051879000	-2.028457000	
C	0.720183000	0.993478000	3.371578000	
C	-0.426807000	0.329806000	3.011897000	
C	-0.418991000	-0.596608000	1.942244000	
H	-0.101629000	-2.361233000	-0.036007000	
H	1.488489000	-0.106130000	-1.360463000	
H	-7.177355000	4.759699000	-0.019392000	
H	-4.997886000	5.617449000	0.790977000	
H	-2.531178000	5.251333000	1.130629000	
H	-0.537300000	3.831628000	0.791813000	
H	0.489890000	1.747868000	-0.098633000	
H	-3.399648000	-2.508814000	-3.569531000	
H	-5.420354000	-1.539481000	-2.531047000	
H	-8.793847000	0.645277000	-1.783129000	
H	-8.501304000	2.994797000	-1.133325000	

H -8.235531000 -1.644460000 -1.275366000<br>H -4.965800000 -4.717123000 2.563377000<br>H -6.427436000 -5.608132000 0.771936000<br>H -7.513140000 -4.049645000 -0.843076000<br>H -3.942435000 -2.768053000 3.725716000<br>H -3.577169000 -0.320354000 3.850739000<br>H -4.333178000 1.160181000 2.029211000<br>H -0.895759000 -2.554536000 -3.857662000<br>H 1.313614000 -1.983190000 -2.964192000<br>H 7.904964000 -4.156752000 -0.127967000<br>H 5.893519000 -5.356103000 -0.925959000<br>H 3.376352000 -5.373808000 -1.260923000<br>H 1.200722000 -4.212518000 -1.041046000<br>H 3.174307000 2.108962000 3.834052000<br>H 5.232087000 1.647600000 2.576220000<br>H 8.863211000 0.183918000 1.586666000<br>H 8.951361000 -2.182146000 0.941405000<br>H 7.878052000 2.355051000 1.167901000<br>H 3.898744000 4.870473000 -2.407639000<br>H 5.244879000 5.963748000 -0.638419000<br>H 6.686128000 4.592280000 0.862785000<br>H 3.205936000 2.797707000 -3.608398000<br>H 3.329039000 0.332022000 -3.834923000<br>H 4.487138000 -1.031546000 -2.136015000<br>H 0.707081000 1.731600000 4.177239000<br>H -1.365220000 0.537261000 3.529248000<br>H -1.359644000 -1.060967000 1.632301000	
(*P*,*P*)-**HC**	
C 4.873106000 -1.634190000 1.510255000<br>C 6.117954000 -1.287478000 0.928828000<br>C 4.830950000 -2.455449000 2.663058000<br>C 7.263120000 -1.967894000 1.325382000<br>C 7.210401000 -2.824564000 2.456977000<br>C 6.039021000 -3.019283000 3.144402000<br>C 3.562826000 -2.736835000 3.246976000<br>C 3.650437000 -1.232177000 0.877402000<br>C 2.421412000 -1.492790000 1.480917000<br>C 2.409750000 -2.258264000 2.691756000<br>C 1.188074000 -1.141125000 0.776108000<br>C 1.253545000 -0.975142000 -0.630376000<br>C 2.531129000 -0.781270000 -1.267193000<br>C 3.718978000 -0.774339000 -0.497726000<br>C 2.603282000 -0.534644000 -2.661509000<br>C 3.856038000 -0.311215000 -3.246488000<br>C 5.007496000 -0.264357000 -2.473713000<br>C 4.951929000 -0.454500000 -1.092291000<br>C 6.144278000 -0.200629000 -0.134108000<br>C 7.492222000 -0.105011000 -0.844781000<br>C 8.536633000 -1.072715000 -0.560739000<br>C 8.473123000 -1.878194000 0.515007000<br>C 5.814501000 1.208769000 0.433553000<br>C 6.057736000 2.313291000 -0.438533000<br>C 6.933458000 2.174575000 -1.563801000<br>C 7.760371000 0.977373000 -1.610311000<br>C 5.490170000 3.583869000 -0.156535000<br>C 5.680595000 4.645391000 -1.082673000<br>C 6.456835000 4.469458000 -2.203667000<br>C 7.115131000 3.241050000 -2.424633000<br>C 4.766526000 3.759133000 1.054044000<br>C 4.653773000 2.720835000 1.941761000	- Thermochemistry -<br>-------------------<br>Temperature 298.150 Kelvin. Pressure 1.00000 Atm.<br><br>Zero-point correction= 0.832111 (Hartree/Particle)<br>Thermal correction to Energy= 0.875959<br>Thermal correction to Enthalpy= 0.876903<br>Thermal correction to Gibbs Free Energy= 0.759191<br>Sum of electronic and zero-point Energies= -2760.587655<br>Sum of electronic and thermal Energies= -2760.543807<br>Sum of electronic and thermal Enthalpies= -2760.542863<br>Sum of electronic and thermal Free Energies= -2760.660575<br><br>Charge = 0 Multiplicity = 1

C	5.173379000	1.440803000	1.629853000	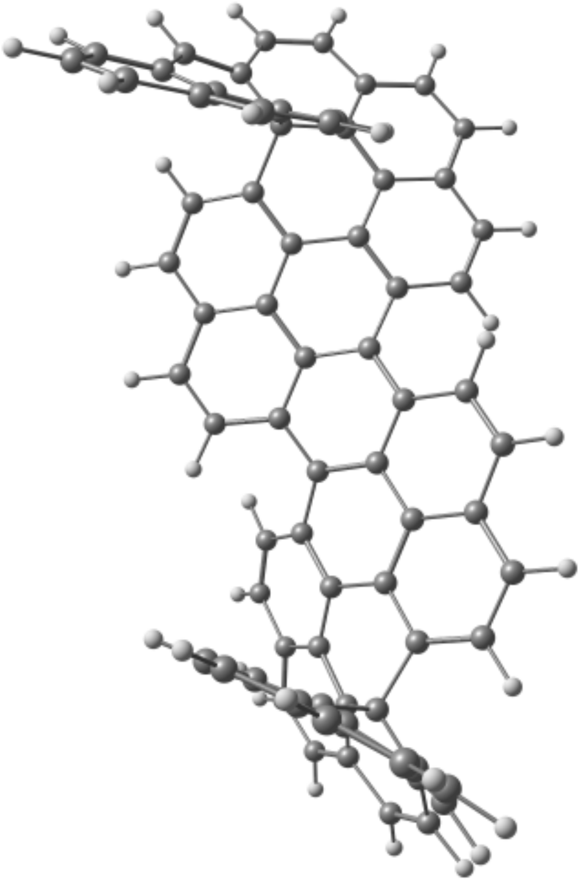
C	1.382795000	-0.476285000	-3.416949000	
C	0.179936000	-0.674456000	-2.824567000	
C	0.063162000	-0.958158000	-1.413916000	
C	-4.873123000	-1.634060000	-1.510366000	
C	-6.117969000	-1.287399000	-0.928904000	
C	-4.830971000	-2.455224000	-2.663236000	
C	-7.263139000	-1.967775000	-1.325517000	
C	-7.210422000	-2.824354000	-2.457181000	
C	-6.039043000	-3.019020000	-3.144623000	
C	-3.562848000	-2.736562000	-3.247180000	
C	-3.650453000	-1.232099000	-0.877482000	
C	-2.421429000	-1.492659000	-1.481022000	
C	-2.409771000	-2.258035000	-2.691923000	
C	-1.188090000	-1.141053000	-0.776185000	
C	-1.253559000	-0.975204000	0.630316000	
C	-2.531143000	-0.781394000	1.267153000	
C	-3.718992000	-0.774389000	0.497688000	
C	-2.603294000	-0.534905000	2.661494000	
C	-3.856049000	-0.311534000	3.246495000	
C	-5.007508000	-0.264598000	2.473726000	
C	-4.951943000	-0.454606000	1.092284000	
C	-6.144285000	-0.200636000	0.134117000	
C	-7.492242000	-0.105040000	0.844772000	
C	-8.536660000	-1.072713000	0.560654000	
C	-8.473146000	-1.878124000	-0.515143000	
C	-5.814463000	1.208786000	-0.433448000	
C	-6.057713000	2.313268000	0.438685000	
C	-6.933474000	2.174508000	1.563917000	
C	-7.760399000	0.977311000	1.610347000	
C	-5.490117000	3.583851000	0.156770000	
C	-5.680560000	4.645330000	1.082953000	
C	-6.456842000	4.469354000	2.203911000	
C	-7.115162000	3.240943000	2.424795000	
C	-4.766423000	3.759162000	-1.053772000	
C	-4.653647000	2.720904000	-1.941535000	
C	-5.173286000	1.440866000	-1.629709000	
C	-1.382807000	-0.476623000	3.416939000	
C	-0.179948000	-0.674736000	2.824536000	
C	-0.063177000	-0.958295000	1.413857000	
H	8.126505000	-3.326430000	2.777931000	
H	6.015091000	-3.651821000	4.034831000	
H	3.518298000	-3.380057000	4.129036000	
H	1.454875000	-2.547219000	3.129529000	
H	3.918912000	-0.141566000	-4.323972000	
H	5.963178000	-0.058061000	-2.953665000	
H	9.439164000	-1.038431000	-1.176108000	
H	9.316880000	-2.520089000	0.779149000	
H	8.705918000	1.032218000	-2.158031000	
H	5.208059000	5.609944000	-0.881589000	
H	6.599499000	5.292538000	-2.906926000	
H	7.802222000	3.138395000	-3.268453000	
H	4.326802000	4.734606000	1.274860000	
H	4.133520000	2.863036000	2.891184000	
H	5.026027000	0.633502000	2.345333000	
H	1.437898000	-0.227761000	-4.479496000	
H	-0.728439000	-0.553071000	-3.413433000	
H	-8.126528000	-3.326189000	-2.778178000	
H	-6.015115000	-3.651484000	-4.035104000	
H	-3.518322000	-3.379713000	-4.129292000	
H	-1.454899000	-2.546959000	-3.129721000	

H	-3.918923000	-0.141992000	4.323996000	
H	-5.963189000	-0.058350000	2.953701000	
H	-9.439201000	-1.038454000	1.176009000	
H	-9.316909000	-2.519989000	-0.779340000	
H	-8.705961000	1.032140000	2.158043000	
H	-5.208002000	5.609886000	0.881933000	
H	-6.599518000	5.292400000	2.907205000	
H	-7.802282000	3.138256000	3.268588000	
H	-4.326675000	4.734639000	-1.274523000	
H	-4.133351000	2.863142000	-2.890928000	
H	-5.025914000	0.633592000	-2.345218000	
H	-1.437909000	-0.228207000	4.479511000	
H	0.728428000	-0.553415000	3.413415000	

(*P*,*M*)-**HC**

C	-4.575494000	-2.377476000	0.061165000	- Thermochemistry -
C	-5.879969000	-1.950444000	-0.294087000	-------------------
C	-4.337227000	-3.733912000	0.388832000	Temperature 298.150 Kelvin. Pressure 1.00000 Atm.
C	-6.858046000	-2.902769000	-0.555353000	
C	-6.606008000	-4.266719000	-0.249721000	Zero-point correction= 0.832181
C	-5.396477000	-4.666797000	0.258595000	(Hartree/Particle)
C	-3.015943000	-4.117259000	0.753187000	Thermal correction to Energy= 0.875992
C	-3.475238000	-1.458479000	-0.010003000	Thermal correction to Enthalpy= 0.876936
C	-2.200528000	-1.835799000	0.411073000	Thermal correction to Gibbs Free Energy= 0.759362
C	-1.997691000	-3.206513000	0.777328000	Sum of electronic and zero-point Energies= -
C	-1.093243000	-0.878409000	0.291696000	2760.582402
C	-1.231512000	0.166399000	-0.658114000	Sum of electronic and thermal Energies= -2760.538591
C	-2.521480000	0.439485000	-1.243629000	Sum of electronic and thermal Enthalpies= -
C	-3.667745000	-0.246733000	-0.783453000	2760.537647
C	-2.666010000	1.438686000	-2.238472000	Sum of electronic and thermal Free Energies= -
C	-3.945252000	1.740178000	-2.719968000	2760.655221
C	-5.070878000	1.125928000	-2.187063000	
C	-4.951114000	0.151803000	-1.196379000	Charge = 0 Multiplicity = 1
C	-6.145669000	-0.458564000	-0.421031000	
C	-7.501278000	-0.149248000	-1.050294000	
C	-8.334505000	-1.226228000	-1.552403000	
C	-8.086107000	-2.510841000	-1.238825000	
C	-6.080859000	0.290915000	0.937943000	
C	-6.541034000	1.642520000	0.932955000	
C	-7.391490000	2.118200000	-0.117472000	
C	-7.981064000	1.114098000	-0.991456000	
C	-6.220185000	2.509498000	2.010357000	
C	-6.620670000	3.871709000	1.941684000	
C	-7.365119000	4.332780000	0.881663000	
C	-7.783158000	3.444070000	-0.131645000	
C	-5.526471000	1.981254000	3.132855000	
C	-5.207860000	0.648709000	3.177461000	
C	-5.479883000	-0.198778000	2.075723000	
C	-1.479775000	2.077448000	-2.737441000	
C	-0.276679000	1.861528000	-2.154479000	
C	-0.122639000	0.991166000	-1.009316000	
C	4.575487000	2.377471000	-0.061143000	
C	5.879962000	1.950443000	0.294112000	
C	4.337215000	3.733908000	-0.388801000	
C	6.858034000	2.902770000	0.555388000	
C	6.605991000	4.266722000	0.249764000	
C	5.396460000	4.666797000	-0.258553000	
C	3.015931000	4.117252000	-0.753157000	
C	3.475235000	1.458468000	0.010014000	
C	2.200524000	1.835785000	-0.411064000	
C	1.997682000	3.206501000	-0.777306000	

C	1.093243000	0.878388000	-0.291696000	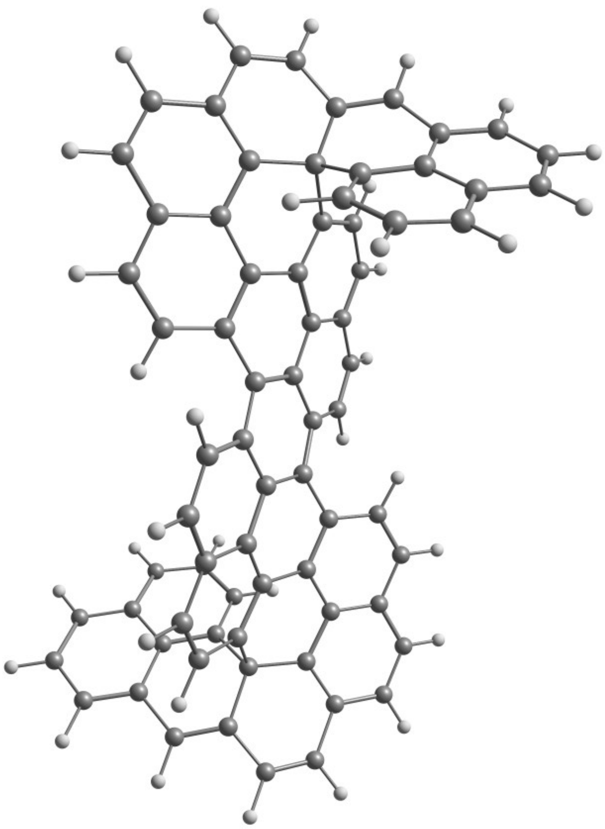
C	1.231514000	-0.166423000	0.658112000	
C	2.521482000	-0.439507000	1.243627000	
C	3.667745000	0.246719000	0.783459000	
C	2.666013000	-1.438714000	2.238464000	
C	3.945255000	-1.740201000	2.719965000	
C	5.070879000	-1.125941000	2.187068000	
C	4.951115000	-0.151812000	1.196387000	
C	6.145667000	0.458564000	0.421044000	
C	7.501282000	0.149248000	1.050297000	
C	8.334501000	1.226226000	1.552421000	
C	8.086096000	2.510842000	1.238858000	
C	6.080852000	-0.290898000	-0.937939000	
C	6.541044000	-1.642498000	-0.932976000	
C	7.391515000	-2.118185000	0.117434000	
C	7.981082000	-1.114091000	0.991433000	
C	6.220194000	-2.509462000	-2.010390000	
C	6.620697000	-3.871669000	-1.941744000	
C	7.365164000	-4.332749000	-0.881738000	
C	7.783201000	-3.444050000	0.131580000	
C	5.526459000	-1.981209000	-3.132871000	
C	5.207827000	-0.648669000	-3.177451000	
C	5.479852000	0.198804000	-2.075702000	
C	1.479781000	-2.077488000	2.737423000	
C	0.276685000	-1.861568000	2.154463000	
C	0.122642000	-0.991192000	1.009311000	
H	-7.397314000	-4.999009000	-0.427839000	
H	-5.219710000	-5.713799000	0.515668000	
H	-2.811848000	-5.168055000	0.972071000	
H	-0.990057000	-3.559422000	0.988375000	
H	-4.056716000	2.491295000	-3.505448000	
H	-6.057898000	1.413413000	-2.547420000	
H	-9.244395000	-0.952550000	-2.092418000	
H	-8.783301000	-3.298957000	-1.532994000	
H	-8.929366000	1.356883000	-1.480184000	
H	-6.336521000	4.545209000	2.753938000	
H	-7.670525000	5.380360000	0.840449000	
H	-8.450999000	3.797970000	-0.921206000	
H	-5.276007000	2.644486000	3.964163000	
H	-4.711454000	0.231137000	4.055813000	
H	-5.173251000	-1.241898000	2.134585000	
H	-1.562321000	2.736195000	-3.605206000	
H	0.610005000	2.322913000	-2.585224000	
H	7.397294000	4.999014000	0.427890000	
H	5.219689000	5.713800000	-0.515619000	
H	2.811830000	5.168048000	-0.972032000	
H	0.990047000	3.559409000	-0.988350000	
H	4.056720000	-2.491322000	3.505441000	
H	6.057899000	-1.413421000	2.547430000	
H	9.244394000	0.952548000	2.092432000	
H	8.783286000	3.298958000	1.533036000	
H	8.929389000	-1.356875000	1.480151000	
H	6.336548000	-4.545159000	-2.754006000	
H	7.670583000	-5.380325000	-0.840545000	
H	8.451053000	-3.797955000	0.921130000	
H	5.275994000	-2.644432000	-3.964186000	
H	4.711403000	-0.231090000	-4.055789000	
H	5.173202000	1.241920000	-2.134541000	
H	1.562329000	-2.736247000	3.605179000	
H	-0.609997000	-2.322966000	2.585199000	

S12. Copies of HR-MS data

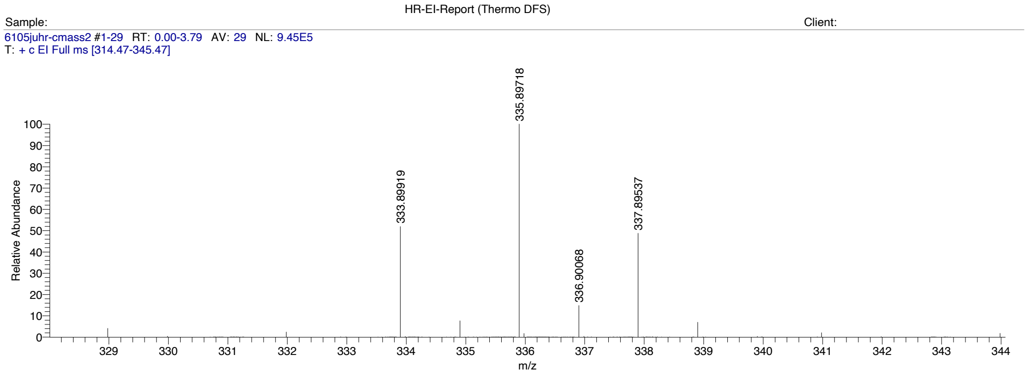

6105juhr-cmass2#1-29 RT: 0.00-3.79 AV: 29
T: + c EI Full ms [314.47-345.47]
m/z= 333.46076-334.17786

m/z	Intensity	Relative	Theo. Mass	Delta (ppm)	Composition
333.89919	490545.9	100.00	333.89873	1.40	$C_{14}H_8Br_2$

Figure S126. (+)-HR-EI-MS of **2**.

Mass Spectrum SmartFormula Report

Analysis Info

Analysis Name	E:\new acq data for data analysis\PR231 002.d	Acquisition Date	19.10.2016 15:03:04
Method	hn Direct_Infusion_pos mode_75-1700 low 4eV.m	Operator	hn
Sample Name	Prince Ravat	Instrument / Ser#	maXis 4G 21243
Comment	PR231, ca 50 ug/ml MeCN/DCM 9:1, mit MeCN/TFA		

Acquisition Parameter

Source Type	ESI	Ion Polarity	Positive	Set Nebulizer	0.4 Bar
Focus	Not active	Set Capillary	3600 V	Set Dry Heater	180 °C
Scan Begin	75 m/z	Set End Plate Offset	-500 V	Set Dry Gas	3.0 l/min
Scan End	1700 m/z	Set Collision Cell RF	350.0 Vpp	Set Ion Energy (MS only)	4.0 eV

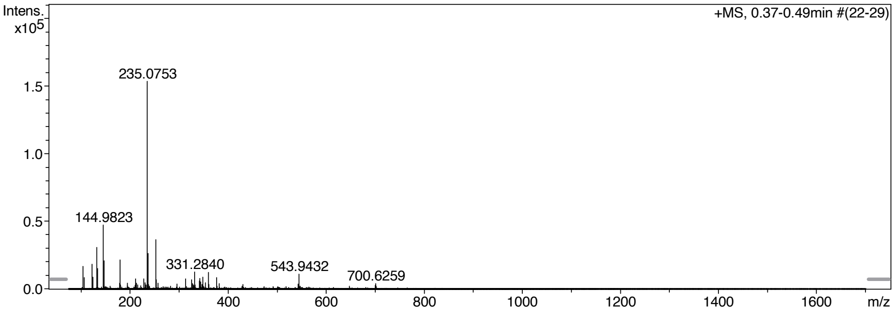

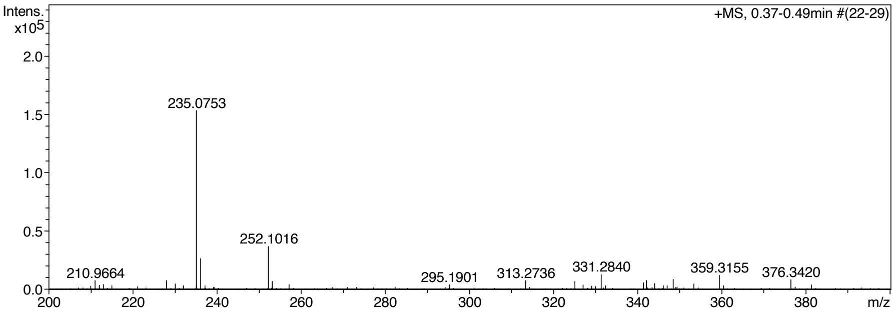

Meas. m/z	#	Formula	Score	m/z	err [mDa]	err [ppm]	mSigma	rdb	e⁻ Conf	N-Rule	z
235.0753	1	C 16 H 11 O 2	100.00	235.0754	0.1	0.4	2.0	11.5	even	ok	1+
252.1016	1	C 16 H 14 N O 2	100.00	252.1019	0.3	1.1	3.8	10.5	even	ok	

Bruker Compass DataAnalysis 4.0 printed: 19.10.2016 15:10:28 Page 1 of 1

Figure S127. (+)-HR-ESI-MS of **3**.

D:\Data\Service\Data\21_juQEx_0373 03/08/21 10:25:15 Sample: DC532
Client: (+)-HR-ESI-MS Solvent: MeOH/CHCl3 3:2

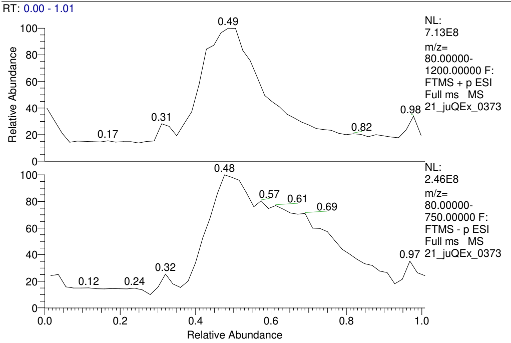

21_juQEx_0373#41-47 RT: 0.41-0.47 AV: 4
SB: 25 0.04-0.25 , 0.72-0.98
T: FTMS + p ESI Full lock ms [100.0000-1500.0000]
m/z= 554.67830-555.61210

m/z	Intensity	Relative	Theo. Mass	Delta (ppm)	RDB equiv.	Composition
555.25354	27365908.0	100.00	555.25349	0.07	9.0	$C_{24}H_{37}O_{10}N_5$
			555.25299	0.99	21.5	$C_{38}H_{35}O_4$
			555.25432	-1.42	26.5	$C_{39}H_{31}N_4$
			555.25164	3.41	22.0	$C_{36}H_{33}O_3N_3$

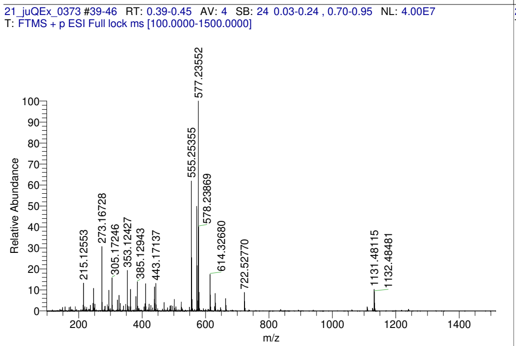

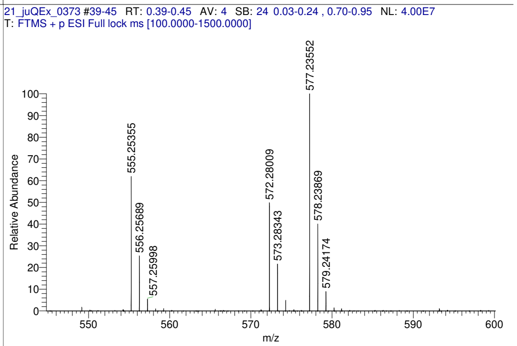

Figure S128. (+)-HR-ESI-MS of **4**.

Mass Spectrum SmartFormula Report

Analysis Info

Analysis Name	E:\new acq data for data analysis\PR235 002.d	Acquisition Date	03.11.2016 16:36:43
Method	hn Direct_Infusion_pos mode_75-1700 mid 4eV.m	Operator	hn
Sample Name	Prince Ravat	Instrument / Ser#	maXis 4G 21243
Comment	PR235, ca. 50 ug/ml MeCN		

Acquisition Parameter

Source Type	ESI	Ion Polarity	Positive	Set Nebulizer	0.4 Bar
Focus	Not active	Set Capillary	3600 V	Set Dry Heater	180 °C
Scan Begin	75 m/z	Set End Plate Offset	-500 V	Set Dry Gas	4.0 l/min
Scan End	1700 m/z	Set Collision Cell RF	350.0 Vpp	Set Ion Energy (MS only)	4.0 eV

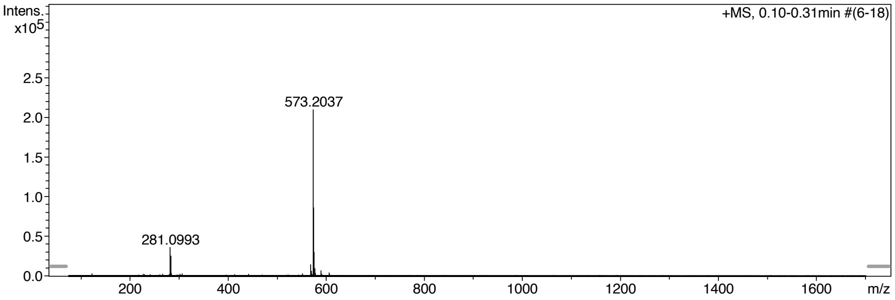

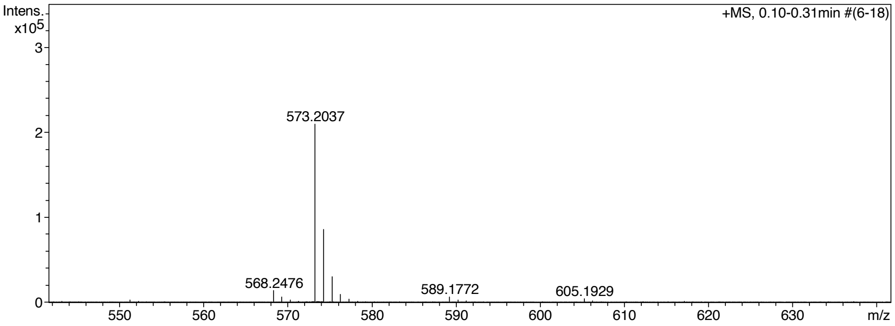

Meas. m/z	#	Formula	Score	m/z	err [mDa]	err [ppm]	mSigma	rdb	e⁻ Conf	z
568.2476	1	C 38 H 34 N O 4	100.00	568.2482	0.6	1.1	45.4	22.5	even	1+
573.2037	1	C 38 H 30 Na O 4	100.00	573.2036	-0.1	-0.1	30.0	23.5	even	
589.1772	1	C 38 H 30 K O 4	100.00	589.1776	0.4	0.7	30.4	23.5	even	

Bruker Compass DataAnalysis 4.0 printed: 03.11.2016 16:45:01 Page 1 of 1

Figure S129. (+)-HR-ESI-MS of **5**.

Mass Spectrum SmartFormula Report

Analysis Info

Analysis Name	E:\new acq data for data analysis\PR237 001.d	Acquisition Date	07.03.2017 17:03:06
Method	hn Direct_Infusion_pos mode_75-1700 mid 4eV.m	Operator	hn
Sample Name	Prince Ravat	Instrument / Ser#	maXis 4G 21243
Comment	PR237, ca. 50 ug/ml MeCN, mit MeCN/HCOOH		

Acquisition Parameter

Source Type	ESI	Ion Polarity	Positive	Set Nebulizer	0.4 Bar
Focus	Not active	Set Capillary	3600 V	Set Dry Heater	180 °C
Scan Begin	75 m/z	Set End Plate Offset	-500 V	Set Dry Gas	4.0 l/min
Scan End	1700 m/z	Collision Energy	8.0 eV	Set Ion Energy (MS only)	4.0 eV

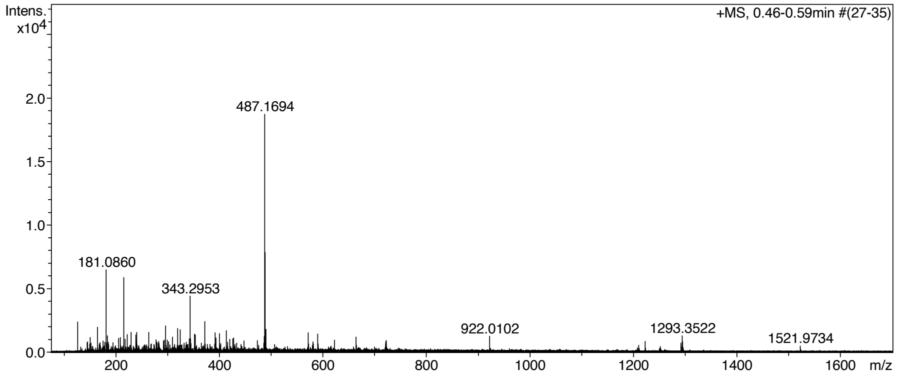

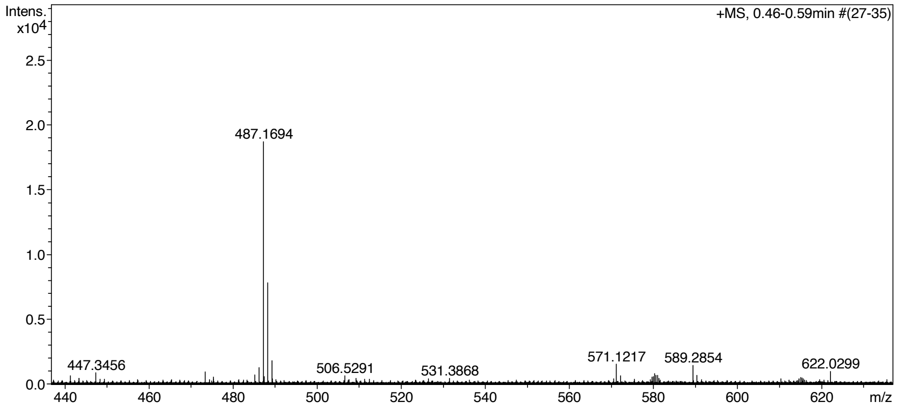

Meas. m/z	#	Formula	Score	m/z	err [mDa]	err [ppm]	mSigma	rdb	e⁻ Conf	z
487.1694	1	C 36 H 23 O 2	100.00	487.1693	-0.1	-0.3	17.4	25.5	even	1+

Bruker Compass DataAnalysis 4.0 printed: 07.03.2017 17:33:15 Page 1 of 1

Figure S130. (+)-HR-ESI-MS of **6**.

Mass Spectrum SmartFormula Report

Analysis Info

Analysis Name	E:\new acq data for data analysis\PR238 001.d	Acquisition Date	03.11.2016 14:25:51
Method	hn Direct_Infusion_pos mode_75-1700 mid 4eV.m	Operator	hn
Sample Name	Prince Ravat	Instrument / Ser#	maXis 4G 21243
Comment	PR238, ca. 50 ug/ml MeCN:DCM (9:1)		

Acquisition Parameter

Source Type	ESI	Ion Polarity	Positive	Set Nebulizer	0.4 Bar
Focus	Not active	Set Capillary	3600 V	Set Dry Heater	180 °C
Scan Begin	75 m/z	Set End Plate Offset	-500 V	Set Dry Gas	4.0 l/min
Scan End	1700 m/z	Set Collision Cell RF	350.0 Vpp	Set Ion Energy (MS only)	4.0 eV

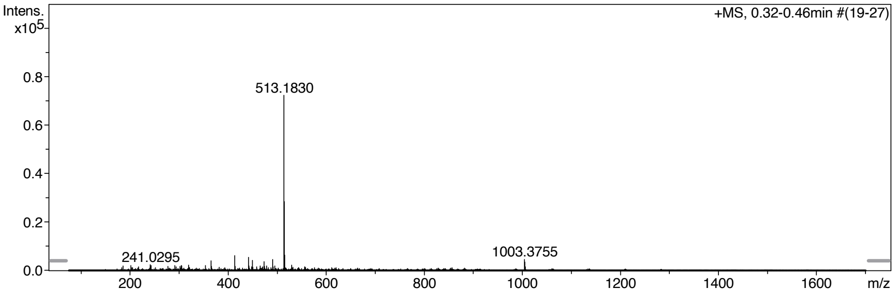

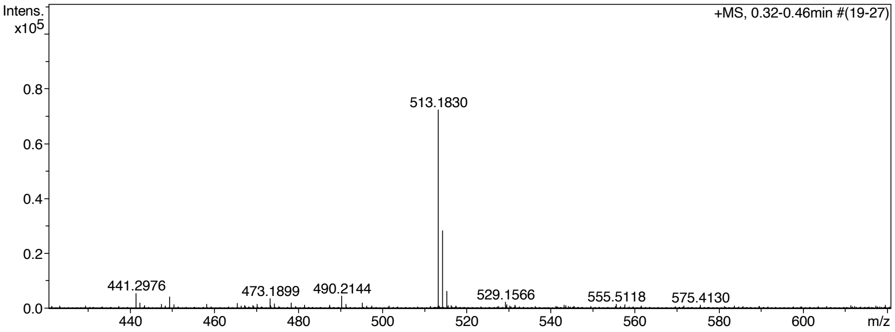

Meas. m/z	#	Formula	Score	m/z	err [mDa]	err [ppm]	mSigma	rdb	e⁻ Conf	z
473.1899	1	C 36 H 25 O	100.00	473.1900	0.1	0.1	42.3	24.5	even	1+
513.1830	1	C 36 H 26 Na O 2	100.00	513.1825	-0.5	-1.1	4.5	23.5	even	
529.1566	1	C 36 H 26 K O 2	100.00	529.1564	-0.2	-0.4	12.1	23.5	even	
1003.3755	1	C 72 H 52 Na O 4	100.00	1003.3758	0.3	0.3	12.6	46.5	even	

Bruker Compass DataAnalysis 4.0 printed: 03.11.2016 14:34:33 Page 1 of 1

Figure S131. (+)-HR-ESI-MS of **7**.

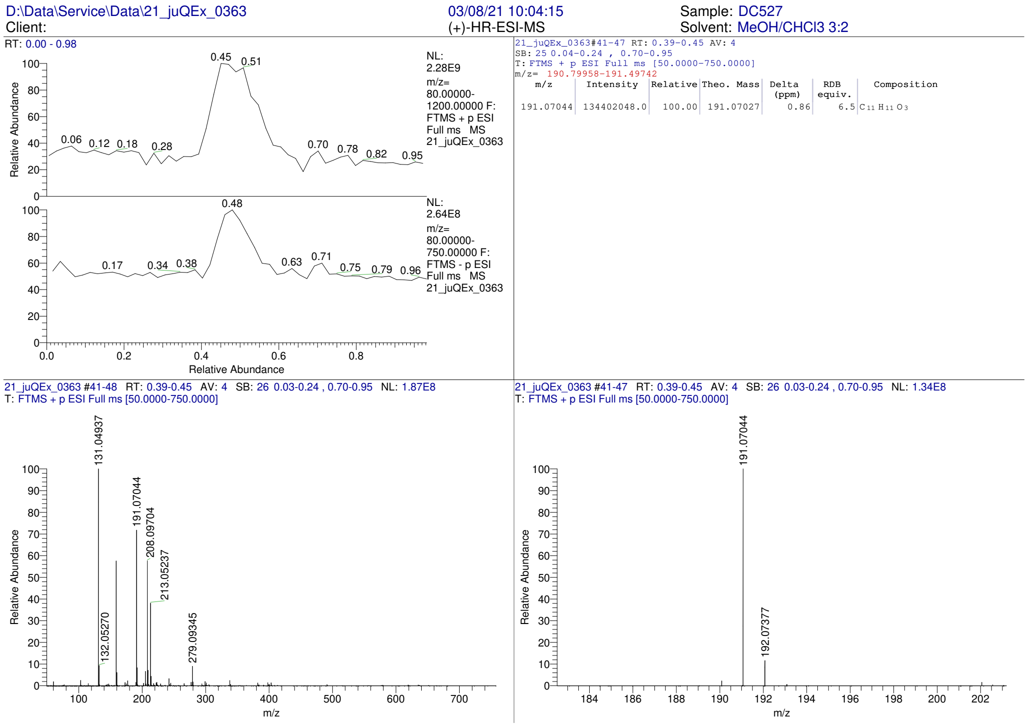

Figure S132. (+)-HR-ESI-MS of **8**.

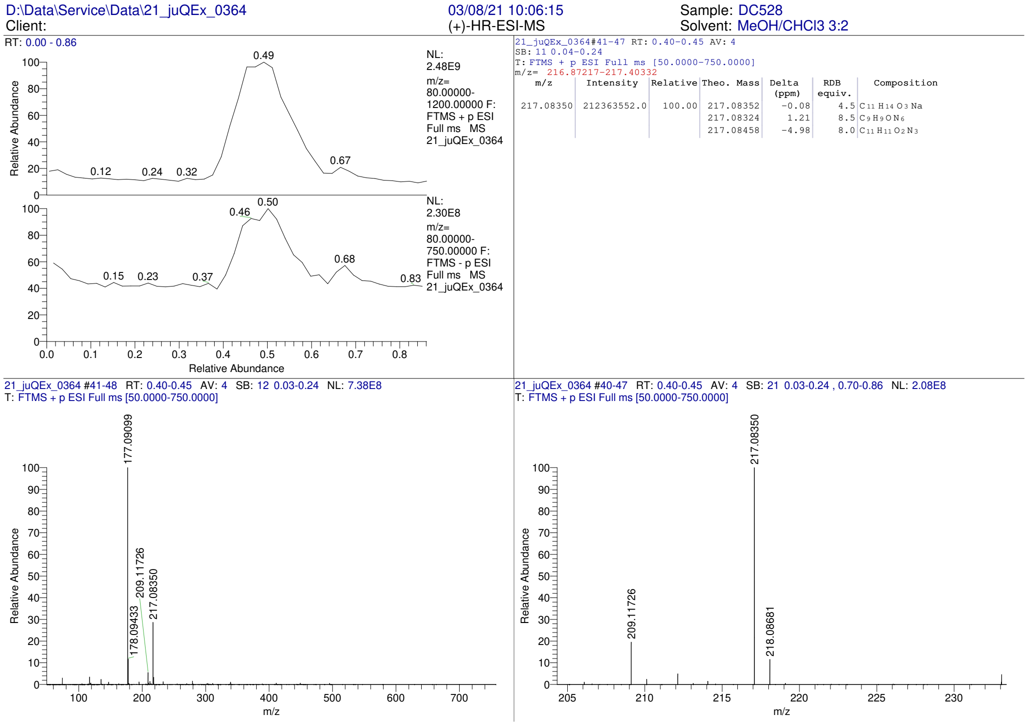

Figure S133. (+)-HR-ESI-MS of **9**.

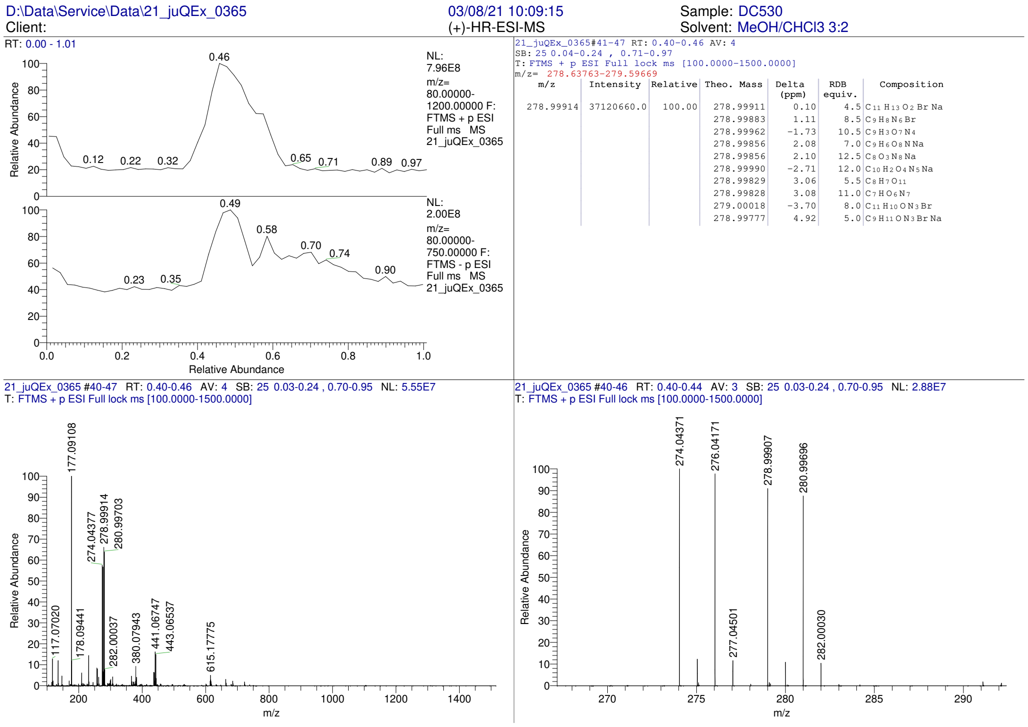

Figure S134. (+)-HR-ESI-MS of **10**.

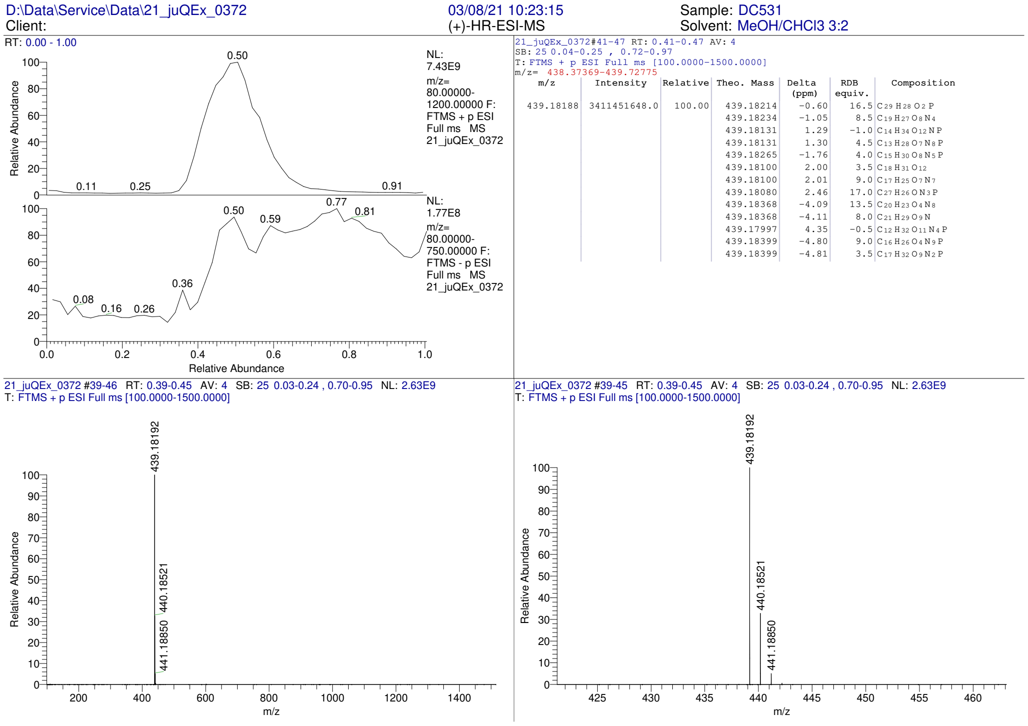

Figure S135. (+)-HR-ESI-MS of **11**.

Mass Spectrum SmartFormula Report

Analysis Info

Analysis Name	E:\new acq data for data analysis\PR236 001.d	Acquisition Date	03.11.2016 15:52:58
Method	hn Direct_Infusion_pos mode_75-1700 mid 4eV.m	Operator	hn
Sample Name	Prince Ravat	Instrument / Ser#	maXis 4G 21243
Comment	PR236, ca. 50 ug/ml MeOH		

Acquisition Parameter

Source Type	ESI	Ion Polarity	Positive	Set Nebulizer	0.4 Bar
Focus	Not active	Set Capillary	3600 V	Set Dry Heater	180 °C
Scan Begin	75 m/z	Set End Plate Offset	-500 V	Set Dry Gas	4.0 l/min
Scan End	1700 m/z	Set Collision Cell RF	350.0 Vpp	Set Ion Energy (MS only)	4.0 eV

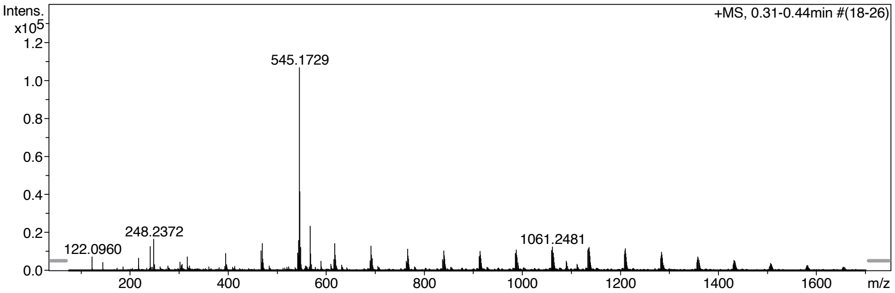

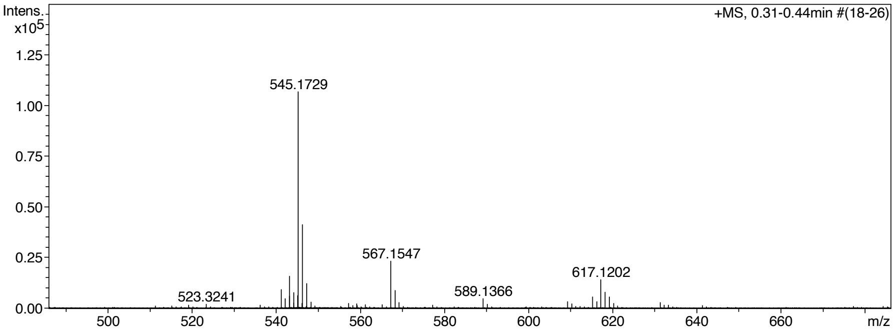

Meas. m/z	#	Formula	Score	m/z	err [mDa]	err [ppm]	mSigma	rdb	e⁻ Conf	z
545.1729	1	C 36 H 26 Na O 4	100.00	545.1723	-0.6	-1.1	17.3	23.5	even	1+
567.1547	1	C 36 H 25 Na 2 O 4	100.00	567.1543	-0.4	-0.7	23.9	23.5	even	

Bruker Compass DataAnalysis 4.0 printed: 03.11.2016 15:59:53 Page 1 of 1

Figure S136. (+)-HR-ESI-MS of **12**.

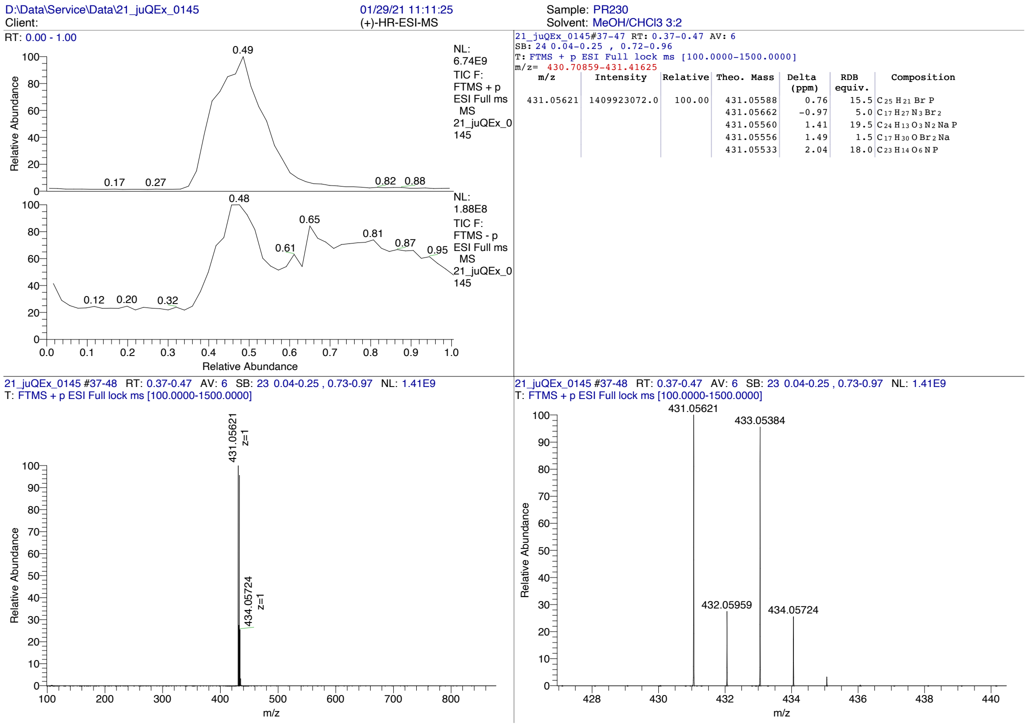

Figure S137. (+)-HR-ESI-MS of **13**.

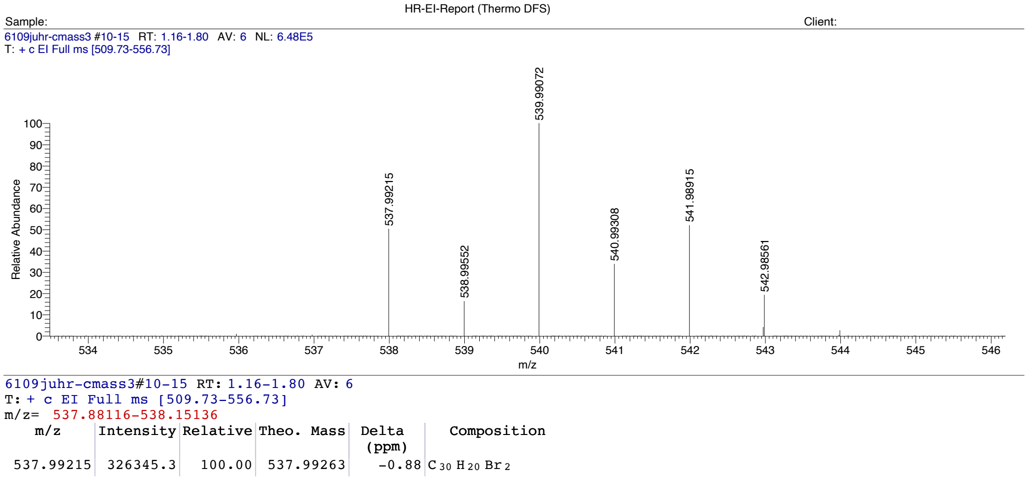

Figure S138. (+)-HR-EI-MS of **14**.

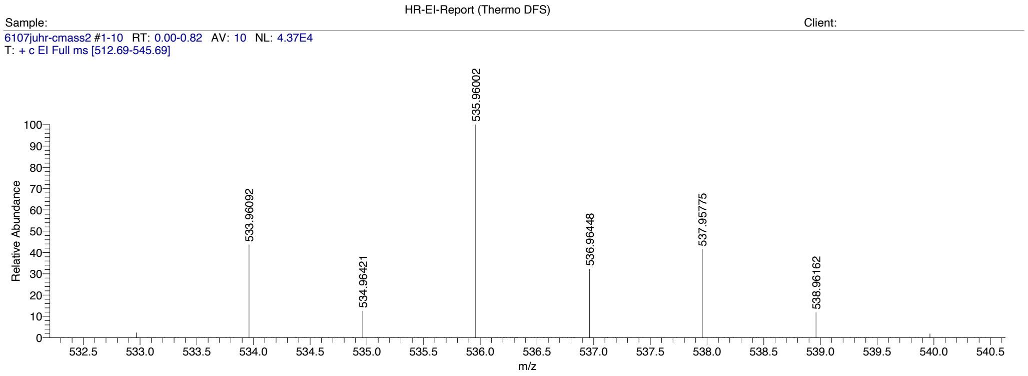

6107juhr-cmass2#1-10 RT: 0.00-0.82 AV: 10
T: + c EI Full ms [512.69-545.69]
m/z= 533.77729-534.06946

m/z	Intensity	Relative	Theo. Mass	Delta (ppm)	Composition
533.96092	19036.3	100.00	533.96133	-0.77	$C_{30}H_{16}Br_2$

Figure S139. (+)-HR-EI-MS of **15**.

Mass Spectrum SmartFormula Report

Analysis Info

Analysis Name	E:\new acq data for data analysis\PR239 001.d	Acquisition Date	16.01.2017 11:50:54
Method	hn Direct_Infusion_pos mode_75-1700 mid 4eV.m	Operator	hn
Sample Name	Prince Ravat	Instrument / Ser#	maXis 4G 21243
Comment	PR239, ca. 50 ug/ml MeCN		

Acquisition Parameter

Source Type	ESI	Ion Polarity	Positive	Set Nebulizer	0.4 Bar
Focus	Not active	Set Capillary	3600 V	Set Dry Heater	180 °C
Scan Begin	75 m/z	Set End Plate Offset	-500 V	Set Dry Gas	4.0 l/min
Scan End	1700 m/z	Collision Energy	8.0 eV	Set Ion Energy (MS only)	4.0 eV

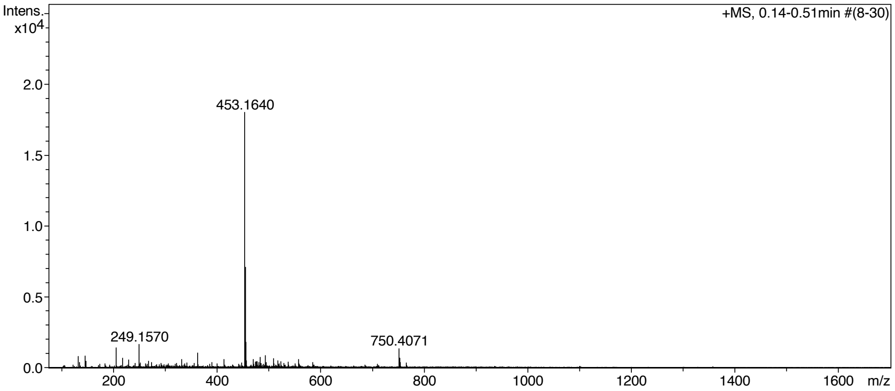

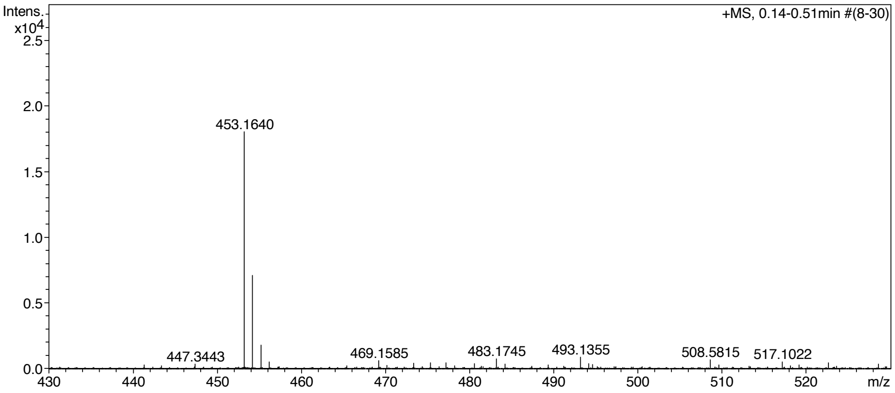

Meas. m/z	#	Formula	Score	m/z	err [mDa]	err [ppm]	mSigma	rdb	e⁻ Conf	z
453.1640	1	C 36 H 21	100.00	453.1638	-0.2	-0.4	15.5	26.5	even	1+

Bruker Compass DataAnalysis 4.0 printed: 16.01.2017 11:57:52 Page 1 of 1

Figure S140. (+)-HR-ESI-MS of 2*H*-**NC**.

Mass Spectrum SmartFormula Report

Analysis Info

Analysis Name	E:\new acq data for data analysis\PR234 002.d	Acquisition Date	08.03.2017 10:13:11
Method	hn Direct_Infusion_pos mode_75-1700 mid 4eV.m	Operator	hn
Sample Name	Prince Ravat	Instrument / Ser#	maXis 4G 21243
Comment	PR234, ca. 50 ug/ml MeCN		

Acquisition Parameter

Source Type	ESI	Ion Polarity	Positive	Set Nebulizer	0.4 Bar
Focus	Not active	Set Capillary	3600 V	Set Dry Heater	180 °C
Scan Begin	75 m/z	Set End Plate Offset	-500 V	Set Dry Gas	4.0 l/min
Scan End	1700 m/z	Collision Energy	58.0 eV	Set Ion Energy (MS only)	4.0 eV

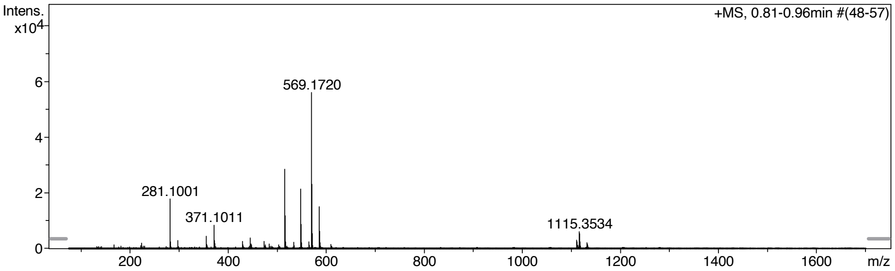

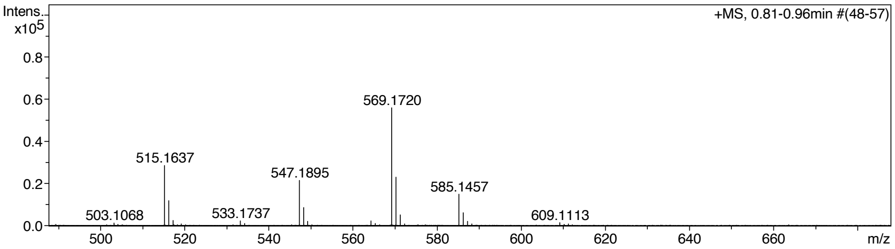

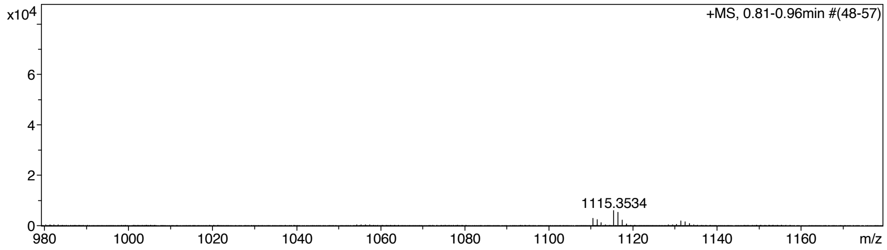

Meas. m/z	#	Formula	Score	m/z	err [mDa]	err [ppm]	mSigma	rdb	e⁻ Conf	z
515.1637	1	C 37 H 23 O 3	100.00	515.1642	0.5	1.0	6.2	26.5	even	1+
547.1895	1	C 38 H 27 O 4	100.00	547.1904	0.9	1.6	3.9	25.5	even	
564.2166	1	C 38 H 30 N O 4	100.00	564.2169	0.3	0.6	34.7	24.5	even	
569.1720	1	C 38 H 26 Na O 4	100.00	569.1723	0.4	0.6	1.4	25.5	even	
585.1457	1	C 38 H 26 K O 4	100.00	585.1463	0.6	1.0	11.4	25.5	even	
1110.3981	1	C 76 H 56 N O 8	100.00	1110.4000	1.9	1.7	25.0	49.5	even	
1115.3534	1	C 76 H 52 Na O 8	100.00	1115.3554	2.1	1.9	23.6	50.5	even	
1131.3263	1	C 76 H 52 K O 8	100.00	1131.3294	3.0	2.7	22.7	50.5	even	

Bruker Compass DataAnalysis 4.0 printed: 08.03.2017 10:32:29 Page 1 of 1

Figure S141. (+)-HR-ESI-MS of **16**.

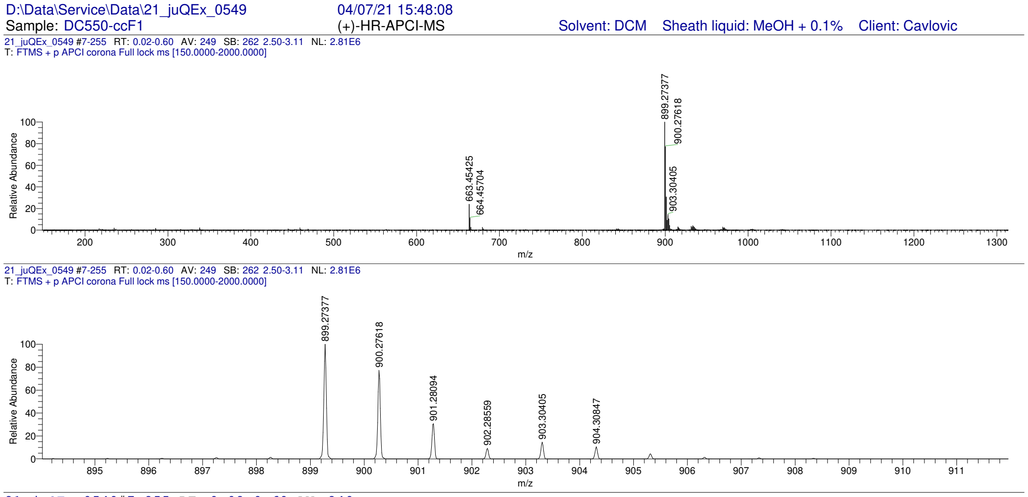

21_juQEx_0549#7-255 RT: 0.02-0.60 AV: 249
SB: 262 2.50-3.11
T: FTMS + p APCI corona Full lock ms [150.0000-2000.0000]
m/z= 899.11392-899.44955

m/z	Intensity	Relative	Theo. Mass	Delta (ppm)	RDB equiv.	Composition
899.27377	2814228.3	100.00	899.27333	0.44	55.5	$C_{72}H_{35}$

Figure S142. (+)-HR-APCI-MS of **HC**.

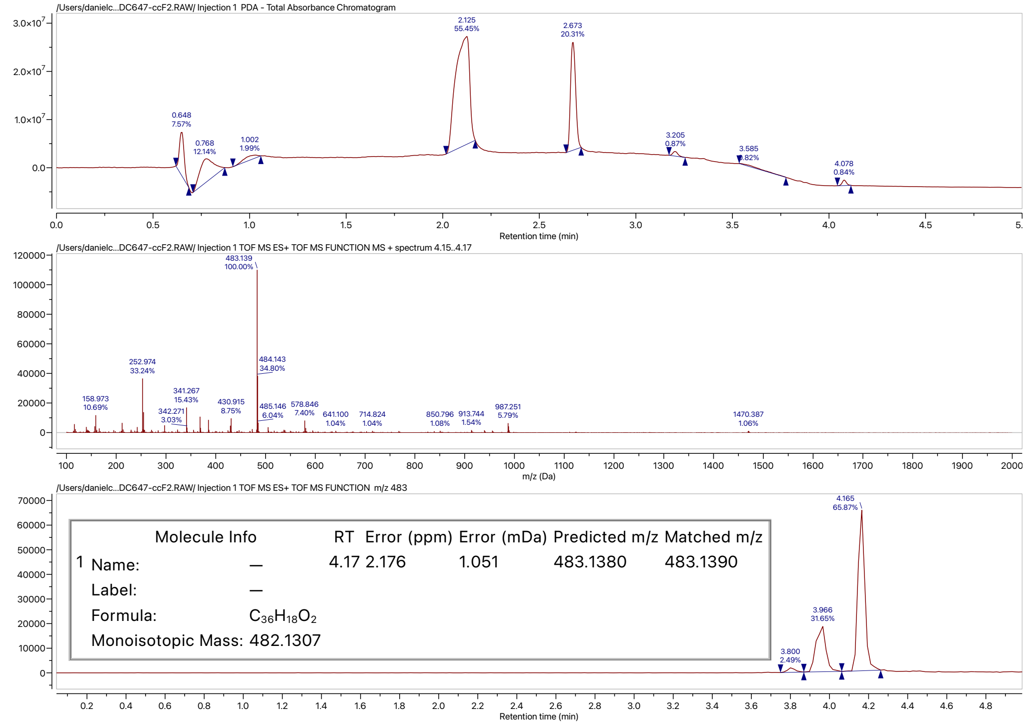

Figure S143. (+)-HR-ESI-MS of 2*O*-**NC**.

Mass Spectrum SmartFormula Report

Analysis Info

Analysis Name	E:\new acq data for data analysis\PR239_CAoxi 003.d	Acquisition Date	10.11.2016 16:02:21
Method	hn Direct_Infusion_pos mode_75-1700 mid 4eV.m	Operator	hn
Sample Name	Prince Ravat	Instrument / Ser#	maXis 4G 21243
Comment	PR239+CAoxi, ca. 50 ug/ml MeCN:DCM (9:1), with ACN/TFA		

Acquisition Parameter

Source Type	ESI	Ion Polarity	Positive	Set Nebulizer	0.4 Bar
Focus	Not active	Set Capillary	3600 V	Set Dry Heater	180 °C
Scan Begin	75 m/z	Set End Plate Offset	-500 V	Set Dry Gas	4.0 l/min
Scan End	1700 m/z	Set Collision Cell RF	350.0 Vpp	Set Ion Energy (MS only)	4.0 eV

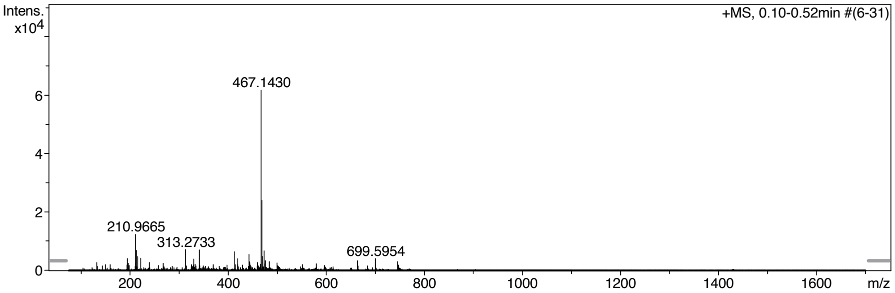

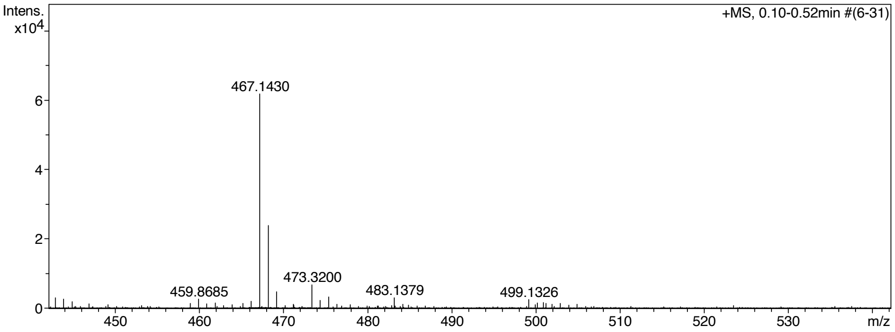

Meas. m/z	#	Formula	Score	m/z	err [mDa]	err [ppm]	mSigma	rdb	e⁻ Conf	z
467.1430	1	C 36 H 19 O	100.00	467.1430	0.0	0.1	2.4	27.5	even	1+

Figure S144. (+)-HR-ESI-MS of *O*-c-**NC**.

S13. References

(S1) Clark, R. C.; Reid, J. S. *Acta Cryst. A* **1995**, 51, 887.

(S2) *CrysAlisPro* (version 1.171.40.68a), Rigaku Oxford Diffraction Ltd, Yarnton, Oxfordshire, England, **2019**.

(S3) "*A complete structure solution, refinement and analysis program*" Dolomanov, O. V.; Bourhis, L. J.; Gildea, R. J.; Howard, J. A. K.; Puschmann, H. *J. Appl. Cryst.* **2009**, 42, 339.

(S4) "*SHELXT - Integrated space-group and crystal-structure determination*" Sheldrick, G. M. *Acta Cryst. A* **2015**, 71, 3.

(S5) "*Crystal structure refinement with SHELXL*" Sheldrick, G. M. *Acta Cryst. C* **2015**, 71, 3.

(S6) "*Structure validation in chemical crystallography*" Spek, A. L. *Acta Cryst. D* **2009**, *65*, 148.

(S7) Rigaku Oxford Diffraction, **2015**.

(S8) "*Single-crystal structure validation with the program PLATON*" Spek, A. L. *J. Appl. Crystallogr.* **2003**, *36*, 7.
